# Aroylhydrazone Diorganotin Complexes Causes DNA Damage and Apoptotic Cell Death: From Chemical Synthesis to Biochemical Effects

**DOI:** 10.3390/ijms222413525

**Published:** 2021-12-16

**Authors:** Wujiu Jiang, Yuxing Tan, Yiyuan Peng

**Affiliations:** 1 Key Laboratory of Functional Small Organic Molecule, Ministry of Education, Key Laboratory of Green Chemistry, College of Chemistry and Chemical Engineering, Jiangxi Normal University, Nanchang 330022, China; tanyuxing@hynu.edu.cn; 2 Hunan Provincial Engineering Research Center for Monitoring and Treatment of Heavy Metals Pollution in the Upper Reaches of XiangJiang River, Key Laboratory of Functional Metal-Organic Compounds of Hunan Province, Key Laboratory of Functional Organometallic Materials, College of Chemistry and Materials Science, University of Hunan Province, Hengyang 421008, China

**Keywords:** diorganotin, synthesis, crystal structure, apoptosis, DNA

## Abstract

Under microwave irradiation, eighteen new aroylhydrazone diorganotin complexes (**1a**–**9b**) were produced through the reaction of aroylhydrazine, 2-ketobutyric acid, and the corresponding diorganotin. Fourier transform infrared spectroscopy, ^1^H, ^13^C, and ^119^Sn nuclear magnetic resonance spectroscopies, high-resolution mass spectroscopy, X-ray crystallography, and thermogravimetric analysis (TGA) were performed to characterize the complexes. The in vitro anticancer activity for complexes were assessed using a CCK-8 assay on human cancer cells of HepG2, NCI-H460, and MCF-7. Complex **4b** revealed more intensive anticancer activity against MCF-7 cells than the other complexes and cisplatin. Flow cytometry analysis and transmission electron microscope observation demonstrated that complex **4b** mediated cell apoptosis of MCF-7 cells and arrested cell cycle in S phase. Western blotting analysis showed that **4b** induced DNA damage in MCF-7 cells and led to apoptosis by the ATM-CHK2-p53 pathway. The single cell gel electrophoreses assay results showed that **4b** induced DNA damage. The DNA binding activity of **4b** was studied by UV–Visible absorption spectrometry, fluorescence competitive, viscosity measurements, gel electrophoresis, and molecular docking, and the results show that **4b** can be well embedded in the groove and cleave DNA.

## 1. Introduction

As standards of living have increased, the incidence and mortality of cancer have also increased gradually. Currently, the three major treatments for cancer are surgery, radiotherapy, and chemotherapy. Since the discovery of the first efficacious anticancer metallodrug cisplatin in 1965 by Rosenberg [1], several platinum chemotherapy compounds *viz*. carboplatin, oxaliplatin, nedaplatin, lobaplatin, and heptaplatin have been studied and approved as anticancer drugs [2]. Nevertheless, the side-effects of platinum chemotherapeutic metallopharmaceuticals [3,4,5,6,7] have led development toward non-platinum chemotherapeutics [8,9,10,11,12,13,14].

During the last few years, it has been noticeable that organotin compounds occupy an important place in cancer chemotherapy reports [15,16,17] because of their cytotoxic effects, ability to bind with DNA, anti-proliferating nature, and apoptotic-inducing nature. Thus, organotins have emerged as impending biologically active metallopharmaceuticals [18,19]. During the research and development of anticancer drugs, the growth inhibition experiment of drugs in vitro cancer cells is the first step in verifying the drug anticancer effects. It is significant in clarifying drug anticancer spectrums and screening efficient anticancer drugs. In 2021, S.K. Hadjikakou et al. reported that the organotin derivatives of cholic acid could induce apoptosis in breast cancer cells (MCF-7) [20]. In 2020, Goran N. Kaluđerović and co-workers studied the cytotoxic potential of the newly synthesized organotin against four malignant cell lines: PC-3, HT-29, MCF-7, and HepG2 [21]. In 2019, Isabel Rozas’ group studied the structure–activity relationships of new organotin (IV) anticancer agents and their cytotoxicity profile on HL-60, MCF-7, and HeLa human cancer cell lines [22]. The different structures of organotin compounds have a better inhibitory effect on cancer cells. Therefore, it is necessary to design a synthesis of more organotin compounds and screen their anticancer activity.

Due to its good biological properties, and electron-donating atoms such as carbonyl oxygen and imino nitrogen, aroylhydrazone ligands have received extensive attention in the design of organotin anticancer drugs [23,24,25,26]. α-Ketobutyric acid is a critical intermediate required of threonine metabolism, which is deaminated by threonine deaminase. Although α-ketobutyric exhibits favorable biocompatibility, it is a thermally unstable compound. The amination of α-carbonyl to form the ONO multidentate ligand can not only improve its thermal stability, but also enhance its coordination ability. Therefore, α-ketobutyric aroylhydrazone was introduced as a ligand into diorganotin to increase the biocompatibility of diorganotin complexes and decrease the large lipid–water partition coefficient of diorganotin. 

**Scheme 1:**
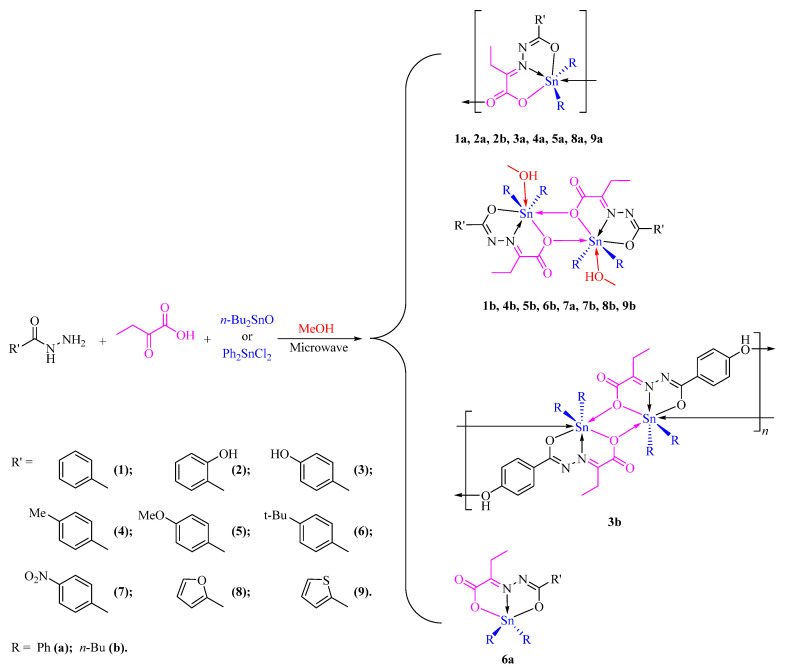
The reactions of **1a**–**9b**.

Thus, we designed a series of substituted aroylhydrazones based on 2-butyric acid as ligands, which have a peptide bond structure and multiple sites, that play a strong regulatory role on the coordination of metal ions. In this paper, we synthesized eighteen substituted aroylhydrazones diorganotin complexes by one-pot microwave-assisted (see Scheme 1), the advantages of which are short reaction times, operational simplicity, and good reproducibility. The complex structures were characterized by ^1^H, ^13^C, and ^119^Sn NMR spectroscopy; FTIR spectroscopy; HRMS; and X-ray crystallography. The in vitro anticancer activity of the complex was investigated by CCK8 assay. Most importantly, the anticancer mechanism of the complex **4b** toward MCF-7 cells was explored by the cell apoptosis assay, cell cycle analysis, TEM observations, western blot analysis, comet assay, and DNA binding test.

## 2. Results

### 2.1. Synthesis

The syntheses procedures are shown in Scheme 1. Complexes **1a**–**9b** were synthesized by the microwave-assisted method. Compared with conventional reflux approaches, microwave reactions require shorter reaction time, exhibit relatively superior operational simplicity, and are highly reproducible [27,28,29]. There are two main reasons for microwave radiation to promote chemical reactions. First, the strong transmission effect of microwave considerably increased the heating rate and improved uniformity. Second, the growing pressure and rising temperature for a sealed microwave container could facilitate the reaction. The products were obtained in a 67–86% yield. These were found to be stable toward air and moisture at room temperature. All compounds are transparent crystals that are soluble in common organic solvents such as methanol, chloroform, acetone, and dimethyl sulfoxide, but insoluble in water and in saturated aliphatic hydrocarbons.

### 2.2. Spectroscopic Data Discussion

The synthesized complexes were characterized using IR, ^1^H NMR, ^13^C NMR, ^119^Sn NMR, and HRMS. The spectral data matched the predicted structure of the complex. In the ^1^H NMR, all protons were in their predictable regions. The integral area ratios per group conformed to the predicted number of protons per group. In the ^13^C NMR, the peaks of each group were consistent with the theoretical prediction of the number of carbon atoms in the structure. The carboxyl carbon, hydrazide carbon, and imino carbon of the complexes can be clearly identified and were located at the low field position. In the ^119^Sn NMR, a sharp single peak was observed in the spectrum for each complex, which indicates that the complexes were very pure. For some complexes containing the Sn_2_O_2_ binuclear structure (**1b**, **3b**, **4b**, **5b**, **6b**, **7a**, **7b**, **8b**, and **9b**), only one single peak was observed in the ^119^Sn NMR spectrum, indicating that the chemical environment of the two tin atoms in this structure was the same.

### 2.3. Crystal Structure

**Figure 1:**
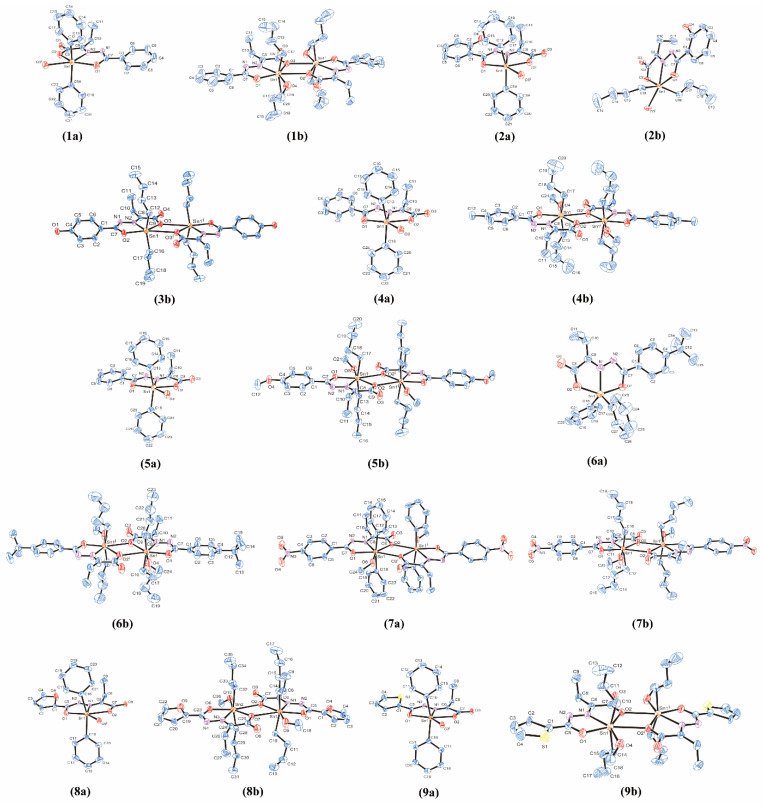
Molecular structure of **1a**–**9b**.

**Figure 2:**
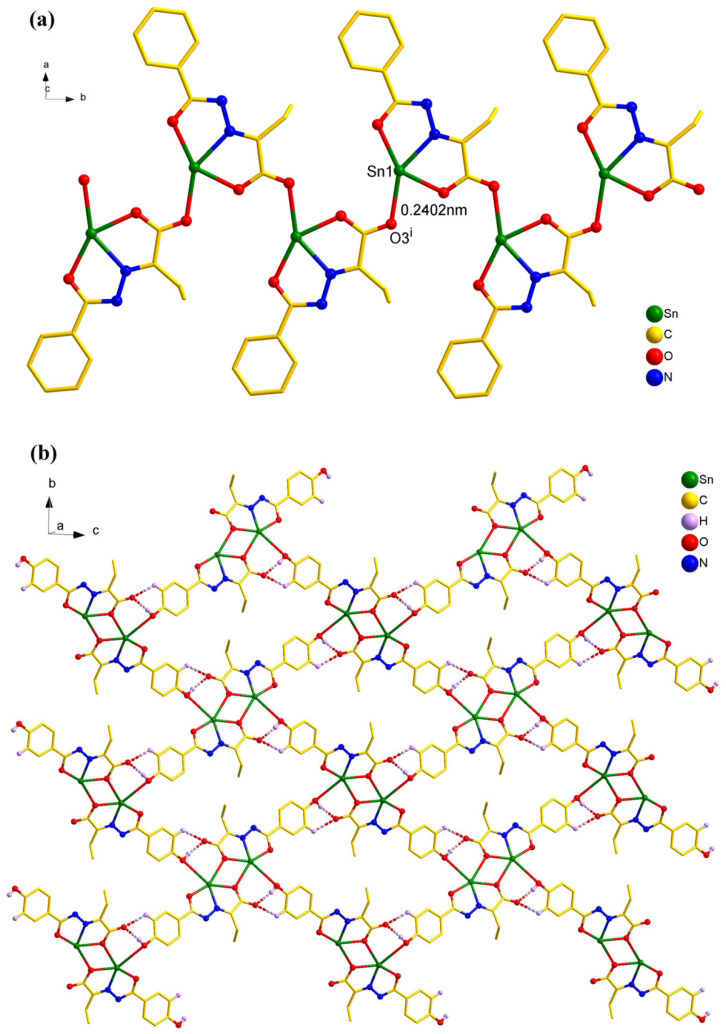
1D or 2D structures. (**a**) One-dimensional chain structure of complex **1a**, (two phenyl groups on the tin atom have been omitted for clarity). (**b**) Two-dimensional network structure of complex **3b**, (two butyl groups on the tin atom have been omitted for clarity).

The molecular structures of **1a**–**9b** are shown in Figure 1. The selected bond lengths and angles for the complexes are listed in Table S5 (Supplementary Materials). The structures of complexes **1a**–**9b** can be divided into four different coordinate types. First, in complexes **1a**, **2a**, **2b**, **3a**, **4a**, **5a**, **8a**, and **9a**, one-dimensional infinite chain structure was formed by a Sn–O bond between two adjacent asymmetric units (one-dimensional chain structure of complex **1a** is shown in Figure 2a, the other in the Supplementary Materials), it was found that the length of the Sn–O bond in each complex was different, but which were much less than the sum of the Van der Waals radii for Sn and O (0.36 nm), which proves that there is a strong interaction between Sn1 and O3^i^ [30]. The central tin atoms were all six coordinations of the distorted octahedral configuration. Second, in complexes **1b**, **4b**, **5b**, **6b**, **7a**, **7b, 8b**, and **9b**, they are centrosymmetric dimer distannoxanes, that is, they are a dinuclear molecule containing a central Sn_2_O_2_ four-membered ring, each carboxyl oxygen atom bridges the two tin atoms in an antisymmetric unit. The Sn1–O2–Sn1^i^–O2^i^ system exhibits a 0.0° torsional angle in every complex, indicating that the four-membered Sn_2_O_2_ ring is a planar structure. The central tin atoms are a seven-coordinate structure exhibiting distorted pentagonal pyramidal geometry. These structures are similar to those reported in the literature [31,32,33]. Third, for complex **3b**, a two-dimensional network structure is formed by a Sn–O bond between two adjacent asymmetric units (in Figure 2b). Unlike **1b**, participating in the Sn–O bond is the hydroxy oxygen atom on the phenyl. Moreover, this complex molecule contains rich hydrogen bonding, *d*_(O1–H1…O4)_ = 0.1830 nm, angle O1–H1…O4 = 168.22°; *d*_(C3–H3…O4)_ = 0.2712 nm, angle O1–H1…O4 = 126.76°. Finally, **6a** is a monomer structure. The structure showed a distorted trigonal bipyramidal configuration with five-coordinations to a central tin atom.

### 2.4. Anticancer Studies

The anticancer activities in complexes **1a**–**9b** and cisplatin were assessed in vitro through the CCK8 assay on cells of human breast adenocarcinoma (MCF-7), human hepatocellular carcinoma (HepG2), and human lung carcinoma (NCI-H460). IC_50_ values are listed in Table 1. For NCI-H460, IC_50_ values in complexes **1a**–**9b** ranged from 3.50 to 9.93 μM, which resembled cisplatin activity (IC_50_ = 5.63 ± 0.43 μM), whereas all complexes exhibited stronger activities than cisplatin against MCF-7 and HepG2. For the same ligand, dibutyltin complexes (**1b**, **2b**, **3b**, **4b**, **5b**, **6b**, **7b**, **8b**, and **9b**) were generally stronger than diphenyltin complexes (**1a**, **2a**, **3a**, **4a**, **5a**, **6a**, **7a**, **8a**, and **9a**). In diorganotin complexes, the discrepancy of cytotoxicity was possibly caused by minor steric hindrance or minor molecular weight. For diphenyl tin complexes, different substituents on the ligand aromatic ring have a certain effect on the activity of the complex. The ranges of IC_50_ values for MCF-7, HepG2, and NCI-H460 were 2.61–5.65, 5.34–8.99, 5.48–9.93 μM, respectively. However, the ranges of IC_50_ values against MCF-7, HepG2, and NCI-H460 were 0.47–1.50, 2.94–3.78, and 3.50–5.40 μM for dibutyltin complexes, respectively. Its variation range was small. This explains that the ligand is only a synergistic effect under this condition.

Therefore, the structure–activity analysis of the anticancer activity of the complex shows that: (i) for most complexes, the drug sensitivity of MCF-7 cells is greater than that of HepG2 and NCI-H460 cells, the dependence of anticancer activity (IC_50_ value) both on the type of alkyl as well as the type of aromatic ring on the ligand; (ii) when the alkyl is stronger, the substituent in the ligand aromatic ring has less effect on the activity, and the inhibitory activity of the butyl group is greater than that of the phenyl group, which proves that the alkyl group attached to the tin atom is the main pharmacophore; (iii) analyzing the same type of alkyl substitution of the central tin atom, with the butyl substituent, the order of influence of the aromatic ring in the ligand is as follows: *p*-Me-Ph > *p*-MeO-Ph > *p*-NO_2_-Ph > 2-furyl > *p*-OH-Ph > *o*-OH-Ph > 2-thienyl > Ph > *p*-*t*-Bu-Ph, when the phenyl group is the substituent, the order is as follows: *p*-NO_2_-Ph > *p*-Me-Ph > *p*-OH-Ph > Ph > *o*-OH-Ph > *p*-MeO-Ph > *p*-*t*-Bu-Ph > 2-thienyl > 2-furyl; (iv) the anticancer activity of aroylhydrazone diorganotin complexes may be related to the synergistic effect of alkyl group and ligand; (v) comparing the toxicity of **4b** to normal cells HL-7702, **4b** shows lower cytotoxic activity in the normal human liver cell line (IC_50_ = 7.31 ± 0.61 μM) than in the MCF-7 cell line (IC_50_ = 0.47 ± 0.04 μM), which indicated the obvious specificity for this type of tumour cell and high selectivity index factors of more than 3.0. In order to further clarify the anticancer mechanism of aroylhydrazone diorganotin complexes, we selected complex **4b**, which had the best inhibitory activity on MCF-7, for subsequent experiments.

### 2.5. Cell Apoptosis Analysis

**Figure 3:**
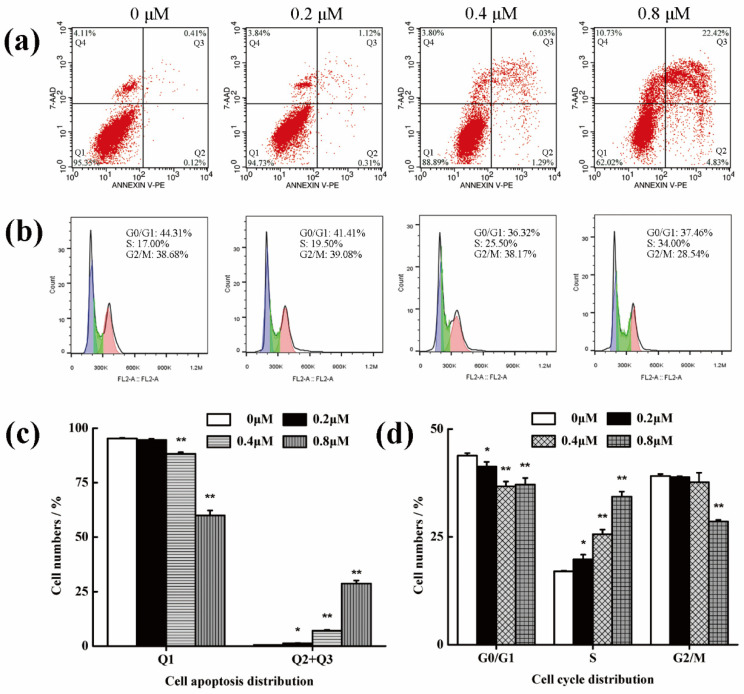
Effects of complex **4b** on cell cycle and apoptosis of MCF-7 cells. (**a**) Apoptotic effect of **4b** on MCF-7 cell line after treatment for 24 h. Q1, Q2, Q3, and Q4 respectively represent live cells, the earlier apoptotic cells, the late apoptotic cells, and cells damaged during the procedure. (**b**) Flow cytometric analysis indicated that **4b** changed MCF-7 cell cycle distribution at 24 h. (**c**) The **4b** increased apoptotic cell numbers. (**d**) **4b** arrested cell cycle S phase of MCF-7 cells. Error bar show the SD, “*” *p* < 0.05, “**” *p* < 0.01, compared with the control cells at 24 h.

Because of the superior inhibitory activity of **4b** for MCF-7 cells, a flow cytometry test was conducted to detect the apoptosis level when MCF-7 cells were exposed to **4b**. Treatment of MCF-7 cells with **4b** for 24 h led to the concentration-dependent apoptosis in Figure 3a,c. The apoptosis percentage is expressed as regions Q2 (early apoptotic cells) and Q3 (late apoptotic cells) and total percentages of apoptosis. The **4b** exhibited a negligible apoptosis induction effect on MCF-7 cells at low concentrations (0.2 μM). The **4b** concentration increased with induction of apoptosis. Total apoptosis percentages were 7.32% and 27.25 % when the concentrations were 0.4 and 0.8 μM, respectively, which were higher than those of the control. As indicated by the data, **4b** can mediate MCF-7 cancer cell apoptosis, which is consistent with the strong anticancer activity in **4b**.

### 2.6. Cell Cycle Distribution Analysis

Cell cycle analysis was performed to investigate the anti-proliferative effects of **4b**. Difference between phases of the cell cycle is based on the content of genetic material. The S phase of the cell cycle is the DNA replication phase, during which a large amount of genetic material is synthesized, so this phase contains more DNA than quiescent cells. As shown from the cell cycle detection (Figure 3b,d), after treatment with **4b**, there was significant upregulation in the S phase and significant downregulation in the G2/M phase, blocking the progression of cancer cells from the S phase to the G2/M phase, thus blocking the mitosis of the cells. **4b** induces apoptosis by inhibiting DNA replication and transcription, and achieves an anti-tumor effect. These results indicated that **4b** can induce cell cycle arrest. Significantly, the cell cycle arrest at S phase of **4b** is similar to the reported metal complexes [34,35,36] and is different from cisplatin.

### 2.7. TEM Observation of Cell Morphology

**Figure 4:**
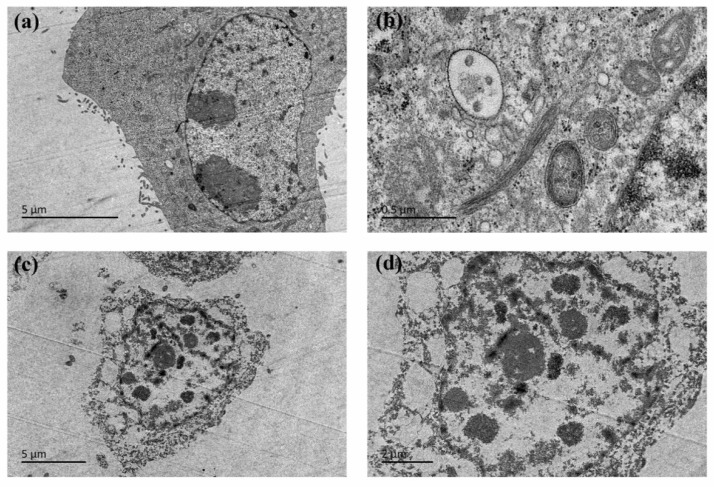
Cell morphology of the MCF-7 cell line with **4b** after treatment for 24 h. (**a**,**b**) Control group, (**c**,**d**) treated with 0.4 μM of the **4b** for 24 h.

In order to further clarify the cell death mode induced by **4b**, we used transmission electron microscopy to observe the effect of **4b** on cell ultrastructure. As shown in Figure 4a,b, the cell membrane, nucleus, and other organelles of MCF-7 cells in the control group did not show abnormal changes. After **4b** treatment, the ultrastructure of MCF-7 cancer cells were obviously destroyed. According to Figure 4c,d, we observed that the cytoplasm was concentrated and the cell volume shrank, so the microvillus on the cell membrane surface disappeared, and the structure of the other organelles were blurred. The nuclear envelope still existed, but its shape was irregular. The chromatin in the nucleus condensed and fragmented into several pieces, which were scattered in the nucleus. It is very obvious that the above appearance belonged to the specific characteristics of apoptosis.

### 2.8. Western Blotting

**Figure 5:**
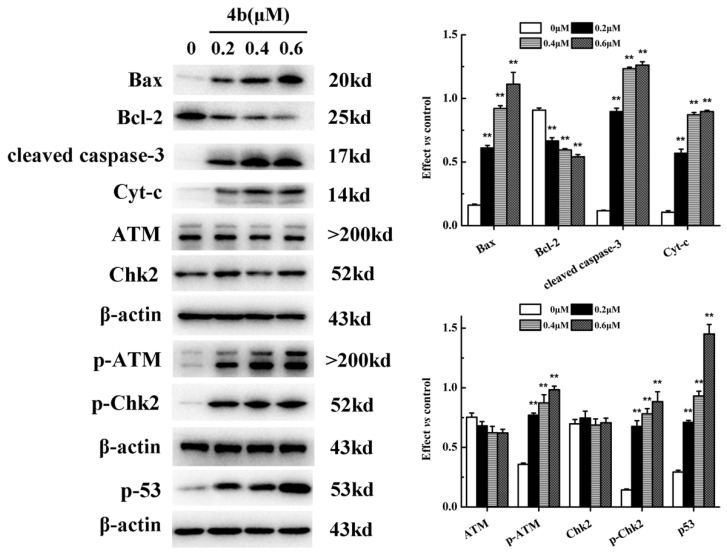
Western blot analysis of complex **4b** in MCF-7 cells. Cells were cultured in the presence of different concentrations of **4b** for 24 h. Whole-cell lysate was analyzed for Bcl-2, Bax, cleaved caspase-3, Cytochrome c, ATM, p-ATM, Chk2, p-Chk2, and p53 by immunoblotting. A representative anti-β-actin immune blot was shown as the loading control. Error bars show the SD, “**” *p* < 0.01, compared with the control.

After the MCF-7 cell line was treated with different concentrations of **4b**, compared with the control group, ATM and Chk2 protein had no obvious change trend, and the difference was not statistically significant. With the increase in **4b** concentration, Bax, cleaved caspase-3, Cytochrome c, p53, p-ATM, and p-Chk2 had a significant upward-regulation trend, Bcl-2 expression was significantly reduced, and the difference was statistically significant (Figure 5).

ATM is the direct receptor of DNA double-strand breaks (DSBs). After damage occurs, ATM automatically undergoes phosphorylation and is activated. Among them, the autophosphorylation of p-ATM (Ser1981) is a recognized marker of ATM activation. ATM is very sensitive to DNA damage, and only a small amount of DSBs can cause rapid phosphorylation of most molecules at the Ser1981 site. The results of western blotting showed that the protein level of total ATM did not change, while the phosphorylated ATM was significantly upregulated with the increase in drug concentration, indicating that phosphorylated ATM was its activated form. Among the many downstream effectors, Chk2 plays a very important role. Chk2 is a serine/threonine kinase that interferes with cell damage repair mechanisms. When there is no DNA damage, Chk2 is mostly uniformly dispersed in the cell nucleus in the state of inactive monomers. Once the external environmental conditions cause DSBs, they will gather at the damaged DNA site. The activated ATM interacts with Chk2 to phosphorylate a series of sites. The DNA damage repair pathway is to complete the repair of damaged DNA through a series of phosphorylated target proteins that bind to the DNA damage site together. If the DNA damage cannot be effectively repaired, the damaged cell will initiate the apoptosis mechanism. One pathway, the DNA damage activates ATM kinase, and ATM directly activates the p53ser15 site to mediate MCF-7 cell apoptosis. Another pathway is that when DNA damage occurs, it is detected by ATM and other related kinases, and then the damage signal is transmitted to downstream Chk2 and p53. The increase in p53 expression leads to the downregulation of the apoptosis inhibitor Bcl-2 expression, activates the expression of pro-apoptotic factor Bax, and opens the mitochondrial membrane channel [37]. The Cytochrome c is released into the cytoplasm, and finally activates the apoptosis executive protein caspase-3 to trigger cell apoptosis. 

### 2.9. Comet Assay

**Figure 6:**
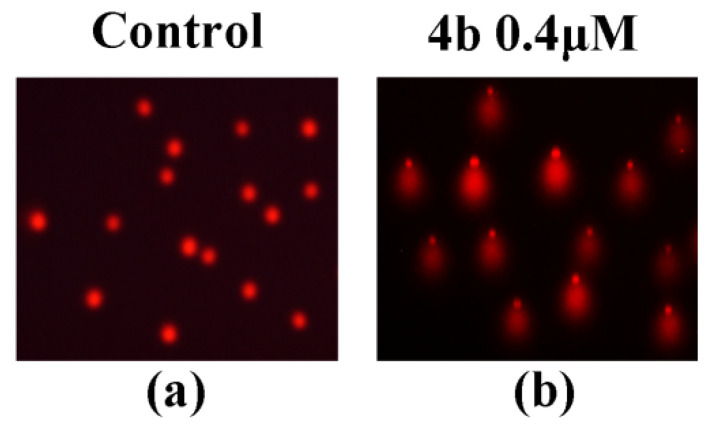
Comet assay of EB-stained MCF-7 cells in the control (**a**) and treated by 0.4 μM of the complex **4b** (**b**) after 24 h incubation.

Slight DNA damage usually leads to cell cycle arrest, while severe DNA damage can lead to cell apoptosis and necrosis. Comet assay is a versatile and sensitive method for measuring DNA damage based on DNA single and double-strand breaks. The comet assay was performed using MCF-7 cells to research the effect of complex **4b** on the DNA of the cells. As shown in Figure 6, in the control (a), no DNA damage was seen. However, MCF-7 cells incubated with the 0.4 μM **4b** for 24 h showed a well-shaped comet (b), and the length of the comet’s tail represents the extent of DNA damage. The results confirm that **4b** can induce DNA damage, which is further evidence of apoptosis.

### 2.10. DNA-Binding Studies

#### 2.10.1. UV–Visible Absorption Spectrometry

**Figure 7:**
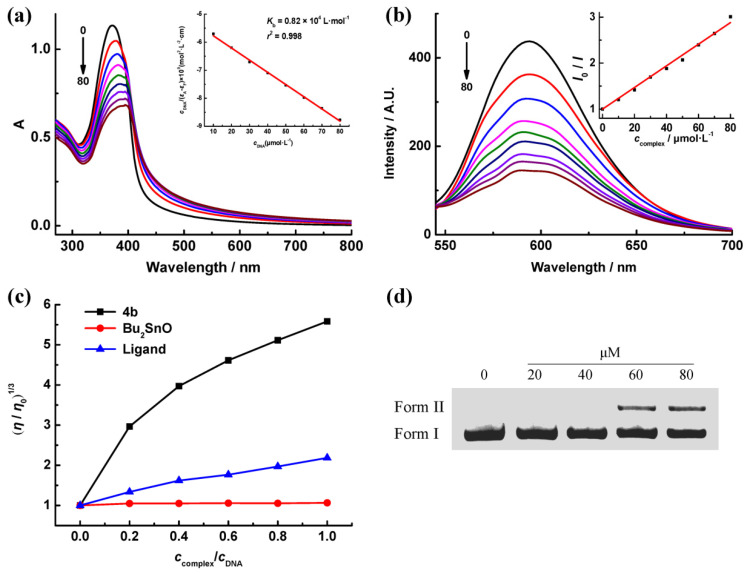
The binding of the complex **4b** and DNA, (**a**) UV–Vis absorption spectra of **4b** (50 μM) in the absence and presence of CT-DNA (0–80 μM), inset: plot of *c*_DNA_/(*ε*_A_ − *ε*_F_) vs. *c*_DNA_, (**b**) effects of **4b** (0–80 μM) on the fluorescent spectra of EB-DNA (30 μM) system, *λ*_ex_ = 258 nm, inset: plot of *I*_0_/*I* vs. *c*_complex_, (**c**) effect of increasing amounts of the **4b**, Bu_2_SnO, and ligand, (**d**) agarose gel electrophoresis of pBR322 treated with different concentrations of **4b** (0–80 μM).

Figure 7a illustrates the absorption spectra of complex **4b** in the presence of increasing amounts of CT-DNA. A strong absorption peak around 380 nm was observed. After interaction with the increasing concentration of CT-DNA, it could be seen that hypochromism was observed along with a red shift of 18 nm. The reason for hypochromism is that the interaction between the complex and the DNA causes the change in the molecule conformation. The change in the spectrum is related to the binding force, where the stronger the effect, the more obvious the hypochromism. From the absorption spectroscopy tests, *K_b_* was calculated as 0.82 × 10^4^ L∙mol^−1^ (*r*^2^ = 0.998), and the observed values of *K_b_* were in good agreement with the reported values (the order of 10^4^ L∙mol^−1^) of similar intercalated metal-complexes [38,39,40]. The relatively large amount of *K_b_* and red shift suggested that there was strong interaction between these complexes and the CT-DNA double helix.

#### 2.10.2. Fluorescence Competitive Study

Figure 7b illustrates the fluorescence quenching curve of complex **4b** at varying concentrations in the EB-DNA system. Fluorescence in the system reduced when **4b** was added, which revealed that **4b** could quench the fluorescence in the EB-DNA system. The complex could compete with EB for binding to the site on CT-DNA. The more **4b** is added, the more EB originally bound to CT-DNA will be replaced by the **4b**, which will reduce the fluorescence intensity of the EB-DNA system. Relevant illustrations are displayed to confirm that the EB-DNA fluorescence quenching by **4b** can be attributed to **4b** rivalry against EB. By using the Stern–Volmer correction formula: *I*_0_/*I* = 1 + *K_SV_*c_complex_, the *K_SV_* was measured as 2.3 × 10^4^ L∙mol^−1^, which was similar to the reported value [41,42,43]. This result implies that **4b** can intercalate into DNA. In case the tin atom in **4b** interacts with the base pairs of DNA, the insertion of terminal ligands into such pairs can be achieved to realize EB competition. Thus, squeezing of EB from the double helix of DNA can be achieved through **4b**.

#### 2.10.3. Viscosity Measurements

The viscosity experiments clearly showed that the relative viscosity of CT-DNA steadily increased with a concentration of **4b**, however, this trend was not obvious for Bu_2_SnO and the ligand. As can be seen from Figure 7c, the order of increase in viscosity was **4b** > Ligand > Bu_2_SnO. This can be explained that the **4b** and DNA employed a classical intercalation model, which demanded that the DNA helix must be extended, causing an increase in DNA viscosity [44,45]. The results further suggest an intercalating binding mode of the complex with DNA and is also consistent with the spectroscopic results and molecular docking.

#### 2.10.4. Gel Electrophoresis Studies

As can be seen from Figure 7d, complex **4b** can effectively cut the pBR322, and its cutting activity is related to the concentration of the complex. As the concentration of the complex is gradually increased, Form II was also observed. However, when the concentration reached 80 μmol∙L^−1^, there was still no Form III. Thus, complex **4b** can effectively cut pBR322 from Form I to Form II.

#### 2.10.5. Molecular Docking

**Figure 8:**
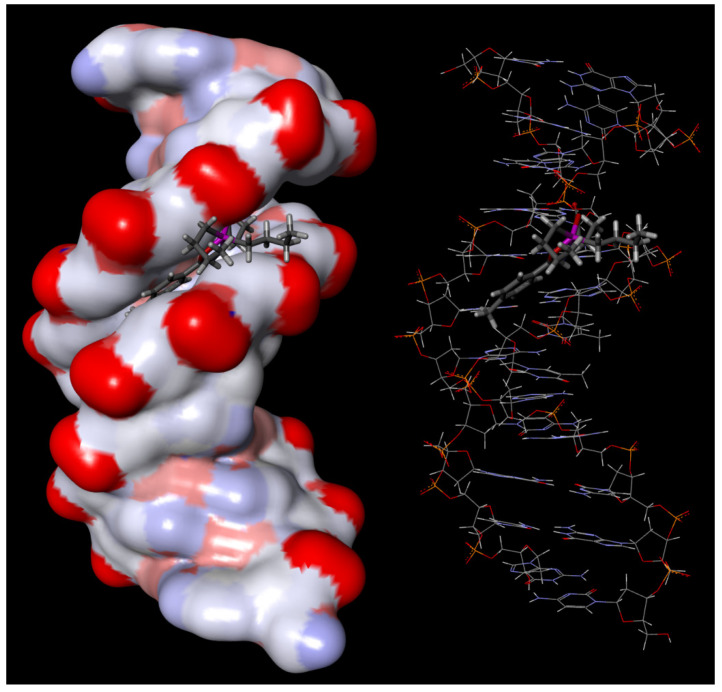
Molecular modeling of the interaction between **4b** and DNA.

Docking results of Figure 8 prove that the binding of **4b** to DNA relies on intercalation. The ligand part of **4b** was completely inserted into the double helix of DNA. The atoms are substantially planar on the ligand, and the steric hindrance is relatively small. The deoxyribonucleotide ring formed a coordinate bond with the central tin atom, with a bond length of 2.605 Å (see Figure S119). The results indicate that **4b** can bind to DNA via intercalation, which was determined through spectroscopic investigations.

## 3. Experimental

### 3.1. Materials and Methods

Calf thymus DNA (Type XV, Activated, lyophilized powder), anti-β-actin antibody, anti-β-actin antibody was obtained from Proteintech Group, Inc (Chicago, IL, USA); anti-cleaved caspase-3, anti-Bcl-2, anti-Chk2, anti-p-Chk2 (phospho T68), and anti-p53 antibodies were obtained from Abcam plc (Waltham, MA, USA); Cytochrome c antibody was obtained from Cell Signaling Technology, Inc (Danvers, MA, USA); anti-ATM and anti-p-ATM (phospho S1981) antibodies were obtained from Santa Cruz Biotechnology, Inc (Dallas, TX, USA); and pBR322 DNA used in this study was synthesized by Sangon Biotech Co. Ltd. (Shanghai, China). α-Ketobutyric acid and 4-nitrobenzhydrazide were from Sigma-Aldrich LLC. (City of Saint Louis, MO, USA); and benzhydrazid, 2-hydroxybenzhydrazide, 4-hydroxybenzhydrazide, and 4-tert-hutylbenzhydrazide were from J&K Scientific Ltd (Guangzhou, China). Dibutyltin oxide, 4-methylbenzohydrazide, 4-methoxybenzohydrazide, 2-furoic hydrazide, and 2-thiophenecarboxylic acid hydrazide were from TCI (Shanghai, China) Development Co. Ltd. (Shanghai, China). Diphenyltin dichloride was from Alfa Aesar (China) Chemical Co. Ltd. (Shanghai, China). Other chemicals were from Sinopharm Chemical Reagent Co. Ltd. (Shanghai, China). All reagents were of analytical grade obtained from commercial sources and used without further purification. Ultrapure water (18.2 MΩ·cm) obtained from a Milli-Q water purification system (Millipore Co., Billerica, MA, USA) was used in all experiments. Tris-HCl (0.01 mol·L^−1^) buffer solution was prepared by a certain amount of Tris dissolved in super pure water before use, and the pH of the solution was adjusted to 7.40 with hydrochloric acid solution (0.1 mol·L^−1^). The purity of CT-DNA was determined by comparing the absorbance at 260 and 280 nm (A_260_/A_280_ = 1.8–1.9/1). The concentration of CT-DNA was calculated by measuring the absorbance at 260 nm (ε_260_ = 6600 L·mol^−1^·cm^−1^). The reserve solution was stored at 4 °C. The ethidium bromide solution was prepared by a certain amount of ethidium bromide solid dissolved in Tris–HCl (0.01 mol·L^−1^) buffer solution.

The microwave synthesis reaction was completed using the Sineo Microwave MDS-10 High-throughput Microwave Sample Preparation Workstation (Shanghai, China). Elemental analyses for C, H, and N were determined on a PE-2400(II) analyzer (Waltham, MA, USA). The IR spectrum was obtained for KBr pellets on a Shimadzu Prestige-21 spectrophotometer in the 4000–400 cm^−1^ (Kyoto, Japan). ^1^H, ^13^C, and ^119^Sn NMR analysis were performed on a Bruker AVANCE NMR spectrometer (Karlsruhe, Germany). HRMS was obtained by Thermo Scientific LTQ Orbitrap XL (Waltham, MA, USA) or Waters UPLC I-Class Xevo G2 XS-Q Tof (Milford, MA, USA) with ESI. Crystal structure was determined on a Bruker SMART APEX II X-ray diffractometer (Karlsruhe, Germany). Thermogravimetric analyses (TGA) were recorded on a NETZSCH TG 209 F3 instrument at a heating rate of 20 °C/min from room temperature to 900 °C under air (Selb, Germany). Melting point measurement was executed on an X-4 binocular micromelting point apparatus with the temperature unadjusted (Beijing, China). UV–Vis absorption spectra was measured by a Shimadzu UV-2550 spectrometer (Kyoto, Japan). Fluorescence spectra were obtained with a Hitachi F-7000 spectrophotometer (Tokyo, Japan) with a quartz cuvette (path length = 1 cm). Viscosity experiments were conducted on an Ubbelodhe viscometer (Shanghai, China). Gel electrophoresis was measured by DYY-6C electrophoresis power supply (Beijing, China). Cell apoptosis was measured by the BD FACSCalibur CellSorting System (Franklin Lakes, NJ, USA).

### 3.2. Synthesis

The mixture comprising substituted hydrazide (1 mmol), α-ketobutyric acid (1 mmol), and relevant diorganotin (1 mmol) in 30 mL of methanol was positioned inside a microwave reactor vessel, followed by 30 min microwave irradiation at 100 °C. When the reaction kettle had cooled to room temperature, the solution in the kettle was filtered. The complex crystals were obtained by controlling solvent evaporation.

#### 3.2.1. Diphenyltin-2-(2-benzoylhydrazono) Butyrate (**1a**)

Colorless crystals, Yield 78%, m.p.: 258–260 °C. Anal. Calcd. (C_23_H_20_N_2_O_3_Sn): C, 56.25; H, 4.10; N, 5.70%. Found: C, 56.11; H, 4.08; N, 5.81%. FTIR (KBr, cm^−1^): 3048, 2978, 2936, 2878, 1636, 1599, 1585, 1503, 1481, 1460, 1433, 1393, 1342, 1298, 1265, 1202, 1175, 1096, 1065, 1043, 1026, 997, 972, 924, 845, 808, 733, 716, 694, 604, 588, 482, 453, 420. UV–Vis (DMSO + H_2_O, λ/nm): 326. ^1^H NMR (500 MHz, CDCl_3_, δ/ppm): 8.33–8.35 (m, 2H, Ar–H), 7.81–7.83 (m, 4H, Ar–H), 7.60 (tt, *J*_1_ = 7.4 Hz, *J*_2_ = 1.6 Hz, 1H, Ar–H), 7.53 (dt, *J*_1_ = 7.8 Hz, *J*_2_ = 1.3 Hz, 2H, Ar–H), 7.44–7.51 (m, 6H, Ar–H), 3.12 (q, *J* = 7.6 Hz, 2H, –CH_2_CH_3_), 1.31 (t, *J* = 7.6 Hz, 3H, –CH_2_CH_3_). ^13^C NMR (126 MHz, CDCl_3_, δ/ppm): 174.80 (–COO), 163.30 (–C(O)=N), 160.27 (–C=N), 136.15, 135.69, 132.85, 132.28, 131.44, 129.42, 128.76, 128.50 (Ar–C), 21.23 (–CH_2_CH_3_), 10.72 (–CH_2_CH_3_). ^119^Sn NMR (187 MHz, CDCl_3_, δ/ppm): −294.40. HRMS (ESI) m/z calcd for C_23_H_21_N_2_O_3_Sn^+^ [M + H]^+^ 493.0569, found 493.0569.

#### 3.2.2. Dibutyltin-2-(2-benzoylhydrazono) Butyrate (**1b**)

Colorless crystals, Yield 76%, m.p.: 88–90 °C. Anal. Calcd. (C_20_H_31_N_2_O_4_Sn): C, 49.81; H, 6.48; N, 5.81%. Found: C, 49.77; H, 6.58; N, 5.77%. FTIR (KBr, cm^−1^): 3234, 3054, 2959, 2923, 2857, 1632, 1610, 1587, 1495, 1461, 1438, 1388, 1333, 1301, 1261, 1195, 1173, 1156, 1086, 1057, 1025, 966, 934, 883, 874, 841, 799, 713, 685, 602, 579, 537, 489, 463, 412. UV–Vis (DMSO + H_2_O, λ/nm): 326. ^1^H NMR (500 MHz, CDCl_3_, δ/ppm): 8.22 (d, *J =* 7.2 Hz, 4H, Ar–H), 7.55 (t, *J =* 7.4 Hz, 2H, Ar–H), 7.47 (t, *J =* 7.6 Hz, 4H, Ar–H), 3.49(s, 6H, CH_3_OH), 3.13 (q, *J =* 7.6 Hz, 4H, –CH_2_CH_3_), 1.54–1.67 (m, 16H, –CH_2_CH_2_CH_2_CH_3_), 1.36–1.28 (m, 14H, –CH_2_CH_2_CH_2_CH_3_, –CH_2_CH_3_), 0.86 (t, *J =* 7.3 Hz, 12H, –CH_2_CH_2_CH_2_CH_3_). ^13^C NMR (126 MHz, CDCl_3_, δ/ppm): 174.88 (–COO), 164.49 (–C(O)=N), 159.76 (–C=N), 132.67, 132.39, 128.63, 128.34 (Ar–C), 50.78 (CH_3_OH), 26.73 (–CH_2_CH_2_CH_2_CH_3_), 26.37 (–CH_2_CH_2_CH_2_CH_3_), 21.99 (–CH_2_CH_3_), 21.05 (–CH_2_CH_2_CH_2_CH_3_), 13.46 (–CH_2_CH_2_CH_2_CH_3_), 10.63 (–CH_2_CH_3_). ^119^Sn NMR (187 MHz, CDCl_3_, δ/ppm): −176.94. HRMS (ESI) m/z calcd for C_20_H_32_N_2_O_4_Sn^+^ [M–CH_3_OH + H]^+^ 453.1195, found 453.1150.

#### 3.2.3. Diphenyltin-2-(2-(2-hydroxylbenzoyl)hydrazono)butyrate (**2a**)

Pale yellow crystals, Yield 71%, m.p.: 230–231 °C. Anal. Calcd. (C_23_H_20_N_2_O_4_Sn): C, 54.47; H, 3.98; N, 5.52%. Found: C, 54.51; H, 4.02; N, 5.53%. FTIR (KBr, cm^−1^): 3312, 3040, 2985, 2952, 2916, 1703, 1662, 1606, 1584, 1540, 1502, 1437, 1260, 1232, 1188, 1146, 1105, 1058, 993, 907, 857, 764, 750, 688, 654, 636, 612, 517, 492, 451, 438, 408. UV–Vis (DMSO + H_2_O, λ/nm): 332. ^1^H NMR (500 MHz, *d*_6_-DMSO, δ/ppm): 12.93 (s, 1H, Ar–OH), 7.96 (dd, *J*_1_ *=* 7.8, *J*_2_ *=* 1.4 Hz, 1H, Ar–H), 7.49–7.77 (m, 4H, Ar-H), 7.37–7.42 (m, 1H, Ar–H), 7.21–7.35 (m, 6H, Ar–H), 6.96–6.86 (m, 2H, Ar–H), 2.65 (q, *J =* 7.5 Hz, 2H, –CH_2_CH_3_), 0.99 (t, *J =* 7.5 Hz, 3H, –CH_2_CH_3_). ^13^C NMR (126 MHz, *d*_6_–DMSO, δ/ppm): 172.89 (–COO), 164.39 (–C(O)=N), 160.02 (–C=N), 154.68, 150.45, 133.81, 133.44, 129.18, 128.09, 127.90, 118.59, 116.95, 116.74 (Ar–C), 19.85 (–CH_2_CH_3_), 9.34 (–CH_2_CH_3_). ^119^Sn NMR (187 MHz, *d*_6_–DMSO, δ/ppm): −595.39. HRMS (ESI) m/z calcd for C_23_H_20_N_2_O_4_Sn^+^ [M + H]^+^ 509.0518, found 509.0509.

#### 3.2.4. Dibutyltin-2-(2-(2-hydroxylbenzoyl)hydrazono)butyrate (**2b**)

Pale yellow crystals, Yield 76%, m.p.: 197–199 °C. Anal. Calcd. (C_19_H_28_N_2_O_4_Sn): C, 48.85; H, 6.04; N, 6.00%. Found: C, 48.77; H, 6.01; N, 5.97%. FTIR (KBr, cm^−1^): 3057, 3021, 2957, 2924, 2872, 2857, 2731, 1616, 1597, 1578, 1560, 1522, 1489, 1456, 1414, 1387, 1331, 1256, 1229, 1206, 1169, 1117, 1090, 1070, 1034, 968, 853, 831, 795, 772, 754, 714, 702, 683, 671, 604, 590, 534, 519, 446, 424. UV–Vis (DMSO + H_2_O, λ/nm): 330. ^1^H NMR (500 MHz, CDCl_3_, δ/ppm): 11.94 (s, 1H, Ar–O**H**), 8.08 (dd, *J*_1_ = 7.9 Hz, *J*_2_ = 1.3 Hz, 1H, Ar–H), 7.46 (dt, *J*_1_ = 8.5 Hz, *J*_2_ = 1.5 Hz, 1H, Ar–H), 7.02 (d, *J* = 8.3 Hz, 1H, Ar–H), 6.96 (t, *J* = 7.5 Hz, 1H, Ar–H), 3.03 (q, *J* = 7.6 Hz, 2H, –C**H**_2_CH_3_), 1.63–1.66 (m, 4H, –CH_2_CH_2_CH_2_CH_3_), 1.46–1.48(m, 4H, –CH_2_CH_2_CH_2_CH_3_), 1.27–1.31 (m, 7H, –CH_2_CH_2_CH_2_CH_3_, –CH_2_CH_3_), 0.84(d, *J* = 7.3 Hz, 6H, –CH_2_CH_2_CH_2_CH_3_). ^13^C NMR (126 MHz, CDCl_3_, δ/ppm): 175.75 (–COO), 165.72 (–C(O)=N), 160.71 (–C=N), 157.59, 134.85, 130.29, 119.2, 117.52, 115.32 (Ar–C), 26.81 (–CH_2_CH_2_CH_2_CH_3_), 26.30 (–CH_2_CH_2_CH_2_CH_3_), 25.04 (–CH_2_CH_2_CH_2_CH_3_), 21.19 (–CH_2_CH_3_), 13.47 (–CH_2_CH_2_CH_2_CH_3_), 9.88 (–CH_2_CH_3_). ^119^Sn NMR (187 MHz, CDCl_3_, δ/ppm): –233.15. HRMS (ESI) m/z calcd for C_19_H_29_N_2_O_4_Sn^+^ [M + H]^+^ 469.1144, found 469.1139.

#### 3.2.5. Diphenyltin-2-(2-(4-hydroxylbenzoyl)hydrazono)butyrate (**3a**)

Pale yellow crystals, Yield 67%, m.p.: 233–235 °C. Anal. Calcd. (C_23_H_20_N_2_O_4_Sn): C, 54.47; H, 3.98; N, 5.52%. Found: C, 54.45; H, 4.04; N, 5.61%. FTIR (KBr, cm^−1^): 3693, 3069, 3053, 2978, 2935, 2877, 1615, 1598, 1586, 1514, 1488, 1480, 1454, 1431, 1415, 1388, 1329, 1252, 1231, 1207, 1172, 1159, 1090, 1073, 1041, 997, 967, 919, 853, 831, 804, 780, 763, 732, 693, 671, 602, 552, 537, 518, 454, 414. UV–Vis (DMSO + H_2_O, λ/nm): 330. ^1^H NMR (500 MHz, *d*_4_–MeOH, δ/ppm): 13.65 (s, 1H, Ar–OH), 7.85 (d, *J =* 7.0 Hz, 4H, Ar–H), 7.76–7.78 (m, 2H, Ar–H), 7.41–7.46 (m, 4H, Ar–H), 6.88–6.95 (m, 4H, Ar–H), 2.75 (q, *J* = 7.5 Hz, 2H, –CH_2_CH_3_), 1.03 (t, *J* = 7.5 Hz, 3H, –CH_2_CH_3_). ^13^C NMR (126 MHz, *d*_4_–MeOH, δ/ppm): 176.75 (–COO), 166.85 (–C(O)=N), 163.31 (–C=N), 145.93, 135.99, 132.40, 131.02, 129.78, 124.02, 116.63, 116.55 (Ar–C), 28.31 (–CH_2_CH_3_), 10.38 (–CH_2_CH_3_). ^119^Sn NMR (187 MHz, *d*_4_–MeOH, δ/ppm): −236.30. HRMS (ESI) m/z calcd for C_23_H_20_N_2_O_4_Sn^+^ [M + H]^+^ 509.0518, found 509.0509.

#### 3.2.6. Dibutyltin-2-(2-(4-hydroxylbenzoyl)hydrazono)butyrate (**3b**)

Light yellow crystals, Yield 76%, m.p.: 231–233 °C. Anal. Calcd. (C_19_H_28_N_2_O_4_Sn): C, 48.85; H, 6.04; N, 6.00%. Found: C, 48.81; H, 6.11; N, 6.05%. FTIR (KBr, cm^−1^): 3121, 3071, 2965, 2930, 2870, 2805, 1653, 1636, 1589, 1489, 1456, 1387, 1331, 1317, 1292, 1250, 1200, 1167, 1092, 1065, 968, 935, 849, 802, 768, 702, 637, 606, 584, 540, 513, 478, 444, 419. UV–Vis (DMSO + H_2_O, λ/nm): 340. ^1^H NMR (500 MHz, (CD_3_)_2_CO, δ/ppm): 9.18 (s, 2H, Ar–OH), 8.09–8.12 (m, 4H, Ar–H), 6.92–6.95 (m, 4H, Ar–H), 3.01 (q, *J* = 7.6 Hz, 4H, –CH_2_CH_3_), 1.60–1.66 (m, 8H, –CH_2_CH_2_CH_2_CH_3_), 1.52–1.59 (m, 8H, –CH_2_CH_2_CH_2_CH_3_), 1.27–1.35 (m, 8H, –CH_2_CH_2_CH_2_CH_3_), 1.21 (t, *J* = 7.6 Hz, 6H, –CH_2_CH_3_), 0.83 (t, *J* = 7.3 Hz, 12H, –CH_2_CH_2_CH_2_CH_3_). ^13^C NMR (126 MHz, CDCl_3_, δ/ppm): 174.4 (–COO), 164.01 (–C(O)=N), 159.44 (–C=N), 130.85, 125.17, 115.24, 99.97 (Ar–C), 50.93 (CH_3_OH), 29.70 (–CH_2_CH_3_), 26.68 (–CH_2_CH_2_CH_2_CH_3_), 26.42 (–CH_2_CH_2_CH_2_CH_3_), 20.67 (–CH_2_CH_2_CH_2_CH_3_), 13.46 (–CH_2_CH_2_CH_2_CH_3_), 10.61 (–CH_2_CH_3_). ^119^Sn NMR (187 MHz, (CD_3_)_2_CO, δ/ppm): −521.81. HRMS (ESI) m/z calcd for C_19_H_29_N_2_O_4_Sn^+^ [M + H]^+^ 469.1144, found 469.1138.

#### 3.2.7. Diphenyltin-2-(2-(4-methylbenzoyl)hydrazono)butyrate (**4a**)

Light yellow crystals, Yield 71%, m.p.: 208–210 °C. Anal. Calcd. (C_24_H_22_N_2_O_3_Sn): C, 57.06; H, 4.39; N, 5.54%. Found: C, 57.01; H, 4.41; N, 5.55%. FTIR (KBr, cm^−1^): 3059, 2980, 2938, 2880, 1634, 1597, 1582, 1491, 1479, 1460, 1433, 1396, 1341, 1327, 1296, 1267, 1206, 1175, 1157, 1094, 1063, 1043, 1022, 972, 845, 814, 750, 733, 716, 694, 606, 586, 544, 488, 451. UV–Vis (DMSO + H_2_O, λ/nm): 326. ^1^H NMR (500 MHz, CDCl_3_, δ/ppm): 8.23 (d, *J* = 8.2 Hz, 2H, Ar–H), 7.81–7.83 (m, 4H, Ar–H), 7.44–7.49 (m, 6H, Ar–H), 7.32 (d, *J* = 8.0 Hz, 2H, Ar–H), 3.10 (q, *J* = 7.6 Hz, 2H, –CH_2_CH_3_), 2.46 (s, 3H, CH_3_–Ph), 1.30 (t, *J* = 7.6 Hz, 3H, –CH_2_CH_3_).^13^C NMR (126 MHz, CDCl_3_, δ/ppm): 174.84 (–COO), 163.45 (–C(O)=N), 159.51 (–C=N), 143.66, 136.14, 135.73, 131.38, 129.44, 129.37, 129.24, 128.78 (Ar–C), 21.81 (CH_3_–Ph), 21.15 (–CH_2_CH_3_), 10.71 (–CH_2_CH_3_). ^119^Sn NMR (187 MHz, CDCl_3_, δ/ppm): –294.44. HRMS (ESI) m/z calcd for C_24_H_23_N_2_O_3_Sn^+^ [M + H]^+^ 507.0725, found 507.0718.

#### 3.2.8. Dibutyltin-2-(2-(4-methylbenzoyl)hydrazono)butyrate (**4b**)

Colorless crystals, Yield 77%, m.p.: 158–160 °C. Anal. Calcd. (C_21_H_34_N_2_O_4_Sn): C, 50.73; H, 6.89; N, 5.63%. Found: C, 50.77; H, 6.84; N, 5.67%. FTIR (KBr, cm^−1^): 3231, 3065, 2961, 2922, 2870, 1611, 1582, 1489, 1458, 1393, 1335, 1300, 1261, 1194, 1177, 1155, 1088, 1059, 1020, 966, 934, 837, 802, 750, 685, 629, 579, 534, 484, 465, 444, 420. UV–Vis (DMSO + H_2_O, λ/nm): 342. ^1^H NMR (500 MHz, CDCl_3_, δ/ppm): 8.10 (d, *J* = 8.2 Hz, 4H, Ar–H), 7.27 (d, *J* = 2.9 Hz, 4H, Ar–H), 3.49(s, 6H, CH_3_OH), 3.12 (q, *J* = 7.6 Hz, 4H, –CH_2_CH_3_), 2.43 (s, 6H, CH_3_–Ph), 1.60–1.65 (m, 14H, –CH_2_CH_2_CH_2_CH_3_, –CH_2_CH_3_), 1.29–1.35 (m, 16H, –CH_2_CH_2_CH_2_CH_3_), 0.87 (t, *J* = 7.3 Hz, 12H, –CH_2_CH_2_CH_2_CH_3_).^13^C NMR (126 MHz, CDCl_3_, δ/ppm): 174.91 (–COO), 164.14 (–C(O)=N), 159.54 (–C=N), 143.14, 129.81, 129.10, 128.62 (Ar–C), 50.84 (CH_3_OH), 26.70 (–CH_2_CH_2_CH_2_CH_3_), 26.39 (–CH_2_CH_2_CH_2_CH_3_), 21.74 (–CH_2_CH_2_CH_2_CH_3_), 21.13 (–CH_2_CH_3_), 13.46 (–CH_2_CH_2_CH_2_CH_3_), 10.63 (–CH_2_CH_3_). ^119^Sn NMR (187 MHz, CDCl_3_, δ/ppm): −158.60. HRMS (ESI) m/z calcd for C_20_H_31_N_2_O_3_Sn^+^ [M + H]^+^ 467.1351, found 467.1343.

#### 3.2.9. Diphenyltin-2-(2-(4-methoxybenzoyl)hydrazono)butyrate (**5a**)

Pale yellow, Yield 79%, m.p.: 234–236 °C. Anal. Calcd. (C_24_H_22_N_2_O_4_Sn): C, 55.31; H, 4.25; N, 5.38%. Found: C, 55.40; H, 4.24; N, 5.37%. FTIR (KBr, cm^−1^): 3449, 3071, 3048, 2965, 2930, 2872, 1634, 1601, 1582, 1495, 1477, 1458, 1431, 1393, 1337, 1323, 1302, 1254, 1204, 1177, 1096, 1067, 1024, 847, 731, 694, 640, 604, 588, 544, 513, 446, 422. UV–Vis (DMSO + H_2_O, λ/nm): 332. ^1^H NMR (500 MHz, CDCl_3_, δ/ppm): 8.31 (d, *J* = 8.7Hz, 2H, Ar–H), 7.80–7.82 (m, 4H, Ar–H), 7.43–7.46(m, 6H, Ar–H), 7.01 (d, *J* =8.6 Hz, 2H, Ar–H), 3.90 (s, 3H, CH_3_O–Ph), 3.08 (q, *J* = 7.6 Hz, 2H, –CH_2_CH_3_), 1.30 (t, *J* = 7.6 Hz, 3H, –CH_2_CH_3_). ^13^C NMR (126 MHz, CDCl_3_, δ/ppm): 174.48 (–COO), 163.53 (–C(O)=N), 158.75 (–C=N), 136.15, 135.84, 131.36, 130.78, 129.36, 124.59, 113.87 (Ar–C), 55.51 (CH_3_O–Ph), 21.09 (–CH_2_CH_3_), 10.66 (–CH_2_CH_3_). ^119^Sn NMR (187 MHz, CDCl_3_, δ/ppm): −294.15. HRMS (ESI) m/z calcd for C_24_H_23_N_2_O_4_Sn ^+^ [M + H]^+^ 523.0674, found 523.0669.

#### 3.2.10. Dibutyltin-2-(2-(4-methoxybenzoyl)hydrazono)butyrate (**5b**)

Yellow, Yield 81%, m.p.: 155–157 °C. Anal. Calcd. (C_21_H_34_N_2_O_5_Sn): C, 49.15; H, 6.68; N, 5.49%. Found: C, 49.20; H, 6.62; N, 5.44%. FTIR (KBr, cm^−1^): 3485, 3076, 2959, 2928, 2872, 2857, 2837, 1603, 1585, 1512, 1489, 1460, 1412, 1389, 1339, 1304, 1254, 1198, 1171, 1088, 1061, 1032, 966, 847, 808, 764, 702, 685, 638, 625, 604, 583, 534, 515, 451, 415. UV–Vis (DMSO + H_2_O, λ/nm): 328. ^1^H NMR (500 MHz, CDCl_3_, δ/ppm): 8.17 (d, *J* = 8.4 Hz, 4H, Ar–H), 6.96 (d, *J* = 8.5 Hz, 4H, Ar–H), 3.88 (s, 6H, CH_3_O–Ph), 3.48 (s, 6H, CH_3_OH), 3.11 (q, *J* = 7.6 Hz, 4H, –CH_2_CH_3_), 1.60–1.64 (m, 14H, –CH_2_CH_2_CH_2_CH_3_, –CH_2_CH_3_), 1.24–1.37 (m,16H, –CH_2_CH_2_CH_2_CH_3_), 0.88 (t, *J* = 7.3 Hz, 12H, –CH_2_CH_2_CH_2_CH_3_).^13^C NMR (126 MHz, CDCl_3_, δ/ppm): 174.56 (–COO), 164.20 (–C(O)=N), 163.18 (–C=N), 158.86, 130.59, 125.00, 113.71 (Ar–C), 55.47 (CH_3_O–Ph), 50.83 (CH_3_OH), 26.71 (–CH_2_CH_2_CH_2_CH_3_), 26.39 (–CH_2_CH_2_CH_2_CH_3_), 21.07 (–CH_2_CH_2_CH_2_CH_3_), 21.03 (–CH_2_CH_3_), 13.45 (–CH_2_CH_2_CH_2_CH_3_), 10.59 (–CH_2_CH_3_). ^119^Sn NMR (187 MHz, CDCl_3_, δ/ppm): −157.87. HRMS (ESI) m/z calcd for C_20_H_31_N_2_O_4_Sn^+^ [M-CH_3_OH + H]^+^ 483.1300, found 483.1297.

#### 3.2.11. Diphenyltin-2-(2-(4-tert-butylbenzoyl)hydrazono)butyrate (**6a**)

Yellow, Yield 86%, m.p.: 267–268 °C. Anal. Calcd. (C_27_H_28_N_2_O_3_Sn): C, 59.26; H, 5.16; N, 5.12%. Found: C, 59.21; H, 5.11; N, 5.07%. FTIR (KBr, cm^−1^): 3055, 2961, 2903, 2868, 1686, 1659, 1628, 1607, 1576, 1476, 1462, 1435, 1396, 1325, 1292, 1246, 1192, 1159, 1084, 1061, 1020, 964, 853, 833, 808, 770, 739, 729, 714, 696, 594, 548, 496, 444, 420. UV–Vis (DMSO + H_2_O, λ/nm): 328. ^1^H NMR (500 MHz, CDCl_3_, δ/ppm): 8.28 (d, *J* = 8.5 Hz, 2H, Ar-H), 7.81–7.83 (m, 4H, Ar–H), 7.54 (d, *J* = 8.5 Hz, 2H, Ar–H), 7.43–7.49 (m, 6H, Ar–H), 3.11 (q, *J* = 7.6 Hz, 2H, –CH_2_CH_3_), 1.38 (s, 9H, –C(CH_3_)_3_), 1.30 (t, *J* = 7.6 Hz, 3H, –CH_2_CH_3_).^13^C NMR (126 MHz, CDCl_3_, δ/ppm): 174.78 (–COO), 163.47 (–C(O)=N), 159.52 (–C=N), 156.69, 136.15, 135.71, 131.38, 129.39, 129.36, 128.62, 125.50 (Ar–C), 35.17 (–C(CH_3_)_3_), 31.15 (–C(CH_3_)_3_), 21.16 (–CH_2_CH_3_), 10.69 (–CH_2_CH_3_). ^119^Sn NMR (187 MHz, CDCl_3_ δ/ppm): −249.57. HRMS (ESI) m/z calcd for C_27_H_29_N_2_O_3_Sn^+^ [M + H]^+^ 549.1195, found 549.1190.

#### 3.2.12. Dibutyltin-2-(2-(4-tert-butylbenzoyl)hydrazono)butyrate (**6b**)

Colorless, Yield 77%, m.p.: 154–156 °C. Anal. Calcd. (C_24_H_40_N_2_O_4_Sn): C, 53.45; H, 7.48; N, 5.20%. Found: C, 53.35; H, 7.39; N, 5.14%. FTIR (KBr, cm^−1^): 3431, 3067, 2959, 2926, 2870, 2859, 1605, 1582, 1572, 1489, 1458, 1391, 1364, 1341, 1304, 1292, 1267, 1190, 1159, 1086, 1061, 1016, 968, 935, 851, 837, 806, 772, 706, 685, 594, 550, 492, 465, 419. UV–Vis (DMSO + H_2_O, λ/nm): 330. ^1^H NMR (500 MHz, CDCl_3_, δ/ppm): 8.13 (dt, *J*_1_ = 8.6 Hz, *J*_2_ = 1.8 Hz, 4H, Ar–H), 7.49 (dt, *J*_1_ = 8.5 Hz, *J*_2_ = 1.8 Hz, 4H, Ar–H), 3.49 (s, 6H, CH_3_OH), 3.12 (q, *J* =7.6 Hz, 4H, –CH_2_CH_3_), 1.58–1.65 (m, 14H, –CH_2_CH_2_CH_2_CH_3_, –CH_2_CH_3_), 1.36 (s, 18H, –C(C**H**_3_)_3_), 1.28–1.34 (m, 16H, –CH_2_CH_2_CH_2_CH_3_), 0.88 (t, *J* = 7.3 Hz, 12H, –CH_2_CH_2_CH_2_CH_3_).^13^C NMR (126 MHz, CDCl_3_, δ/ppm): 174.87 (–COO), 164.11 (–C(O)=N), 159.60 (–C=N), 156.19, 129.80, 128.46, 125.36 (Ar–C), 50.84 (CH_3_OH), 35.10 (–C(CH_3_)_3_), 31.16 (–C(CH_3_)_3_), 26.68 (–CH_2_CH_2_CH_2_CH_3_), 26.39 (–CH_2_CH_2_CH_2_CH_3_), 21.07 (–CH_2_CH_3_), 21.01 (–CH_2_CH_2_CH_2_CH_3_), 13.45 (–CH_2_CH_2_CH_2_CH_3_), 10.59 (–CH_2_CH_3_). ^119^Sn NMR (187 MHz, CDCl_3_, δ/ppm): −157.43. HRMS (ESI) m/z calcd for C_23_H_37_N_2_O_3_Sn^+^ [M–CH_3_OH + H]^+^ 509.1821, found 509.1816.

#### 3.2.13. Diphenyltin-2-(2-(4-nitrobenzoyl)hydrazono)butyrate (**7a**)

Yellow, Yield 83%, m.p.: 213–214 °C. Anal. Calcd. (C_48_H_46_N_6_O_12_Sn_2_): C, 50.73; H, 4.08; N, 7.40%. Found: C, 50.77; H, 4.10; N, 7.37%. FTIR (KBr, cm^−1^): 3464, 3057, 2972, 2938, 1690, 1597, 1564, 1528, 1508, 1485, 1458, 1431, 1387, 1339, 1317, 1292, 1263, 1192, 1169, 1155, 1105, 1086, 1055, 1018, 999, 966, 868, 854, 804, 779, 718, 698, 689, 588, 550, 521, 453, 417. UV–Vis (DMSO + H_2_O, λ/nm): 348. ^1^H NMR (500 MHz, CDCl_3_, δ/ppm): 8.49 (d, *J* = 8.8 Hz, 4H, Ar–H), 8.35 (d, *J* = 8.8 Hz, 4H, Ar–H), 7.79–7.81 (m, 8H, Ar–H), 7.45–7.52 (m, 12H, Ar–H), 3.14 (q, *J* = 7.6Hz, 4H, –CH_2_CH_3_), 1.33 (t, *J* = 7.6 Hz, 6H, –CH_2_CH_3_). ^13^C NMR (126 MHz, CDCl_3_, δ/ppm): 172.73 (–COO), 162.82 (–C(O)=N), 162.67 (–C=N), 150.33, 138.03, 136.04, 135.34, 131.66, 129.67, 129.57, 123.61 (Ar–C), 21.48 (–CH_2_CH_3_), 10.83 (–CH_2_CH_3_). ^119^Sn NMR (187 MHz, CDCl_3_, δ/ppm): −294.33. HRMS (ESI) m/z calcd for C_23_H_20_N_3_O_5_Sn^+^ [M-CH_3_OH + H]^+^ 538.0419, found 538.0415.

#### 3.2.14. Dibutyltin-2-(2-(4-nitrobenzoyl)hydrazono)butyrate (**7b**)

Yellow, Yield 80%, m.p.: 151–153 °C. Anal. Calcd. (C_40_H_62_N_6_O_12_Sn_2_): C, 45.48; H, 5.92; N, 7.96%. Found: C, 45.52; H, 5.99; N, 8.03%. FTIR (KBr, cm^−1^): 3422, 3113, 3055, 2957, 2926, 2857, 1636, 1612, 1597, 1533, 1503, 1458, 1387, 1335, 1321, 1292, 1261, 1194, 1169, 1155, 1105, 1084, 1057, 1015, 966, 870, 854, 806, 719, 702, 602, 581, 548, 521, 480, 463, 444. UV–Vis (DMSO + H_2_O, λ/nm): 346. ^1^H NMR (500 MHz, CDCl_3_, δ/ppm): 8.39 (dt, *J*_1_ = 8.9 Hz, *J*_2_ = 1.9 Hz, 4H, Ar–H), 8.31 (dt, *J*_1_ = 8.9 Hz, *J*_2_ = 1.9 Hz, 4H, Ar–H), 3.50 (s, 6H, CH_3_OH), 3.15 (q, *J* = 7.6 Hz, 4H, –CH_2_CH_3_), 1.64–1.71 (m, 8H, –CH_2_CH_2_CH_2_CH_3_), 1.52–1.57 (m, 8H, –CH_2_CH_2_CH_2_CH_3_), 1.31–1.35 (m, 14H, –CH_2_CH_2_CH_2_CH_3_, –CH_2_CH_3_), 0.87 (t, *J* = 7.3 Hz, 12H, –CH_2_CH_2_CH_2_CH_3_). ^13^C NMR (126 MHz, CDCl_3_, δ/ppm): 172.83 (–COO), 164.41 (–**C**(O)=N), 161.81 (–**C**=N), 150.04, 138.54, 129.62, 123.45 (Ar–**C**), 50.85 (CH_3_OH), 26.77 (–CH_2_CH_2_CH_2_CH_3_), 26.36 (–CH_2_CH_2_CH_2_CH_3_), 22.94 (–CH_2_CH_2_CH_2_CH_3_), 21.25 (–**C**H_2_CH_3_), 13.45 (–CH_2_CH_2_CH_2_CH_3_), 10.75 (–CH_2_**C**H_3_). ^119^Sn NMR (187 MHz, CDCl_3_, δ/ppm): −183.68. HRMS (ESI) m/z calcd for C_19_H_28_N_3_O_5_Sn^+^ [M-CH_3_OH + H]^+^ 498.1045, found 498.1040.

#### 3.2.15. Diphenyltin-2-(2-furoylhydrazono)butyrate (**8a**)

Yellow, Yield 72%, m.p.: 270–272 °C. Anal. Calcd. (C_21_H_18_N_2_O_4_Sn): C, 52.43; H, 3.77; N, 5.82%. Found: C, 52.51; H, 3.89; N, 5.88%. FTIR (KBr, cm^−1^): 3464, 3144, 3053, 2980, 2936, 2874, 1628, 1595, 1580, 1504, 1481, 1470, 1431, 1408, 1368, 1327, 1263, 1225, 1198, 1144, 1105, 1061, 1009, 972, 899, 883, 835, 802, 764, 735, 694, 669, 600, 581, 544, 507, 453, 424. UV–Vis (DMSO + H_2_O, λ/nm): 332. ^1^H NMR (500 MHz, CDCl_3_, δ/ppm): 7.79–7.81 (m, 4H, Ar–H), 7.69–7.70 (m, 1H, Ar–H), 7.45–7.50 (m, 6H, Ar–H), 7.42 (dd, *J*_1_ = 3.5 Hz, *J*_2_ = 0.7 Hz, 1H, Ar–H), 6.62 (q, *J* = 1.7 Hz, 1H, Ar–H), 3.09 (q, *J* = 7.6 Hz, 2H, –CH_2_CH_3_), 1.29 (t, *J* = 7.6 Hz, 3H, –CH_2_CH_3_). ^13^C NMR (126 MHz, CDCl_3_, δ/ppm): 167.24 (–COO), 163.10 (–C(O)=N), 160.19 (–C=N), 147.15, 146.58, 136.13, 135.47, 131.50, 129.43, 117.58, 112.30 (Ar–C), 21.25 (–CH_2_CH_3_), 10.81 (–CH_2_CH_3_). ^119^Sn NMR (187 MHz, CDCl_3_ δ/ppm): −296.62. HRMS (ESI) m/z calcd for C_21_H_19_N_2_O_4_Sn^+^ [M + H]^+^ 483.0361, found 483.0355.

#### 3.2.16. Dibutyltin-2-(2-furoylhydrazono)butyrate (**8b**)

Yellow, Yield 77%, m.p.: 132–134 °C. Anal. Calcd. (C_18_H_30_N_2_O_5_Sn): C, 45.69; H, 6.39; N, 5.92%. Found: C, 45.55; H, 6.43; N, 5.93%. FTIR (KBr, cm^−1^): 3462, 3140, 3119, 2963, 2926, 2870, 2859, 1628, 1609, 1578, 1503, 1474, 1460, 1427, 1406, 1364, 1331, 1263, 1227, 1198, 1140, 1076, 1057, 1009, 970, 897, 885, 872, 837, 800, 764, 754, 702, 683, 596, 575, 536, 515, 444. UV–Vis (DMSO + H_2_O, λ/nm): 334. ^1^H NMR (500 MHz, CDCl_3_, δ/ppm): 7.65–7.65 (m, 2H, Ar–H), 7.89 (dd, *J*_1_ = 3.5 Hz, *J*_2_ = 0.7 Hz, 2H, Ar–H), 6.58–6.59 (m, 2H, Ar–H), 3.49 (d, *J* = 5.2 Hz, 6H, CH_3_OH), 3.09 (q, *J* = 7.5 Hz, 4H, –CH_2_CH_3_), 1.63–1.67 (m, 8H, –CH_2_CH_2_CH_2_CH_3_), 1.51–1.58 (m, 8H, –CH_2_CH_2_CH_2_CH_3_), 1.27–1.35 (m, 14H, –CH_2_CH_2_CH_2_CH_3_, –CH_2_CH_3_), 0.86 (t, *J* = 7.3 Hz, 12H, –CH_2_CH_2_CH_2_CH_3_).^13^C NMR (126 MHz, CDCl_3_, δ/ppm): 167.41 (–COO), 164.55 (–C(O)=N), 158.58 (–C=N), 147.45, 146.10, 116.71, 112.15 (Ar–C), 50.83 (CH_3_OH), 26.68 (–CH_2_CH_2_CH_2_CH_3_), 26.38 (–CH_2_CH_2_CH_2_CH_3_), 22.00 (–CH_2_CH_2_CH_2_CH_3_), 21.06 (–CH_2_CH_3_), 13.45 (–CH_2_CH_2_CH_2_CH_3_), 10.74 (–CH_2_CH_3_). ^119^Sn NMR (187 MHz, CDCl_3_, δ/ppm): −176.68. HRMS (ESI) m/z calcd for C_17_H_27_N_2_O_4_Sn^+^ [M–CH_3_OH + H]^+^ 443.0987, found 443.0980.

#### 3.2.17. Diphenyltin-2-(2-thienoylhydrazono)butyrate (**9a**)

Yellow, Yield 70%, m.p.: 161–162 °C. Anal. Calcd. (C_21_H_18_N_2_O_3_SSn): C, 50.73; H, 3.65; N, 5.63; S, 6.45%. Found: C, 50.80; H, 3.71; N, 5.72; S, 6.55%. FTIR (KBr, cm^−1^): 3441, 3094, 3049, 2974, 2936, 2876, 1630, 1618, 1593, 1578, 1560, 1530, 1497, 1477, 1458, 1431, 1387, 1358, 1317, 1308, 1265, 1202, 1148, 1094, 1065, 1024, 997, 966, 922, 854, 839, 802, 733, 710, 694, 604, 573, 563, 536, 453, 419. UV–Vis (DMSO + H_2_O, λ/nm): 338. ^1^H NMR (500 MHz, CDCl_3_, δ/ppm): 8.05 (dd, *J*_1_ = 2.5 Hz, *J*_2_ = 1.2 Hz, 1H, Ar–H), 7.80–7.82 (m, 4H, Ar–H), 7.63 (dd, *J*_1_ = 3.8 Hz, *J*_2_ = 1.2 Hz, 1H, Ar–H), 7.45–7.50 (m, 6H, Ar–H), 7.20–7.19 (m, 1H, Ar–H), 3.07 (q, *J* = 7.6 Hz, 2H, –CH_2_CH_3_), 1.28 (t, *J* = 7.6 Hz, 3H, –CH_2_CH_3_). ^13^C NMR (126 MHz, CDCl_3_, δ/ppm): 170.99 (–COO), 163.32 (–C(O)=N), 159.19 (–C=N), 136.38, 136.14, 135.61, 132.38, 132.21, 131.47, 129.42, 128.02 (Ar–C), 21.18 (–CH_2_CH_3_), 10.69 (–CH_2_CH_3_). ^119^Sn NMR (187 MHz, CDCl_3_ δ/ppm): –294.76. HRMS (ESI) m/z calcd for C_21_H_19_N_2_O_3_SSn^+^ [M + H]^+^ 499.0133, found 499.0128.

#### 3.2.18. Dibutyltin-2-(2-thienoylhydrazono)butyrate (**9b**)

Yellow, Yield 79%, m.p.: 90–92 °C. Anal. Calcd. (C_18_H_30_N_2_O_4_SSn): C, 44.19; H, 6.18; N, 5.73; S, 6.55%. Found: C, 44.22; H, 6.23; N, 5.77; S, 6.62%. FTIR (KBr, cm^−1^): 3431, 3102, 2957, 2924, 2870, 2857, 1578, 1530, 1489, 1458, 1431, 1387, 1360, 1346, 1325, 1267, 1225, 1198, 1144, 1080, 1059, 1032, 962, 856, 837, 800, 745, 710, 685, 662, 602, 557, 525, 492, 461. UV–Vis (DMSO + H_2_O, λ/nm): 354. ^1^H NMR (500 MHz, CDCl_3_, δ/ppm): 7.89 (dd, *J*_1_ = 3.7 Hz, *J*_2_ = 1.2 Hz, 4H, Ar–H), 7.57 (dt, *J*_1_ = 5.0 Hz, *J*_2_ = 1.2 Hz, 4H, Ar–H), 7.14–7.15 (m, 2H, Ar–H), 3.08 (q, *J* = 7.6 Hz, 4H, –CH_2_CH_3_), 1.62–1.69 (m, 8H, –CH_2_CH_2_CH_2_CH_3_), 1.54–1.60 (m, 8H, –CH_2_CH_2_CH_2_CH_3_), 1.27–1.37 (m, 14H, –CH_2_CH_2_CH_2_CH_3,_ –CH_2_CH_3_), 0.87 (t, *J* = 7.3 Hz, 12H, –CH_2_CH_2_CH_2_CH_3_).^13^C NMR (126 MHz, CDCl_3_, δ/ppm): 171.06 (–**C**OO), 164.50 (–**C**(O)=N), 158.50 (–**C**=N), 136.85, 131.73, 131.67, 127.85 (Ar–**C**), 50.84 (CH_3_OH), 26.70 (–CH_2_CH_2_CH_2_CH_3_), 26.30 (–CH_2_CH_2_CH_2_CH_3_), 22.21 (–CH_2_CH_2_CH_2_CH_3_), 21.03 (–**C**H_2_CH_3_), 13.47 (–CH_2_CH_2_CH_2_CH_3_), 10.63 (–CH_2_**C**H_3_). ^119^Sn NMR (187 MHz, CDCl_3_, δ/ppm): −509.34. HRMS (ESI) m/z calcd for C_17_H_27_N_2_O_3_SSn^+^ [M–CH_3_OH + H]^+^ 459.0759, found 459.0750.

### 3.3. Single Crystal Structure Determination

X-ray diffraction spectral collection for crystals was achieved using a Smart Apex II CCD diffractometer from Bruker through Mo–K*_α_* (*λ* = 0.71073 Å) radiation using a graphite monochromator. The *φ–ω* scan technique was used for diffraction spectral gathering at an ambient temperature. The acquired data were subjected to multiscan correction of absorption. The crystal structures were determined through direct manipulation, and full-matrix least-squares was performed to refine the crystal structures on *F*^2^. Before anisotropical refinement, successive difference Fourier syntheses yielded all the remaining atoms (nonhydrogen). The hydrogen atoms were positioned in measured locations or placed onto the Fourier maps, followed by isotropical refinement using the atom (nonhydrogen)-associated variables of isotropic vibration they were bound to. Measurements were performed using SHELXL [46] on WINGX [47]. Complex-related crystal information and structure refinement parameters are presented in Table S4 (Supplementary Materials). Crystallographic data for the structures of complexes reported in this paper have been deposited with the Cambridge Crystallographic Data Center as supplementary publication nos. CCDC 2112812-2112828 (**1a**, **1b**, **2a**, **2b**, **3b**, **4a**, **4b**, **5a**, **5b**, **6a**, **6b**, **7a**, **7b**, **8a**, **8b**, **9a,** and **9b**).

### 3.4. In Vitro Anticancer Activity

NCI-H460(ATCC No: HTB-177), HepG2(ATCC No: HB-8065), and MCF-7(ATCC No: HTB-22) cells were obtained from American Tissue Culture Collection (ATCC). HL-7702 cell was obtained from the Chinese Academy of Sciences. The cells were maintained at 37 °C in a 5% CO_2_ incubator in RPMI 1640 (GIBICO, Invitrogen) containing 10% fetal bovine serum (GIBICO, Invitrogen). The 5 mM stock solutions of complexes were prepared in DMSO and diluted in fresh medium for use. The final concentration of DMSO never exceeded 0.1% (*v*/*v*). Cell proliferation was assessed by the CCK8 assay. A total of 100 μL of cells (40 cells·μL^−1^) incubated at 37 °C, 5% CO_2_ was seeded into 96-well plates. Then, the medium was replaced with the respective medium containing complexes at different concentrations and incubated for 24 h. Ten μL CCK8 solution was added to each well and incubated at 37 °C for an additional 4 h. The optical density was detected with a microplate reader at 450 nm. Eleven concentrations (0.03 μM–32 μM) were set for the compounds and at least three parallels of every concentration were used. All experiments were repeated at least three times. The data were calculated using Graph Pad Prism version 7.0. The IC_50_ was fitted using a non-linear regression model with a sigmoidal dose response.

### 3.5. Cell Apoptosis Assay

Approximately 1 × 10^5^ cells were inoculated on each well of a six-well plate and subjected to 24 h cultivation. Next, cells were processed using candidate complexes at varying concentrations for 24 h, and were then subjected to two rounds of cold PBS rinsing, followed by assaying with an Annexin-V-FITC/PI Apoptosis Kit (MultiSciences Biotech, China) according to the manufacturer’s instructions. Cell apoptosis was tested using a flow cytometer (BD^TM^ FACS Calibur, Franklin Lakes, NJ, USA).

### 3.6. Cell Cycle Analysis

Approximately 1 × 10^5^ cells were inoculated on each well of a six-well plate and subjected to 24 h cultivation. The cell cycle progression after treatment with **4b** was performed by using the Cell Cycle and Apoptosis Analysis Kit (Beyotime Biotechnology, China), according to the manufacturer’s instructions and reference methods. MCF-7 cells were treated with indicated concentration of **4b** in 2 mL total medium for 24 h before harvest. The cells were fixed with ice-cold 70% ethanol overnight at 4 °C. After centrifugation, the cell pellets were washed with cold PBS once, and RNaseA (10 μL) and PI (10 μL) were added to the cells. Cells were then incubated for 15 min at room temperature in the dark and then detected by flow cytometry. Parallel determination was conducted three times.

### 3.7. Transmission Electron Microscope (TEM)

MCF-7 cell aggregates were fixed with 2.5% glutaraldehyde-PBS buffer for 2 h at 4 °C. After rinsing three times with PBS buffer for 30 min, the samples were post-fixed with 1% OsO_4_ for another 2 h at 4 °C. Then, the samples were dehydrated in a graded series of ethanol–acetone with gradient ascent (50–70–90–100%), and embedded in epoxy resins. The sample was sliced through with an ultramicrotome, stained with uranyl acetate, and examined under electron microscope (Talos F200C, FEI, Hillsboro, OR, USA).

### 3.8. Western Blot Analysis

Inoculate logarithmic growth phase was recorded for MCF-7 cells in a 25 cm^2^ cell culture flask (2 × 10^6^ cells per flask) and cultured for 24 h. They were then exposed to the specified concentration of complex **4b** in an incubator at 37 °C and 5% CO_2_ for 24 h. The cell lysates were prepared using RIPA lysis buffer; the cytoplasmic extracts were prepared using a Cytoplasmic Protein Extraction Kit following the manufacturer’s instructions. Equal amounts of protein were separated by 10% SDS−PAGE and transferred onto PVDF membranes. After blocking with 5% skimmed milk, the membranes were incubated for 120 min at 37 °C with the specific rabbit or mouse anti-human primary antibodies including anti-β-actin (1:10000), anti-cleaved caspase-3 (1:1000), anti-Bax (1:2000), anti-Bcl-2 (1:1000), Cytochrome c (Cyt c; 1:1000), anti-ATM (1:1000), anti-p-ATM (phospho S1981) (1:1000), anti-Chk2(1:1000), anti-p-Chk2 (phospho T68) (1:1000), and anti-p53 (1:1000) antibodies, respectively. Next, HRP-conjugated affinipure goat anti-rabbit or anti-mouse secondary antibody (1:5000) was added for 90 min at room temperature. Then, the wells were washed with 0.05% PBST solution. Immunoreactive bands were visualized using enhanced chemiluminescence reagent and autoradiography following the manufacturer’s instructions. Optical density of each band was analyzed with ImageJ software (National Institutes of Health, Bethesda, MD, USA) using β-actin as an internal control.

### 3.9. Comet Assay

MCF-7 cells (2 × 10^5^ cells/mL) were performed to plant in 6-well plates and incubated for 24 h. Cells were dealt with **4b** (0.4 uM) for 24 h. Moreover, cells were collected and washed with PBS, then cell suspension and 1% low melting point agarose were mixed to drop onto slides and placed with glass coverslips, which was allowed to settle for 10 min at 4 °C. Coverslips were removed and cells were lysed for 1 h at 4 °C. The slides were then placed in freshly prepared alkaline electrophoresis solution to unwind DNA for 20 min. Electrophoresis was performed under alkaline conditions for 20 min at 25 V and 300 mA. After electrophoresis the cells were stained with ethidium bromide (EB) solution for 20 min in darkness at 4 °C. Comet images were obtained by fluorescence microscope (Nikon ECLIPSE Ti, Japan).

### 3.10. DNA-Binding Studies

#### 3.10.1. UV–Visible Absorption Spectrometry

The potential modes of the complex binding with DNA were assessed with a UV–Vis spectroscope, which was also used for the determination of *K_b_*, the relevant intrinsic DNA-binding constants. The following Wolfe–Shimmer equation was used for *K_b_* value determination [48]:

*c*_DNA_/(*ε*_A_ − *ε*_F_) = *c*_DNA_/(*ε*_B_ − *ε*_F_) + 1/*K_b_* (*ε*_B_ − *ε*_F_) (1)

where *c*_DNA_ refers to the CT-DNA concentration; *ε*_A_ represents the extinction coefficient detected at a random concentration of DNA; *ε*_F_ denotes the free complex’s extinction coefficient; and *ε*_B_ denotes the extinction coefficient during full complex–CT-DNA binding. We recorded the UV–Vis data of DNA under a 25 °C condition over 0–80 μM concentrations at a complex concentration of 50 μM. We collected the spectra in a 270–800 nm range. Corresponding slit width was set to 5 nm. The *K_b_* values were determined using the Wolfe–Shimmer formula plus *c*_DNA_/(*ε*_A_ − *ε*_F_) vs. *c*_DNA_ plots_._

#### 3.10.2. Fluorescence Competitive Study

For the fluorescence investigation, a volumetric flask (5 mL) was used to prepare a mixture comprising 30 μM CT-DNA, 3 μM EB, and 0–80 μM complex. Fluorescence data collection was performed for 3 h under 25 °C. The results revealed emission wavelengths ranging from 540 to 700 nm and an excitation wavelength of 258 nm. Slit width setting was 5 nm for both emission and excitation. Finally, the Stern–Volmer equation was used to determine the quenching constant (*K*sv) of the complex.

#### 3.10.3. Viscosity Measurements

Viscosity tests were performed using the DNA concentration of 0–50 μM. The test was performed on the Ubbelohde viscometer. The obtained samples were stored at 25 (±0.02) °C in a water bath. For the determination of the flow time in samples, the digital stopwatch was used. Viscosity values were computed based on the flow time of DNA including solutions to correct the flow time in buffer (*t*_0_), *η* = (*t* − *t*_0_) [49]. The data are presented by (*η*/*η*_0_)^1/3^ vs. (*c*_complex_/*c*_DNA_), in which *η* indicates the DNA viscosity of the complex; *η*_0_ represents the DNA viscosity; and *c*_complex_ refers to the complex concentration. Here, *c*_DNA_ denotes the DNA concentration.

#### 3.10.4. Gel Electrophoresis Studies

For the gel electrophoresis experiments, the gel electrophoresis experiments were performed by fixed pBR322 plasmid DNA concentration and increasing the concentration of **4b** (0, 20, 40, 60, 80 μM), and the contents were incubated for 1 h at 25 °C and they were electrophoresed for 1 h at 150 V on 1.0% agarose gel in TAE buffer (pH = 8.0). Goldview was used for staining, the image was photographed by using a gel imaging documentation system under UV light mode.

#### 3.10.5. Molecular Docking

The molecular operating environment (MOE) program was used to perform docking between molecules. We obtained the crystal architecture information of the DNA molecules from the Protein Data Bank (code: 453D) and eliminated water and embedded small molecules through MOE. Hydrogen atoms were introduced into DNA according to the electrostatic requirements. Next, point charges were computed, with minimal corresponding energy. The CIF data for the complex were transformed into its PDB counterpart by using Mercury 3.8 before it was imported into MOE. Next, symmetrical units and coordinated solvent molecules were erased, along with minimization of corresponding energies, thus obtaining the complex for subsequent docking. Discovery Studio 3.5 could be used to derive the molecular docking outcomes.

## 4. Conclusions

Eighteen diorganotin (IV) complexes were synthesized through one-pot microwave irradiation. NMR, HRMS, and X-ray single-crystal diffraction were performed to characterize complex structure. Biological assay results indicated that all the complexes exhibited anticancer activity against test cancer cells (HepG2, NCI-H460, MCF-7), and **4b** exhibited superior inhibitory activity in MCF-7 cell lines to other complexes. More importantly, we studied the anti-tumor mechanism of complex **4b**, where the results demonstrated that **4b** causes cell apoptosis by damaging DNA. DNA binding studies have shown that complex **4b** can interact with DNA through insertion mode.

## Figures and Tables

**Table 1:** Inhibition action of the complexes to cancer cells in vitro.

Complex	IC_50_/μM			
	MCF-7	HepG2	NCI-H460	HL-7702
**1a**	3.85 ± 0.29	8.19 ± 0.21	9.47 ± 0.31	
**1b**	1.39 ± 0.04	3.18 ± 0.23	4.38 ± 0.29	
**2a**	3.97 ± 0.20	8.99 ± 0.35	7.84 ± 0.26	
**2b**	1.29 ± 0.05	3.02 ± 0.12	4.19 ± 0.16	
**3a**	3.77 ± 0.16	7.25 ± 0.17	7.47 ± 0.24	
**3b**	1.18 ± 0.07	3.07 ± 0.10	5.15 ± 0.28	
**4a**	3.00 ± 0.09	5.34 ± 0.11	5.48 ± 0.16	
**4b**	0.47 ± 0.04	3.00 ± 0.25	3.50 ± 0.28	7.31 ± 0.61 (15.6) ^a^
**5a**	4.36 ± 0.20	7.22 ± 0.12	6.88 ± 0.20	
**5b**	0.66 ± 0.05	3.78 ± 0.24	4.38 ± 0.13	
**6a**	4.91 ± 0.07	6.27 ± 0.16	6.08 ± 0.42	
**6b**	1.50 ± 0.21	3.06 ± 0.11	4.24 ± 0.26	
**7a**	2.61 ± 0.33	5.91 ± 0.17	8.30 ± 0.32	
**7b**	1.06 ± 0.09	3.57 ± 0.19	5.25 ± 0.31	
**8a**	5.65 ± 0.17	8.38 ± 0.15	9.93 ± 0.28	
**8b**	1.11 ± 0.07	2.94 ± 0.09	5.26 ± 0.13	
**9a**	5.38 ± 0.22	8.42 ± 0.36	7.00 ± 0.24	
**9b**	1.32 ± 0.05	3.09 ± 0.16	5.40 ± 0.38	
**Cisplatin**	16.47 ± 1.45	17.56 ± 1.84	5.63 ± 0.43	

^a^ The selectivity index factor, defined as IC_50_ (normal HL-7702 cells)/IC_50_ MCF-7 cancer cells), is given in parentheses.

## Supplementary Materials

The following are available online at https://www.mdpi.com/article/10.3390/ijms222413525/s1.
