# Supplementary material for: Aroylhydrazone Diorganotin Complexes Causes DNA Damage and Apoptotic Cell Death: From Chemical Synthesis to Biochemical Effects
Source: Int J Mol Sci. 2021 Dec 16;22(24):13525. doi: 10.3390/ijms222413525 (PMC8709053; doi:10.3390/ijms222413525)
Supplement: Supplementary file 1 [file ijms-22-13525-s001.zip › ijms-1494281-supplementary.pdf]

Aroylhydrazone diorganotin complexes causes DNA damage and apoptotic cell death: From chemical synthesis to biochemical effects

Wujiu Jiang [1,2,*], Yuxing Tan [1,2] and Yiyuan Peng [1,*]

1 Key Laboratory of Functional Small Organic Molecule, Ministry of Education, Key Laboratory of Green Chemistry, Jiangxi Province, College of Chemistry and Chemical Engineering, Jiangxi Normal University, Nanchang, Jiangxi 330022, PR China; jiang59@jxnu.edu.cn (W.J); tanyuxing@hynu.edu.cn (Y.T); yypeng@jxnu.edu.cn (Y.P)

2 Key Laboratory of Functional Metal-Organic Compounds of Hunan Province, Key Laboratory of Functional Organometallic Materials, University of Hunan Province, Hunan Provincial Engineering Research Center for Monitoring and Treatment of Heavy Metals Pollution in the Upper Reaches of XiangJiang River, College of Chemistry and Materials Science, Hengyang Normal University, Hengyang, Hunan 421008, PR China.

1 Synthesis

2-(2-(4-methylbenzoyl)hydrazono)butanoic acid (**H_2L**)

2 Crystallographic data

2.1 Crystallographic data for **1a-2b, 3b-9b**.

2.2 The selected bond length(Å) and angles(°) for **1a-2b, 3b-9b**.

2.3 The 1D structures of **2a**, **2b**, **4a**, **5a**, **8a**, **9a**.

3 Spectra

3.1 The IR spectra of **1a-9b**.

3.2 The NMR spectra of **1a-9b**.

3.3 The HRMS spectra of **1a-9b**.

3.4 The UV-vis spectra of **1a-9b**.

4 TGA

5 Cell

6 Docking

1 Synthesis

2-(2-(4-methylbenzoyl)hydrazono)butanoic acid (**H_2L**)

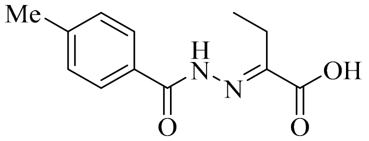

To a mixture of 2-ketobutyric acid (2.02 g, 20 mmol), sodium hydroxide (0.80 g, 20 mmol) and methanol (100 mL) were stired in the reaction flask. When the solid is completely dissolved, 4-methylbenzohydrazide (3.00 g, 20 mmol) was added to the reaction flask. The reaction mixture were heated to reflux for 5 hrs. The end of the reaction, the solvent was removed by evaporation in vacuo, the gray solid were obtained. Dissolve all solids in distilled water (100mL), and then the solution collected by vacuum filtration. The pH of the filtrate was adjusted to 2~3 with hydrochloric acid, and vacuum dry the solid after suction filtration, 2-(2-(4-methylbenzoyl) hydrazono)butanoic acid were obtained, yield 79 %. m.p.: 135~137°C. IR (KBr, cm^{-1}): 3233, 3209, 2974, 2937, 1695, 1685, 1668, 1661, 1652, 1636, 1613, 1595, 1570, 1528, 1496, 1474, 1465, 1457, 1450, 1319, 1295, 1274, 1252, 1190, 1161, 1148, 1122, 1088, 1055, 915, 829, 801, 774, 746, 667, 652, 636, 611, 579, 481. ^{1}H NMR (500 MHz, $CDCl_3$, δ/ppm): 13.85 (s, 1H), 9.21 (s, 1H), 7.77 (d, J = 7.8 Hz, 3H), 7.19 (d, J = 7.9 Hz, 3H), 2.60 (q, J = 7.4 Hz, 2H), 2.35 (s, 3H), 1.14 (t, J = 7.6 Hz, 3H). ^{13}C NMR (126 MHz, $CDCl_3$, δ/ppm): 177.79, 165.24, 143.31, 129.43, 129.38, 127.68, 127.48, 27.51, 21.52, 11.67.

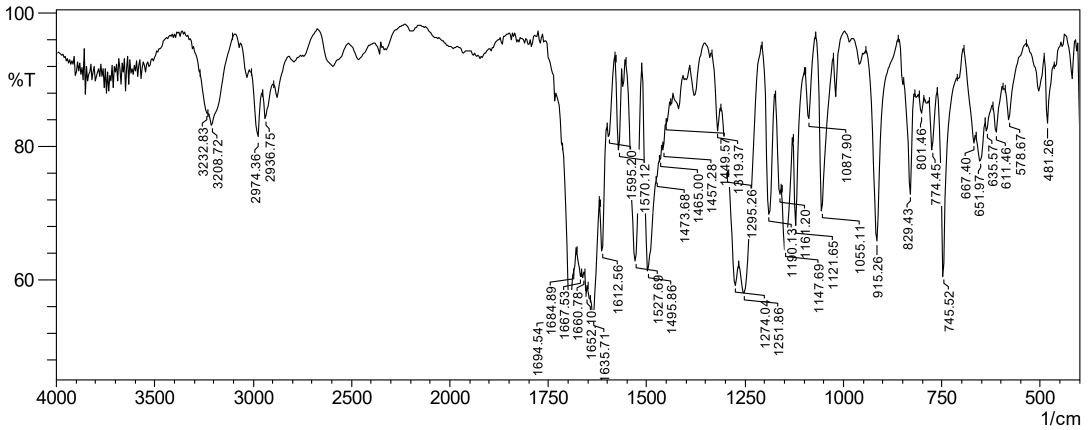

Figure S1 The IR spectra of **H_2L**

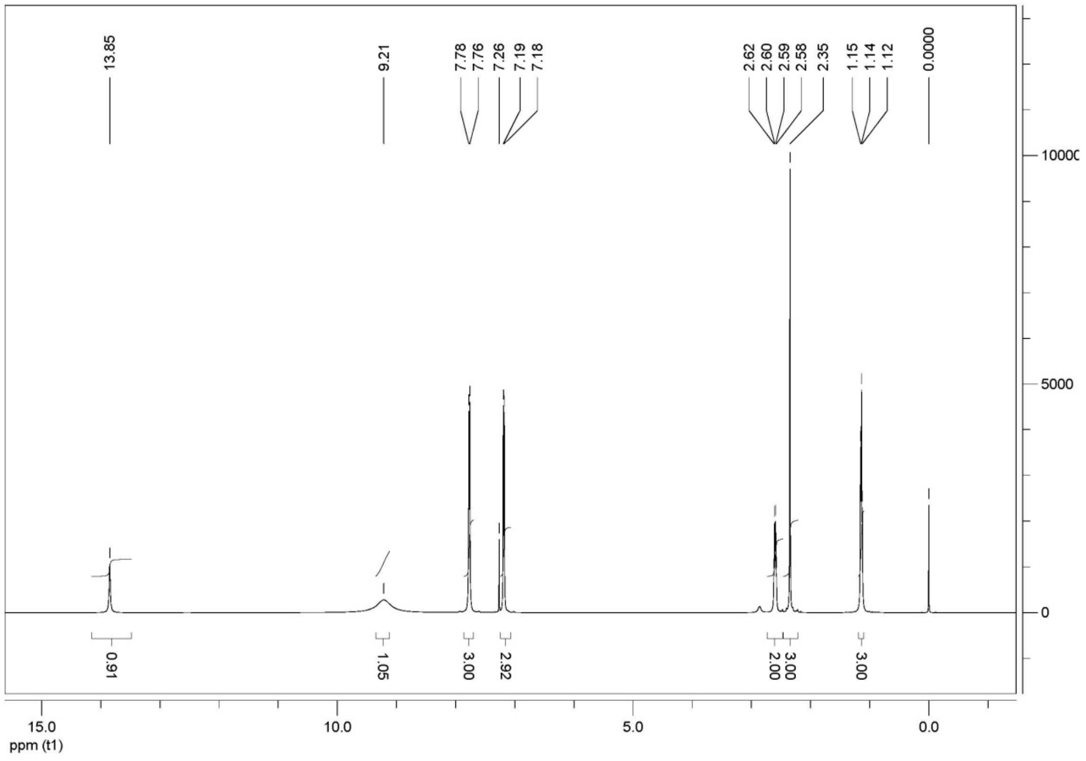

Figure S2 The ^{1}H NMR spectra of $\mathbf{H_2L}$

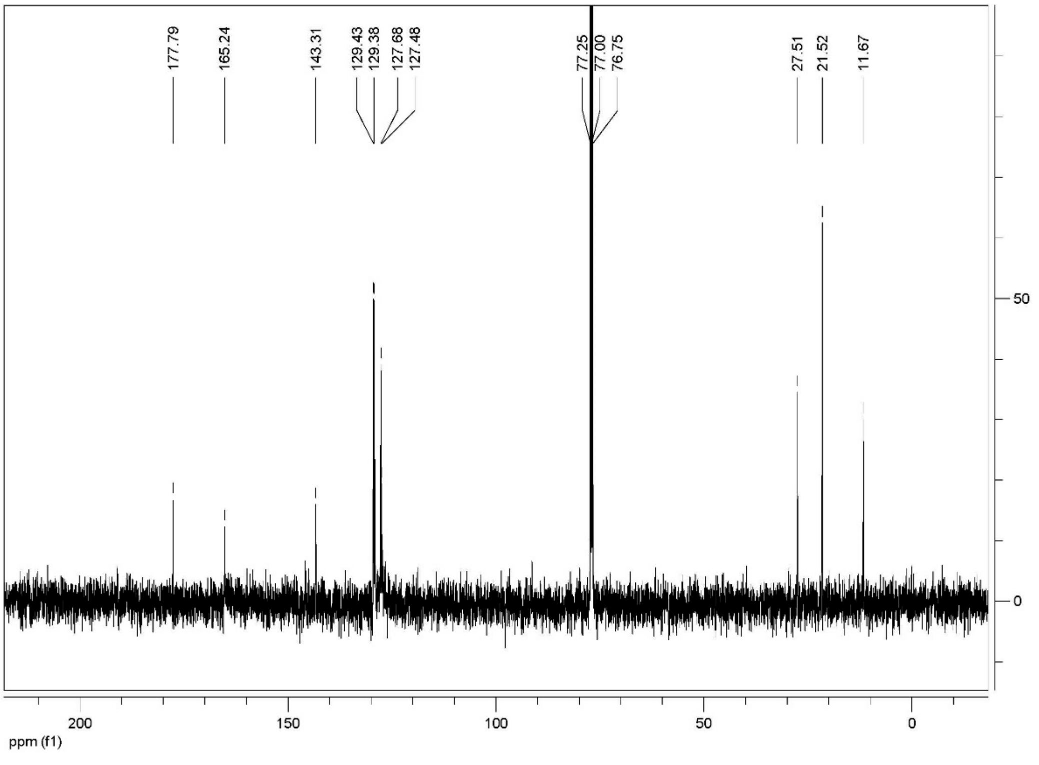

Figure S3 The ^{13}C NMR spectra of $\mathbf{H_2L}$

2 Crystallographic data

2.1 Crystallographic data for **1a-2b, 3b-9b**.

Table S1 Crystallographic data for **1a-2b, 3b**.

**Complexes**	**1a**	**1b**	**2a**	**2b**	**3b**
Empirical formula	$C_{23}H_{20}N_2O_3Sn$	$C_{20}H_{31}N_2O_4Sn$	$C_{23}H_{20}N_2O_4Sn$	$C_{19}H_{28}N_2O_4Sn$	$C_{19}H_{28}N_2O_4Sn$
Formula weight	491.10	482.16	507.10	467.12	467.12
Temperature / K	296(2)	296(2)	296(2)	296(2)	293(2)
Wavelength / Å	0.71073	0.71073	0.71073	0.71073	0.71073
Crystal system	Monoclinic	Monoclinic	Monoclinic	Monoclinic	Monoclinic
space group	$P2_1/n$	$P2_1/c$	$P2_1/n$	$C2/c$	$C2/c$
a / Å	11.8100(7)	12.8306(10)	12.0454(9)	23.7834(9)	16.8486(15)
b / Å	8.9659(5)	20.4640(17)	8.9146(7)	8.7817(3)	13.7348(9)
c / Å	20.2830(11)	8.8888(7)	20.2009(15)	23.4607(9)	19.0796(19)
α / °	90	90	90	90	90
β / °	99.8920(10)	99.6540(10)	99.2560(10)	118.4680(10)	92.053(8)
γ / °	90	90	90	90	90
Volume / Å^3	2115.8(2)	2300.8(3)	2140.9(3)	4307.5(3)	4412.4(7)
Z	4	4	4	8	8
D_c / Mg/m^3	1.542	1.392	1.573	1.441	1.406
Absorption coefficient / mm^{-1}	1.233	1.135	1.225	1.210	1.181
F(000)	984	988	1016	1904	1904
Crystal size / mm	0.23×0.21×0.17	0.33×0.29×0.24	0.27×0.24×0.13	0.21×0.19×0.18	0.20×0.20×0.19
θ range / (°)	1.868 ~ 27.595	2.53 ~ 25.10	2.13 ~ 25.10	3.37 ~ 25.10	2.84 ~ 25.10
Limiting indices	$-15\le h\le 14$, $-10\le k\le 11$, $-26\le l\le 20$	-15<=h<=15, -24<=k<=23, -10<=l<=10	-14<=h<=14, 0<=k<=10, 0<=l<=24	$-28\le h\le 28$, $-10\le k\le 10$, $-28\le l\le 27$	$-20\le h\le 18$, $-16\le k\le 16$, $-16\le l\le 22$
Reflections collected / unique	12485 / 4838 [R_{int} = 0.0156]	23777 / 4088 [R_{int} = 0.0225]	3807 / 3807 [R_{int} = 0.0271]	27454 / 3829 [R_{int} = 0.0751]	11294 / 3912 [R_{int} = 0.0329]

Completeness	99.8 %	99.8 %	99.9 %	99.8 %	99.9 %
Max. and min. transmission	0.7646 and 0.8177	0.7723 and 0.7058	0.8570 and 0.7333	0.8116 and 0.7852	0.8067 and 0.7980
Data / restraints / parameters	4838 / 0 / 264	4088 / 28 / 251	3807 / 0 / 273	3829 / 1 / 248	3912 / 1 / 239
Goodness-of-fit on F^2	1.102	1.073	1.185	1.062	1.023
Final R indices [I>2δ(I)]	R_1 = 0.0185, wR_2 = 0.0458	R_1 = 0.0431, wR_2 = 0.1131	R_1 = 0.0448, wR_2 = 0.0985	R_1 = 0.0370, wR_2 = 0.0927	R_1 = 0.0372, wR_2 = 0.0857
R indices (all data)	R_1 = 0.0210, wR_2 = 0.0472	R_1 = 0.0496, wR_2 = 0.1168	R_1 = 0.0507, wR_2 = 0.1008	R_1 = 0.0451, wR_2 = 0.0961	R_1 = 0.0629, wR_2 = 0.0954
$\Delta\rho_{max}$ and $\Delta\rho_{min}$ / (e·Å^{-3})	0.371 and -0.424	1.181 and -0.493	0.894 and -1.062	0.869 and -0.701	0.599 and -0.335

Table S2 Crystallographic data for **4a-5b**.

**Complexes**	**4a**	**4b**	**5a**	**5b**
Empirical formula	$C_{24}H_{22}N_2O_3Sn$	$C_{21}H_{34}N_2O_4Sn$	$C_{24}H_{22}N_2O_4Sn$	$C_{21}H_{34}N_2O_5Sn$
Formula weight	505.13	497.19	521.13	513.19
Temperature / K	296(2)	296(2)	296(2)	296(2)
Wavelength / Å	0.71073	0.71073	0.71073	0.71073
Crystal system	Monoclinic	Triclinic	Orthorhombic	Triclinic
space group	$P2_1/n$	P-1	$P2_12_12_1$	P-1
a / Å	13.093(3)	9.939(4)	8.950(4)	9.7696(8)
b / Å	8.9534(17)	11.174(5)	13.753(7)	10.9742(9)
c / Å	19.338(4	11.607(5)	18.650(9)	11.6713(9)
α / °	90	75.776(5)	90	74.4910(10)
β / °	100.208(3)	84.118(5)	90	86.0210(10)
γ / °	90	76.716(5)	90	86.6250(10)
Volume / Å^3	2231.0(7)	1214.7(9)	2296(2)	1201.79(17)
Z	4	2	4	2
D_c / Mg/m^3	1.504	1.359	1.508	1.418
Absorption coefficient / mm^{-1}	1.172	1.077	1.145	1.094
F(000)	1016	512	1048	528
Crystal size / mm	0.23×0.23×0.22	0.23×0.23×0.21	0.24×0.24×0.22	0.24×0.22×0.20

θ range / (°)	1.74 ~ 25.10	2.32 ~ 25.10	1.84~ 25.10	2.69 ~ 25.10
Limiting indices	$-15\leq h\leq 15$, $-10\leq k\leq 10$, $-19\leq l\leq 23$	$-11\leq h\leq 11$, $-13\leq k\leq 13$, $-13\leq l\leq 13$	$-10\leq h\leq 10$, $-12\leq k\leq 16$, $-22\leq l\leq 22$	$-11\leq h\leq 11$, $-13\leq k\leq 13$, $-13\leq l\leq 13$
Reflections collected / unique	10965 / 3966 [R_{int} = 0.0164]	12454 / 4318 [R_{int} = 0.0230]	11548 / 4070 [R_{int} = 0.0179]	12388 / 4264 [R_{int} = 0.0171]
Completeness	99.8 %	99.6 %	100.0 %	99.6 %
Max. and min. transmission	0.7826 and 0.7743	0.8054 and 0.7897	0.7868 and 0.7707	0.8109 and 0.7792
Data / restraints / parameters	3966 / 0 / 273	4318 / 9 / 262	4070 / 0 / 282	4264 / 1 / 271
Goodness-of-fit on F^2	1.113	1.070	1.148	1.063
Final R indices [I>2δ(I)]	R_1 = 0.0190, wR_2 = 0.0481	R_1 = 0.0351, wR_2 = 0.1018	R_1 = 0.0167, wR_2 = 0.0408	R_1 = 0.0246, wR_2 = 0.0665
R indices (all data)	R_1 = 0.0215, wR_2 = 0.0492	R_1 = 0.0402, wR_2 = 0.1060	R_1 = 0.0169, wR_2 = 0.0409	R_1 = 0.0267, wR_2 = 0.0681
$\Delta\rho_{max}$ and $\Delta\rho_{min}$ / (e·Å^{-3})	0.242 and -0.560	0.816 and -0.510	0.679 and -0.607	0.601 and -0.416

Table S3 Crystallographic data for **6a-7b**.

**Complexes**	**6a**	**6b**	**7a**	**7b**
Empirical formula	$C_{27}H_{28}N_2O_3Sn$	$C_{24}H_{40}N_2O_4Sn$	$C_{48}H_{46}N_6O_{12}Sn_2$	$C_{40}H_{62}N_6O_{12}Sn_2$
Formula weight	547.20	539.27	1136.29	1056.34
Temperature / K	293(2)	296(2)	296(2)	296(2)
Wavelength / Å	0.71073	0.71073	0.71073	0.71073
Crystal system	Triclinic	Triclinic	Monoclinic	Tetragonal
space group	P-1	P-1	$P2_1/c$	$I4_1/a$
a / Å	10.242(4)	10.637(3)	12.375(6)	25.542(10)
b / Å	11.021(4)	12.087(3)	11.479(6)	25.542(10)
c / Å	12.065(4)	12.177(3)	16.659(8)	15.019(7)
α / °	104.799(4)	100.935(3)	90	90
β / °	102.901(4)	108.490(3)	100.266(7)	90
γ / °	92.927(4)	104.481(3)	90	90
Volume / Å^3	1275.0(8)	1374.5(6)	2328(2)	9798(7)
Z	2	2	2	8
D_c / Mg/m^3	1.425	1.303	1.621	1.432
Absorption	1.031	0.957	1.143	1.080

coefficient / mm^{-1}				
$F(000)$	556	520	1144	4320
Crystal size / mm	0.22×0.22×0.21	0.21×0.21×0.20	0.22×0.20×0.20	0.23×0.22×0.22
θ range / (°)	2.65 ~ 25.10	2.76 ~ 25.10	1.67 ~ 25.10	2.75 ~ 25.10
Limiting indices	$-12\le h\le 12$, $-13\le k\le 13$, $-14\le l\le 14$	$-12\le h\le 12$, $-14\le k\le 14$, $-14\le l\le 14$	$-14\le h\le 14$, $-11\le k\le 13$, $-19\le l\le 11$	$-30\le h\le 30$, $-30\le k\le 30$, $-17\le l\le 17$
Reflections collected / unique	13133 / 4530 [R_{int} = 0.0146]	14057 / 4865 [R_{int} = 0.0206]	9472 / 4126 [R_{int} = 0.0189]	49687 / 436 [R_{int} = 0.0279]
Completeness	99.6 %	99.4 %	99.5 %	99.9 %
Max. and min. transmission	0.8125 and 0.8049	0.8316 and 0.8242	0.8036 and 0.7871	0.7971 and 0.7893
Data / restraints / parameters	4530 / 0 / 302	4865 / 162 / 372	4126 / 7 / 313	4361 / 26 / 279
Goodness-of-fit on F^2	1.057	1.071	1.039	1.058
Final R indices [I>2$\delta(I)$]	R_1 = 0.0206, wR_2 = 0.0554	R_1 = 0.0378, wR_2 = 0.1099	R_1 = 0.0245, wR_2 = 0.0618	R_1 = 0.0437, wR_2 = 0.1325
R indices (all data)	R_1 = 0.0218, wR_2 = 0.0564	R_1 = 0.0459, wR_2 = 0.1177	R_1 = 0.0295, wR_2 = 0.0641	R_1 = 0.0523, wR_2 = 0.1400
$\Delta\rho_{max}$ and $\Delta\rho_{min}$ / (e·Å^{-3})	0.596 and -0.241	0.639 and -0.426	0.422 and -0.645	1.235 and -0.993

Table S4 Crystallographic data for **8a-9b**.

**Complexes**	**8a**	**8b**	**9a**	**9b**
Empirical formula	$C_{21}H_{18}N_2O_4Sn$	$C_{18}H_{30}N_2O_5Sn$	$C_{21}H_{18}N_2O_3SSn$	$C_{18}H_{30}N_2O_4SSn$
Formula weight	481.06	473.13	497.12	489.19
Temperature / K	293(2)	296(2)	296(2)	296(2)
Wavelength / Å	0.71073	0.71073	0.71073	0.71073
Crystal system	Monoclinic	Monoclinic	Monoclinic	Monoclinic
space group	$P2_1/c$	$P2_1/c$	$P2_1/c$	$P2_1/c$
a / Å	10.777(4)	11.6256(6)	11.5889(14)	12.4982(8)
b / Å	9.044(3)	24.3106(13)	8.9899(10)	20.7695(14)
c / Å	20.784(7)	16.0727(8)	20.490(2)	8.7242(6)
α / °	90	90	90	90
β / °	99.399(5)	107.8840(10)	100.885(2)	101.5360(10)
γ / °	90	90	90	90
Volume / Å^3	1998.6(12)	4323.1(4)	2096.3(4)	2218.9(3)

Z	4	8	4	4
D_c / Mg/m^3	1.599	1.454	1.575	1.464
Absorption coefficient / mm^{-1}	1.307	1.210	1.342	1.269
F(000)	960	1936	992	1000
Crystal size / mm	0.22×0.22×0.20	0.23×0.22×0.22	0.22×0.20×0.18	0.23×0.23×0.22
θ range / (°)	2.30 ~ 25.10	1.57 ~ 25.10	1.88 ~ 25.10	1.93 ~ 25.10
Limiting indices	$-8\leq h\leq 12$, $-10\leq k\leq 10$, $-22\leq l\leq 24$	$-10\leq h\leq 13$, $-29\leq k\leq 28$, $-19\leq l\leq 18$	$-13\leq h\leq 13$, $-10\leq k\leq 10$, $-19\leq l\leq 24$	$-11\leq h\leq 14$, $-24\leq k\leq 23$, $-10\leq l\leq 10$
Reflections collected / unique	8097 / 3533 [R_{int} = 0.0110]	21936 / 7701 [R_{int} = 0.0194]	10354 / 3727 [R_{int} = 0.0140]	11003 / 3939 [R_{int} = 0.0195]
Completeness	99.4 %	100 %	99.7 %	99.7 %
Max. and min. transmission	0.7800 and 0.7619	0.7767 and 0.7683	0.7942 and 0.7568	0.7677 and 0.7591
Data / restraints / parameters	3533 / 10 / 294	7701 / 40 / 485	3727 / 40 / 255	3939 / 7 / 273
Goodness-of-fit on F^2	1.075	1.044	1.106	1.122
Final R indices [I>2δ(I)]	R_1 = 0.0222, wR_2 = 0.0487	R_1 = 0.0266, wR_2 = 0.0622	R_1 = 0.0285, wR_2 = 0.0687	R_1 = 0.0307, wR_2 = 0.0748
R indices (all data)	R_1 = 0.0276, wR_2 = 0.0507	R_1 = 0.0353, wR_2 = 0.0652	R_1 = 0.0331, wR_2 = 0.0705	R_1 = 0.0361, wR_2 = 0.0774
$\Delta\rho_{max}$ and $\Delta\rho_{min}$ / (e·Å^{-3})	0.246 and -0.318	0.424 and -0.325	0.760 and -0.651	0.423 and -0.459

2.2 The selected bond length(Å) and angles(°) for **1a-2b, 3b-9b**.

Table S5 The selected bond length(Å) and angles(°) for **1a-2b, 3b-9b**.

**1a**					
Sn1-C12	2.1171(17)	Sn1-C18	2.1181(17)	Sn1-N2	2.2275(13)
Sn1-O2	2.1678(11)	Sn1-O1	2.2570(11)	Sn1-O3^i	2.4020(12)
C12-Sn1-C18	153.86(7)	O2-Sn1-N2	72.33(4)	C12-Sn1-O3^i	84.69(5)
C12-Sn1-O2	101.42(6)	C12-Sn1-O1	88.90(6)	C18-Sn1-O3^i	48(5)
C18-Sn1-O2	97.05(6)	C18-Sn1-O1	88.33(6)	O2-Sn1-O3^i	72.31(4)
C12-Sn1-N2	97.38(6)	O2-Sn1-O1	140.88(4)	N2-Sn1-O3^i	144.27(4)
C18-Sn1-N2	105.81(5)	N2-Sn1-O1	68.94(4)	O1-Sn1-O3^i	146.72(4)
Symmetry codes: i: -x+3/2, y-1/2, -z+1/2					
**1b**					
Sn1-C16	2.106(5)	Sn1-O1	2.171(3)	Sn1-O2	2.287(3)

Sn1-C12	2.114(5)	Sn1-N2	2.244(4)	Sn1-O4	2.501(4)
Sn1-O2^{i}	2.8097(2)				
C16-Sn1-C12	158.8(2)	O1-Sn1-N2	69.96(13)	C16-Sn1-O4	84.26(19)
C16-Sn1-O1	95.85(18)	C16-Sn1-O2	92.19(17)	C12-Sn1-O4	79.97(19)
C12-Sn1-O1	94.4(2)	C12-Sn1-O2	91.69(19)	O1-Sn1-O4	77.58(14)
C16-Sn1-N2	101.43(17)	O1-Sn1-O2	140.11(12)	N2-Sn1-O4	147.42(14)
C12-Sn1-N2	99.46(17)	N2-Sn1-O2	70.15(12)	O2-Sn1-O4	142.21(13)
O2-Sn1-O2^{i}	67.151(2)	N2-Sn1-O2^{i}	137.286(3)	O1-Sn1-O2^{i}	152.708(3)
O4-Sn1-O2^{i}	75.124(2)	C12-Sn1-O2^{i}	80.896(2)	C16-Sn1-O2^{i}	81.480(2)
Symmetry codes: i: -x, -y+1, -z+1					
**2a**					
Sn1-C12	2.109(6)	Sn1-O2	2.169(3)	Sn1-O1	2.282(3)
Sn1-C18	2.119(5)	Sn1-N2	2.238(4)	Sn1-O3^{i}	2.387(3)
C12-Sn1-C18	155.5(2)	O2-Sn1-N2	71.91(13)	C12-Sn1-O3^{i}	85.12(17)
C12-Sn1-O2	100.41(18)	C12-Sn1-O1	88.79(18)	C18-Sn1-O3^{i}	84.23(16)
C18-Sn1-O2	97.31(18)	C18-Sn1-O1	88.23(17)	O2-Sn1-O3^{i}	72.42(12)
C12-Sn1-N2	96.76(18)	O2-Sn1-O1	140.49(12)	N2-Sn1-O3^{i}	144.02(13)
C18-Sn1-N2	104.80(18)	N2-Sn1-O1	68.87(13)	O1-Sn1-O3^{i}	147.04(12)
Symmetry codes: i: -x+1/2, y+1/2, -z+1/2					
**2b**					
Sn1-C12	2.104(4)	Sn1-O2	2.155(2)	Sn1-O1	2.294(2)
Sn1-C16	2.116(4)	Sn1-N1	2.243(3)	Sn1-O3^{i}	2.464(2)
C12-Sn1-C16	147.3(2)	O2-Sn1-N1	71.88(8)	C12-Sn1-O3^{i}	86.63(12)
C12-Sn1-O2	104.17(14)	C12-Sn1-O1	83.95(12)	C16-Sn1-O3^{i}	83.58(14)
C16-Sn1-O2	101.96(17)	C16-Sn1-O1	88.58(16)	O2-Sn1-O3^{i}	71.05(8)
C12-Sn1-N1	106.09(13)	O2-Sn1-O1	139.97(8)	O2-Sn1-O4^{i}	142.72(9)
C16-Sn1-N1	100.47(15)	N1-Sn1-O1	68.24(9)	O2-Sn1-O5^{i}	148.97(8)
Symmetry codes: i: -x+3/2, y+1/2, -z+3/2					
**4b**					
Sn1-C13	2.130(4)	Sn1-O1	2.184(3)	Sn1-O2	2.332(3)
Sn1-C17	2.135(4)	Sn1-N1	2.254(3)	Sn1-O4	2.510(3)
Sn1-O2^{i}	2.8102(30)				
C13-Sn1-C17	159.24(19)	O1-Sn1-N1	70.45(10)	C13-Sn1-O4	81.63(17)
C13-Sn1-O1	95.01(15)	C13-Sn1-O2	90.76(15)	C17-Sn1-O4	84.38(16)
C17-Sn1-O1	97.07(14)	C17-Sn1-O2	90.65(14)	O1-Sn1-O4	78.23(10)
C13-Sn1-N1	99.96(15)	O1-Sn1-O2	140.32(9)	N1-Sn1-O4	148.67(11)
C17-Sn1-N1	99.96(14)	N1-Sn1-O2	69.89(9)	O2-Sn1-O4	141.40(10)
C13-Sn1-C17	159.24(19)	O1-Sn1-N1	70.45(10)	C13-Sn1-O4	81.63(17)
C13-Sn1-O2^{i}	80.327(133)	C17-Sn1-O2^{i}	81.414(138)	O4-Sn1-O2^{i}	75.684(98)
O2-Sn1-O2^{i}	65.737(89)				
Symmetry codes: i: -x+1, -y+1, -z+1					
**3b**					
Sn1-C12	2.103(4)	Sn1-O2	2.183(3)	Sn1-O3	2.224(3)
Sn1-C16	2.104(4)	Sn1-N2	2.190(3)	Sn1-O3^{i}	2.9911(28)

C12-Sn1-C16	145.73(19)	C16-Sn1-N2	111.09(17)	C16-Sn1-O3	95.42(16)
C12-Sn1-O2	95.06(15)	O2-Sn1-N2	70.85(11)	O2-Sn1-O3	142.53(10)
C16-Sn1-O2	94.91(16)	C12-Sn1-O3	96.31(15)	N2-Sn1-O3	71.82(11)
C12-Sn1-N2	103.16(14)	O3-Sn1-O3^{i}	62.291(95)	C12-Sn1-O3^{i}	78.908(139)
C16-Sn1-O3^{i}	78.350(132)	O2-Sn1-O3^{i}	155.170(92)		

Symmetry codes: i: -x, -y, -z

4a

Sn1-C19	2.1112(19)	Sn1-N1	2.2218(15)	Sn1-O2	2.1557(13)
Sn1-C13	2.1166(19)	Sn1-O1	2.2315(13)	Sn1-O3^{i}	2.4170(13)
C19-Sn1-C13	154.50(8)	O2-Sn1-N1	72.55(5)	C19-Sn1-O3^{i}	83.03(6)
C19-Sn1-O2	97.10(7)	C19-Sn1-O1	88.18(7)	C13-Sn1-O3^{i}	84.63(6)
C13-Sn1-O2	100.40(7)	C13-Sn1-O1	89.58(7)	O2-Sn1-O3^{i}	72.71(5)
C19-Sn1-N1	106.22(7)	O2-Sn1-O1	141.43(5)	N1-Sn1-O3^{i}	144.88(5)
C13-Sn1-N1	96.68(6)	N1-Sn1-O1	69.32(5)	O1-Sn1-O3^{i}	145.74(5)

Symmetry codes: i: -x+3/2, y-1/2, -z+1/2

5a

Sn1-C13	2.141(3)	Sn1-O2	2.1814(17)	Sn1-O1	2.2915(18)
Sn1-C19	2.147(3)	Sn1-N1	2.247(2)	Sn1-O3^{i}	2.4474(19)
C13-Sn1-C19	151.93(9)	O2-Sn1-N1	72.22(7)	C13-Sn1-O3^{i}	84.64(8)
C13-Sn1-O2	100.08(8)	C13-Sn1-O1	88.29(8)	C19-Sn1-O3^{i}	84.19(7)
C19-Sn1-O2	100.34(8)	C19-Sn1-O1	87.73(8)	O2-Sn1-O3^{i}	70.61(5)
C13-Sn1-N1	97.76(8)	O2-Sn1-O1	141.28(6)	N1-Sn1-O3^{i}	142.58(6)
C19-Sn1-N1	106.70(8)	N1-Sn1-O1	69.20(7)	O1-Sn1-O3^{i}	148.10(5)

Symmetry codes: i: x-1/2, -y+1/2, -z

5b

Sn1-C17	2.119(3)	Sn1-O1	2.1681(18)	Sn1-O2	2.3019(17)
Sn1-C13	2.122(3)	Sn1-N1	2.241(2)	Sn1-O5	2.487(2)
Sn1-O2^{i}	2.787(2)				
C17-Sn1-C13	159.30(12)	O1-Sn1-N1	70.19(7)	C17-Sn1-O5	86.05(11)
C17-Sn1-O1	96.84(10)	C17-Sn1-O2	90.04(10)	C13-Sn1-O5	80.04(10)
C13-Sn1-O1	95.06(9)	C13-Sn1-O2	91.54(9)	O1-Sn1-O5	77.57(7)
C17-Sn1-N1	98.67(10)	O1-Sn1-O2	140.38(6)	N1-Sn1-O5	147.73(8)
C13-Sn1-N1	101.28(9)	N1-Sn1-O2	70.20(7)	O2-Sn1-O5	141.99(7)
C13-Sn1-O2^{i}	81.64(7)	C17-Sn1-O2^{i}	80.09(10)	O5-Sn1-O2^{i}	75.64(6)
O2-Sn1-O2^{i}	66.45(6)				

Symmetry codes: i: -x+1, -y+1, -z+1

6a

Sn1-C16	2.108(2)	Sn1-O2	2.1230(15)	Sn1-N1	2.1498(17)
Sn1-C22	2.108(2)	Sn1-O1	2.1451(15)		
C16-Sn1-C22	122.93(8)	C22-Sn1-O1	96.78(7)	C22-Sn1-N1	120.57(7)
C16-Sn1-O2	98.37(7)	O2-Sn1-O1	147.04(6)	O2-Sn1-N1	74.80(6)
C22-Sn1-O2	99.04(8)	C16-Sn1-N1	116.40(7)	O1-Sn1-N1	72.26(6)
C16-Sn1-O1	96.94(7)				

6b

Sn1-C20	2.101(5)	Sn1-O2	2.310(3)	Sn1-O1	2.175(3)
Sn1-C16	2.115(5)	Sn1-O4	2.443(4)	Sn1-N1	2.245(3)
Sn1-O2^{i}	2.7598(29)				
C20-Sn1-C16	160.5(2)	O1-Sn1-N1	70.06(11)	C20-Sn1-O4	81.9(2)
C20-Sn1-O1	94.23(17)	C20-Sn1-O2	91.67(17)	C16-Sn1-O4	84.3(2)
C16-Sn1-O1	96.23(18)	C16-Sn1-O2	90.9(2)	O2-Sn1-O4	142.15(12)
C20-Sn1-N1	99.98(17)	O1-Sn1-O2	139.83(10)	O1-Sn1-O4	78.00(12)
C16-Sn1-N1	99.0(2)	N1-Sn1-O2	69.78(10)	N1-Sn1-O4	148.06(12)
C16-Sn1-O2^{i}	81.898(182)	C20-Sn1-O2^{i}	81.409(149)	O4-Sn1-O2^{i}	75.377(128)
O2-Sn1-O2^{i}	66.776(82)				
Symmetry codes: i: -x+1, -y+2, -z+1					
**7a**					
Sn1-C18	2.117(3)	Sn1-O1	2.1730(18)	Sn1-O6	2.299(2)
Sn1-C12	2.131(3)	Sn1-N1	2.273(2)	Sn1-O2	2.3854(18)
Sn1-O2^{i}	2.566(1)				
C18-Sn1-C12	171.04(9)	O1-Sn1-N1	69.51(7)	C18-Sn1-O2	88.82(9)
C18-Sn1-O1	92.42(9)	C18-Sn1-O6	89.56(10)	C12-Sn1-O2	89.92(9)
C12-Sn1-O1	94.47(9)	C12-Sn1-O6	86.34(10)	O1-Sn1-O2	137.75(6)
C18-Sn1-N1	95.09(9)	O1-Sn1-O6	77.54(7)	N1-Sn1-O2	68.32(7)
C12-Sn1-N1	92.68(9)	N1-Sn1-O6	146.87(7)	O6-Sn1-O2	144.71(7)
C18-Sn1-O2^{i}	85.39(2)	C12-Sn1-O2^{i}	85.95(2)	O1-Sn1-O2^{i}	155.03(2)
O2-Sn1-O2^{i}	67.16(2)	N1-Sn1-O2^{i}	135.46(2)	O6-Sn1-O2^{i}	77.57(2)
Symmetry codes: i: -x+1, -y, -z+2					
**7b**					
Sn1-C16	2.081(6)	Sn1-O1	2.194(3)	Sn1-O2	2.329(3)
Sn1-C12	2.123(7)	Sn1-N1	2.256(3)	Sn1-O6	2.457(4)
Sn1-O2^{i}	2.7294(33)				
C16-Sn1-C12	160.0(3)	O1-Sn1-N1	69.69(12)	C16-Sn1-O6	82.7(2)
C16-Sn1-O1	94.55(19)	C16-Sn1-O2	91.9(2)	C12-Sn1-O6	84.1(3)
C12-Sn1-O1	96.8(2)	C12-Sn1-O2	90.3(2)	O1-Sn1-O6	76.28(13)
C16-Sn1-N1	101.60(18)	O1-Sn1-O2	138.98(10)	N1-Sn1-O6	145.91(13)
C12-Sn1-N1	97.7(3)	N1-Sn1-O2	69.33(11)	O2-Sn1-O6	144.74(12)
C12-Sn1-O2^{i}	81.440(215)	C16-Sn1-O2^{i}	81.269(147)	O6-Sn1-O2^{i}	78.316(124)
Symmetry codes: i: -x+3/2, -y+3/2, -z+3/2					
**8a**					
Sn1-C16	2.132(3)	Sn1-N1	2.2405(19)	Sn1-O2	2.1734(17)
Sn1-C10	2.133(2)	Sn1-O1	2.2553(18)	Sn1-O3^{i}	2.4090(18)
C16-Sn1-C10	154.96(10)	O2-Sn1-N1	72.25(6)	C16-Sn1-O3^{i}	85.02(8)
C16-Sn1-O2	101.39(8)	C16-Sn1-O1	87.36(8)	C10-Sn1-O3^{i}	84.33(8)
C10-Sn1-O2	97.42(8)	C10-Sn1-O1	88.16(8)	O2-Sn1-O3^{i}	74.27(6)
C16-Sn1-N1	97.02(8)	O2-Sn1-O1	141.45(6)	N1-Sn1-O3^{i}	146.20(6)
C10-Sn1-N1	104.40(8)	N1-Sn1-O1	69.43(7)	O1-Sn1-O3^{i}	144.25(6)
Symmetry codes: i: -x+1/2, y+1/2, -z+1/2					
**8b**					

Sn1-C10	2.115(3)	Sn1-C14	2.120(3)	Sn1-O1	2.1771(18)
Sn1-N1	2.245(2)	Sn1-O2	2.3206(18)	Sn1-O5	2.428(2)
Sn2-C32	2.114(3)	Sn2-C28	2.123(3)	Sn2-O6	2.1814(19)
Sn2-N3	2.255(2)	Sn2-O7	2.2944(18)	Sn2-O10	2.484(2)
Sn1-O7	2.8216(17)	Sn2-O2	2.7928(17)		
C10-Sn1-C14	160.57(12)	C10-Sn1-O1	96.85(10)	C14-Sn1-O1	95.85(10)
C10-Sn1-N1	99.70(10)	C14-Sn1-N1	98.38(10)	O1-Sn1-N1	70.43(7)
C10-Sn1-O2	89.17(9)	C14-Sn1-O2	90.46(10)	O1-Sn1-O2	140.16(6)
N1-Sn1-O2	69.74(7)	C10-Sn1-O5	85.03(11)	C14-Sn1-O5	83.23(11)
O1-Sn1-O5	78.23(8)	N1-Sn1-O5	148.63(8)	O2-Sn1-O5	141.61(7)
C32-Sn2-C28	160.84(11)	C32-Sn2-O6	93.94(10)	C28-Sn2-O6	95.29(9)
C32-Sn2-N3	101.99(10)	C28-Sn2-N3	96.94(9)	O6-Sn2-N3	70.16(8)
C32-Sn2-O7	91.67(10)	C28-Sn2-O7	92.00(9)	O6-Sn2-O7	140.00(7)
N3-Sn2-O7	69.93(7)	C32-Sn2-O10	80.99(10)	C28-Sn2-O10	84.66(9)
O6-Sn2-O10	77.51(7)	N3-Sn2-O10	147.64(8)	O7-Sn2-O10	142.42(7)
C10-Sn1-O7	79.352(85)	C14-Sn1-O7	82.653(91)	O2-Sn1-O7	66.832(60)
O5-Sn1-O7	74.802(70)	C32-Sn2-O2	82.422(90)	C28-Sn2-O2	81.60(8)
O10-Sn2-O2	74.811(64)	O7-Sn2-O2	67.669(61)		

9a

Sn1-C16	2.122(3)	Sn1-C10	2.125(3)	Sn1-O2	2.174(2)
Sn1-O1	2.261(2)	Sn1-N1	2.234(2)	Sn1-O3^{i}	2.396(2)
C16-Sn1-C10	156.51(13)	O2-Sn1-N1	72.04(8)	C16-Sn1-O3	84.13(10)
C16-Sn1-O2	96.96(11)	C16-Sn1-O1	88.00(11)	C10-Sn1-O3^{i}	85.35(10)
C10-Sn1-O2	100.06(11)	C10-Sn1-O1	88.77(11)	O2-Sn1-O3^{i}	73.42(7)
C16-Sn1-N1	103.85(11)	O2-Sn1-O1	141.23(8)	N1-Sn1-O3^{i}	145.24(8)
C10-Sn1-N1	96.75(11)	N1-Sn1-O1	69.45(8)	O1-Sn1-O3^{i}	145.27(7)

Symmetry codes: i: -x+1/2, y+1/2, -z+1/2

9b

Sn1-C10	2.106(4)	Sn1-O1	2.175(2)	Sn1-O2	2.293(2)
Sn1-C14	2.115(3)	Sn1-N1	2.244(3)	Sn1-O4	2.473(3)
Sn1-O2^{i}	2.7895(22)				
C10-Sn1-C14	159.70(15)	O1-Sn1-N1	70.31(9)	C10-Sn1-O4	80.90(15)
C10-Sn1-O1	94.79(14)	C10-Sn1-O2	90.90(14)	C14-Sn1-O4	84.47(13)
C14-Sn1-O1	95.65(12)	C14-Sn1-O2	92.10(12)	O1-Sn1-O4	77.35(9)
C10-Sn1-N1	99.04(14)	O1-Sn1-O2	140.35(8)	N1-Sn1-O4	147.56(10)
C14-Sn1-N1	100.86(12)	N1-Sn1-O2	70.05(8)	O2-Sn1-O4	142.21(9)
C10-Sn1-O2^{i}	80.829(114)	C14-Sn1-O2^{i}	81.905(112)	O4-Sn1-O2^{i}	75.251(88)
O2-Sn1-O2^{i}	67.011(78)				

Symmetry codes: i: -x+1, -y+1, -z+1

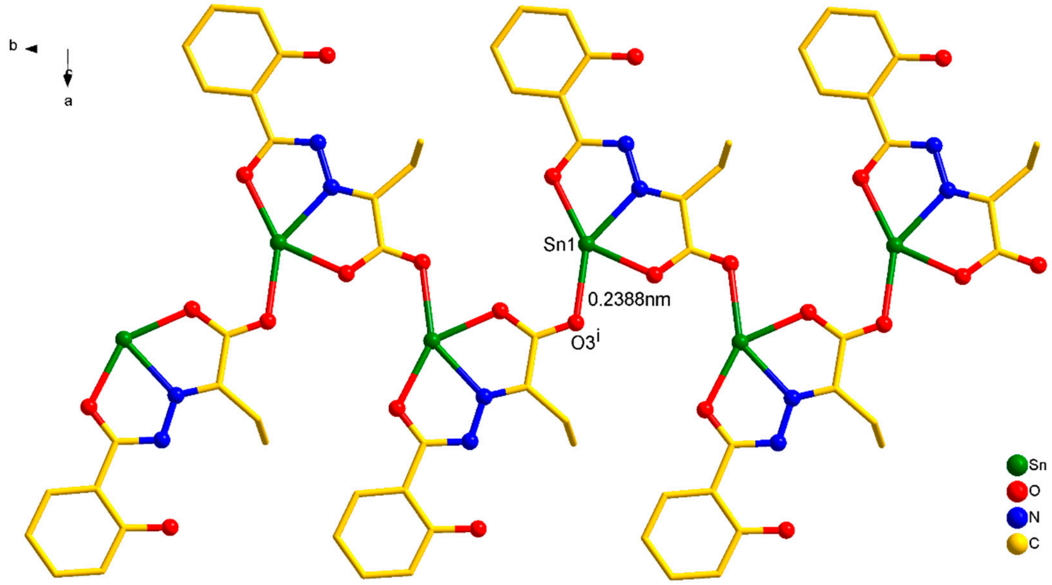

Figure S4 The 1D structures of **2a.**

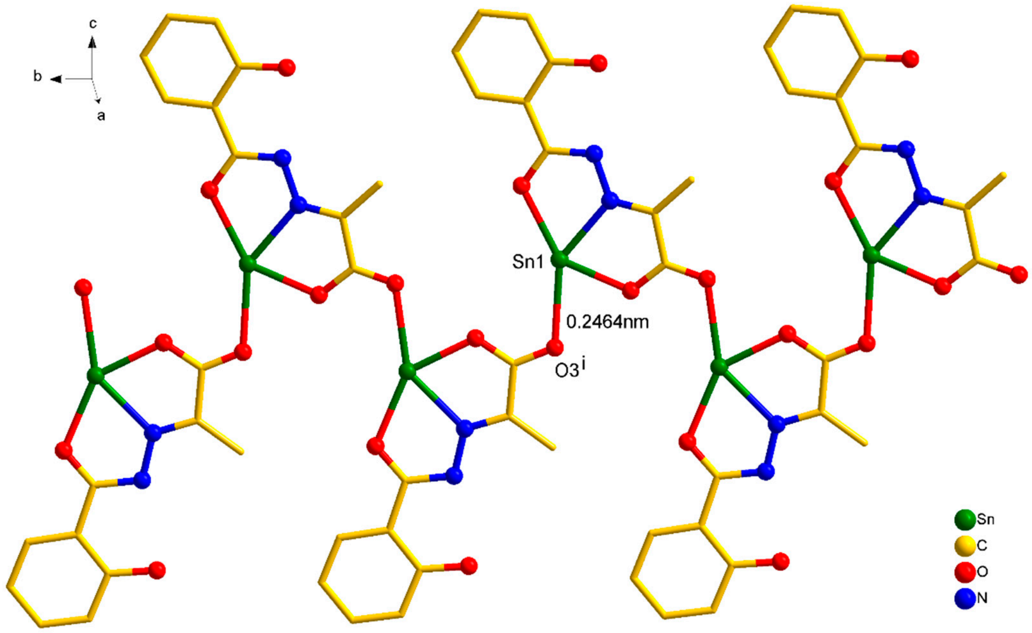

Figure S5 The 1D structures of **2b**.

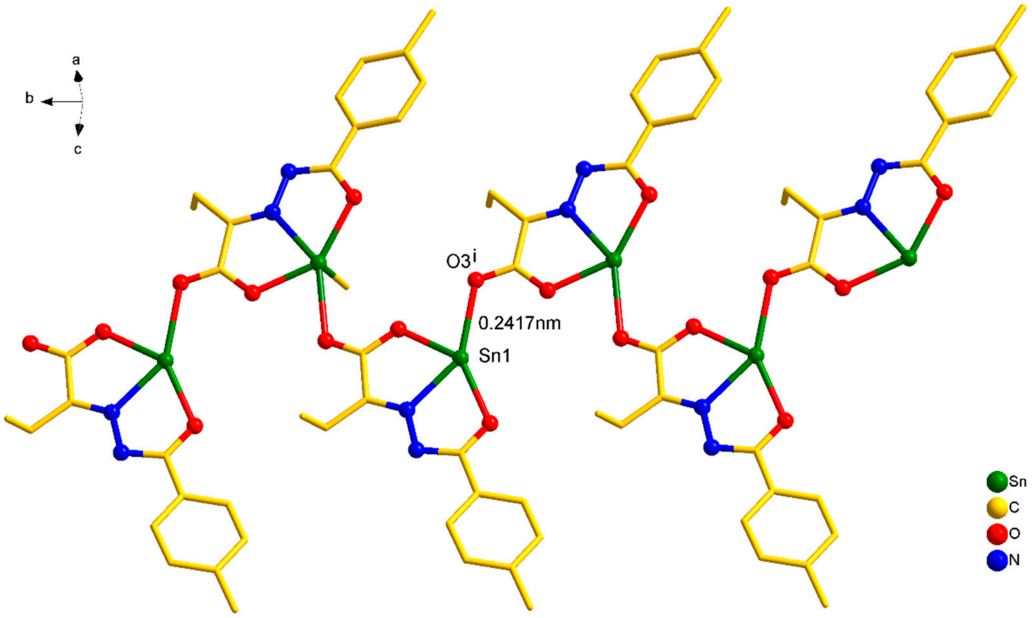

Figure S6 The 1D structures of **4a**.

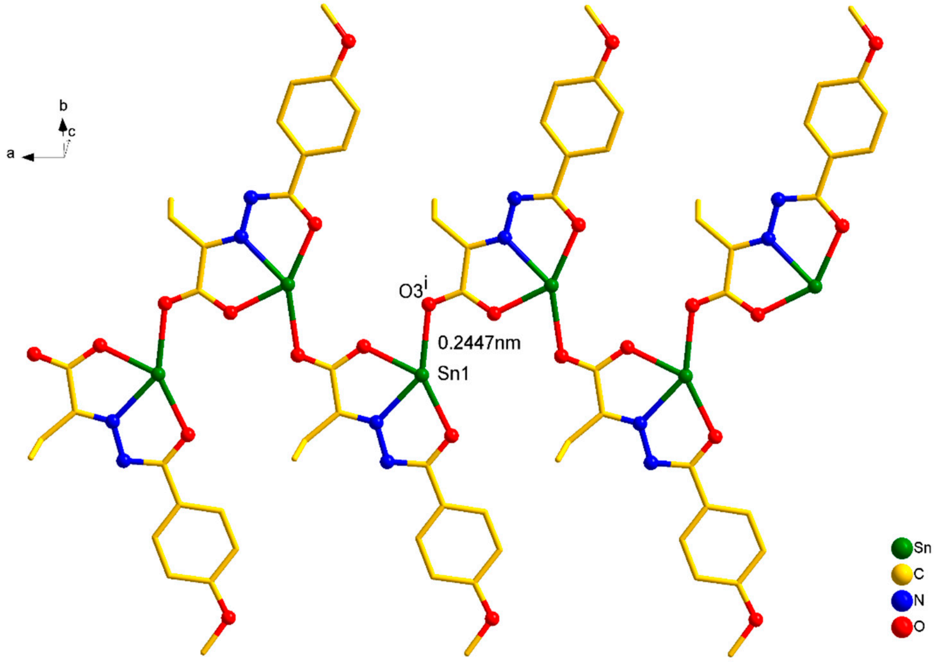

Figure S7 The 1D structures of **5a**.

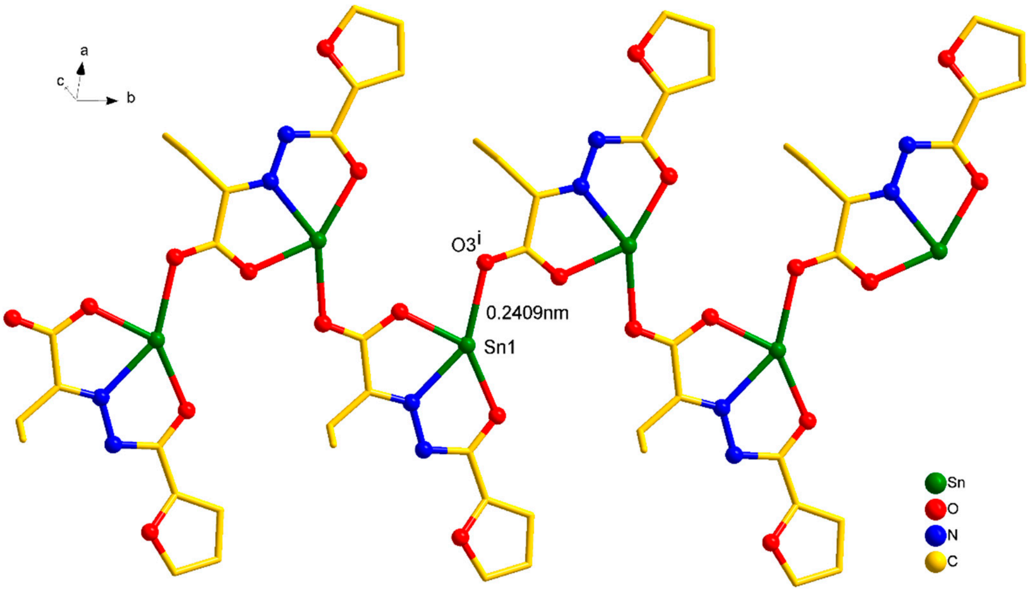

Figure S8 The 1D structures of **8a**.

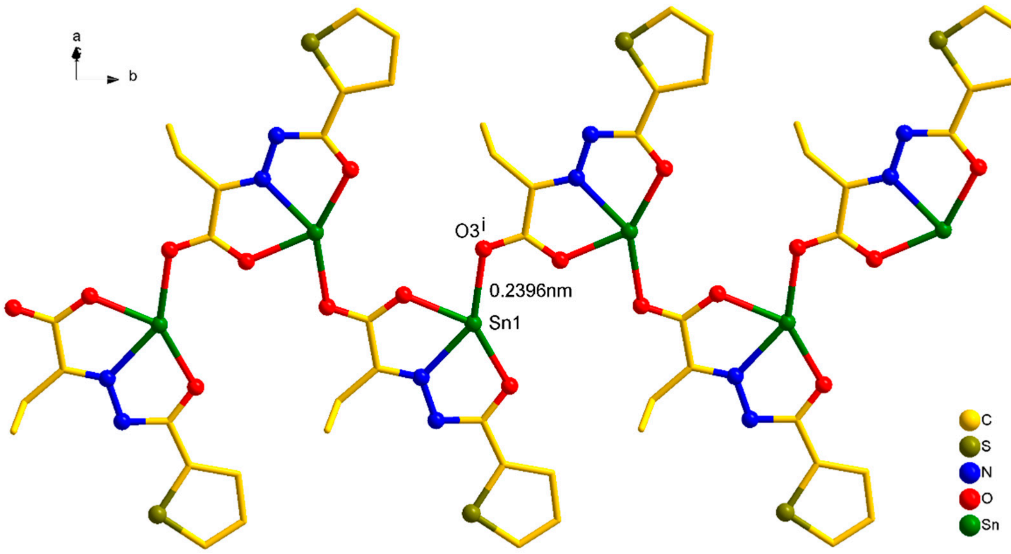

Figure S9 The 1D structures of **9a**.

3 Spectra

3.1 The IR spectra of **1a-9b**.

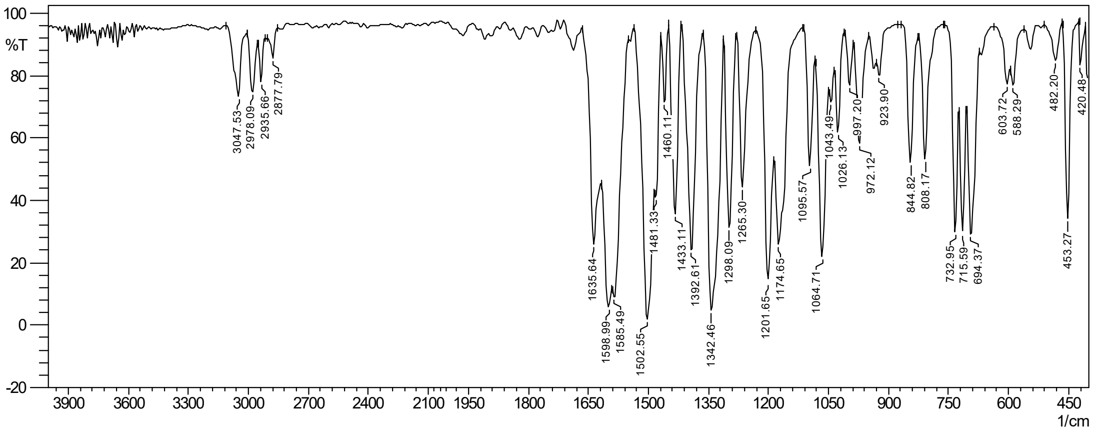

Figure S10 The IR spectra of **1a**

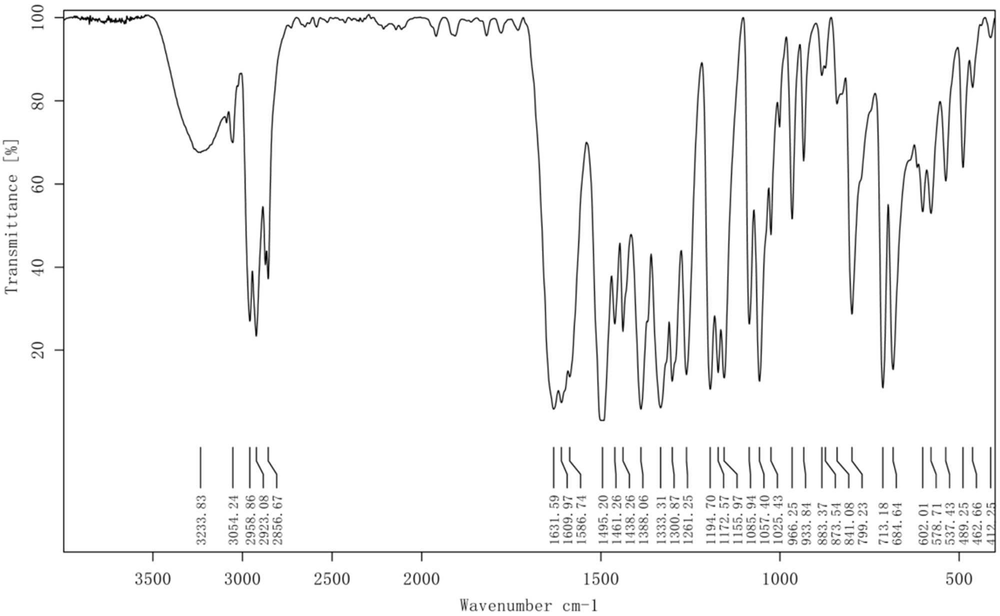

Figure S11 The IR spectra of **1b**

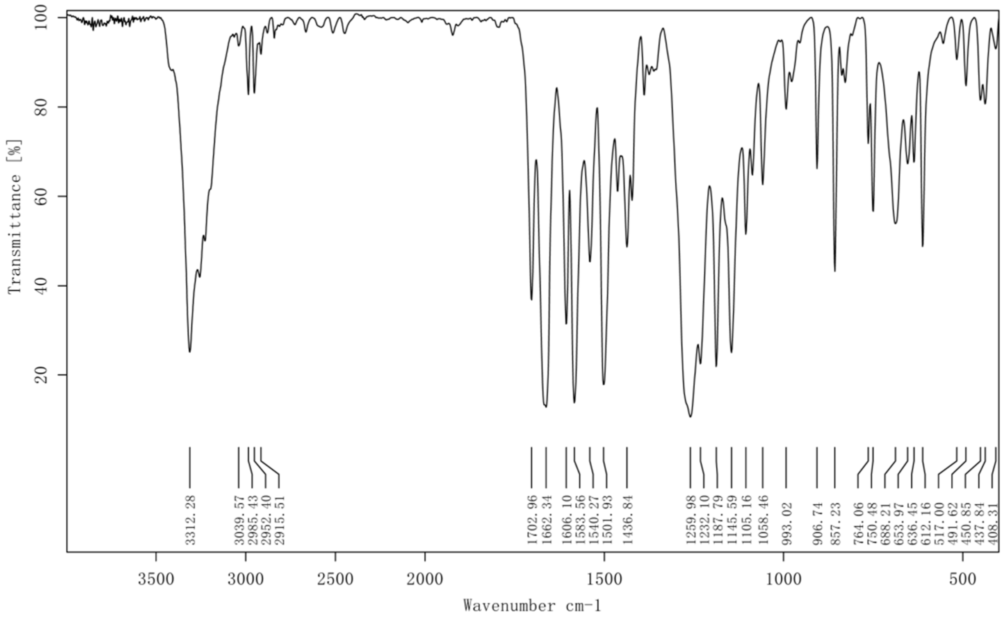

Figure S12 The IR spectra of **2a**

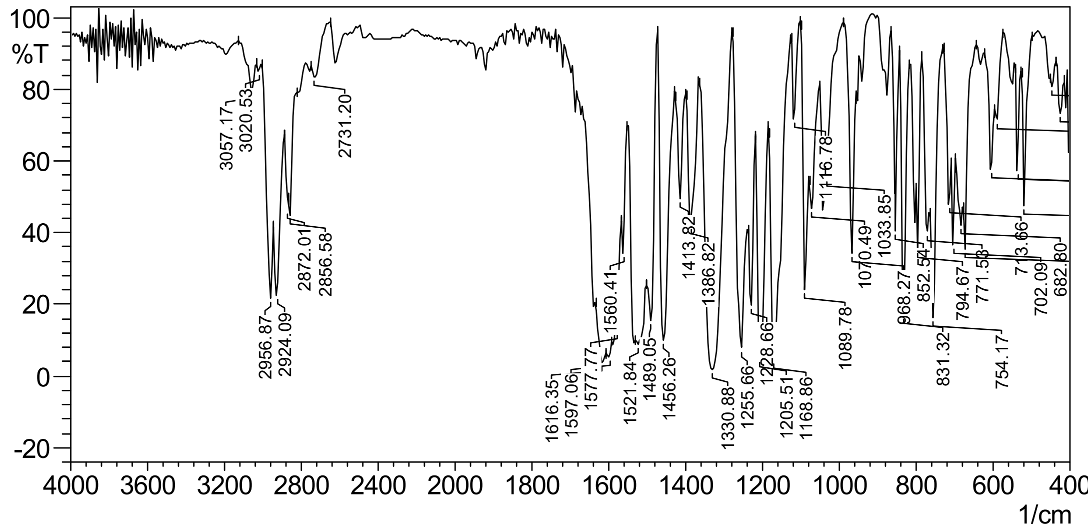

Figure S13 The IR spectra of **2b**

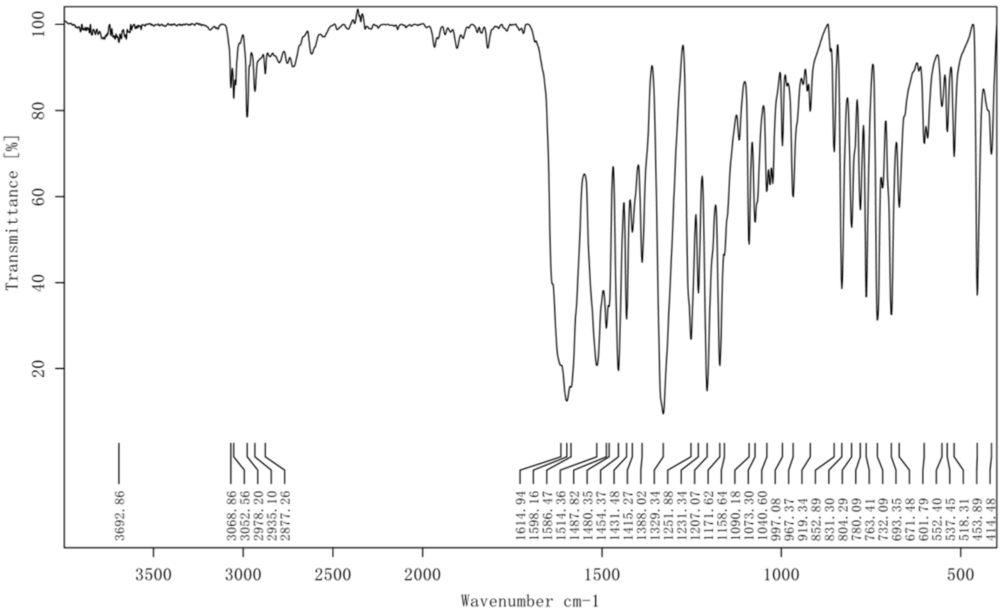

Figure S14 The IR spectra of **3a**

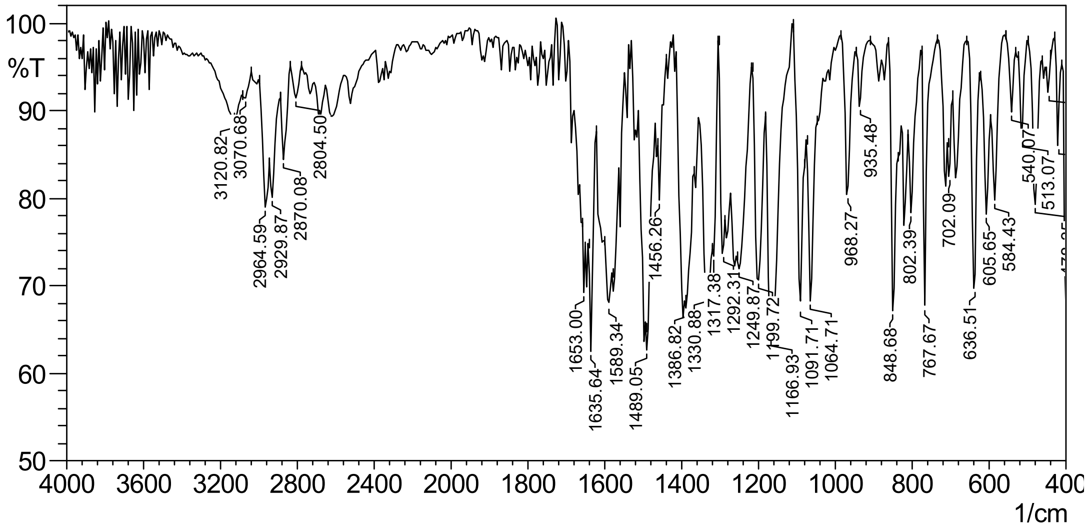

Figure S15 The IR spectra of **3b**

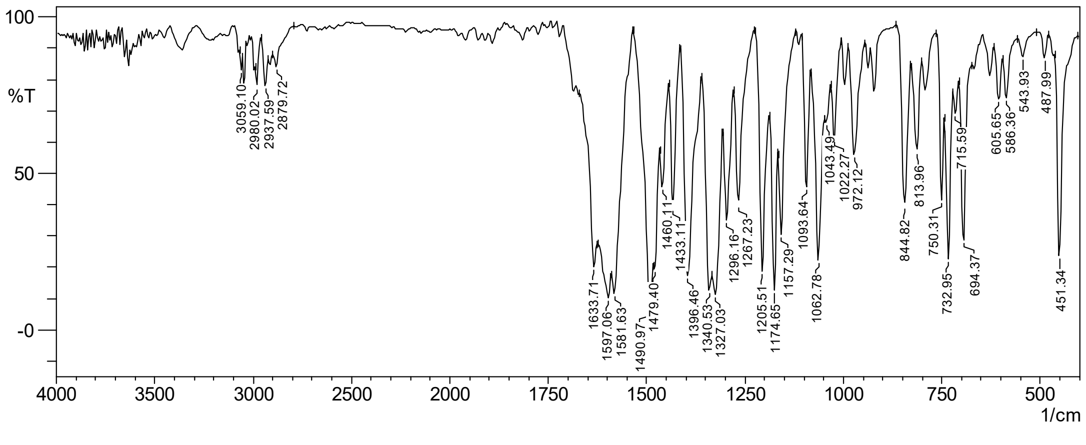

Figure S16 The IR spectra of **4a**

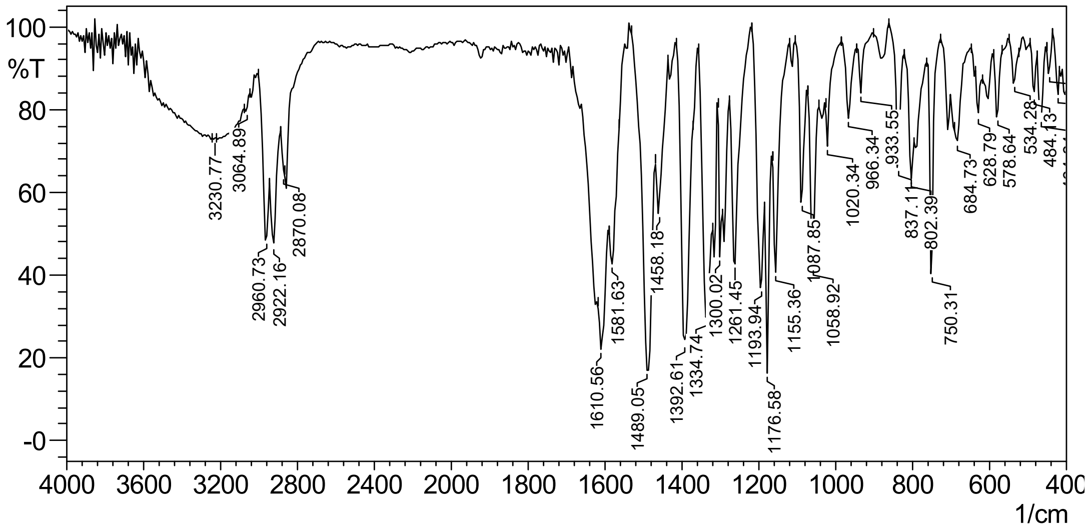

Figure S17 The IR spectra of **4b**

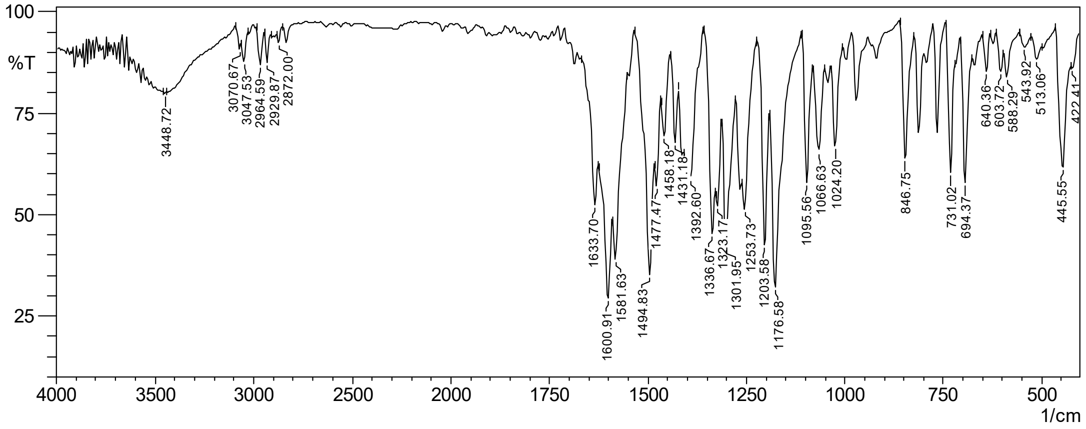

Figure S18 The IR spectra of **5a**

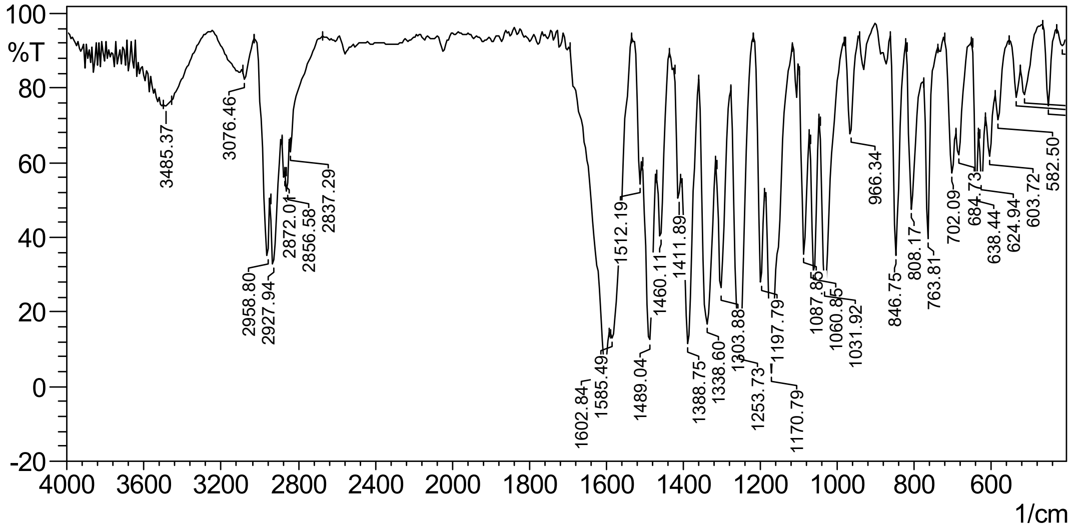

Figure S19 The IR spectra of **5b**

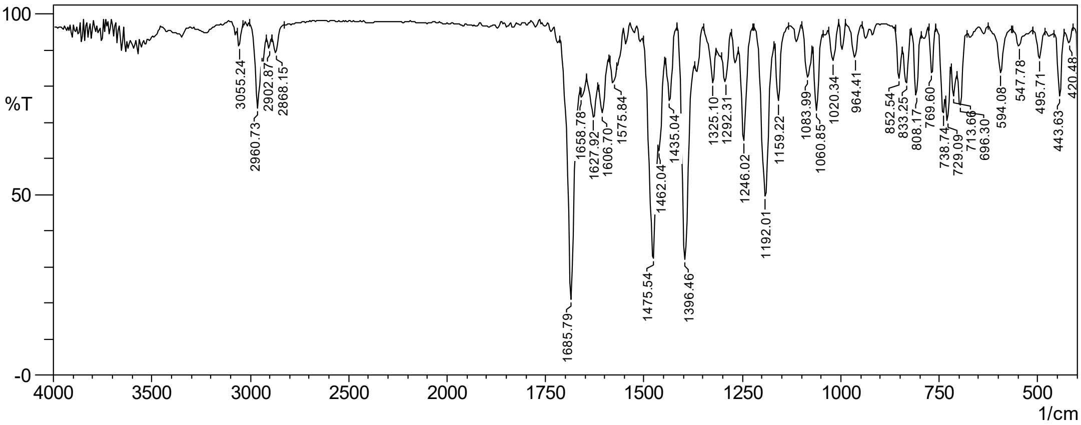

Figure S20 The IR spectra of **6a**

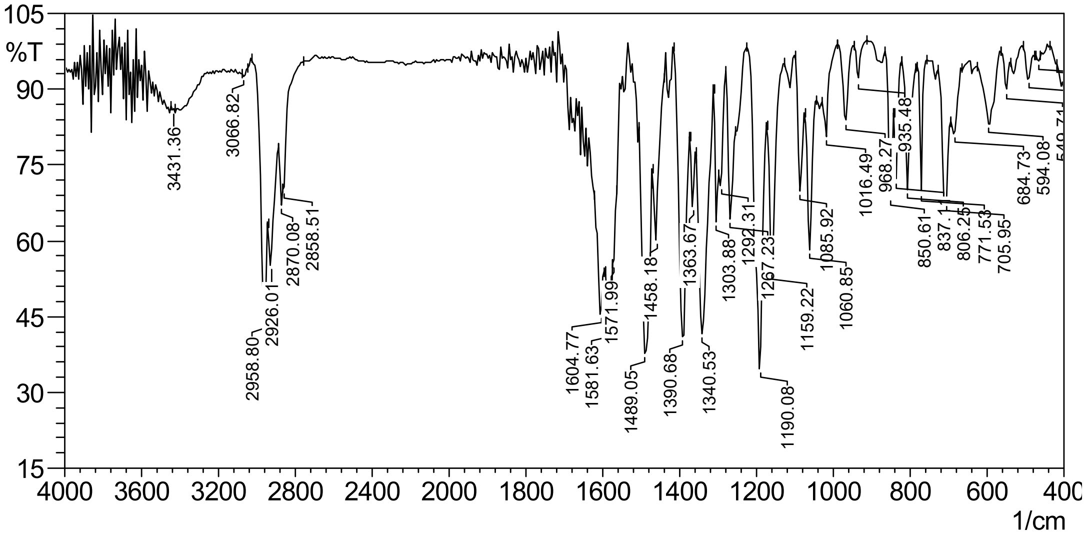

Figure S21 The IR spectra of **6b**

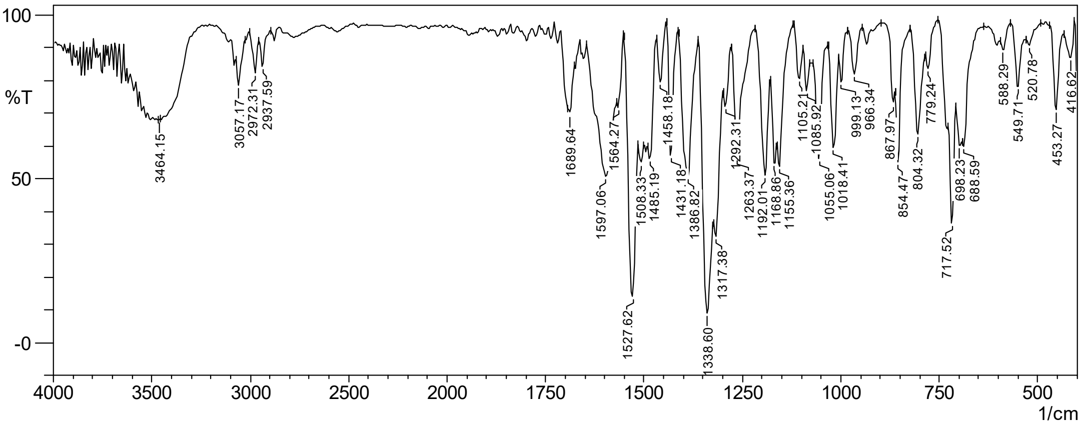

Figure S22 The IR spectra of **7a**

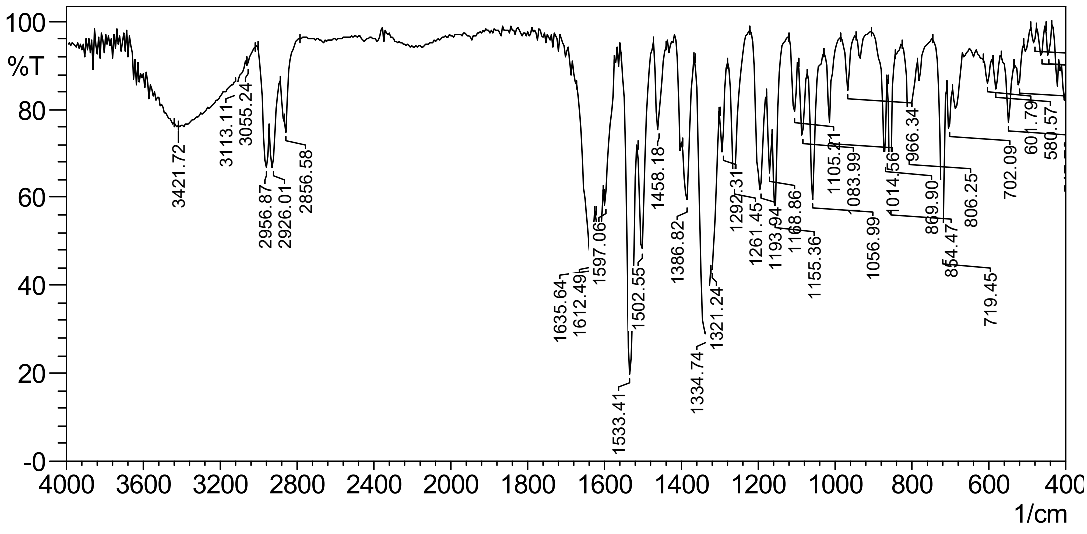

Figure S23 The IR spectra of **7b**

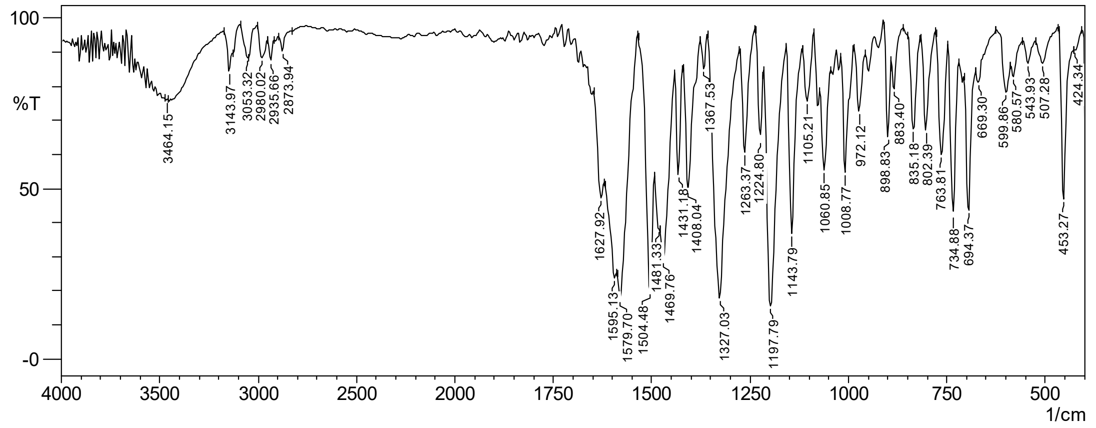

Figure S24 The IR spectra of **8a**

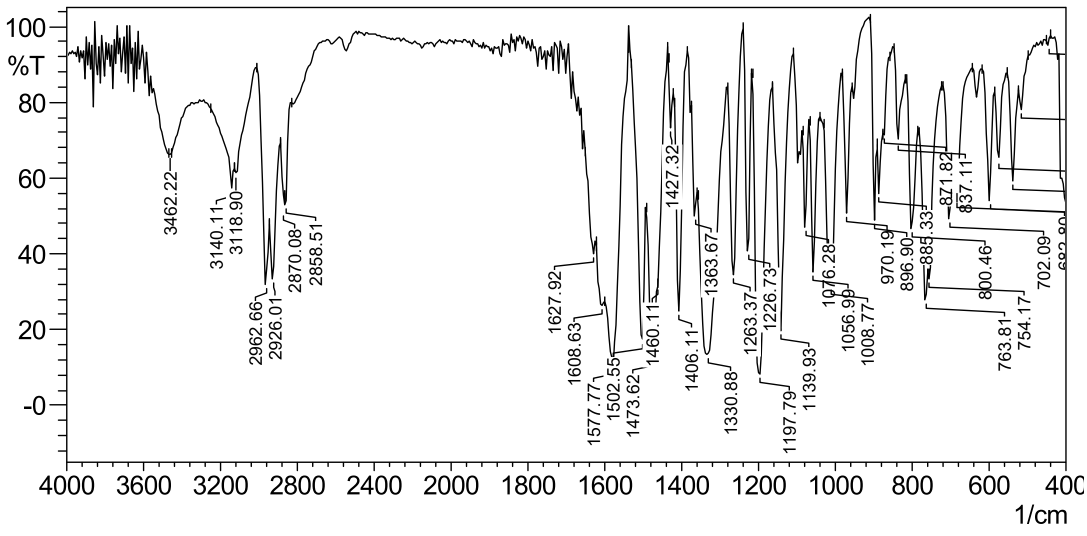

Figure S25 The IR spectra of **8b**

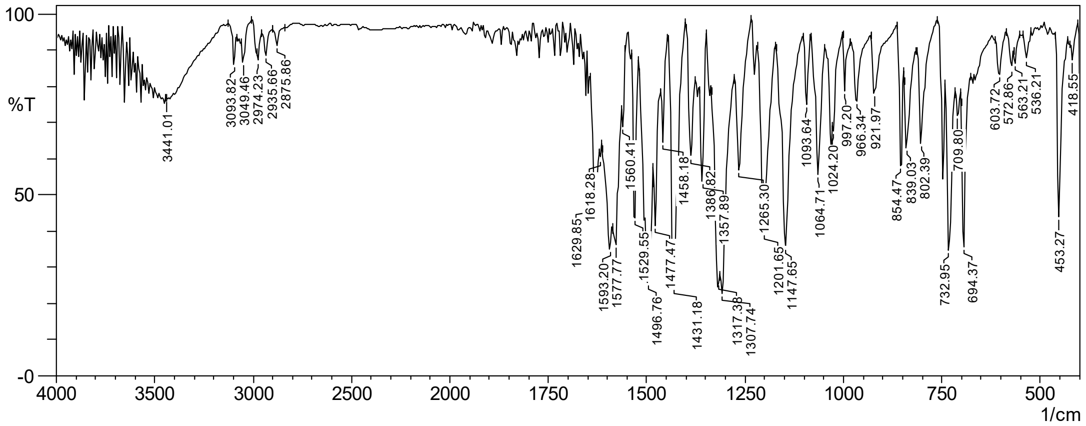

Figure S26 The IR spectra of **9a**

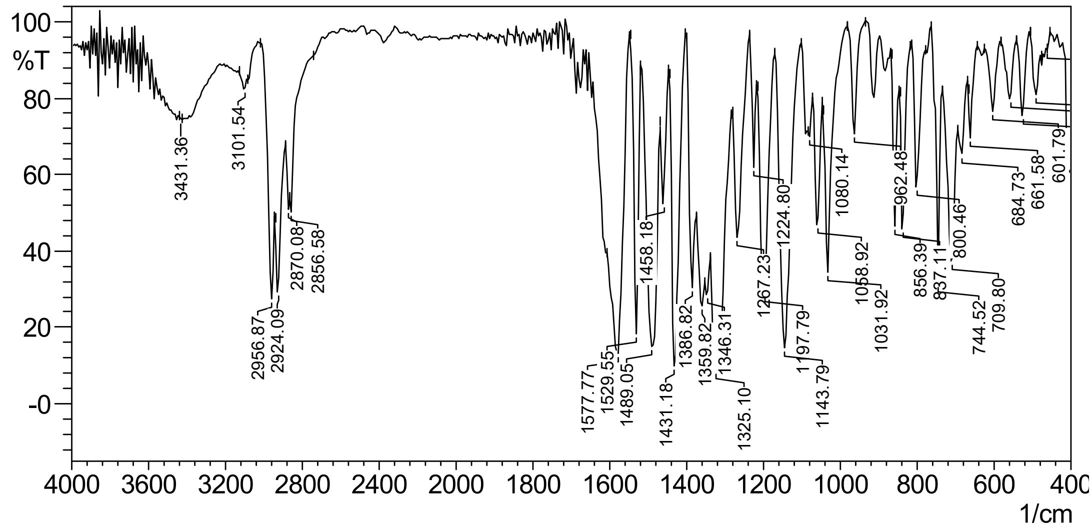

Figure S27 The IR spectra of **9b**

3.2 The NMR spectra of **1a-9b**.

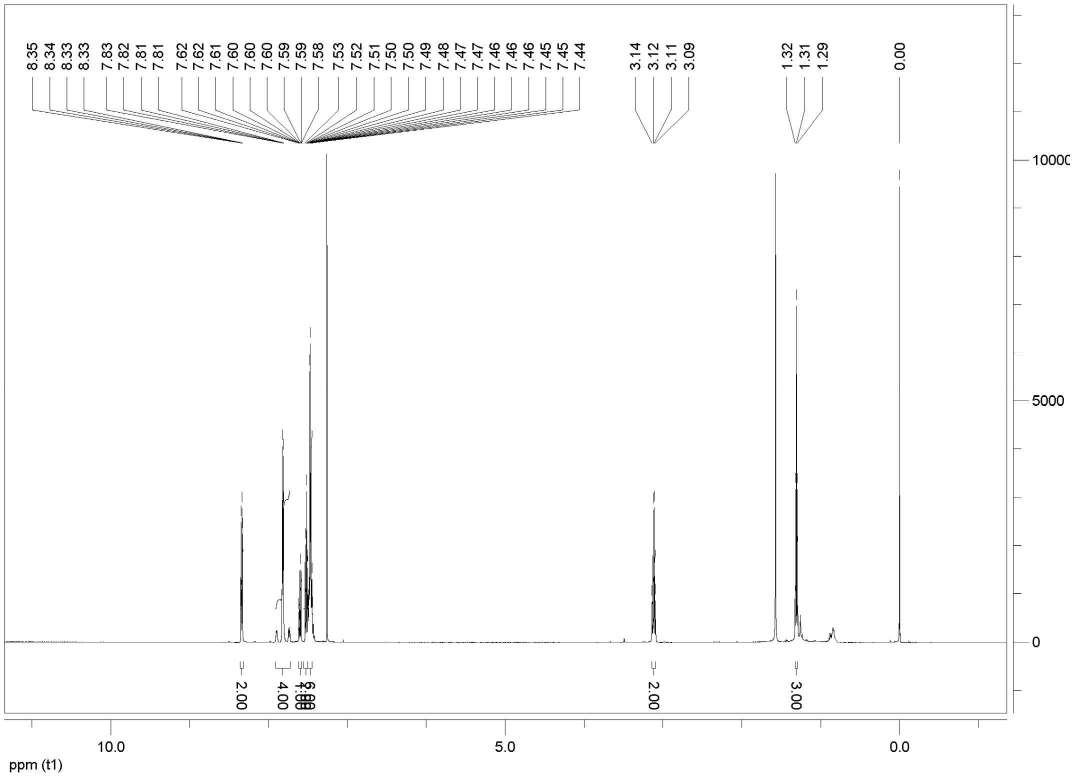

Figure S28 The ^{1}H NMR spectra of **1a**

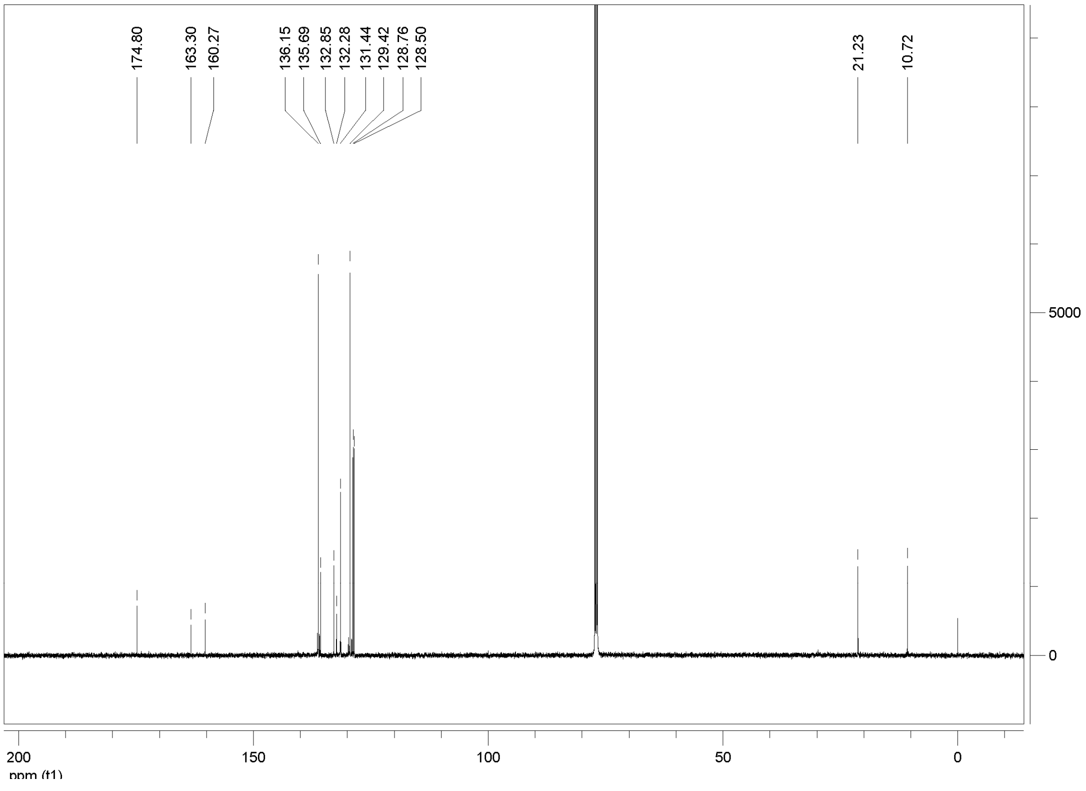

Figure S29 The ^{13}C NMR spectra of **1a**

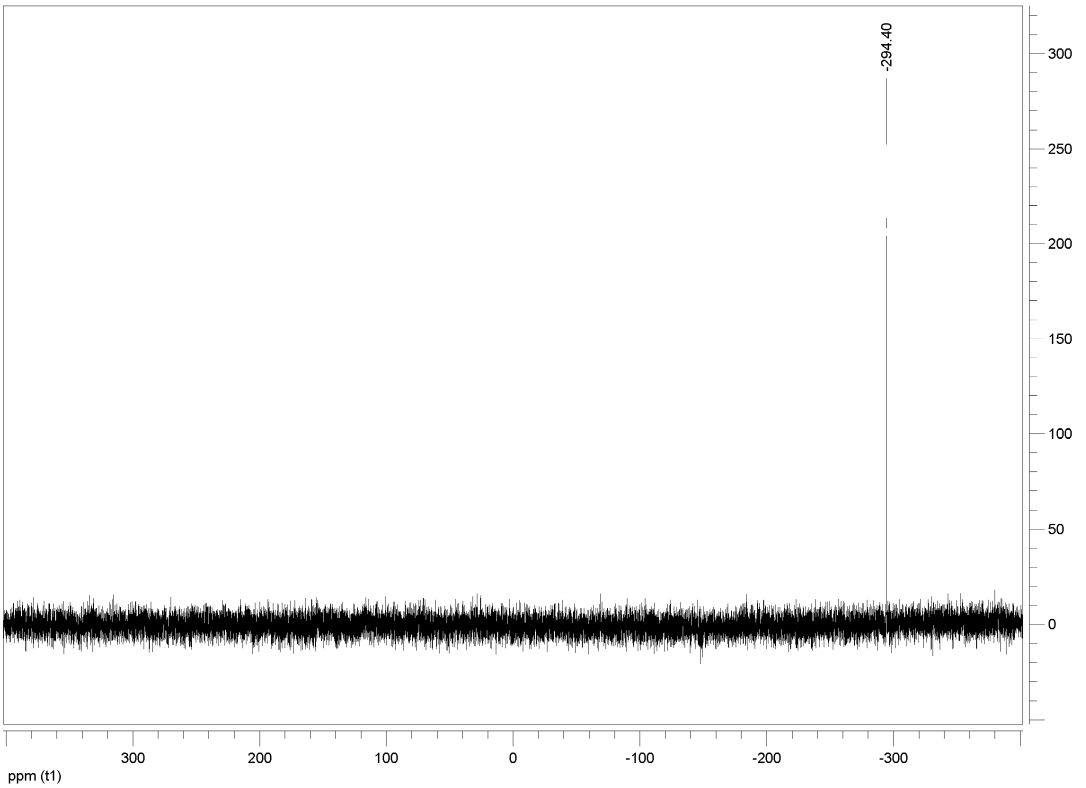

Figure S30 The ^{119}Sn NMR spectra of **1a**

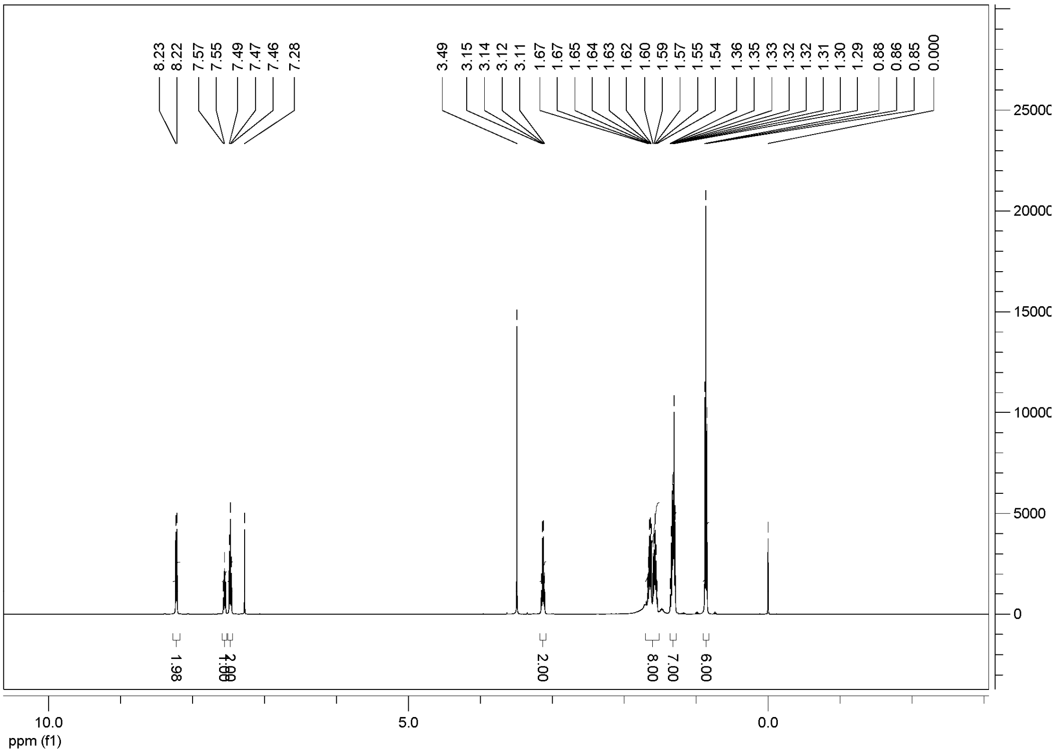

Figure S31 The ^{1}H NMR spectra of **1b**

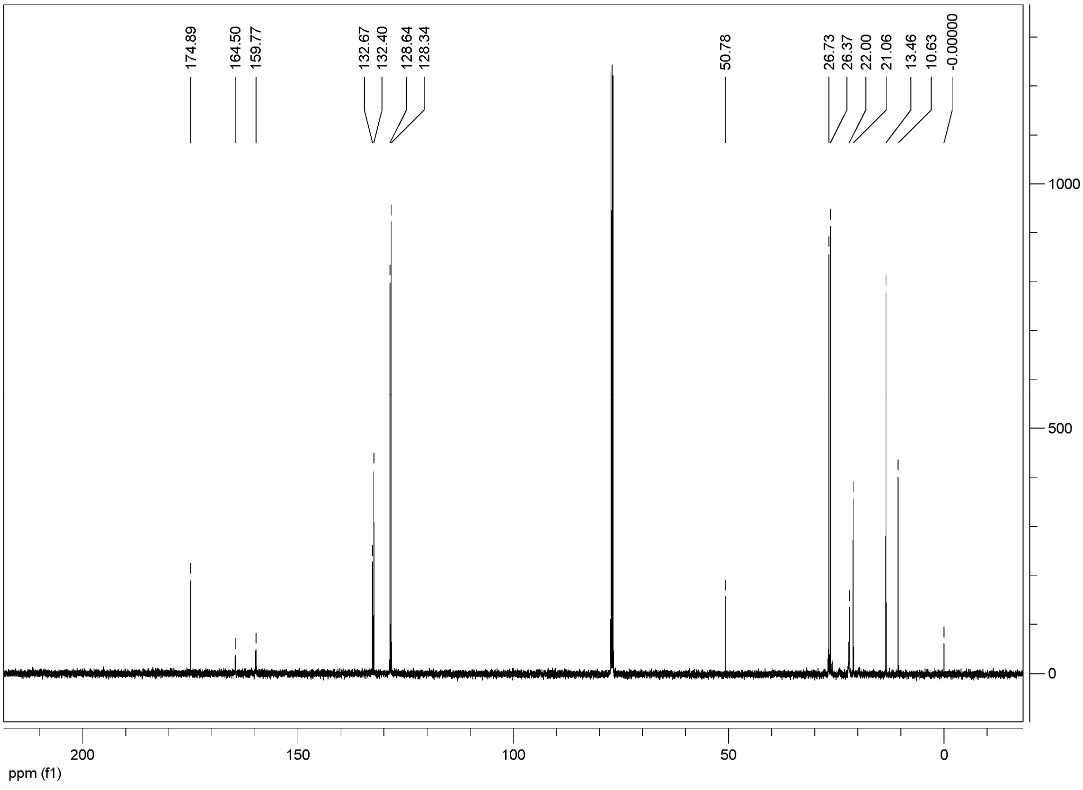

Figure S32 The ^{13}C NMR spectra of **1b**

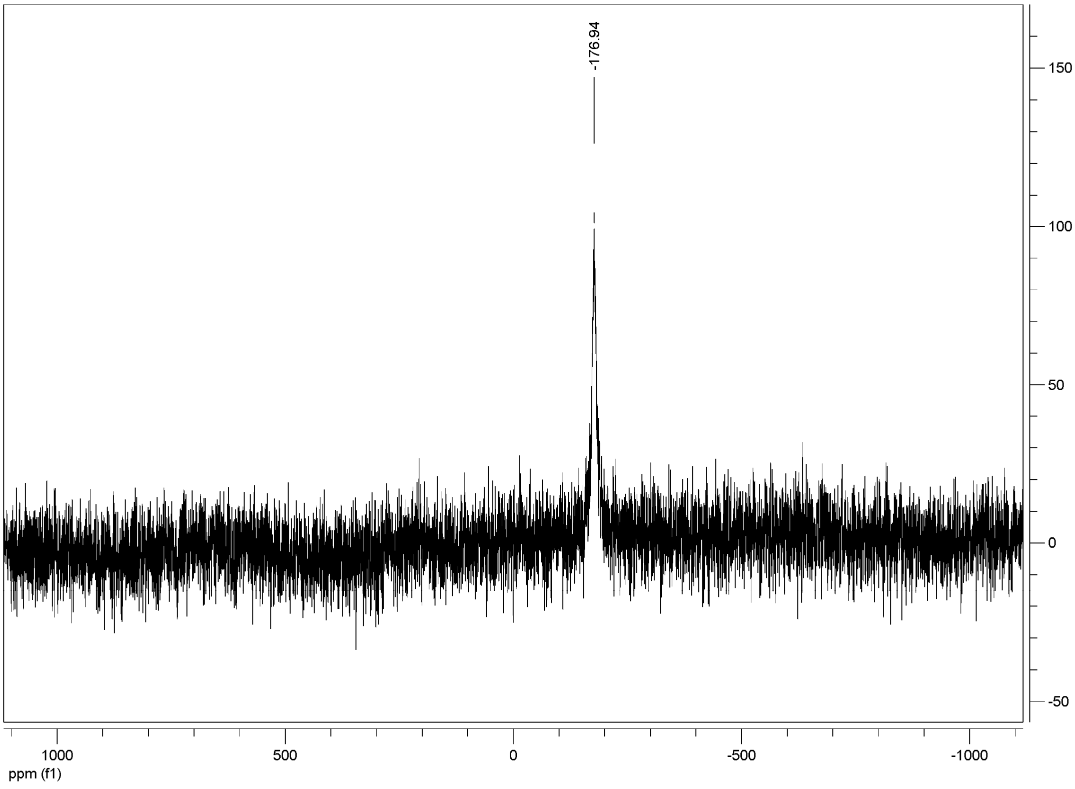

Figure S33 The ^{119}Sn NMR spectra of **1b**

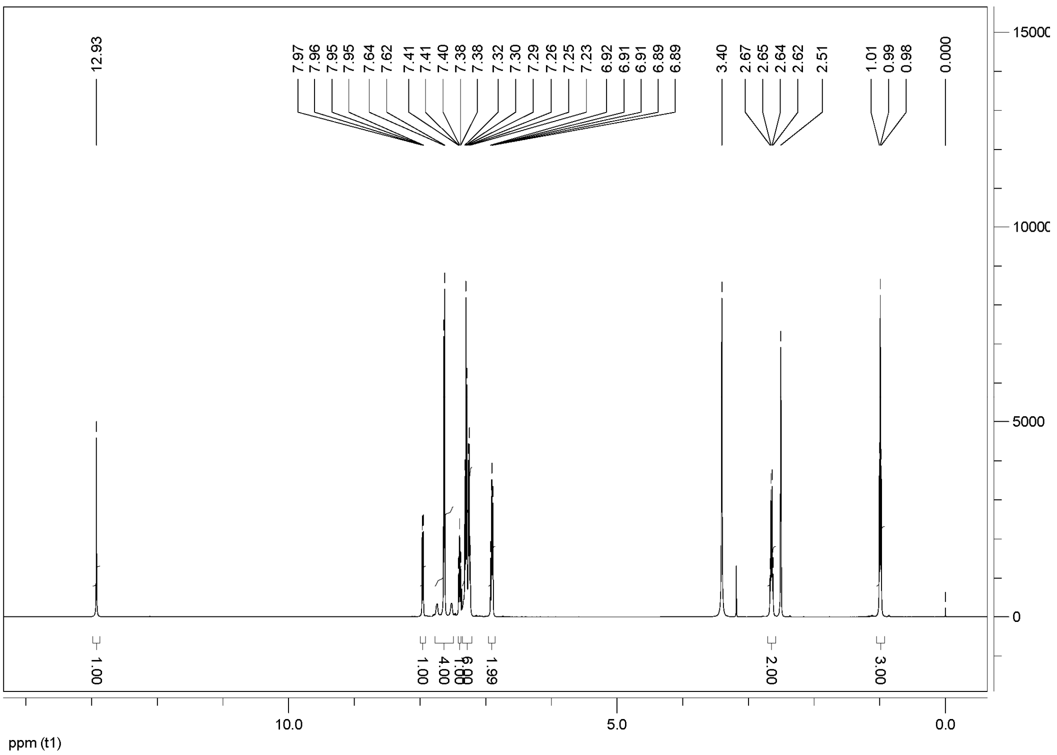

Figure S34 The ^{1}H NMR spectra of **2a**

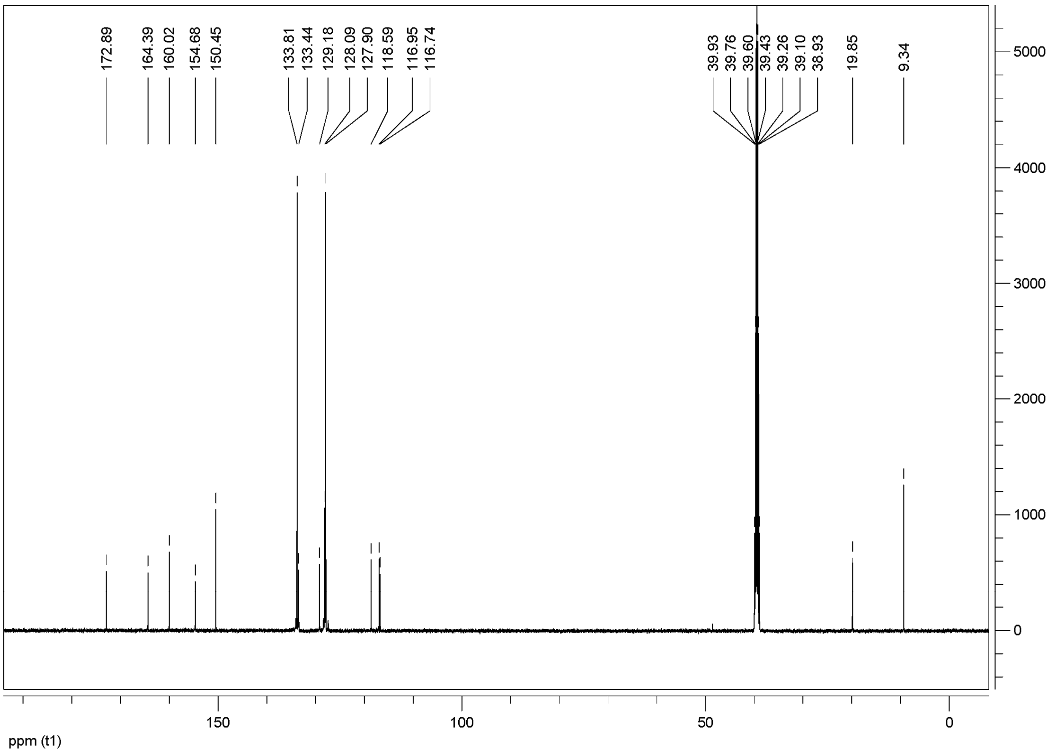

Figure S35 The ^{13}C NMR spectra of **2a**

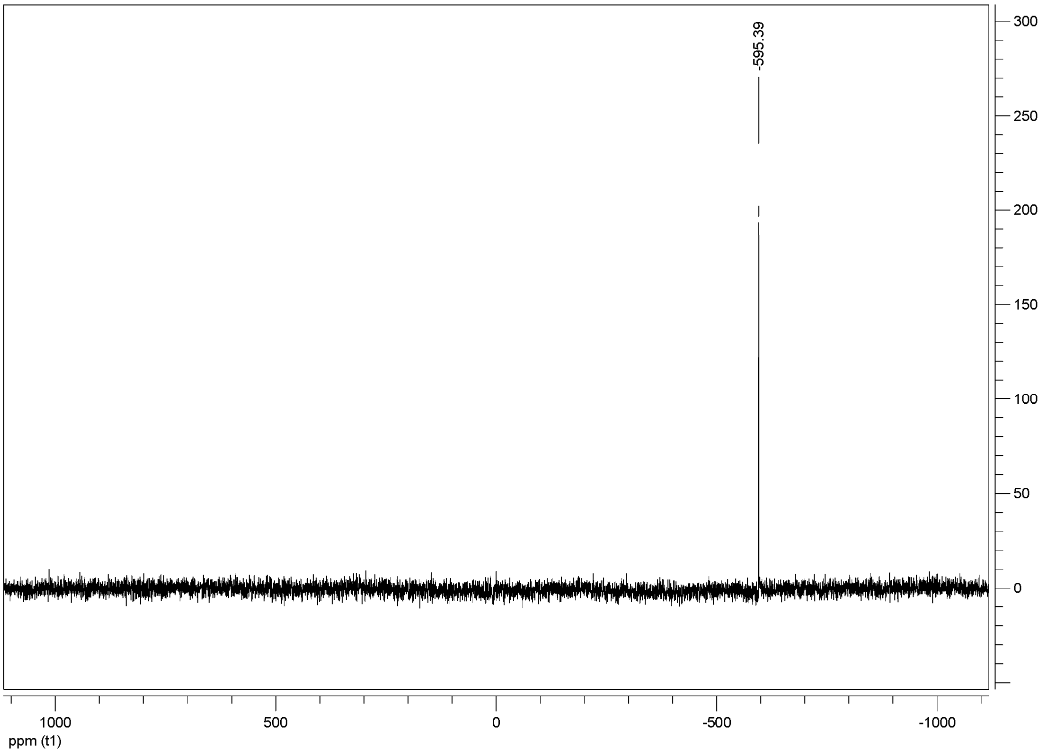

Figure S36 The ^{119}Sn NMR spectra of **2a**

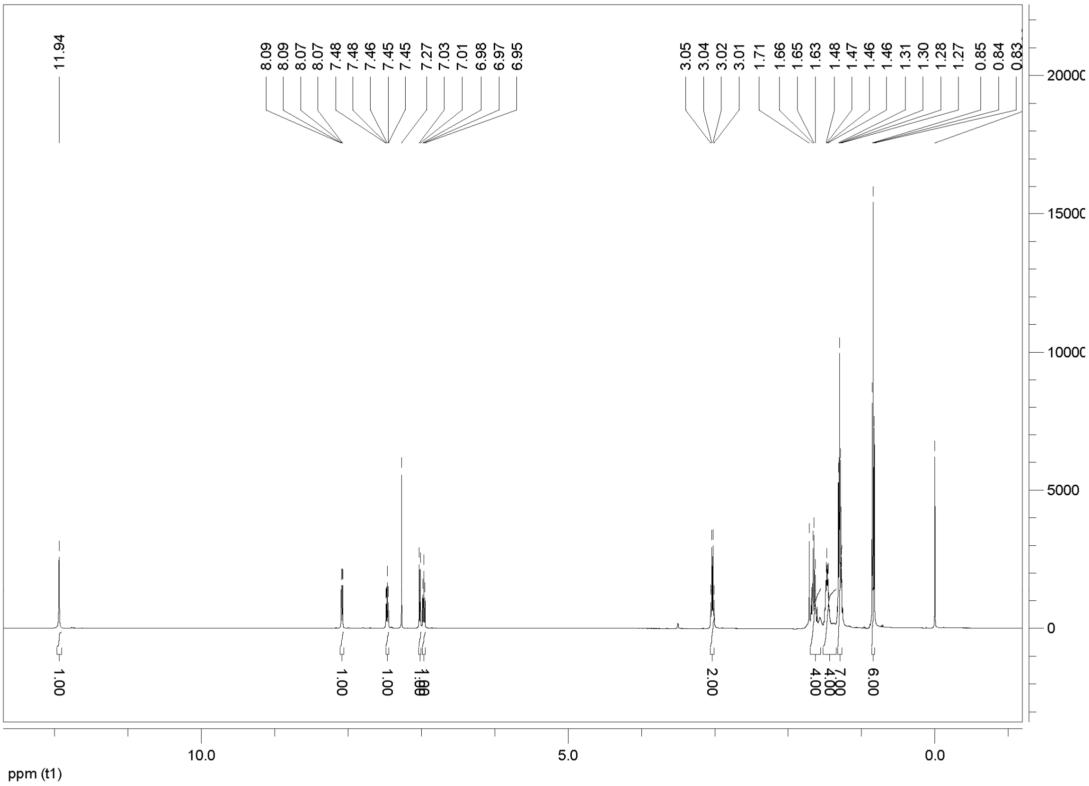

Figure S37 The ^{1}H NMR spectra of **2b**

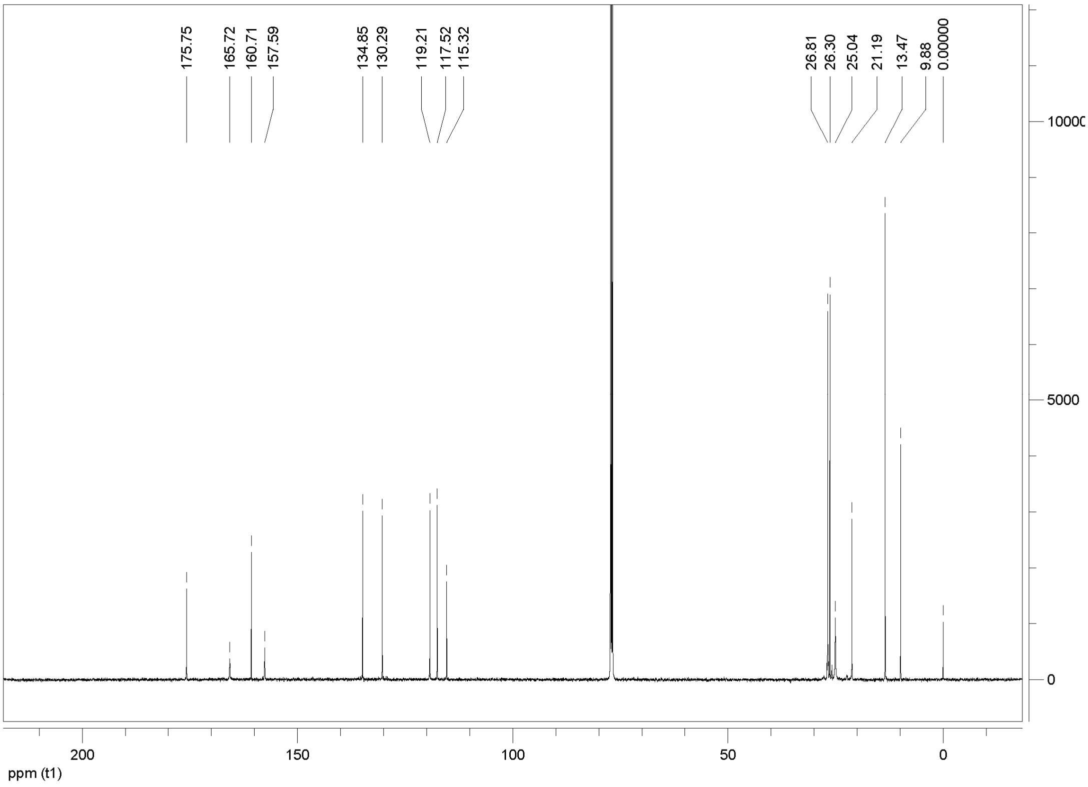

Figure S38 The ^{13}C NMR spectra of **2b**

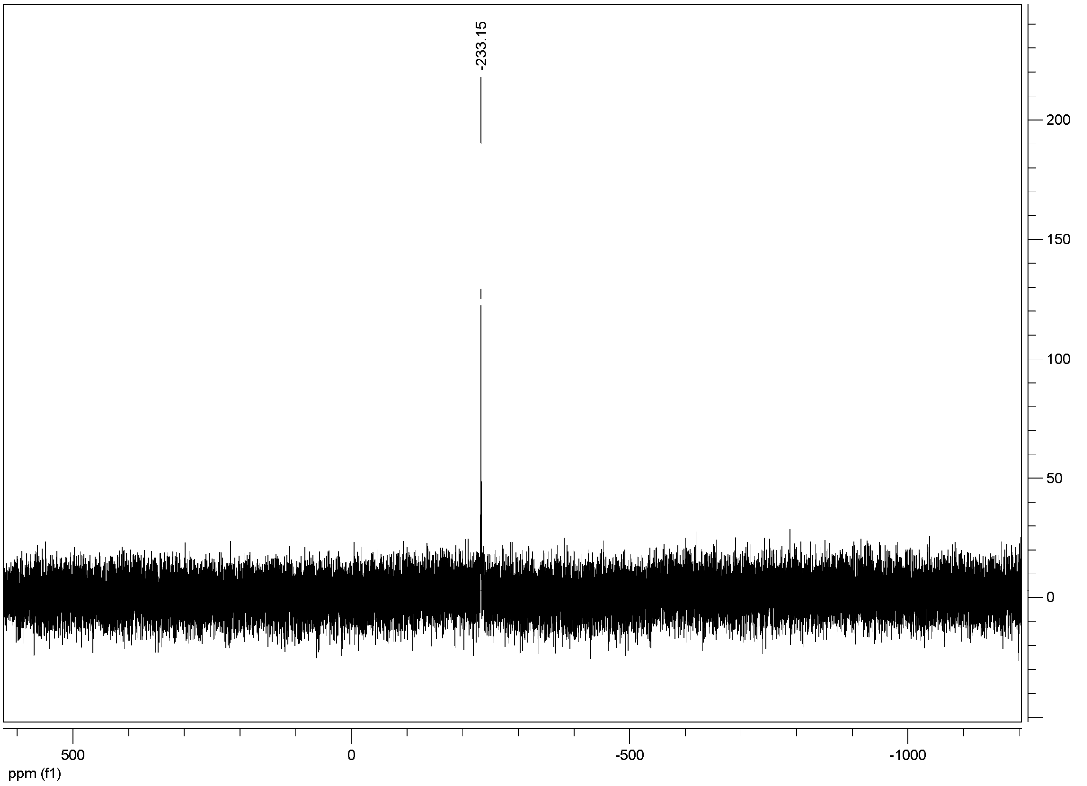

Figure S39 The ^{119}Sn NMR spectra of **2b**

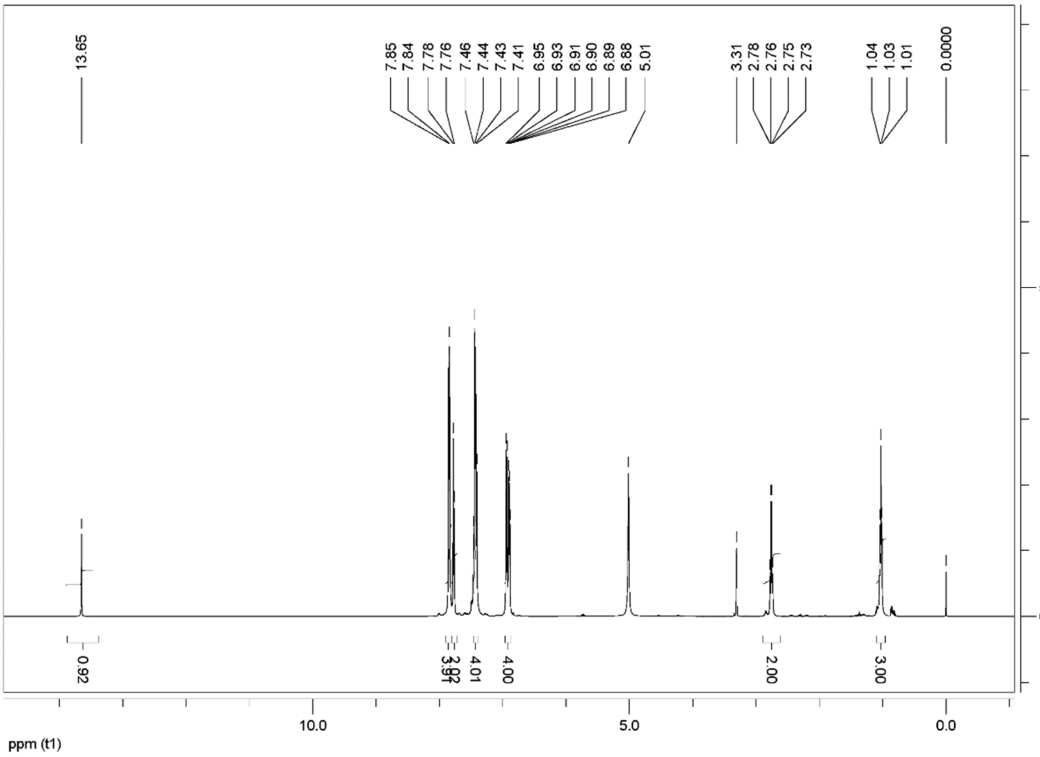

Figure S40 The ^{1}H NMR spectra of **3a**

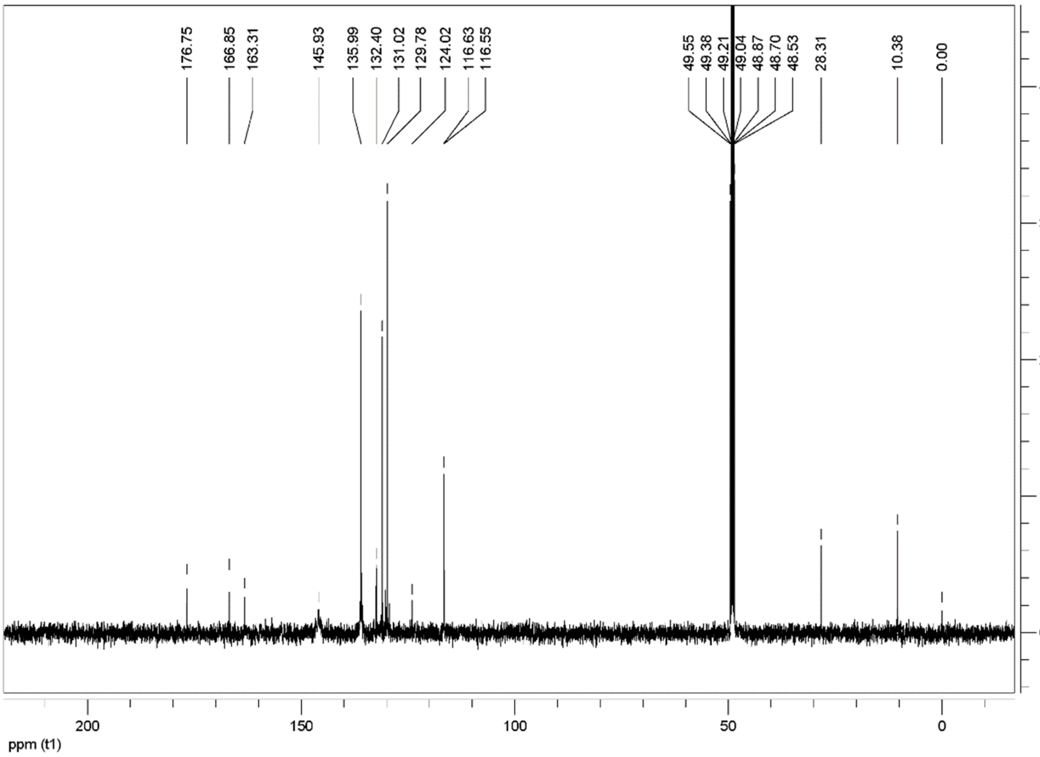

Figure S41 The ^{13}C NMR spectra of **3a**

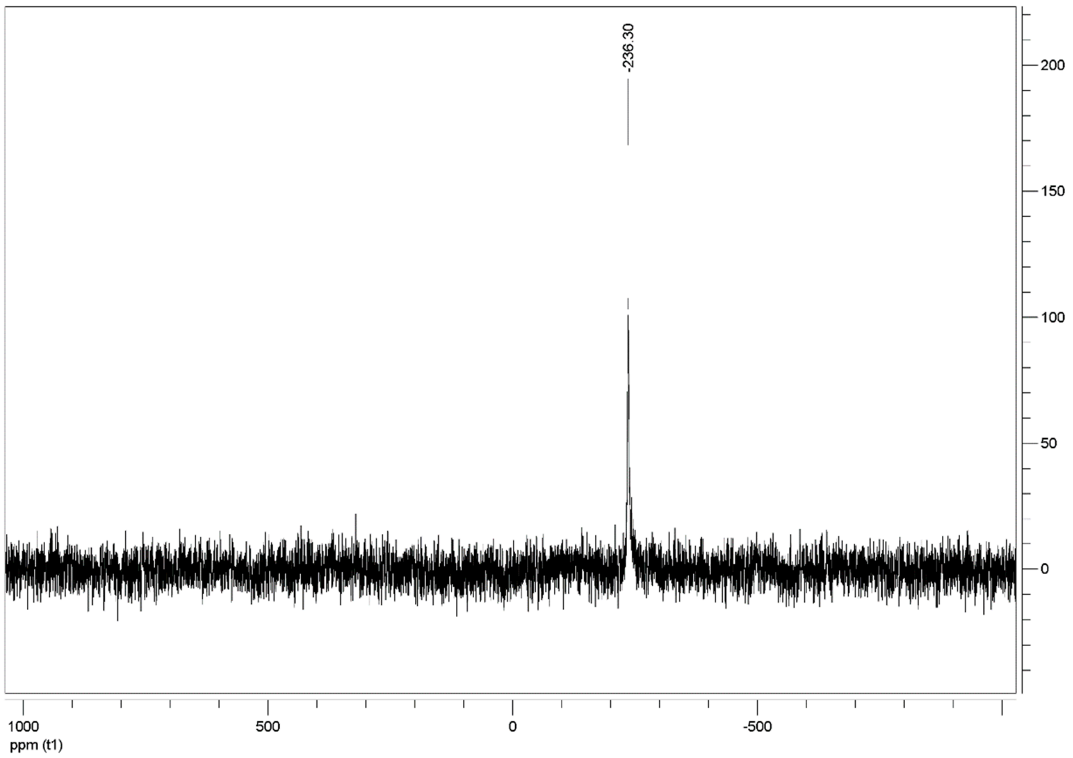

Figure S42 The ^{119}Sn NMR spectra of **3a**

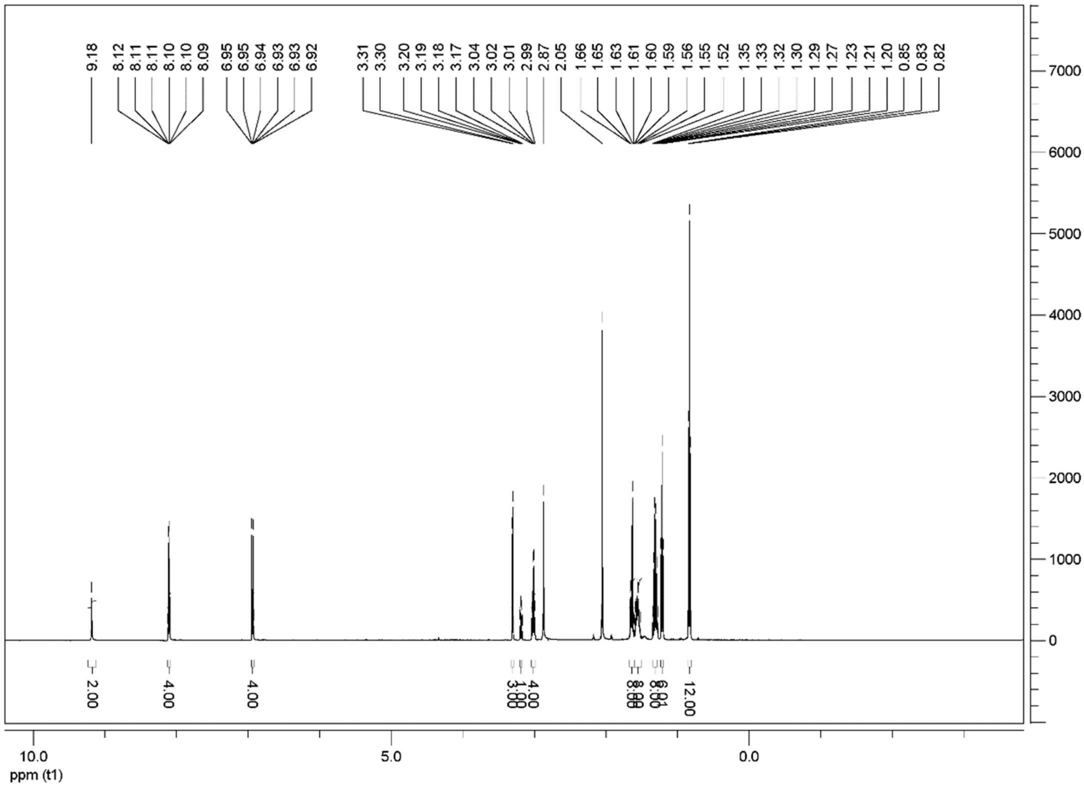

Figure S43 The ^{1}H NMR spectra of **3b**

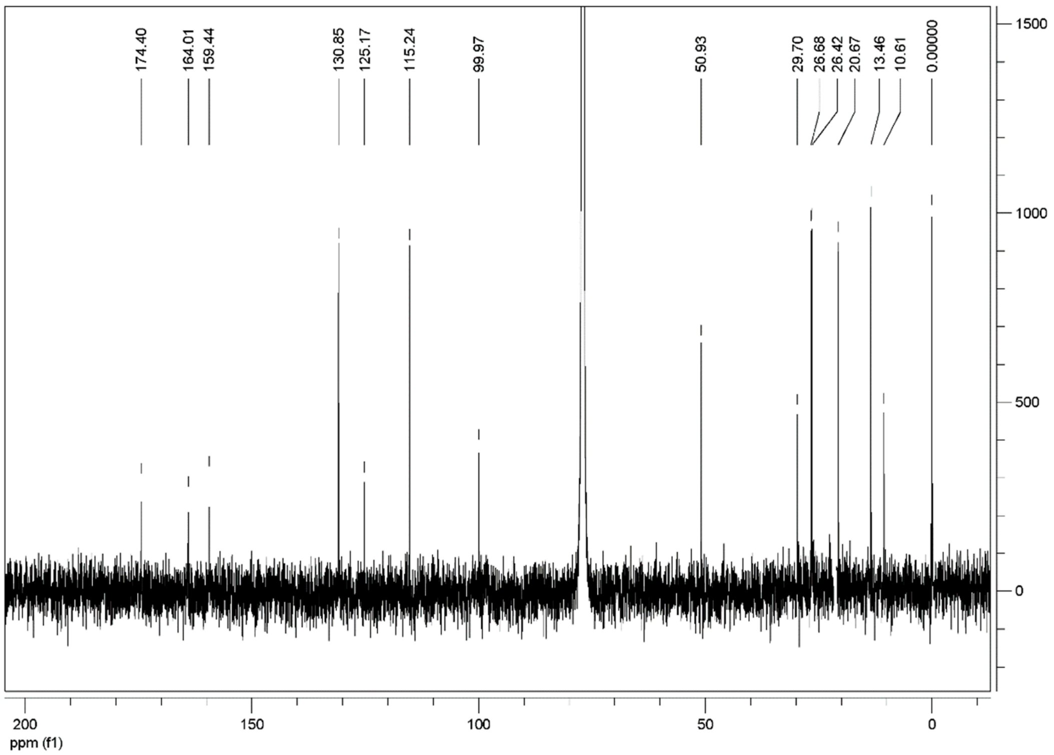

Figure S44 The ^{13}C NMR spectra of **3b**

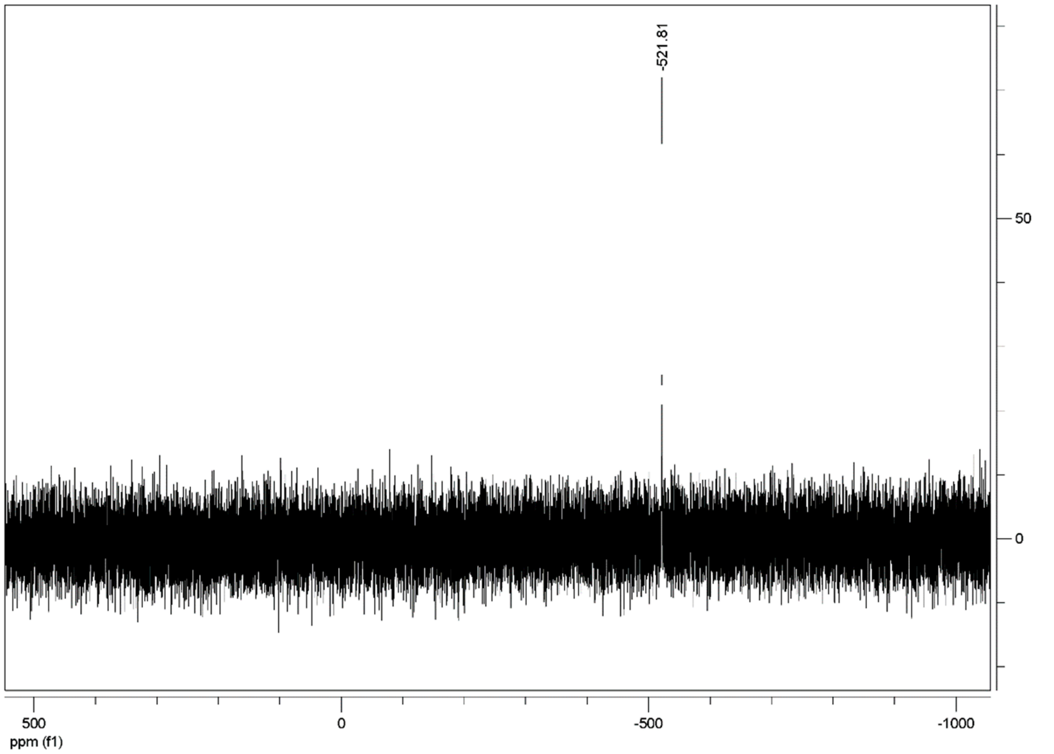

Figure S45 The ^{119}Sn NMR spectra of **3b**

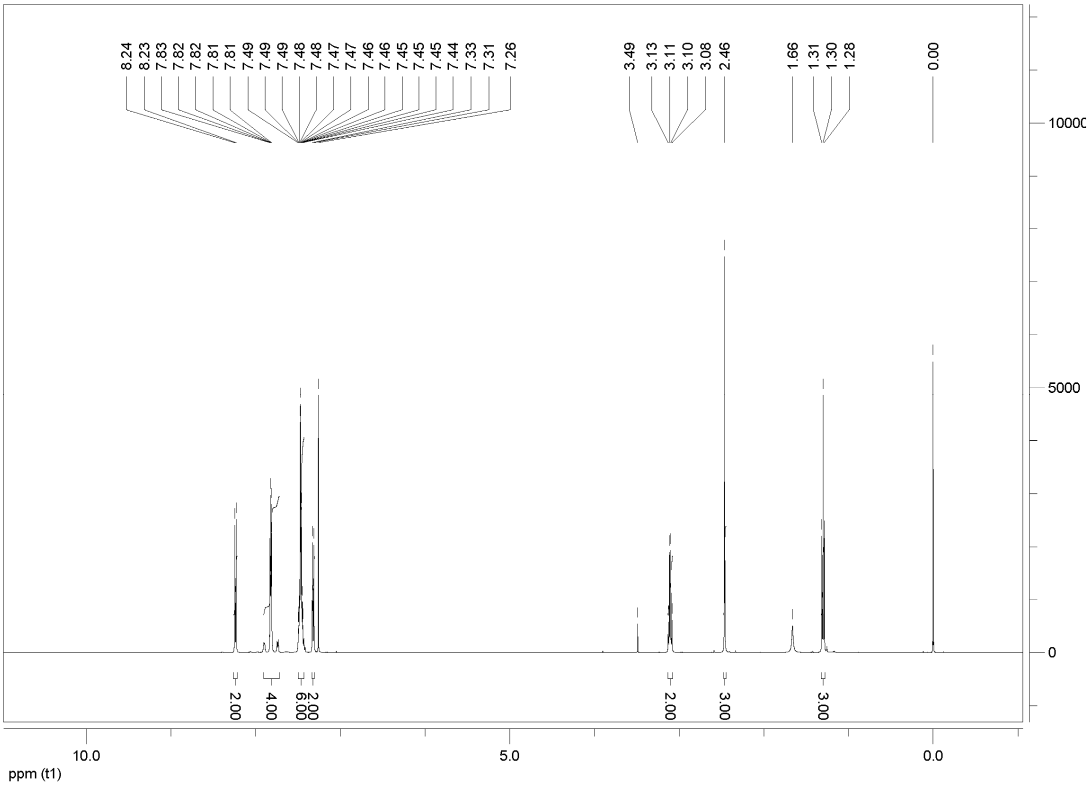

Figure S46 The ^{1}H NMR spectra of **4a**

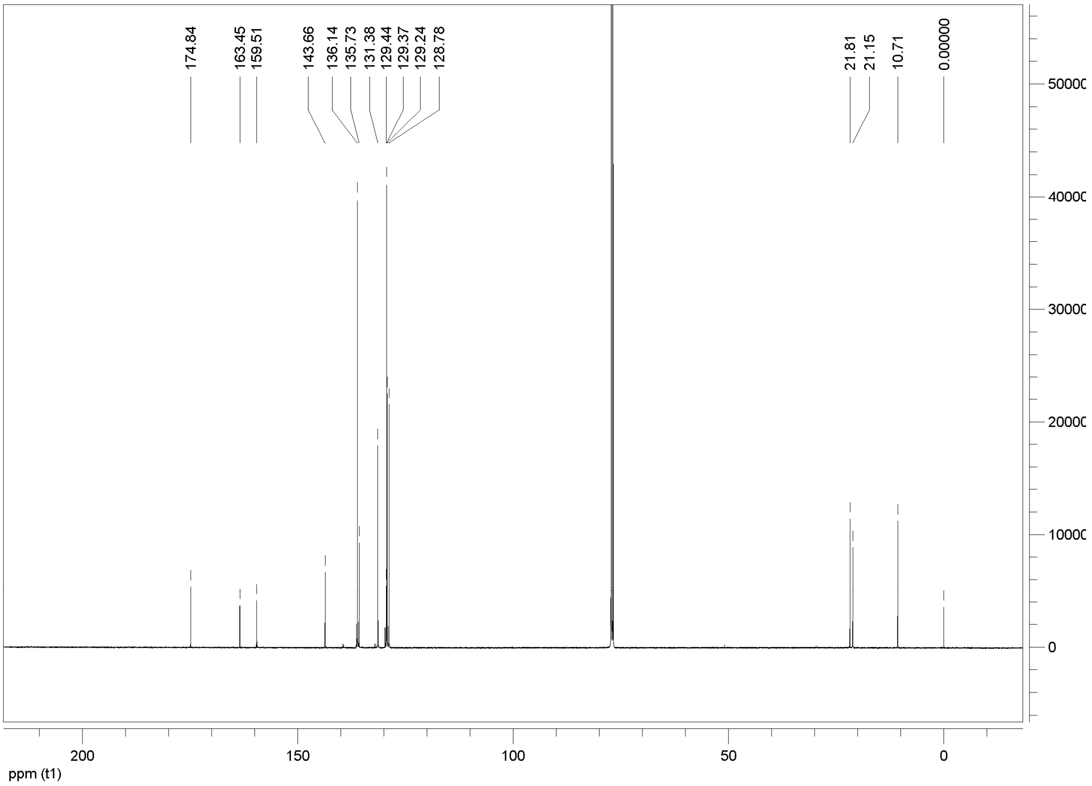

Figure S47 The ^{13}C NMR spectra of **4a**

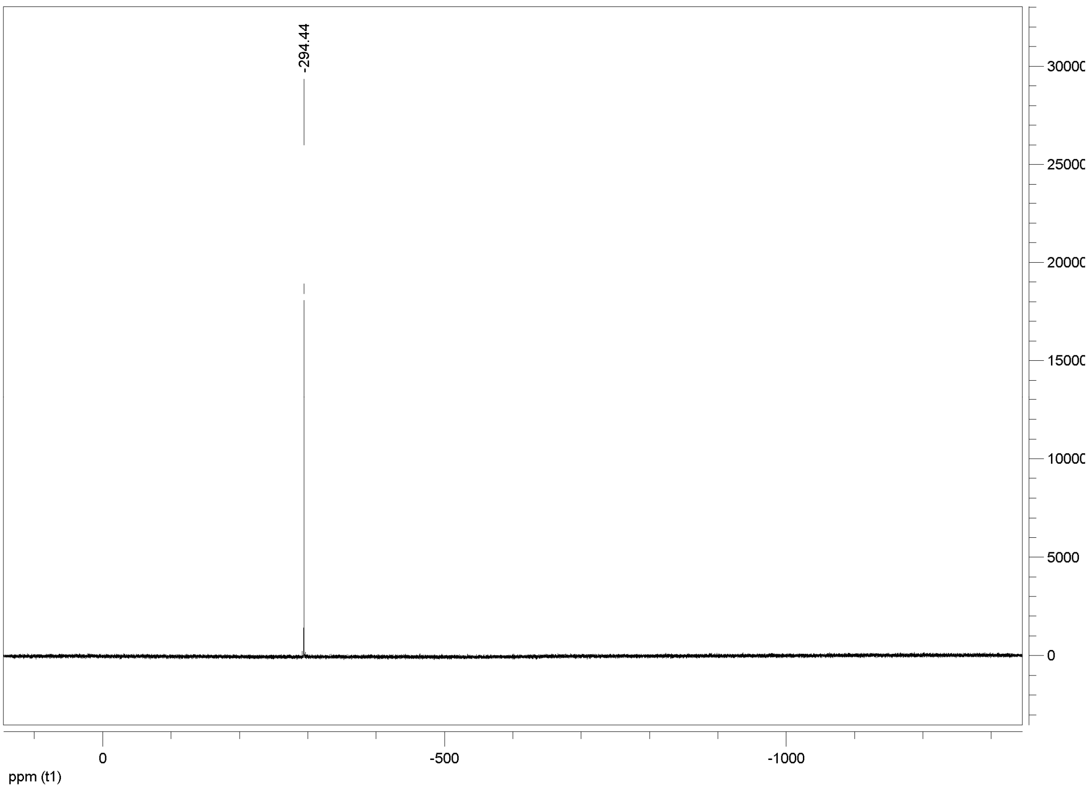

Figure S48 The ^{119}Sn NMR spectra of **4a**

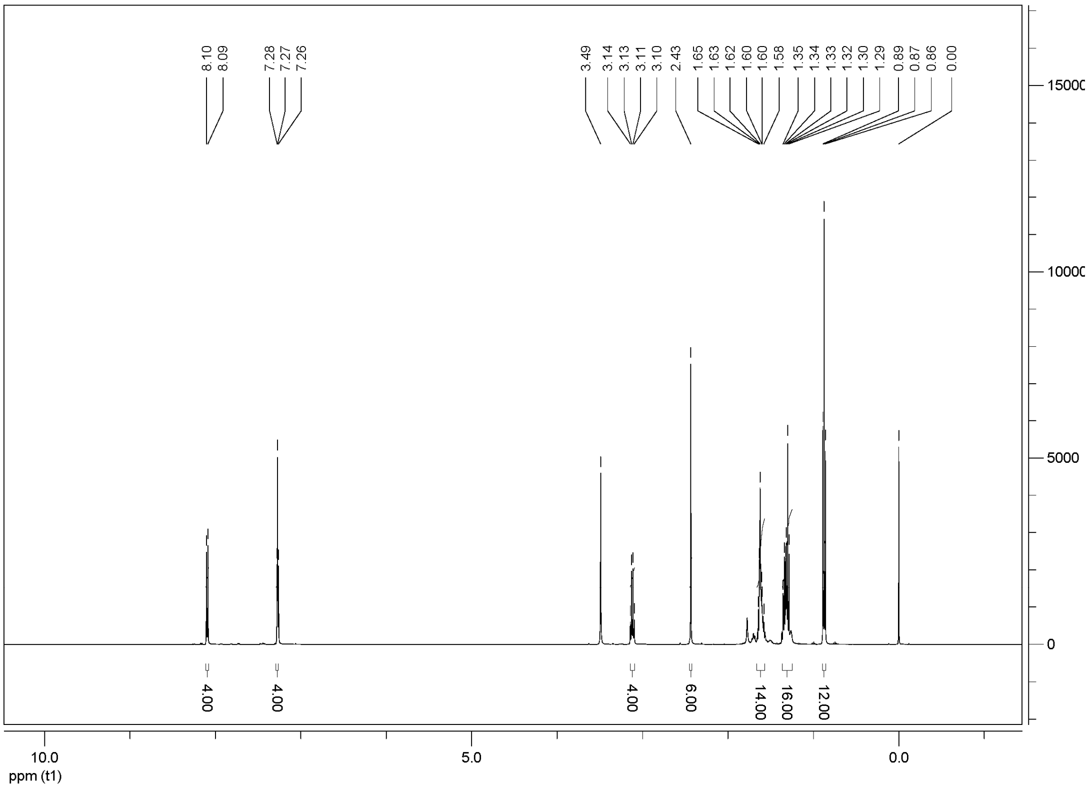

Figure S49 The ^{1}H NMR spectra of **4b**

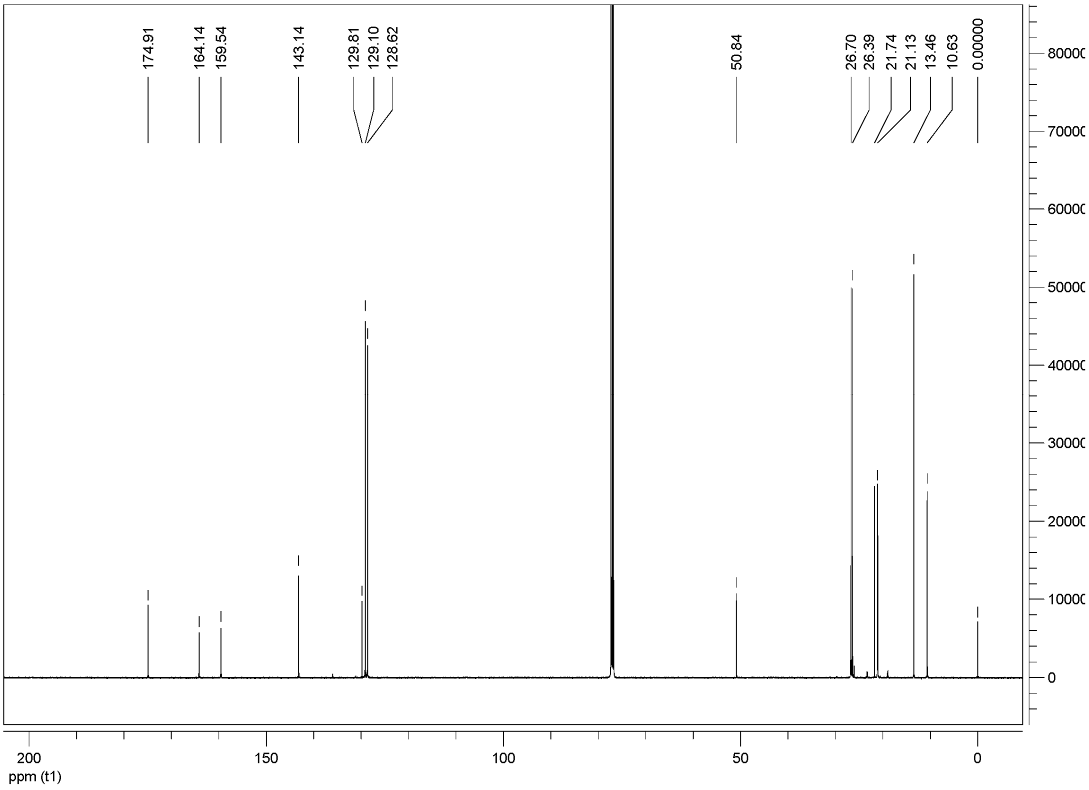

Figure S50 The ^{13}C NMR spectra of **4b**

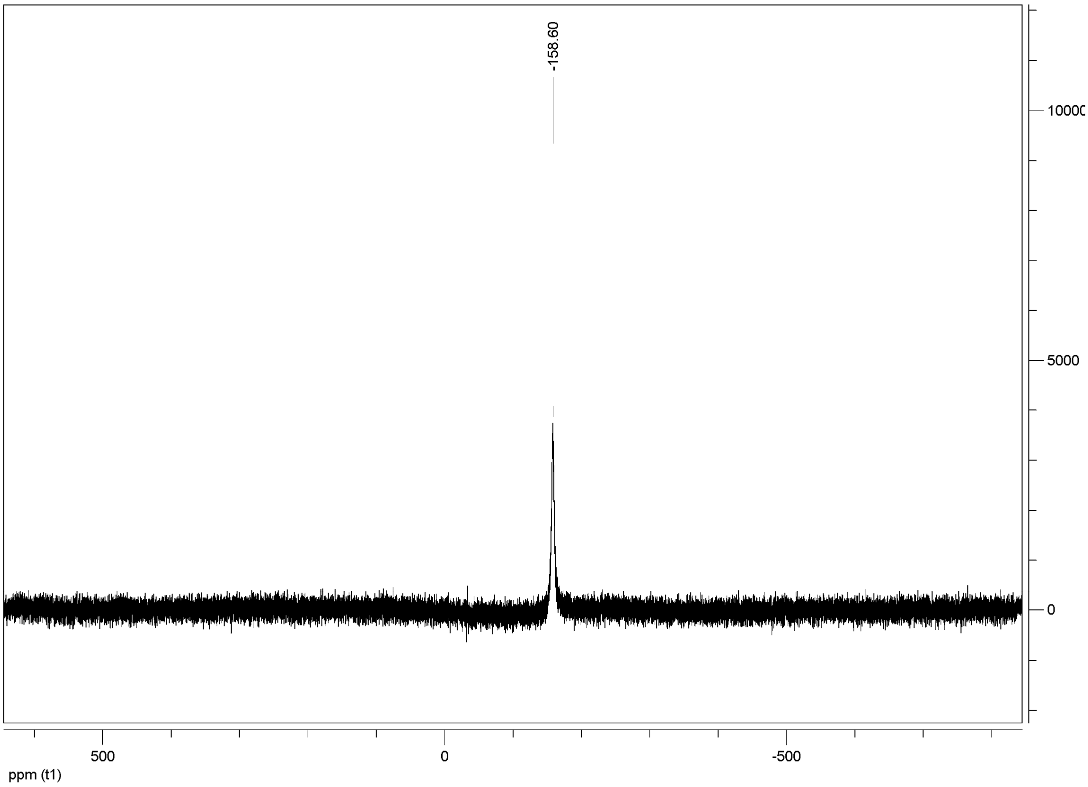

Figure S51 The ^{119}Sn NMR spectra of **4b**

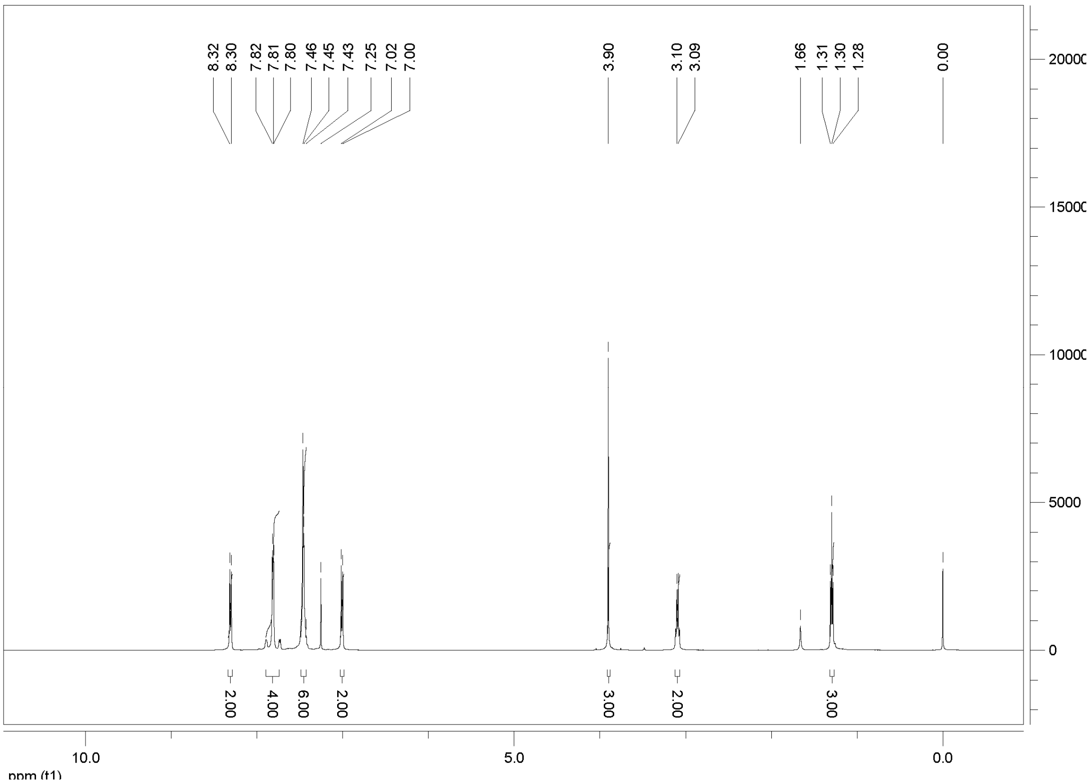

Figure S52 The ^{1}H NMR spectra of **5a**

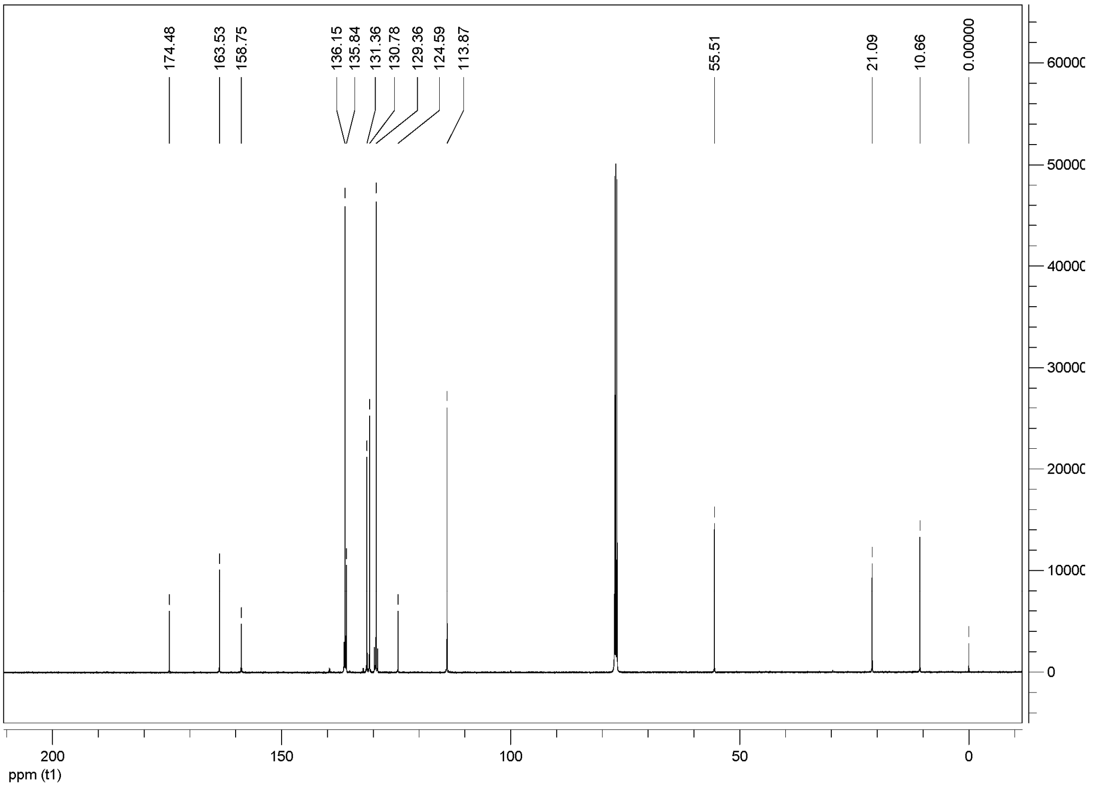

Figure S53 The ^{13}C NMR spectra of **5a**

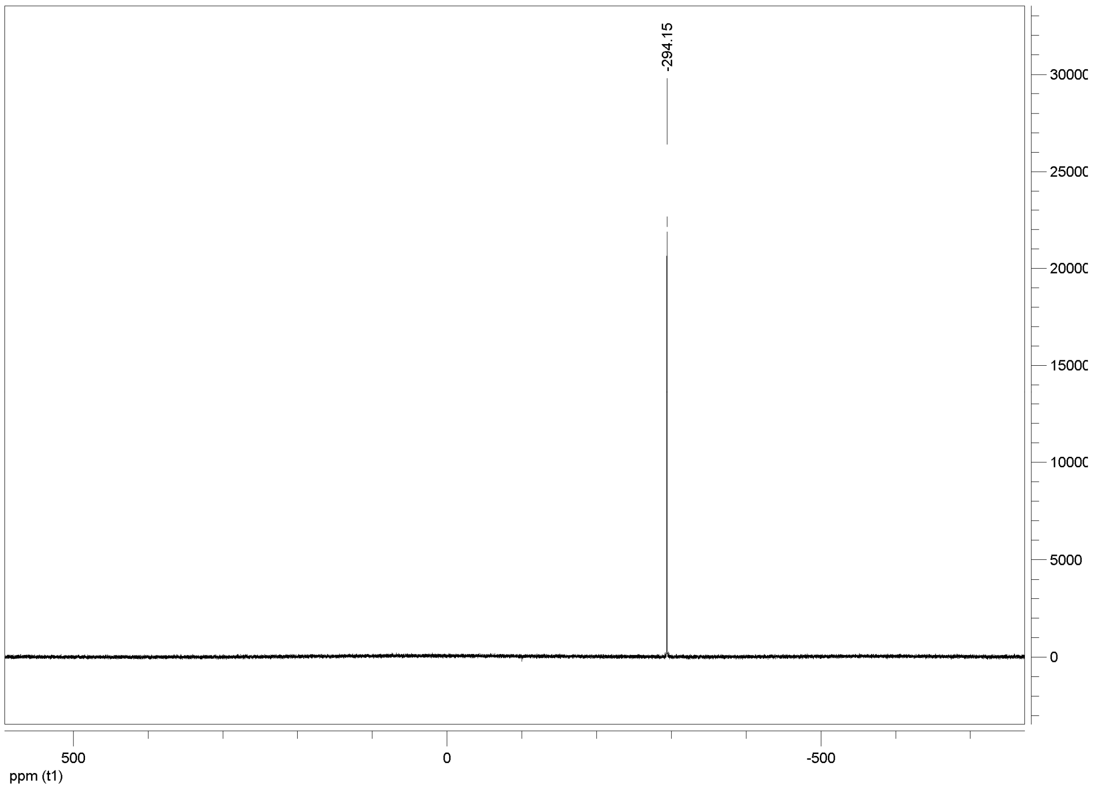

Figure S54 The ^{119}Sn NMR spectra of **5a**

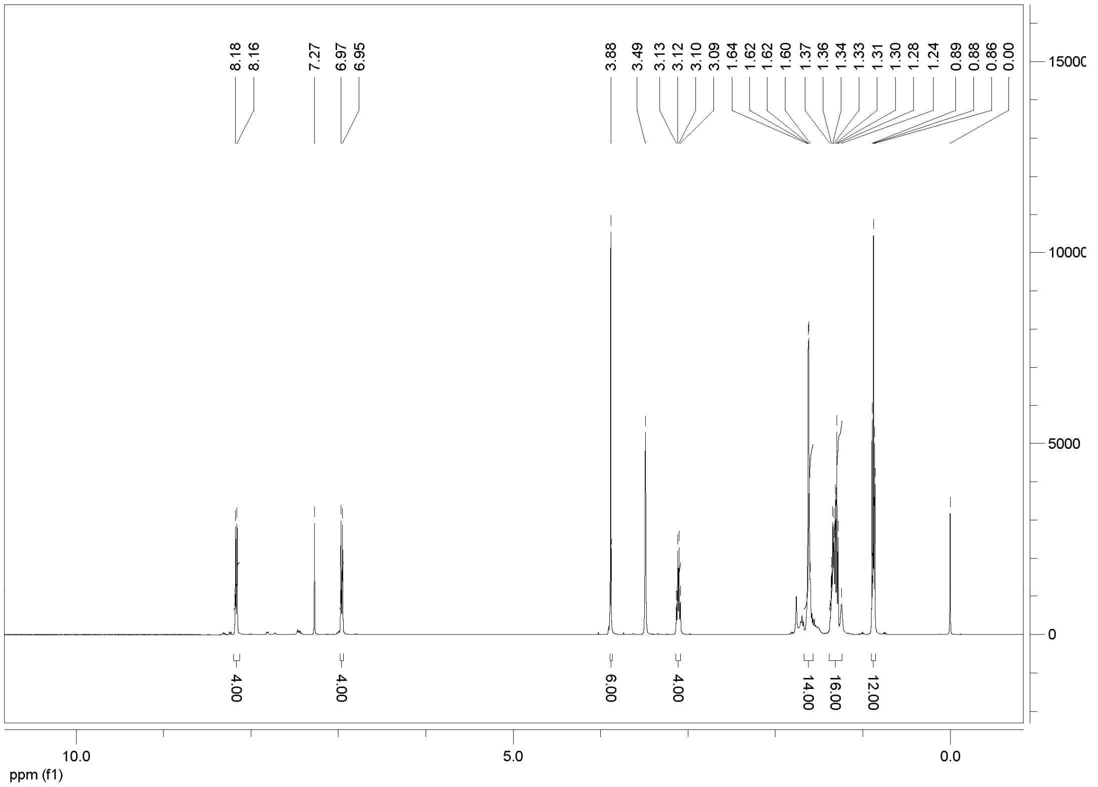

Figure S55 The ^{1}H NMR spectra of **5b**

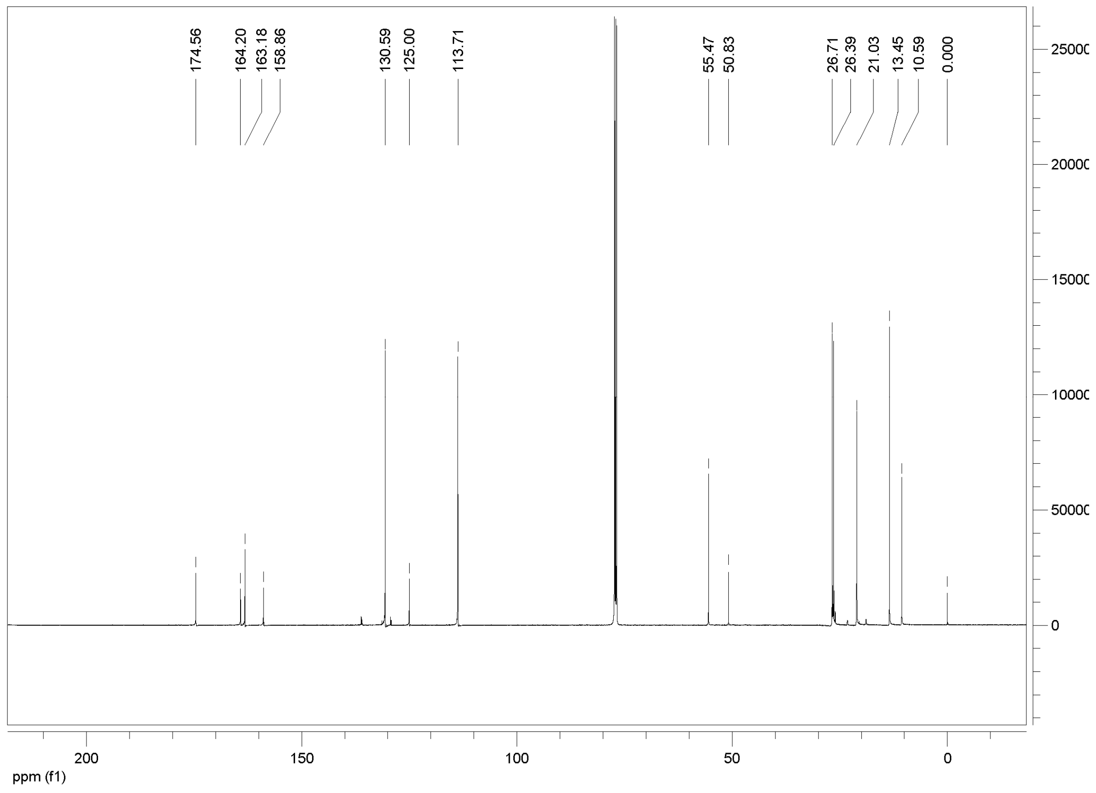

Figure S56 The ^{13}C NMR spectra of **5b**

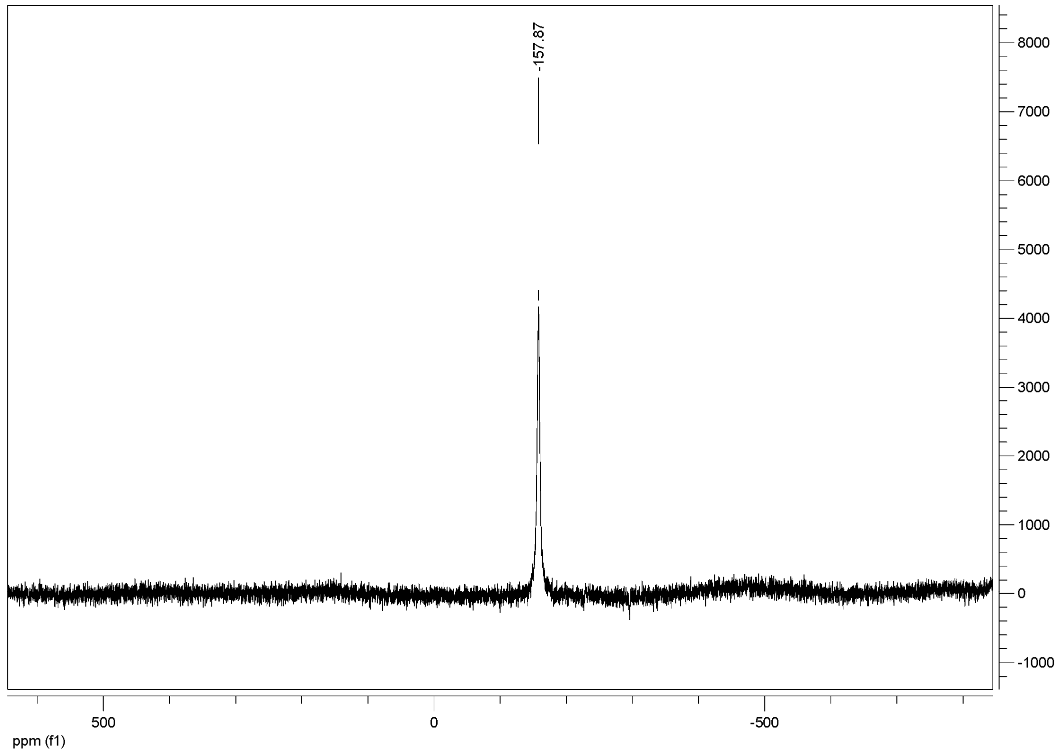

Figure S57 The ^{119}Sn NMR spectra of **5b**

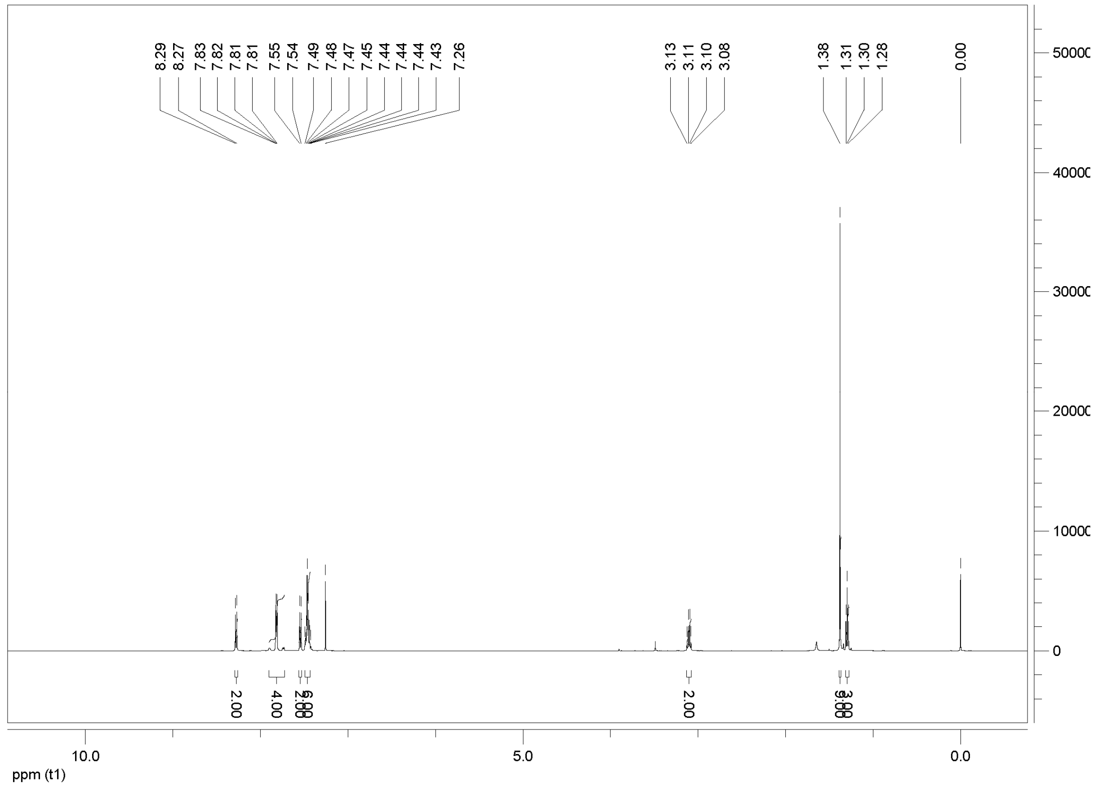

Figure S58 The ^{1}H NMR spectra of **6a**

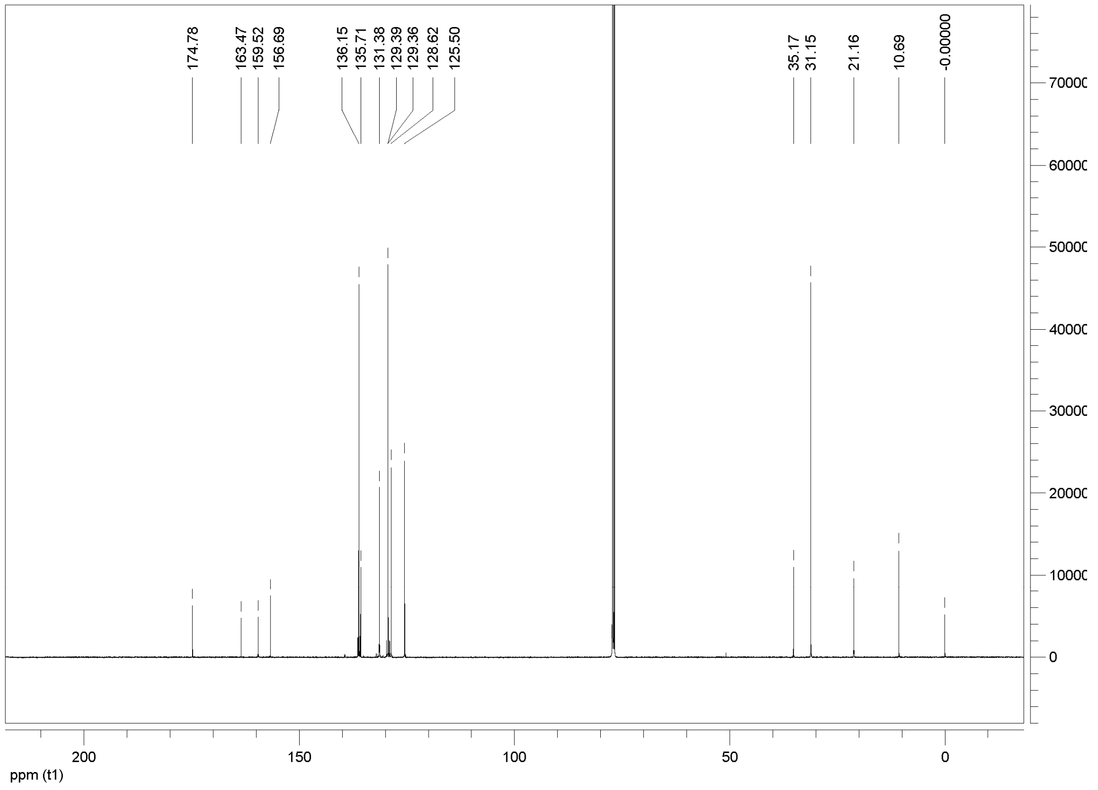

Figure S59 The ^{13}C NMR spectra of **6a**

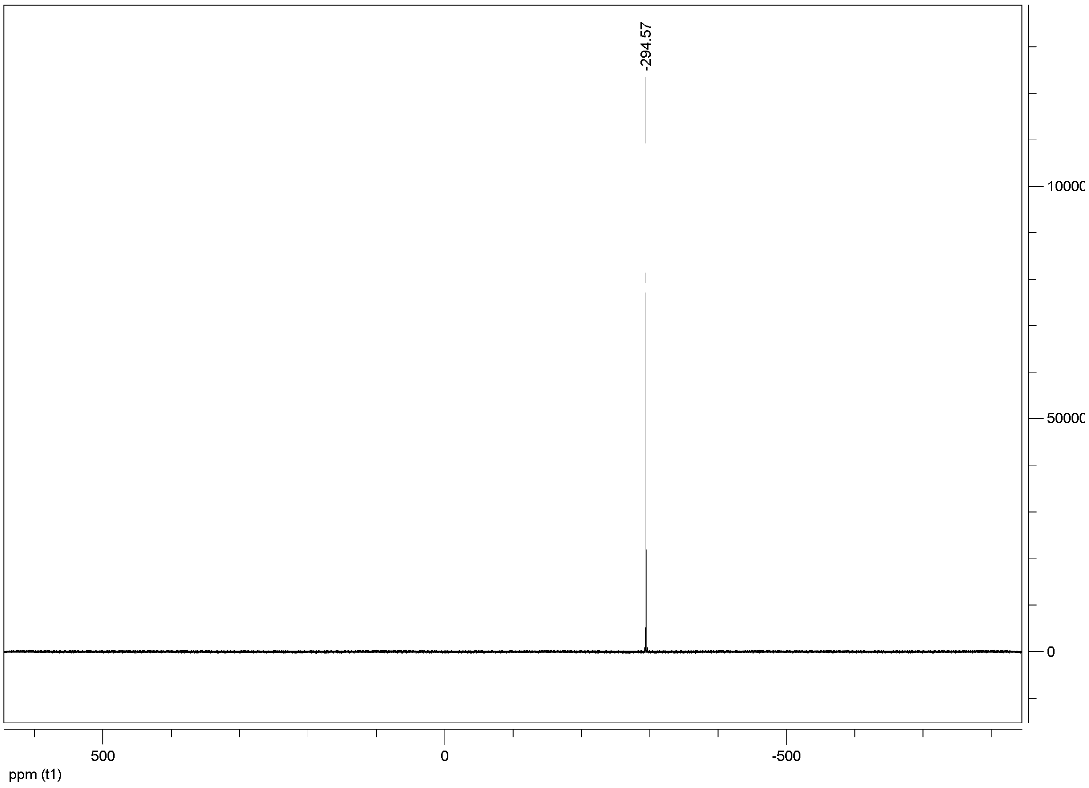

Figure S60 The ^{119}Sn NMR spectra of **6a**

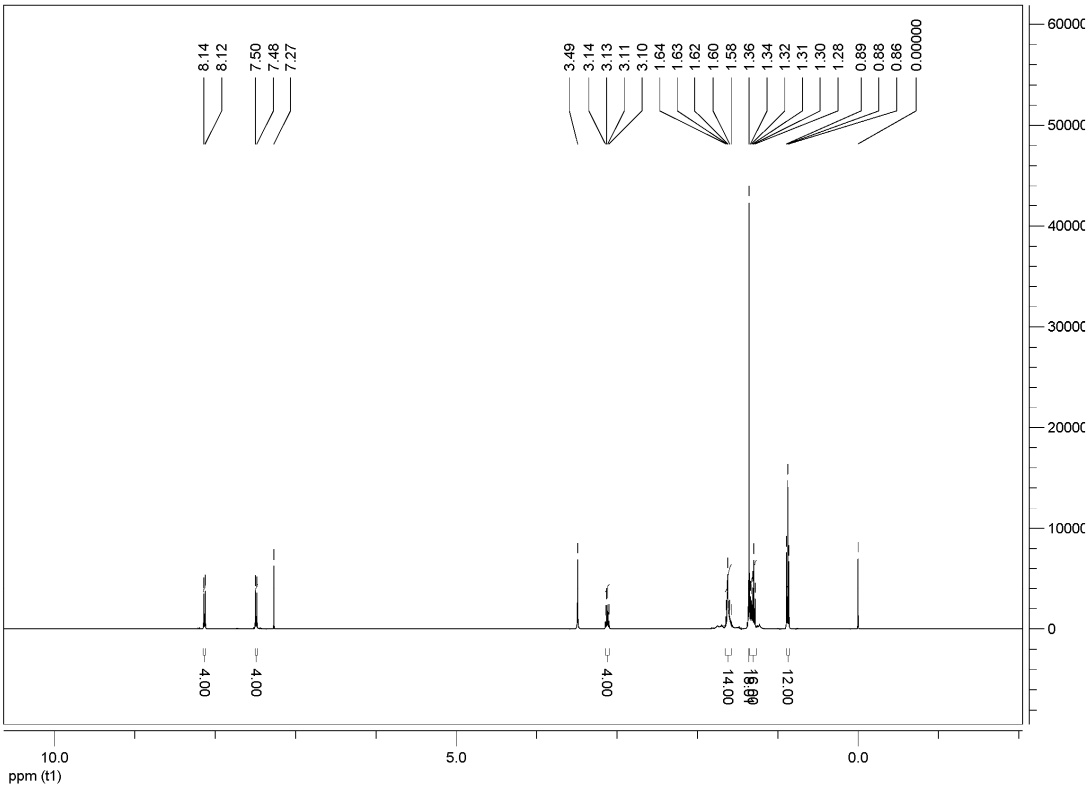

Figure S61 The ^{1}H NMR spectra of **6b**

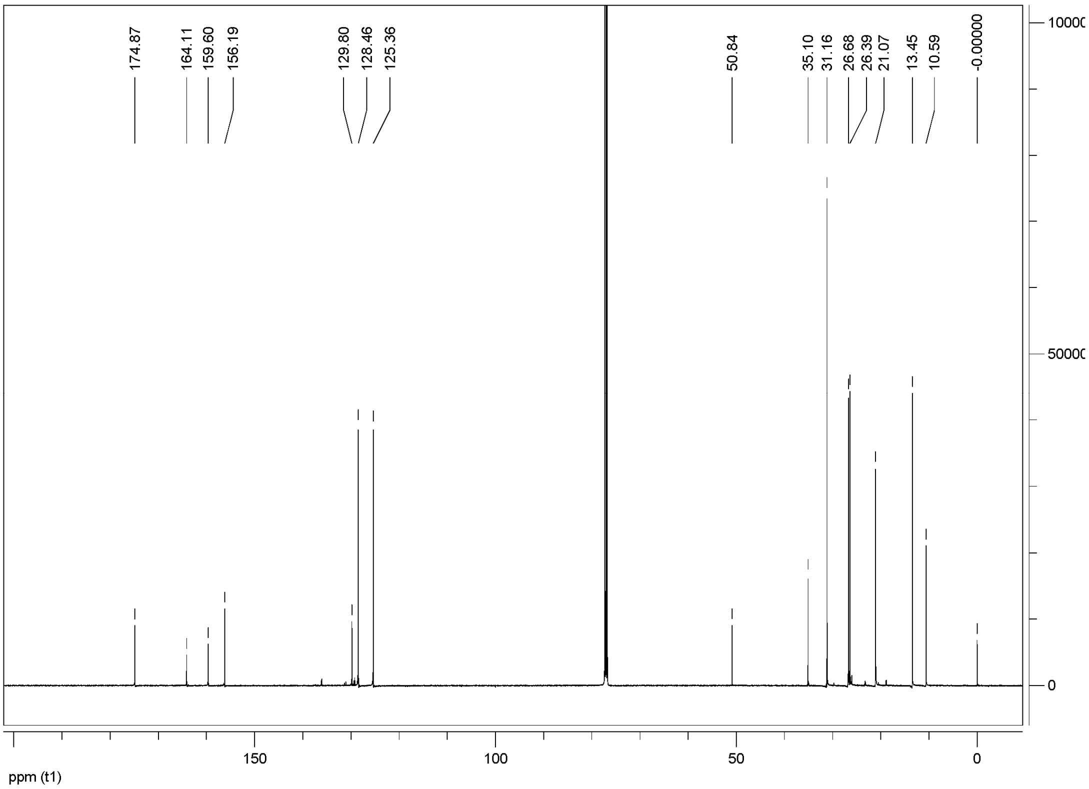

Figure S62 The ^{13}C NMR spectra of **6b**

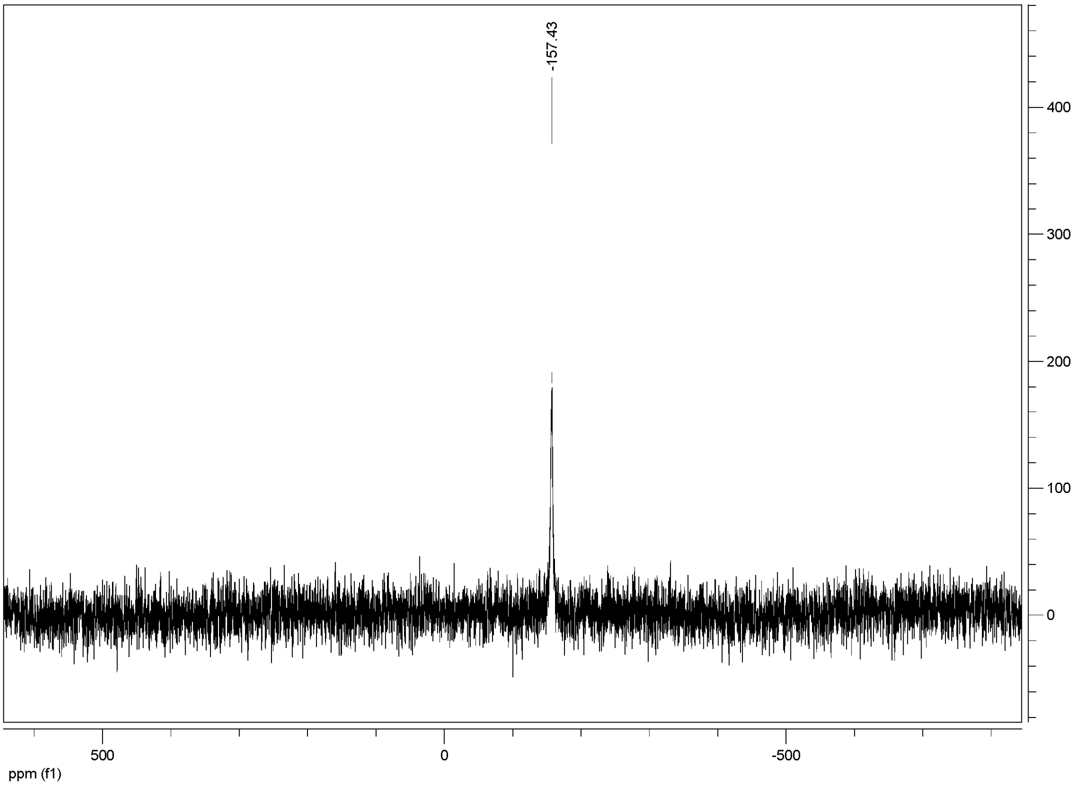

Figure S63 The ^{119}Sn NMR spectra of **6b**

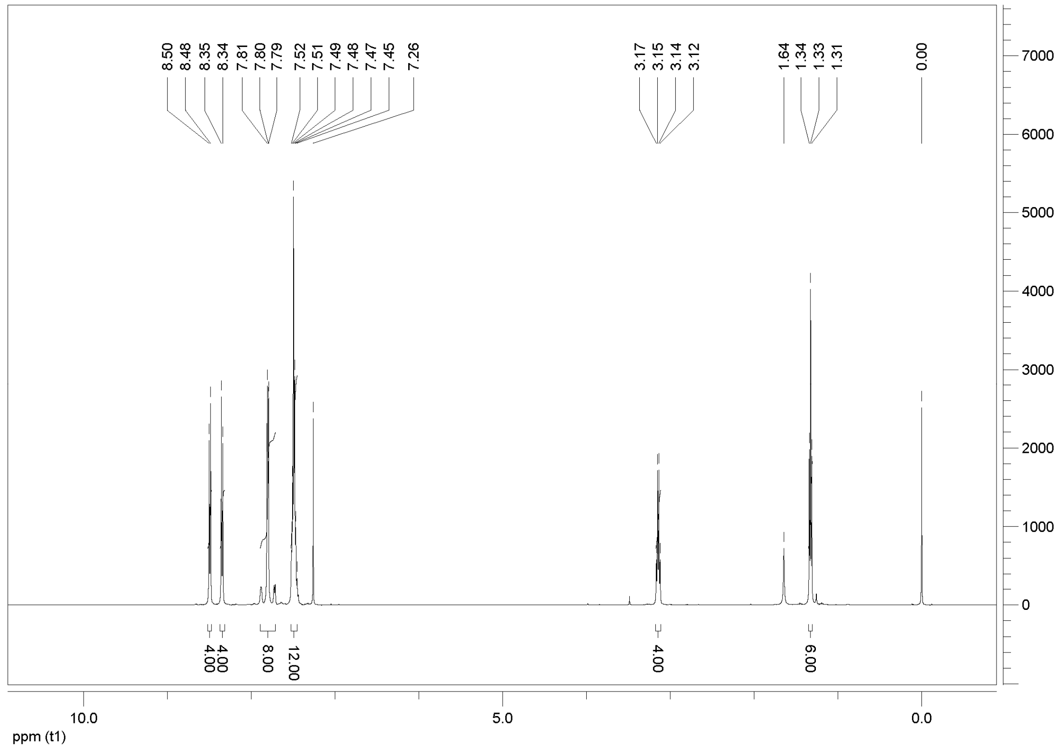

Figure S64 The ^{1}H NMR spectra of **7a**

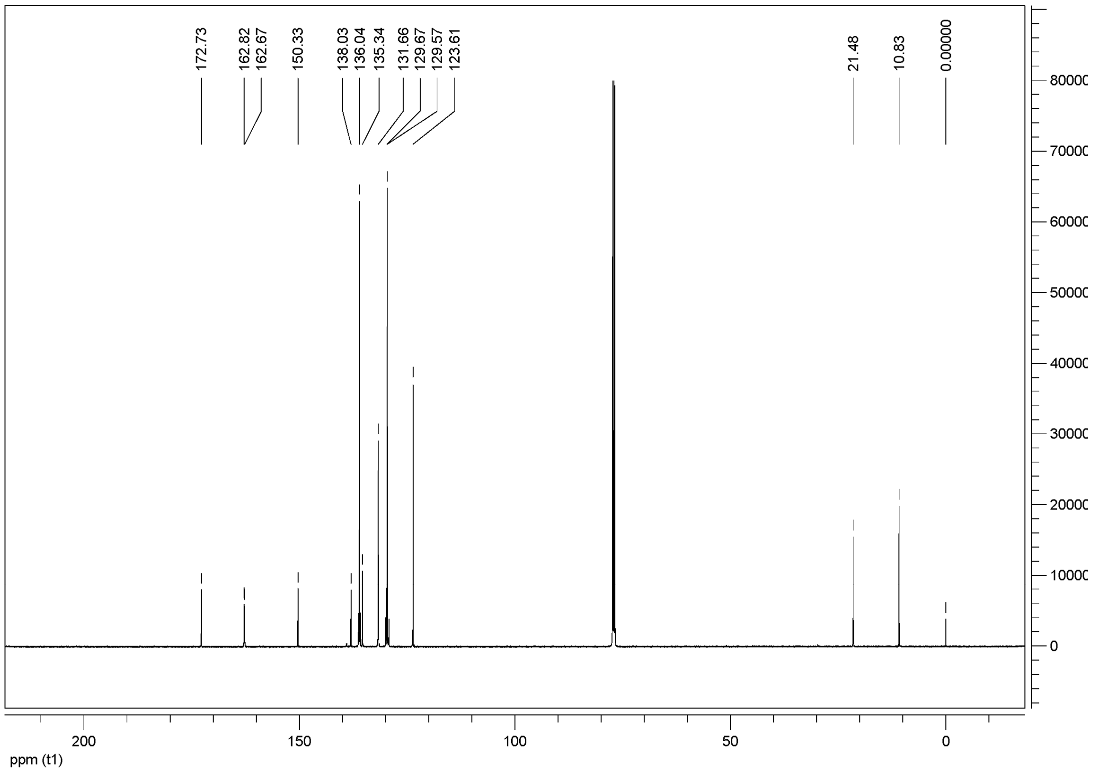

Figure S65 The ^{13}C NMR spectra of **7a**

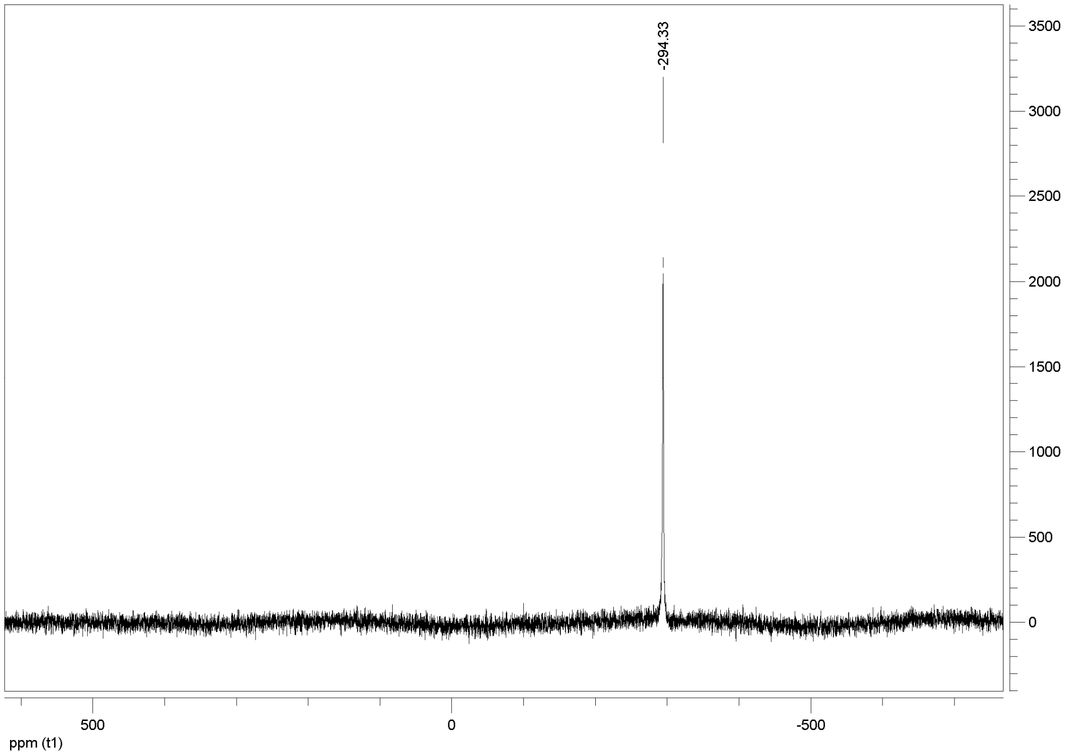

Figure S66 The ^{119}Sn NMR spectra of **7a**

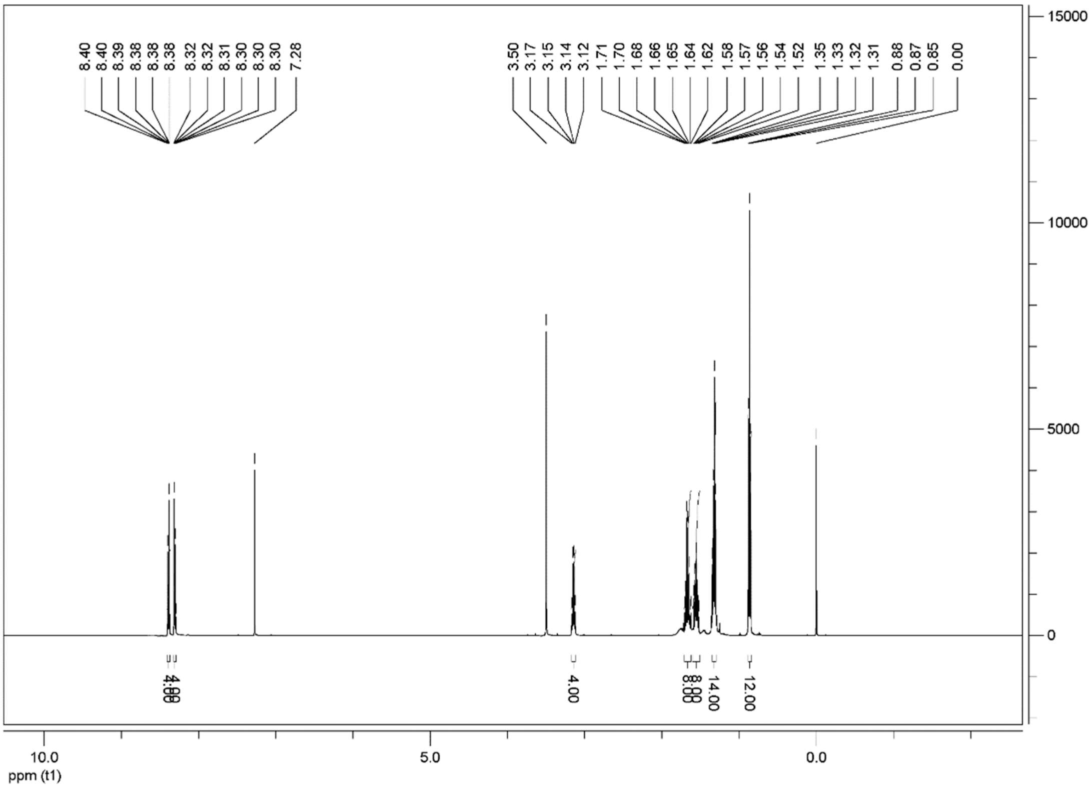

Figure S67 The ^{1}H NMR spectra of **7b**

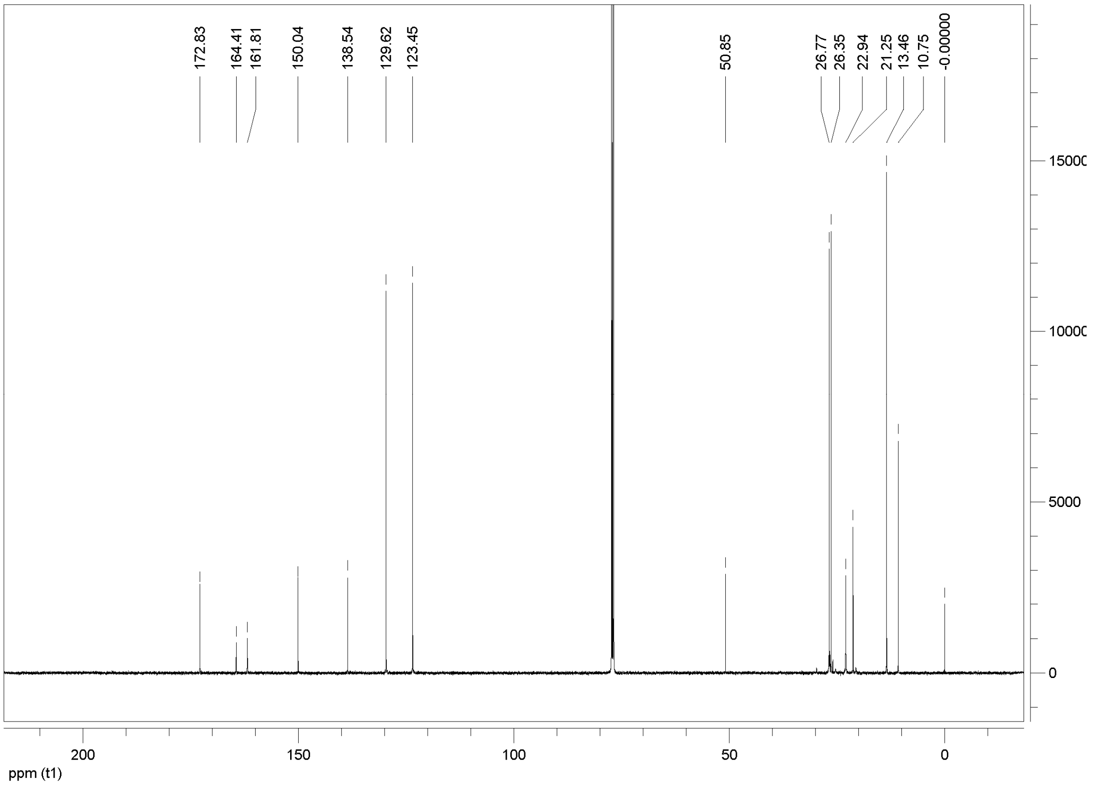

Figure S68 The ^{13}C NMR spectra of **7b**

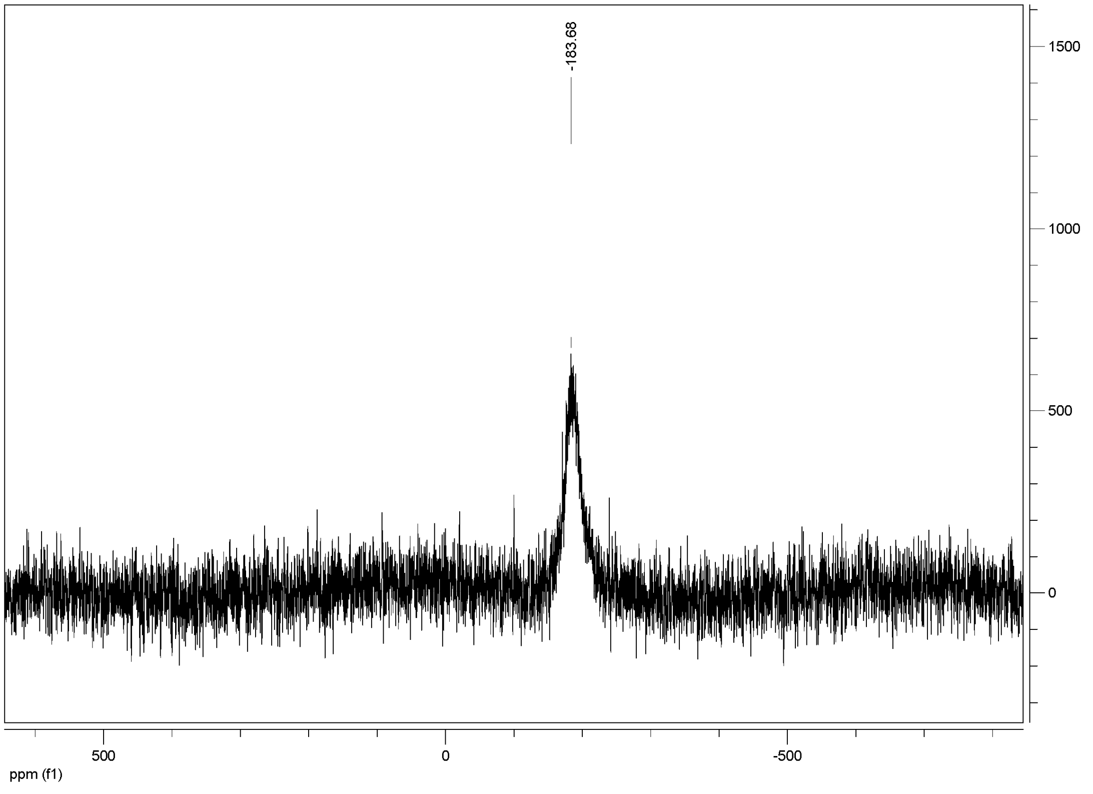

Figure S69 The ^{119}Sn NMR spectra of **7b**

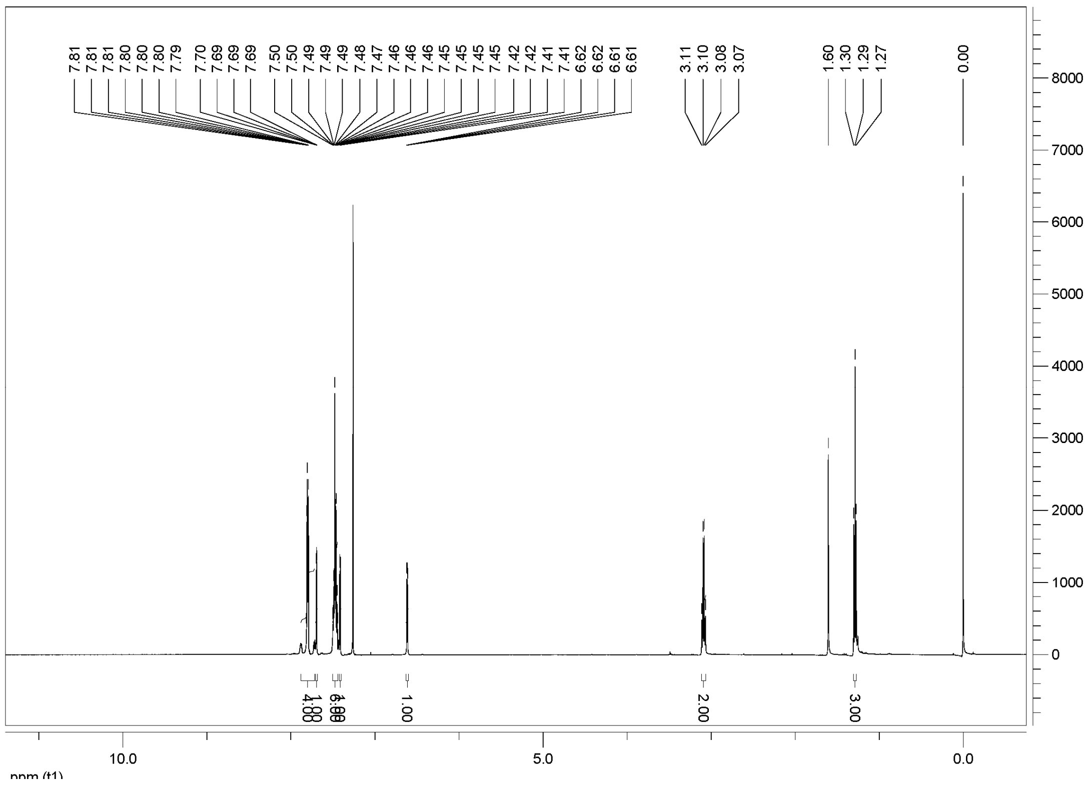

Figure S70 The ^{1}H NMR spectra of **8a**

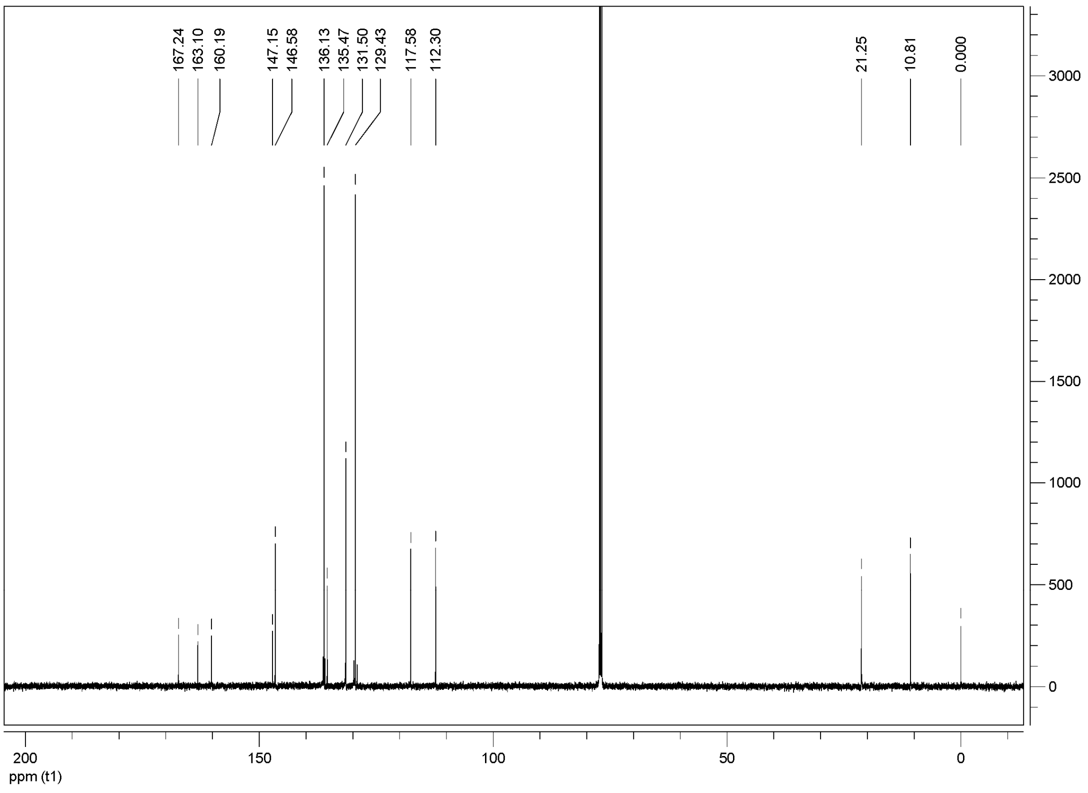

Figure S71 The ^{13}C NMR spectra of **8a**

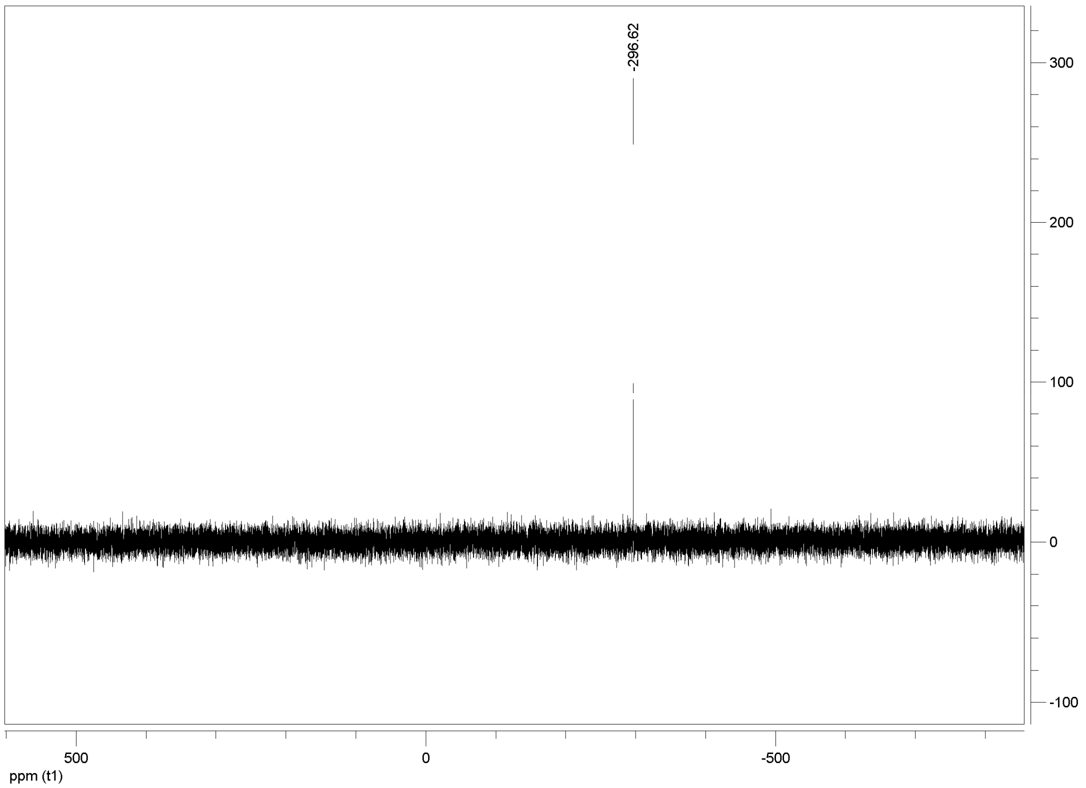

Figure S72 The ^{119}Sn NMR spectra of **8a**

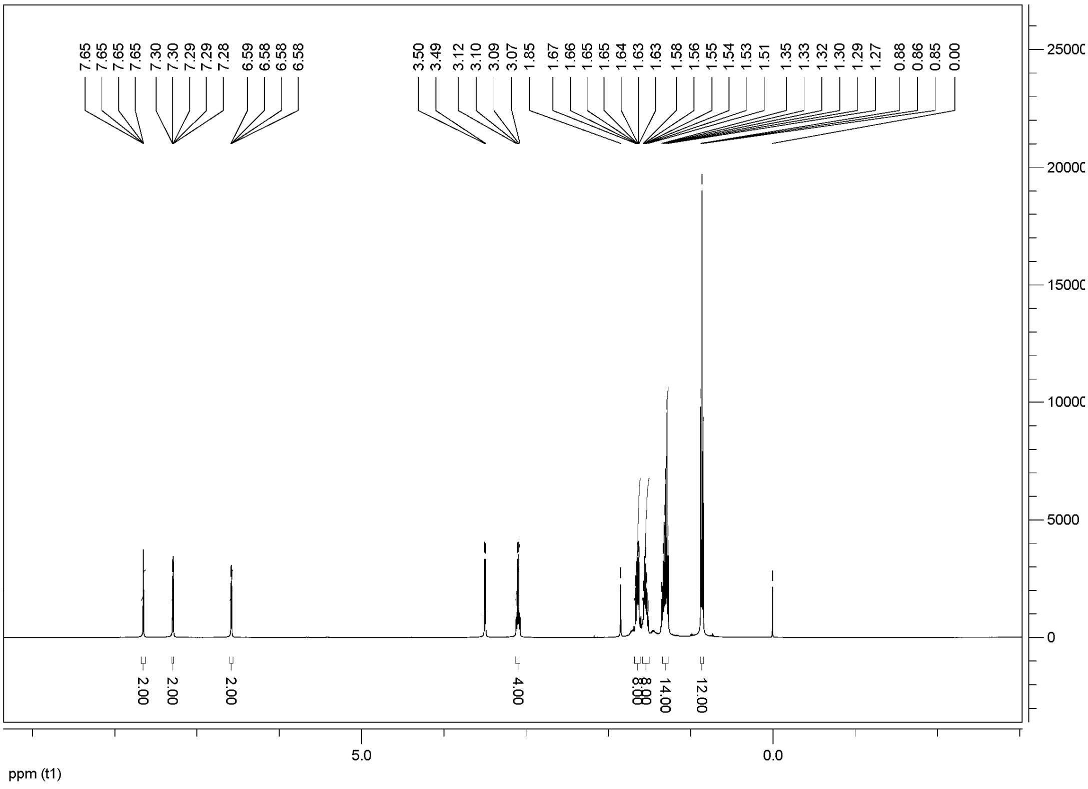

Figure S73 The ^{1}H NMR spectra of **8b**

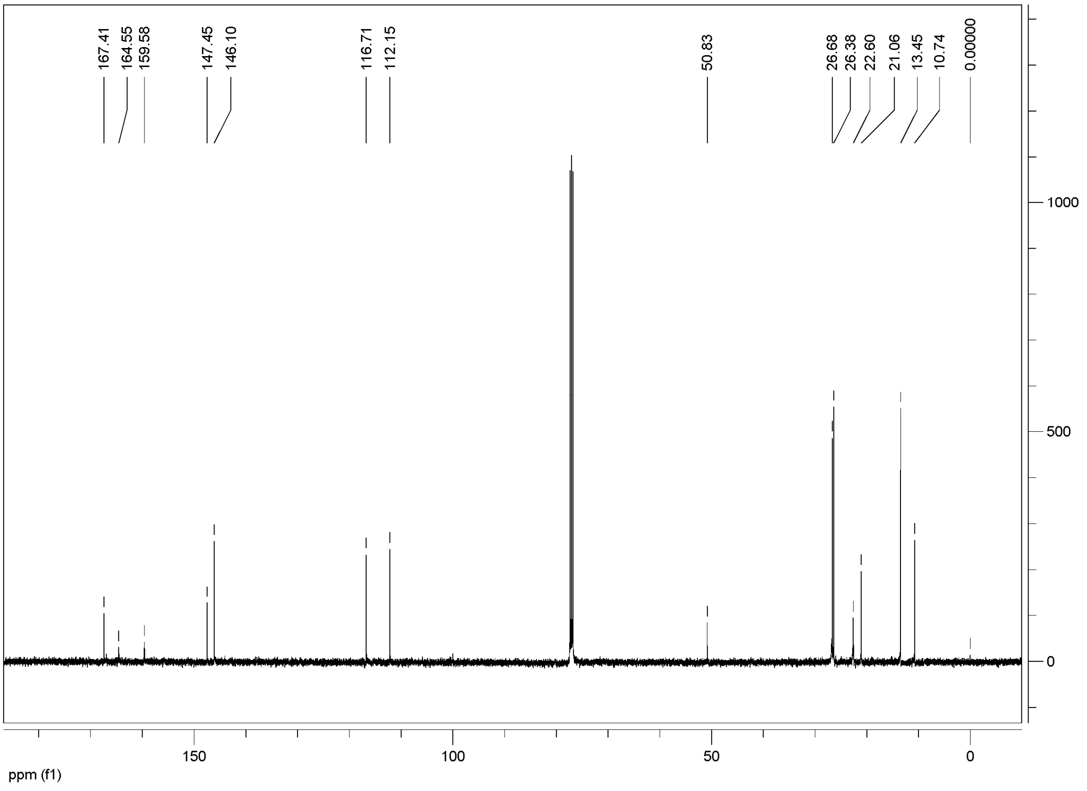

Figure S74 The ^{13}C NMR spectra of **8b**

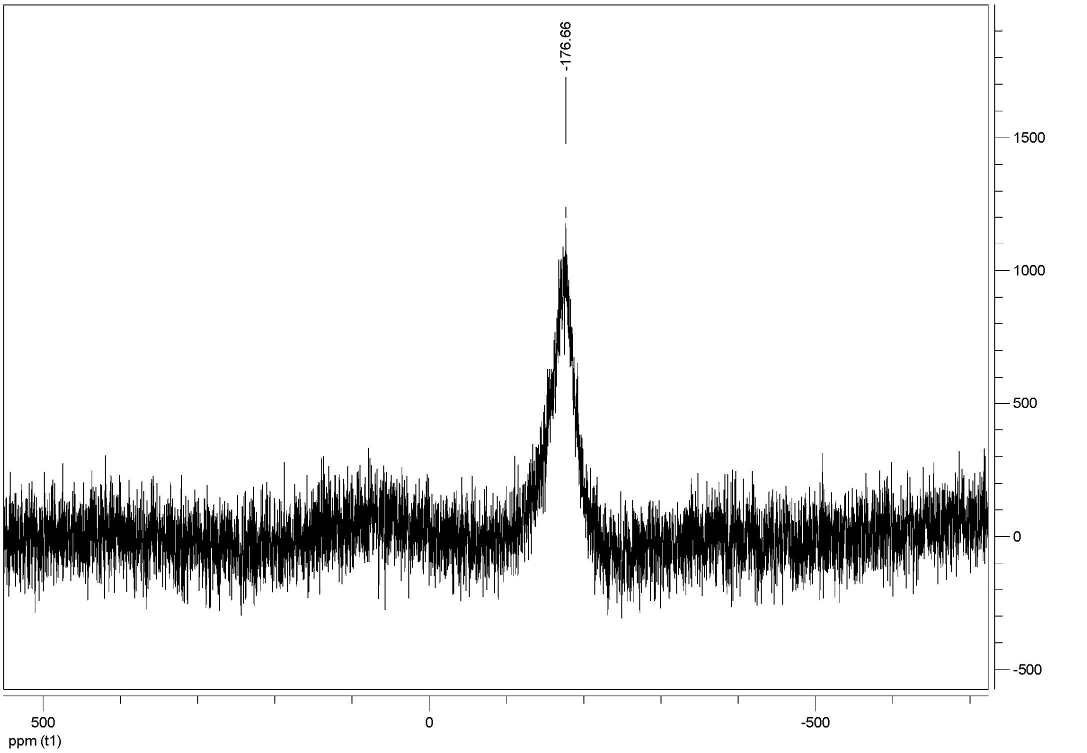

Figure S75 The ^{119}Sn NMR spectra of **8b**

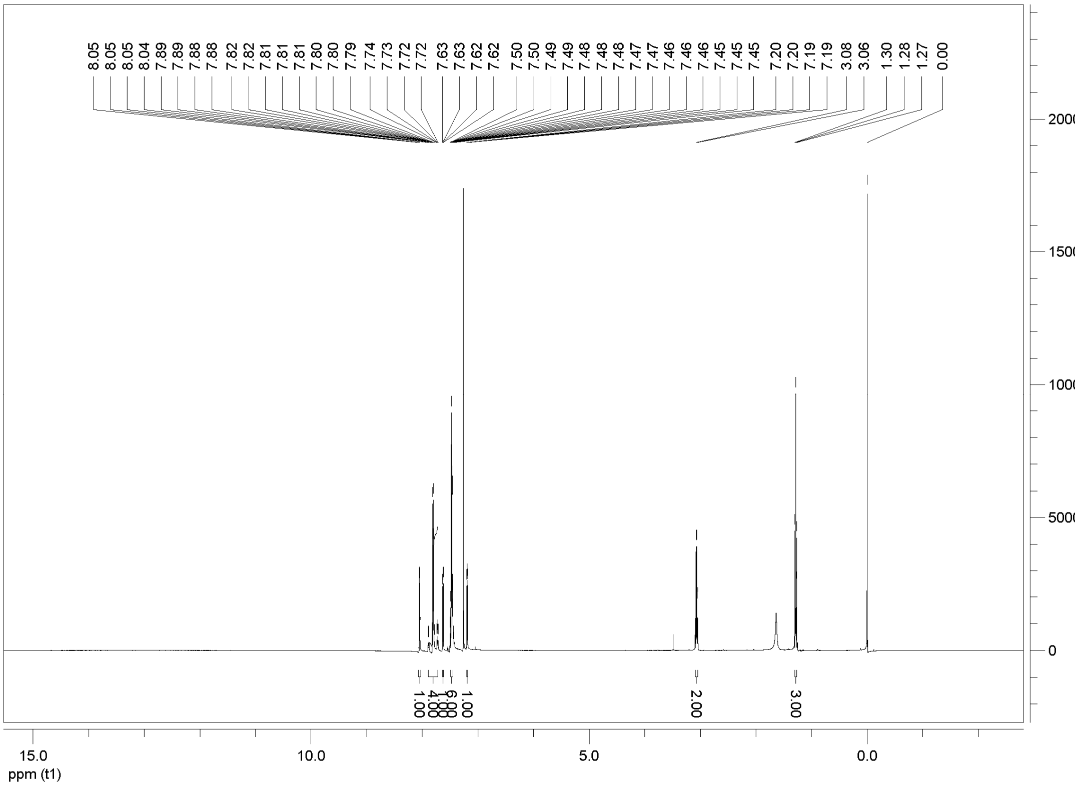

Figure S76 The ^{1}H NMR spectra of **9a**

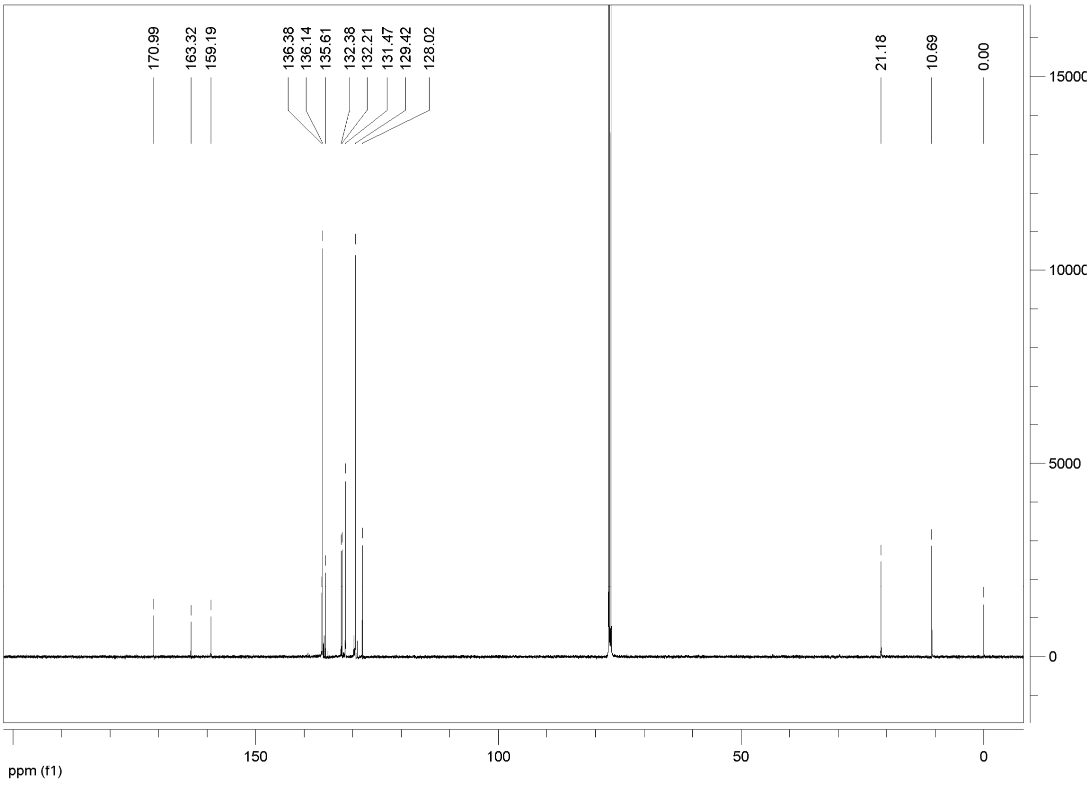

Figure S77 The ^{13}C NMR spectra of **9a**

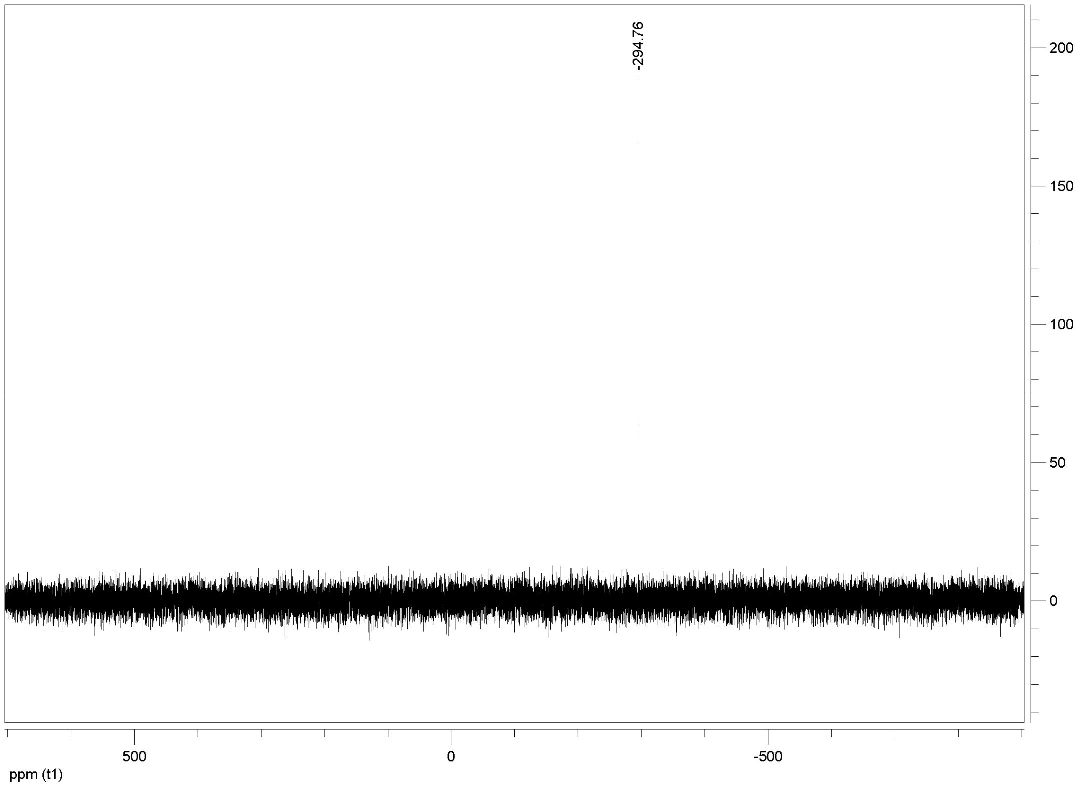

Figure S78 The ^{119}Sn NMR spectra of **9a**

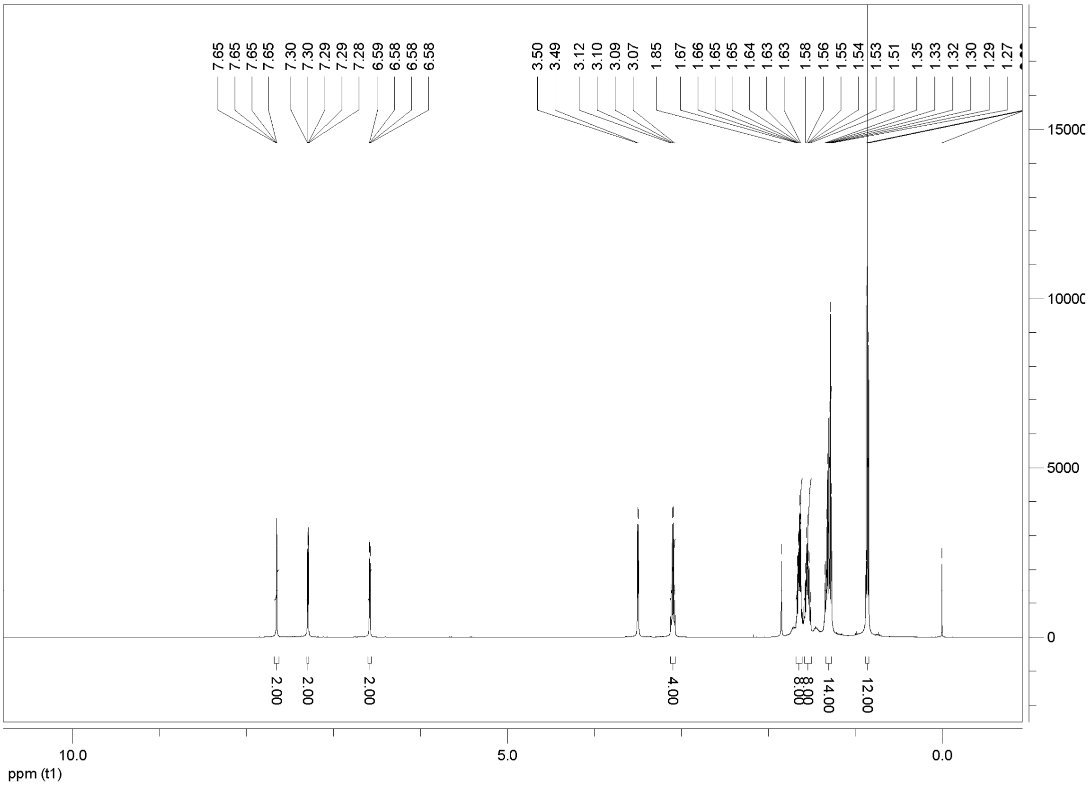

Figure S79 The ^{1}H NMR spectra of **9b**

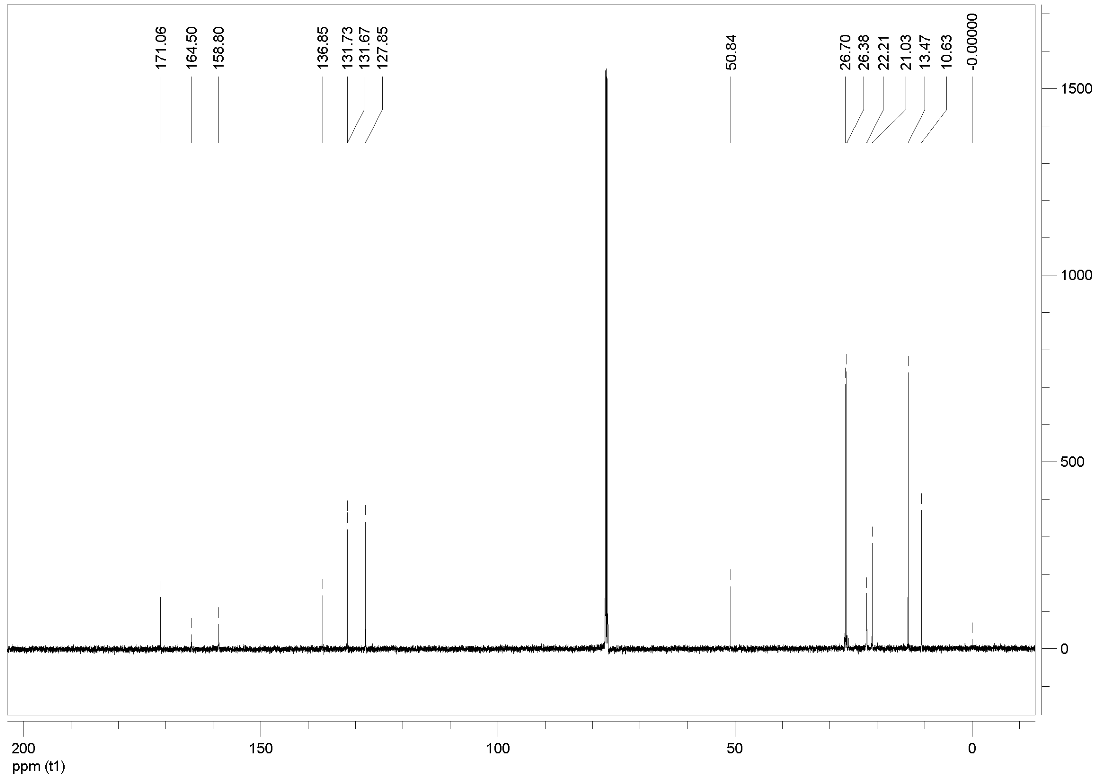

Figure S80 The ^{13}C NMR spectra of **9b**

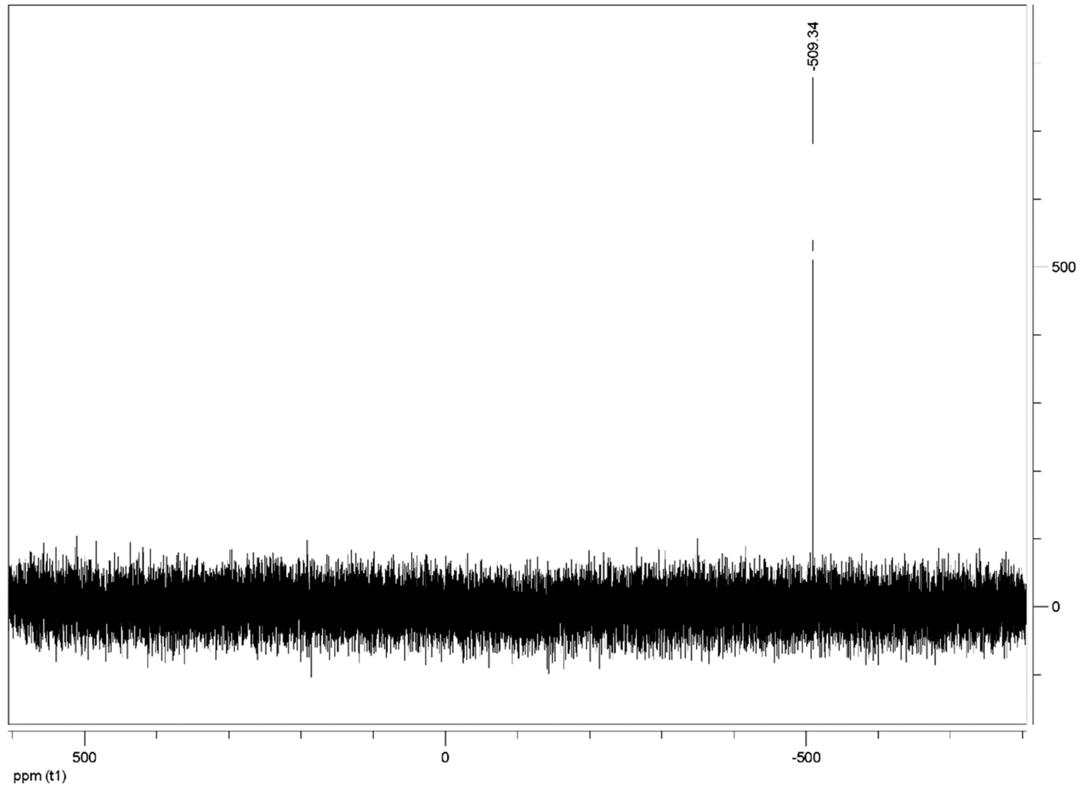

Figure S81 The ^{119}Sn NMR spectra of **9b**

3.3 The HRMS spectra of **1a-9b**.

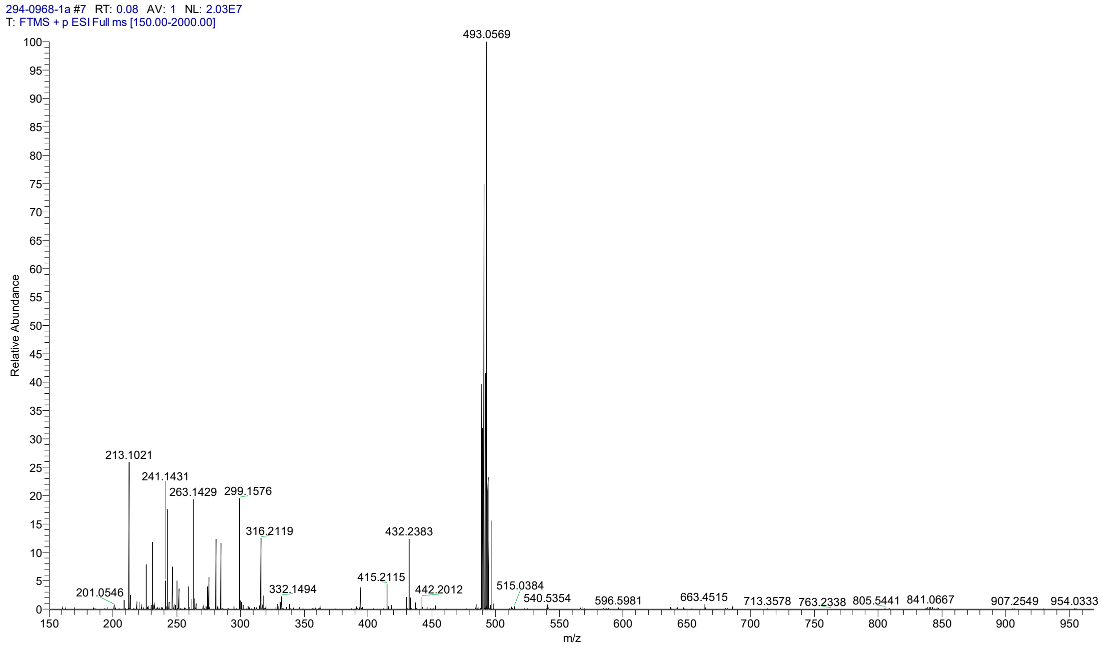

Figure S82 The HRMS spectra of **1a**

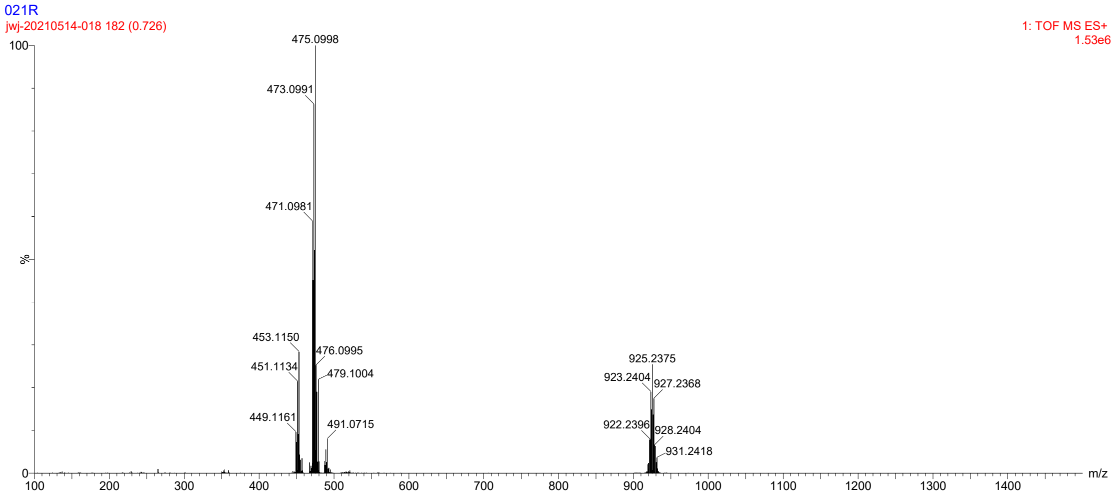

Figure S83 The HRMS spectra of **1b**

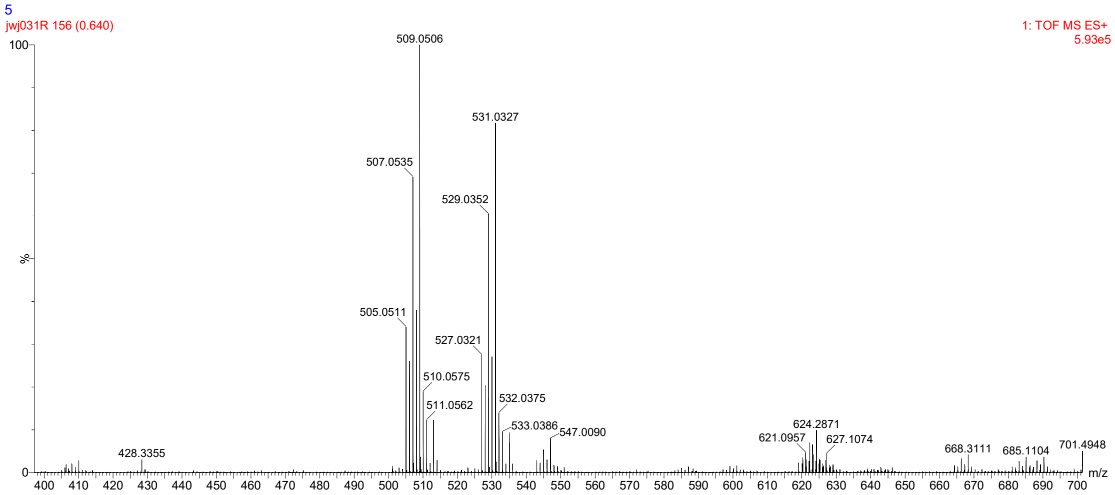

Figure S84 The HRMS spectra of **2a**

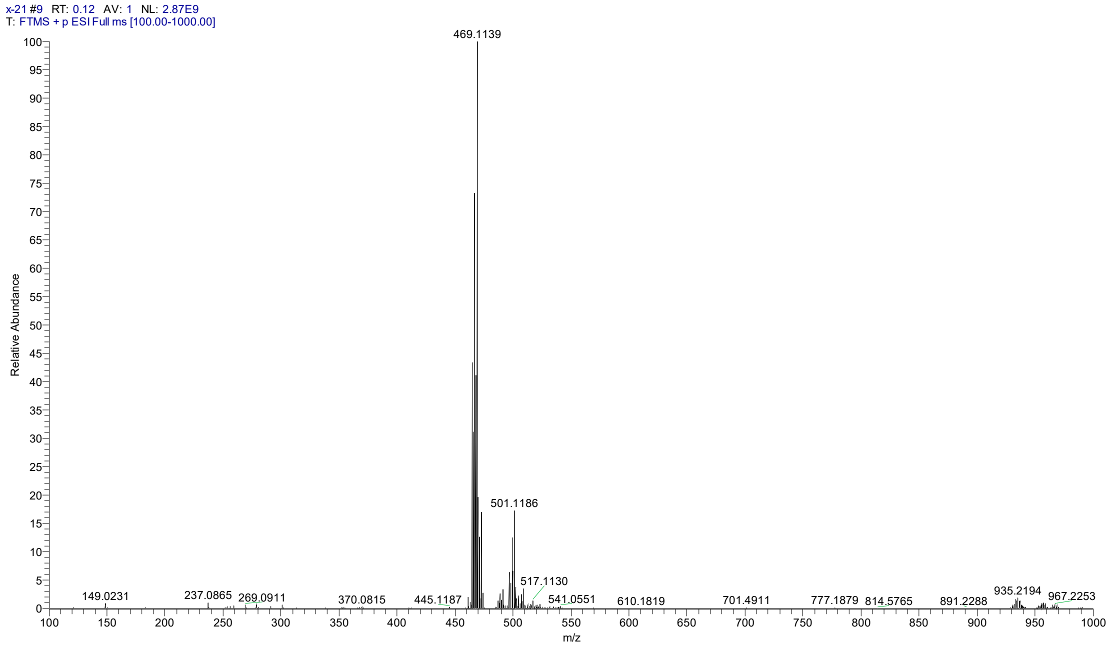

Figure S85 The HRMS spectra of **2b**

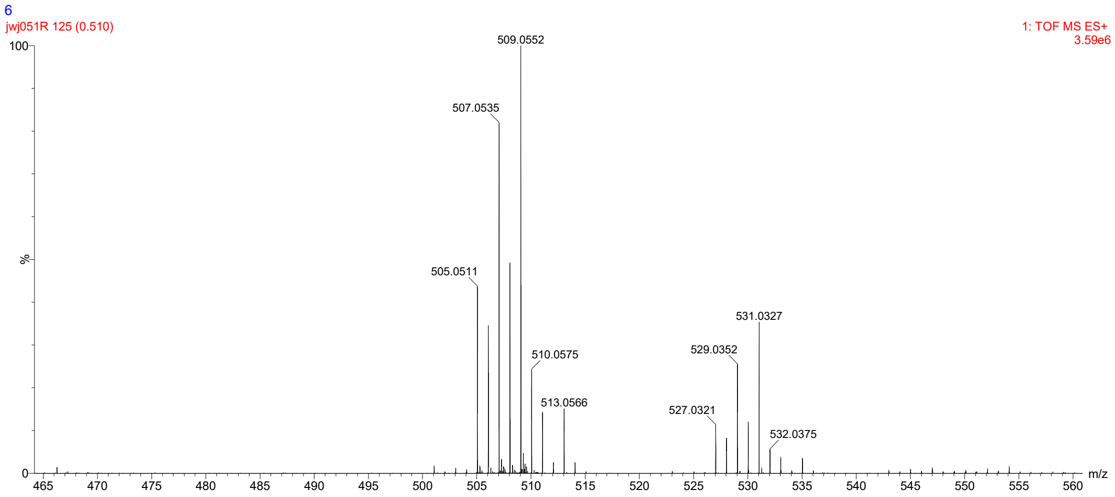

Figure S86 The HRMS spectra of **3a**

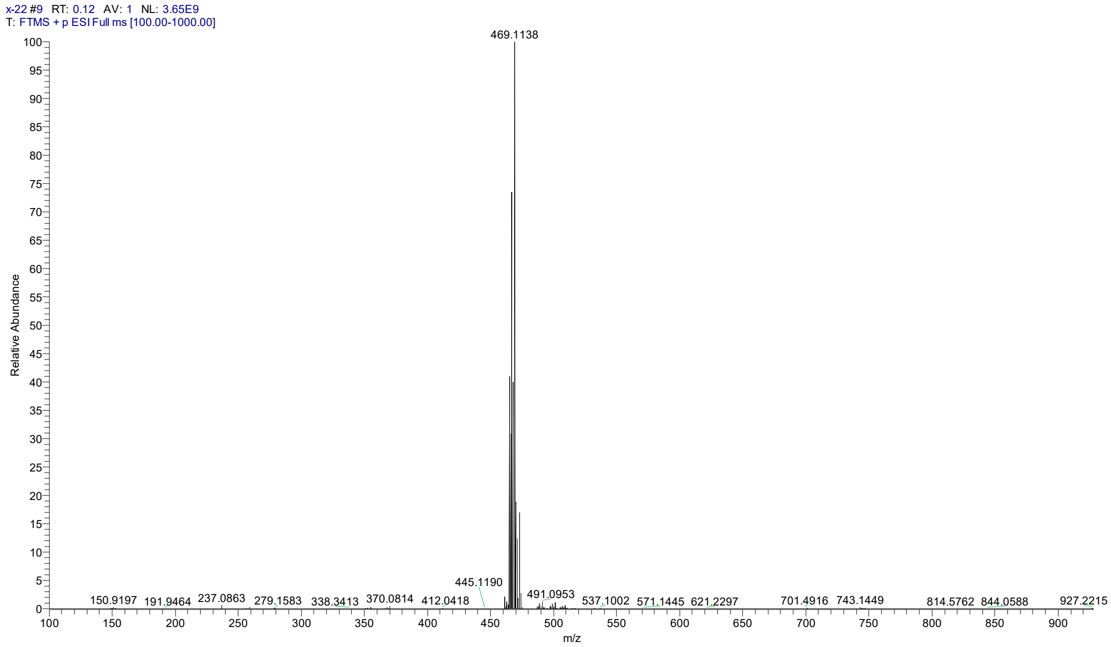

Figure S87 The HRMS spectra of **3b**

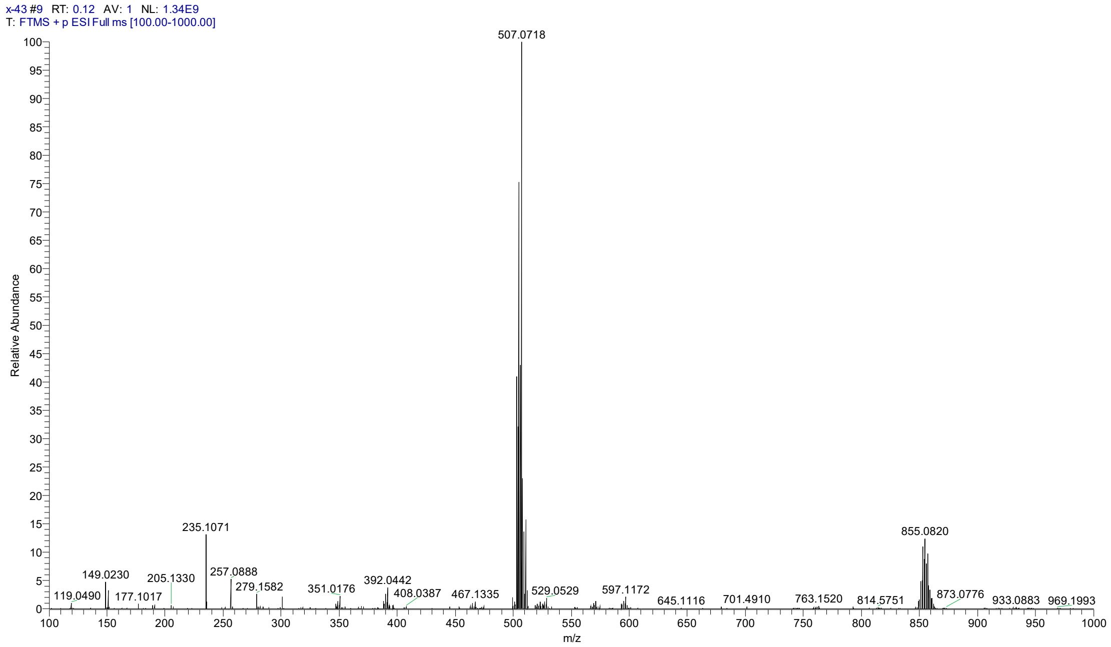

Figure S88 The HRMS spectra of **4a**

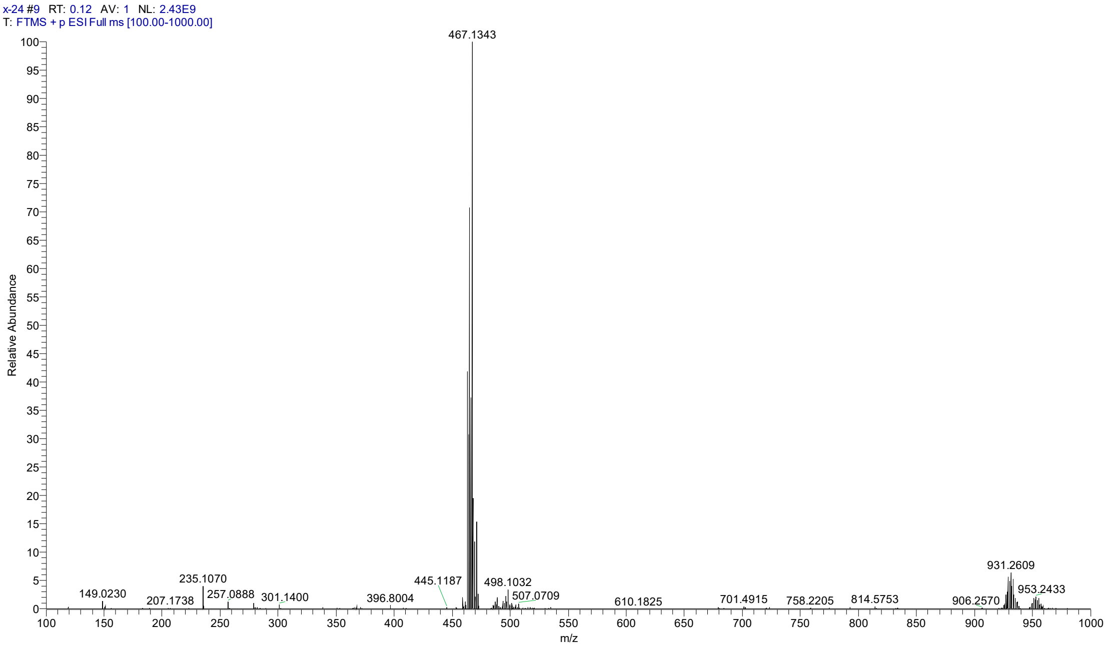

Figure S89 The HRMS spectra of **4b**

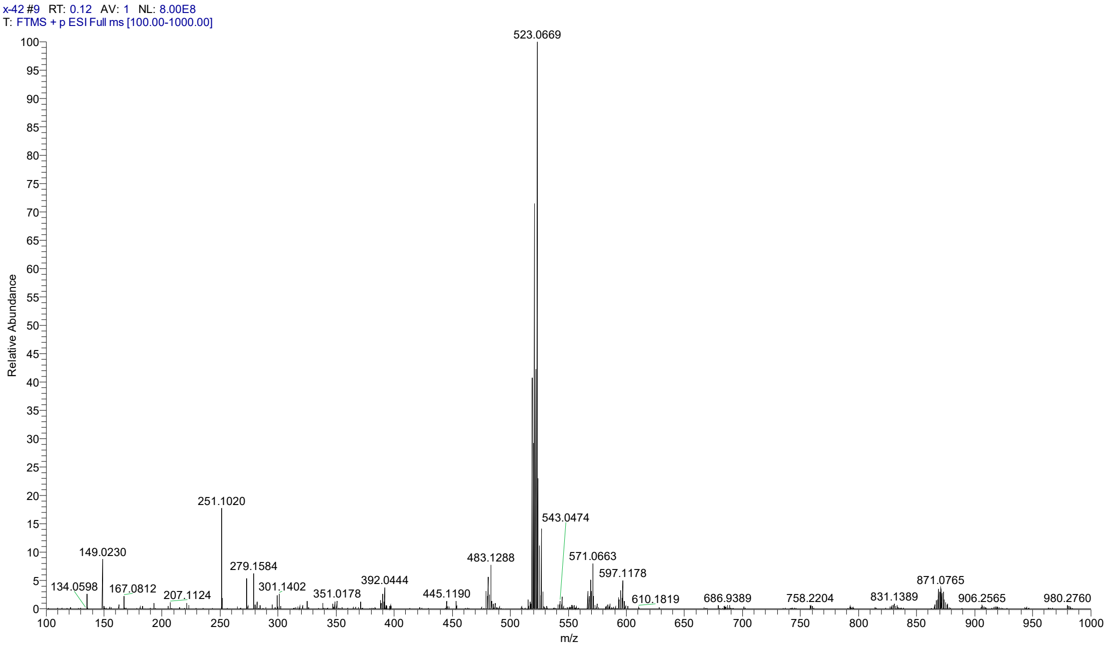

Figure S90 The HRMS spectra of **5a**

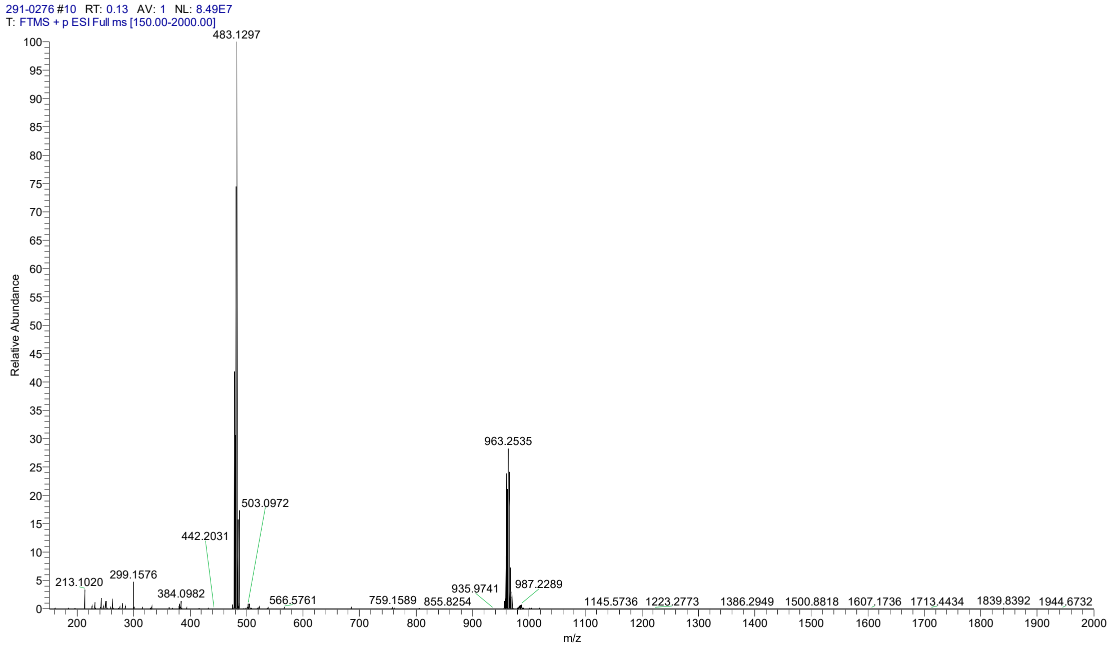

Figure S91 The HRMS spectra of **5b**

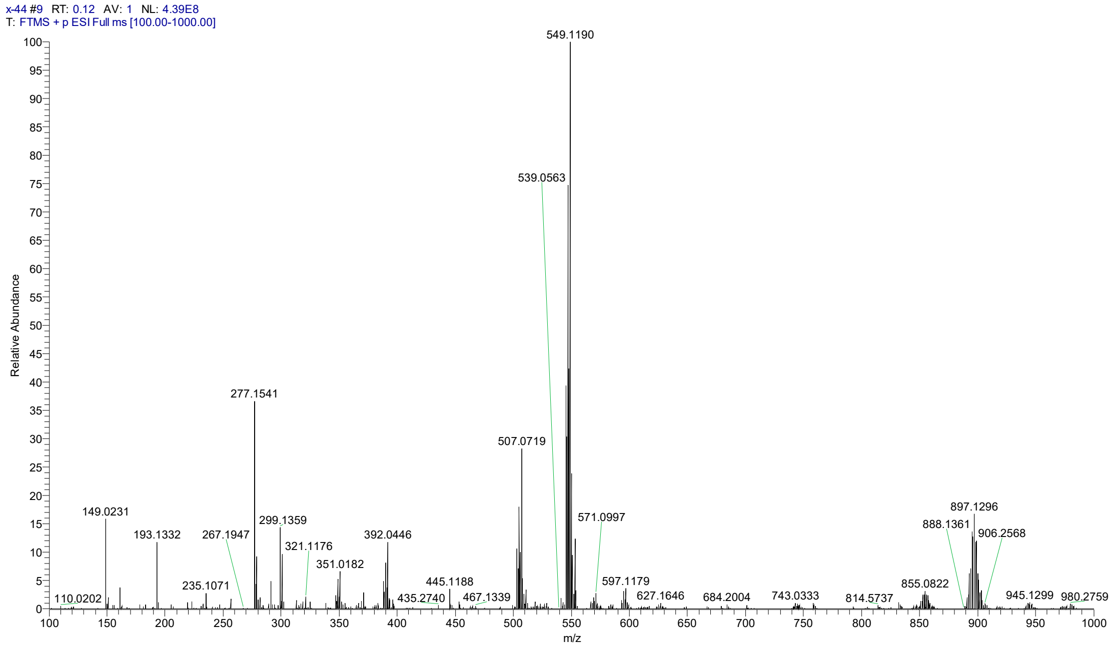

Figure S92 The HRMS spectra of **6a**

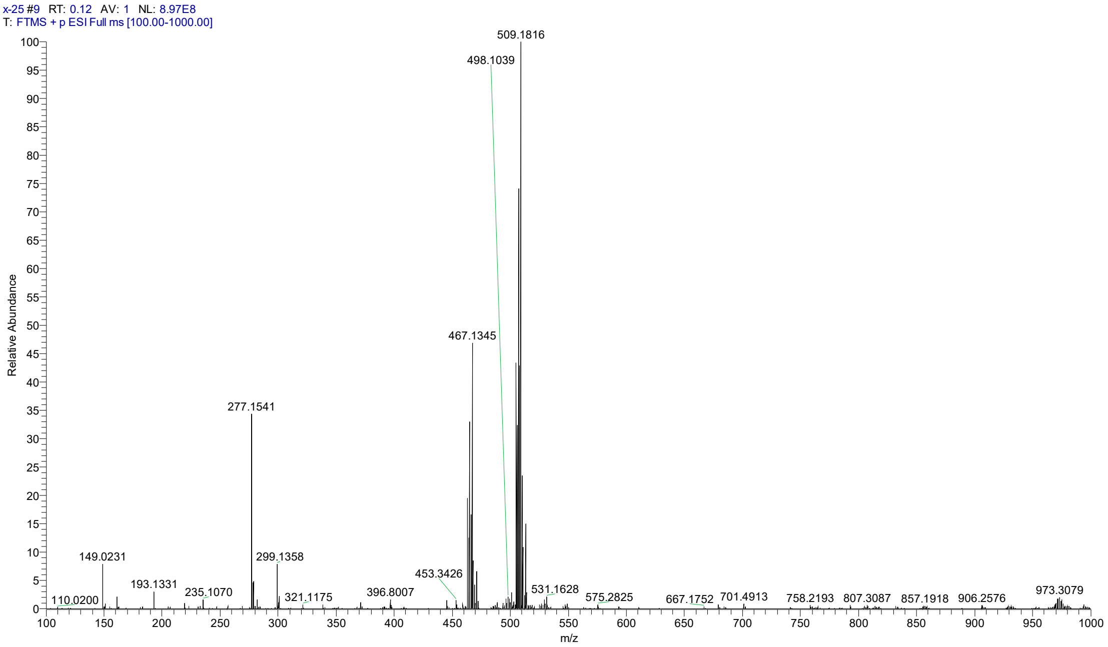

Figure S93 The HRMS spectra of **6b**

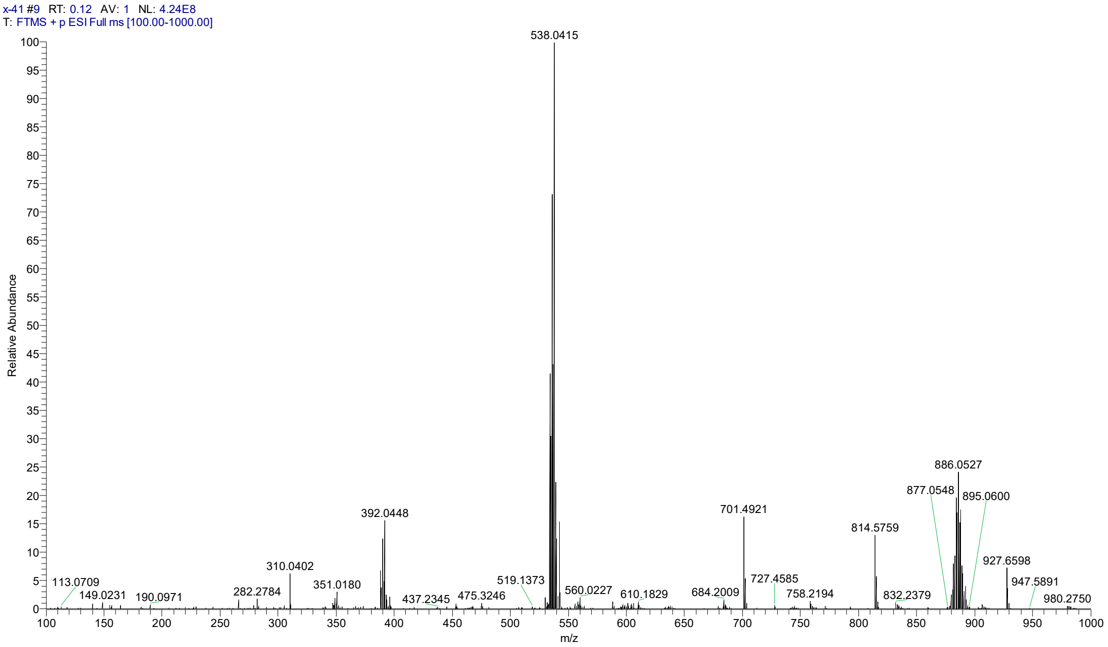

Figure S94 The HRMS spectra of **7a**

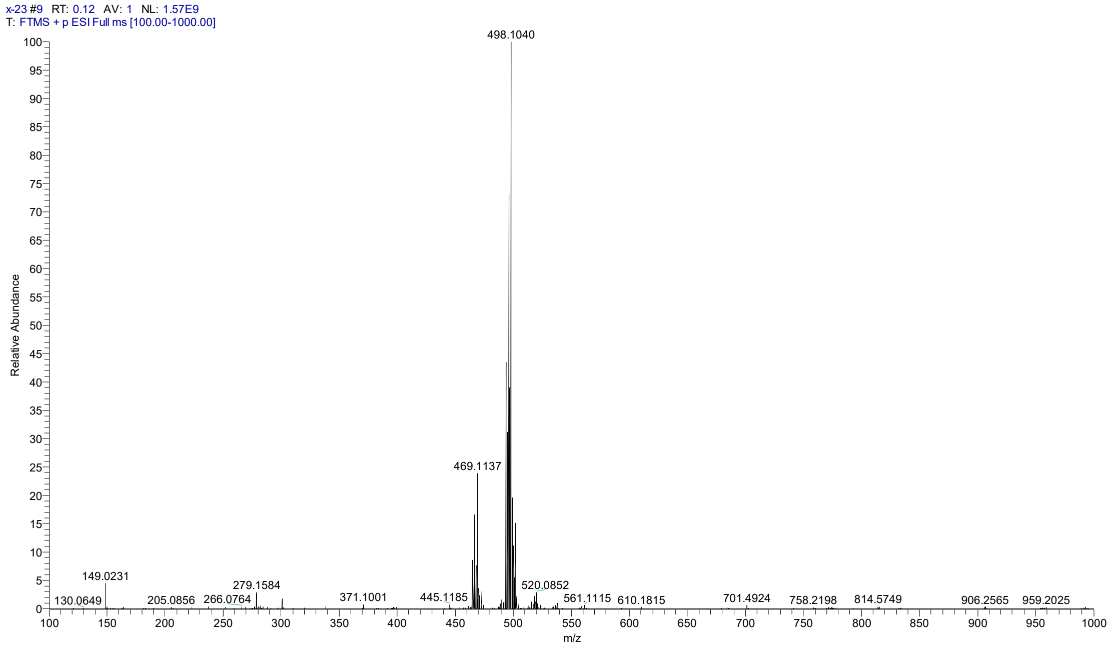

Figure S95 The HRMS spectra of **7b**

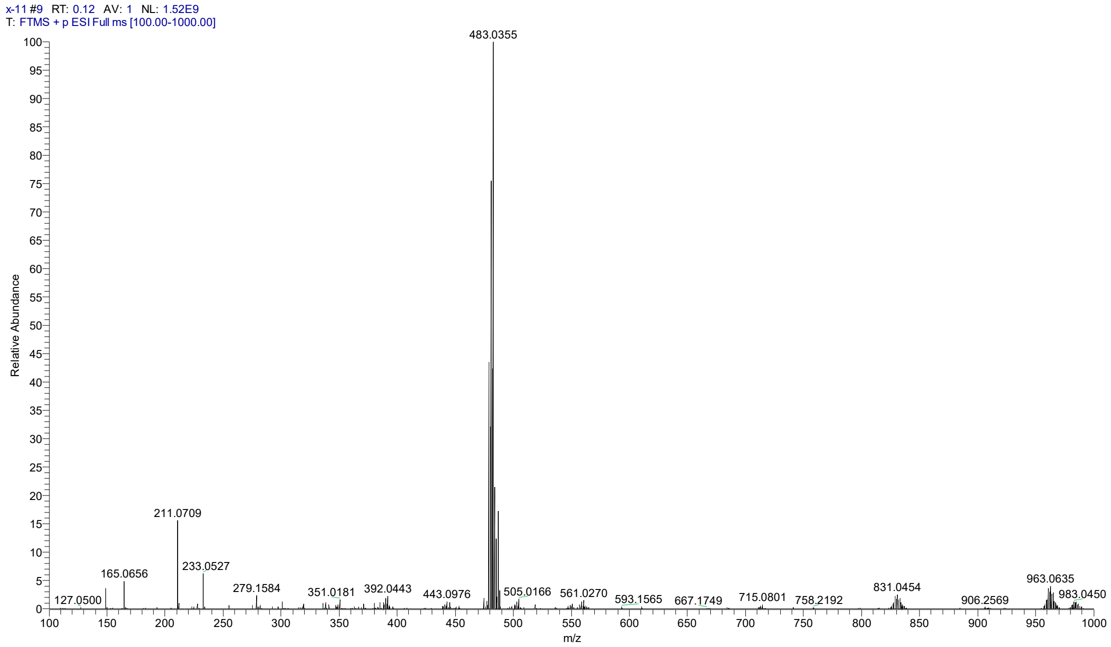

Figure S96 The HRMS spectra of **8a**

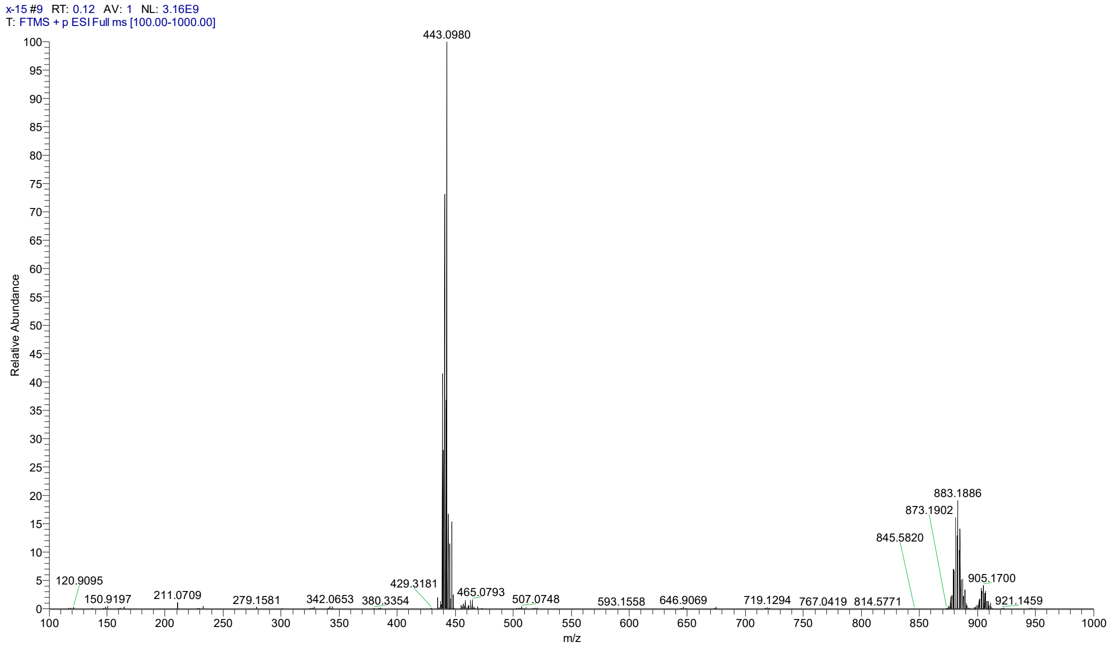

Figure S97 The HRMS spectra of **8b**

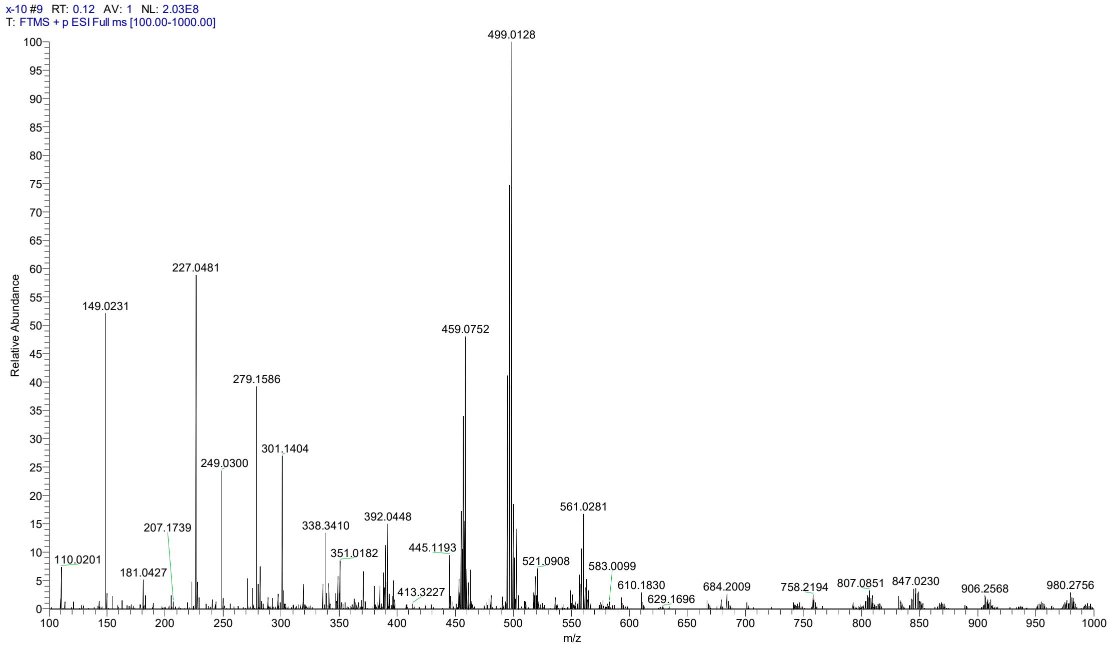

Figure S98 The HRMS spectra of **9a**

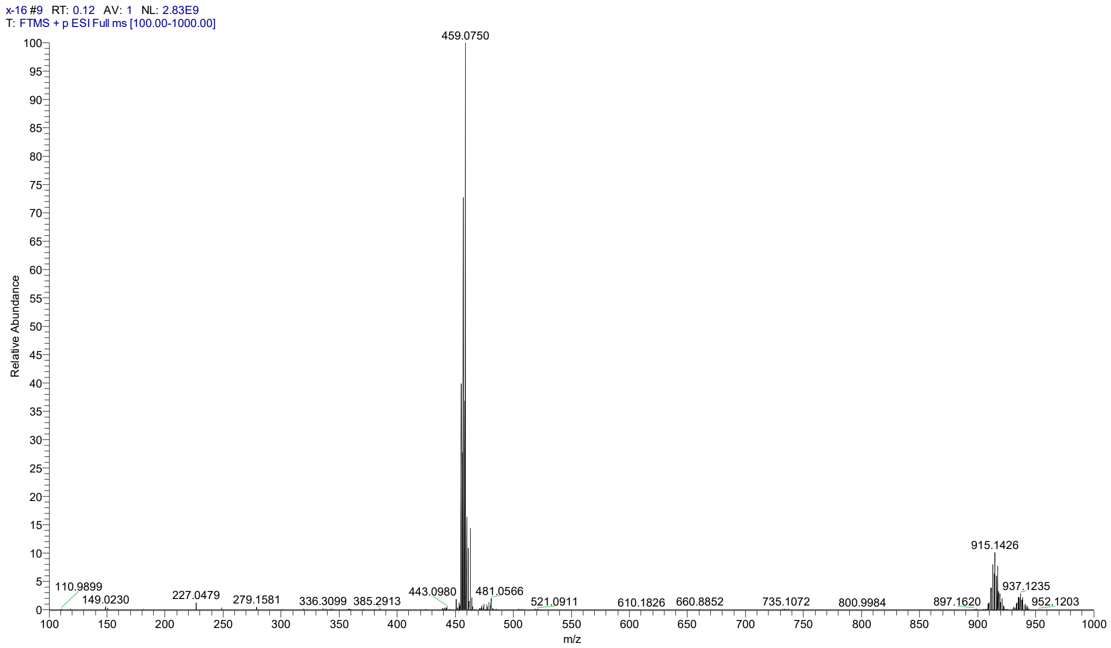

Figure S99 The HRMS spectra of **9b**

3.4 The UV-vis spectra of **1a-9b**.

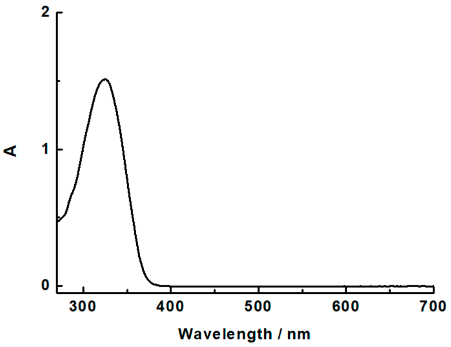

1a

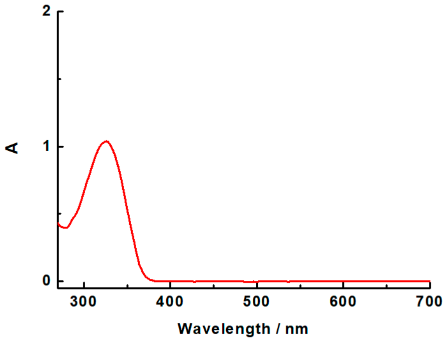

1b

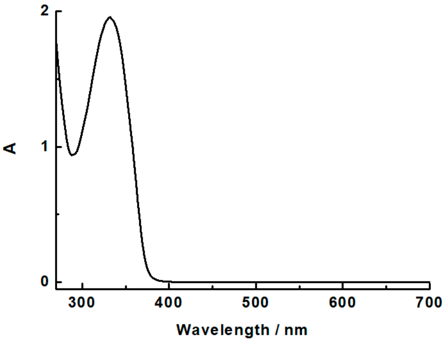

2a

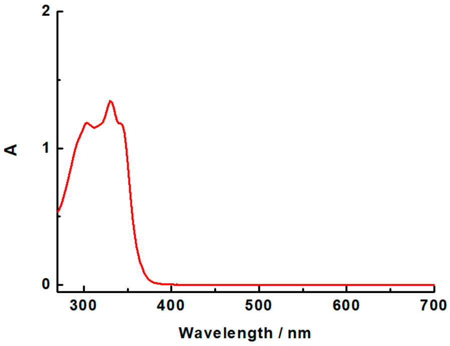

2b

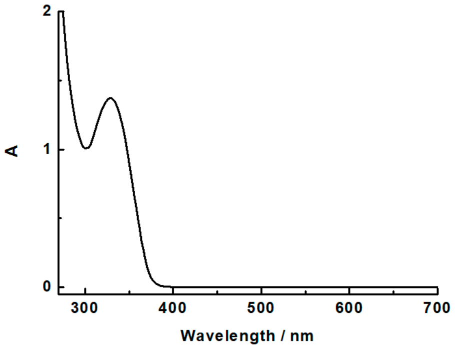

3a

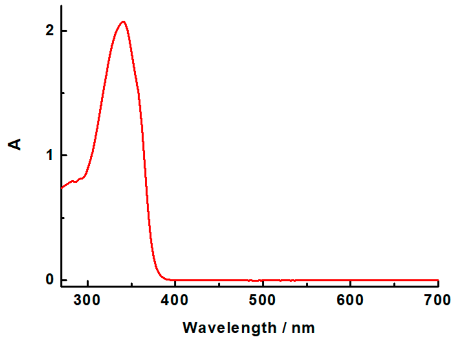

3b

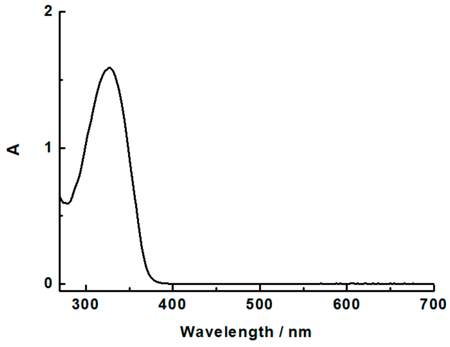

4a

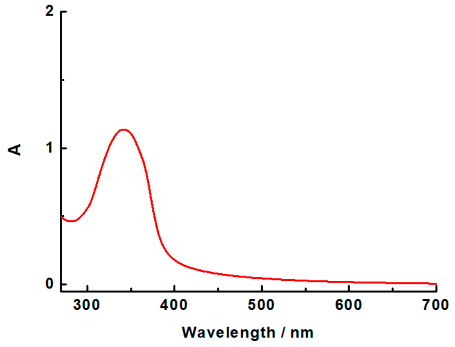

4b

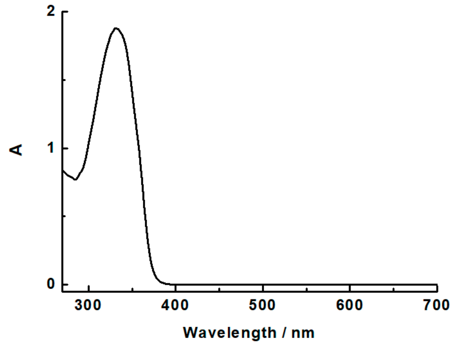

5a

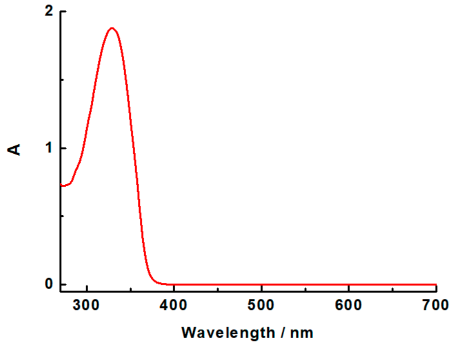

5b

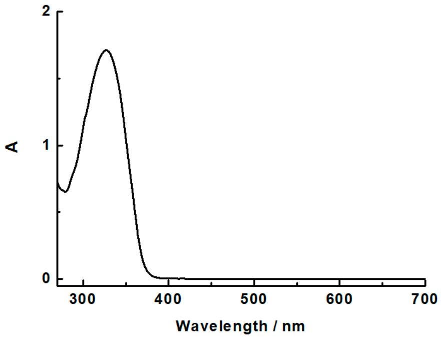

6a

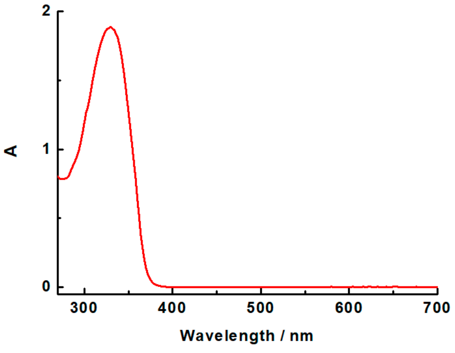

6b

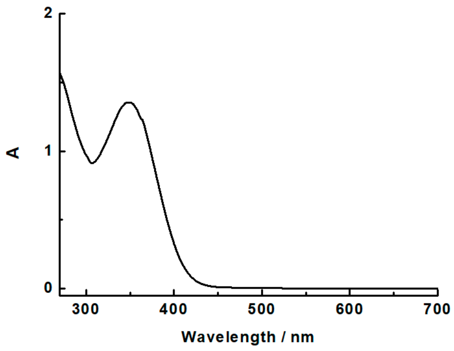

7a

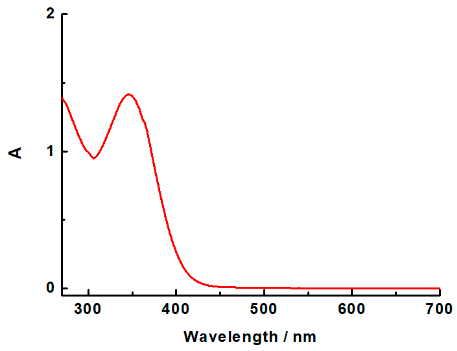

7b

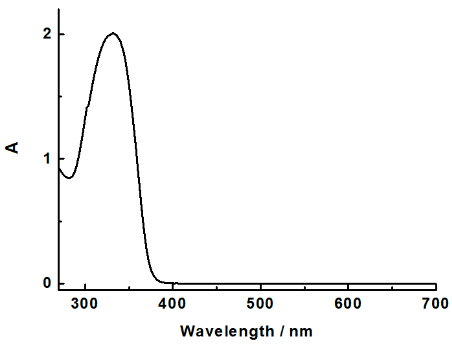

8a

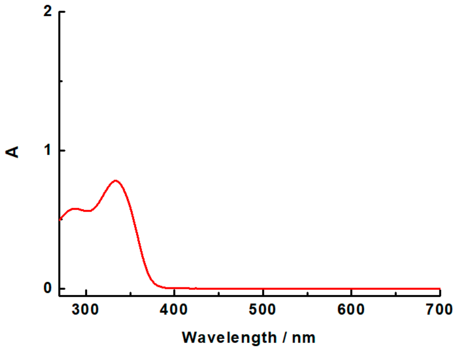

8b

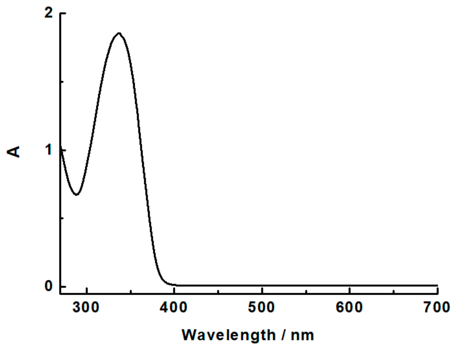

9a

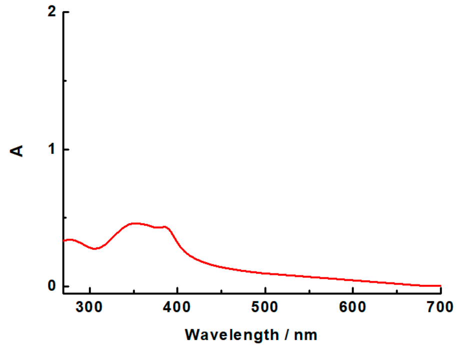

9b

4 TGA

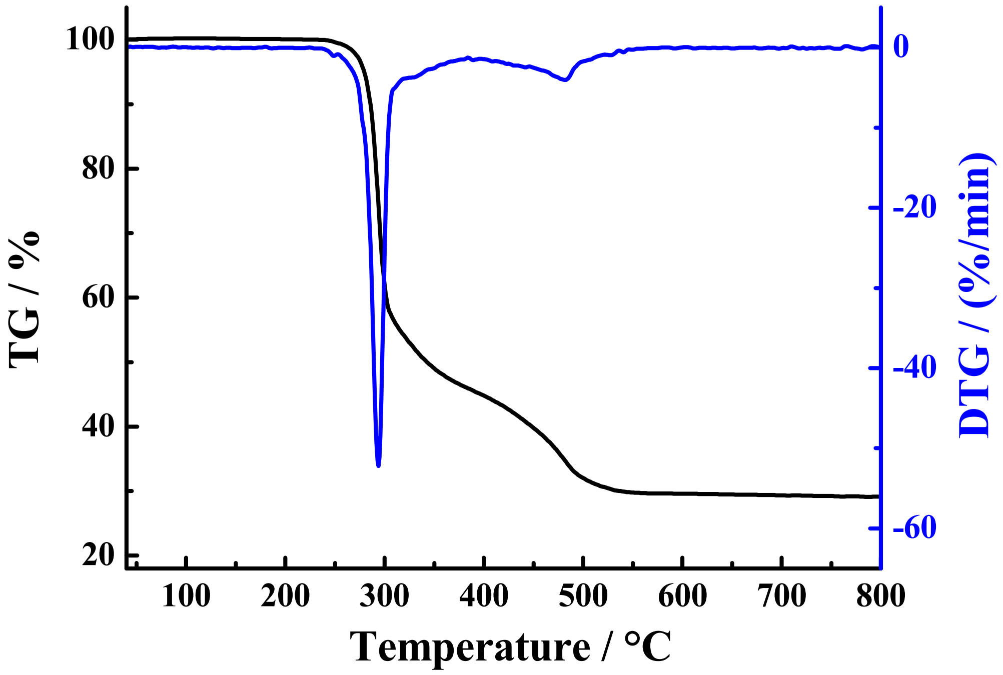

Figure S100 The TG-DTG curve of **1a**

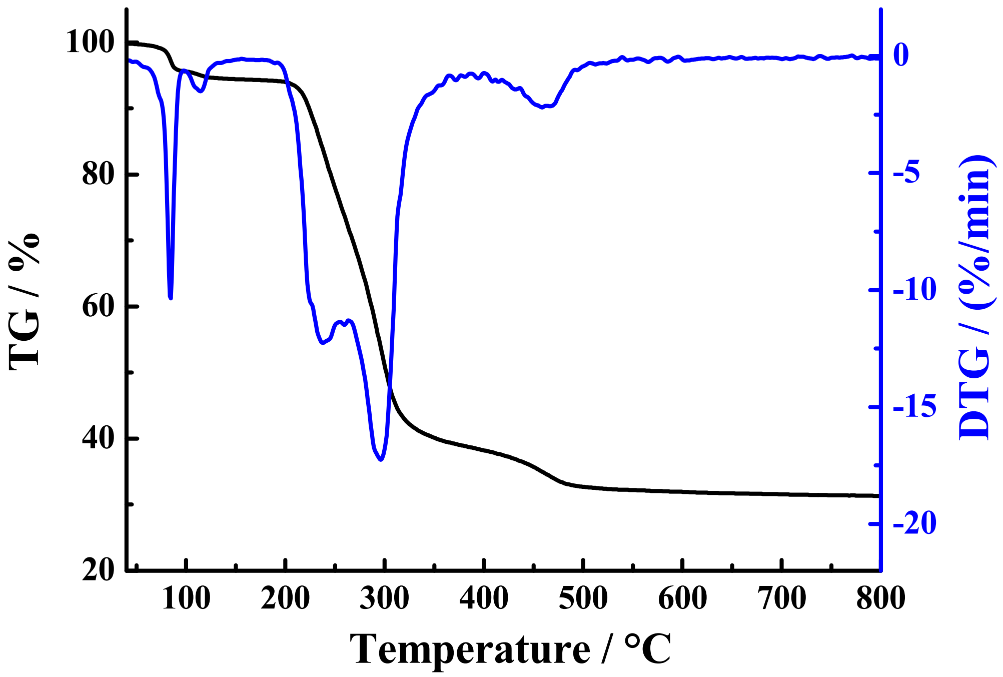

Figure S101 The TG-DTG curve of **1b**

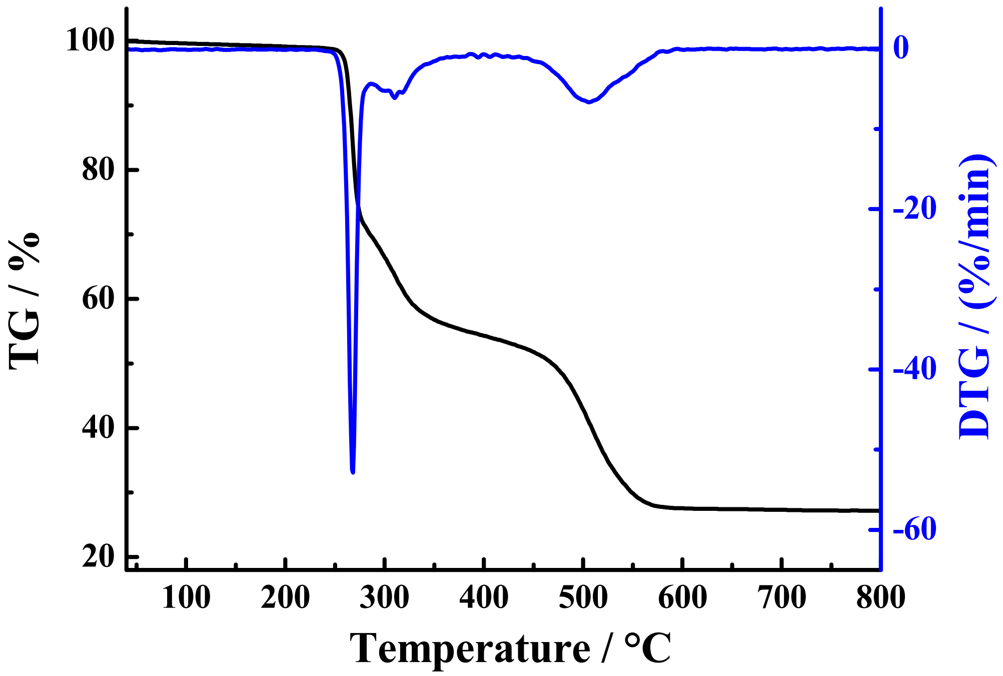

Figure S102 The TG-DTG curve of **2a**

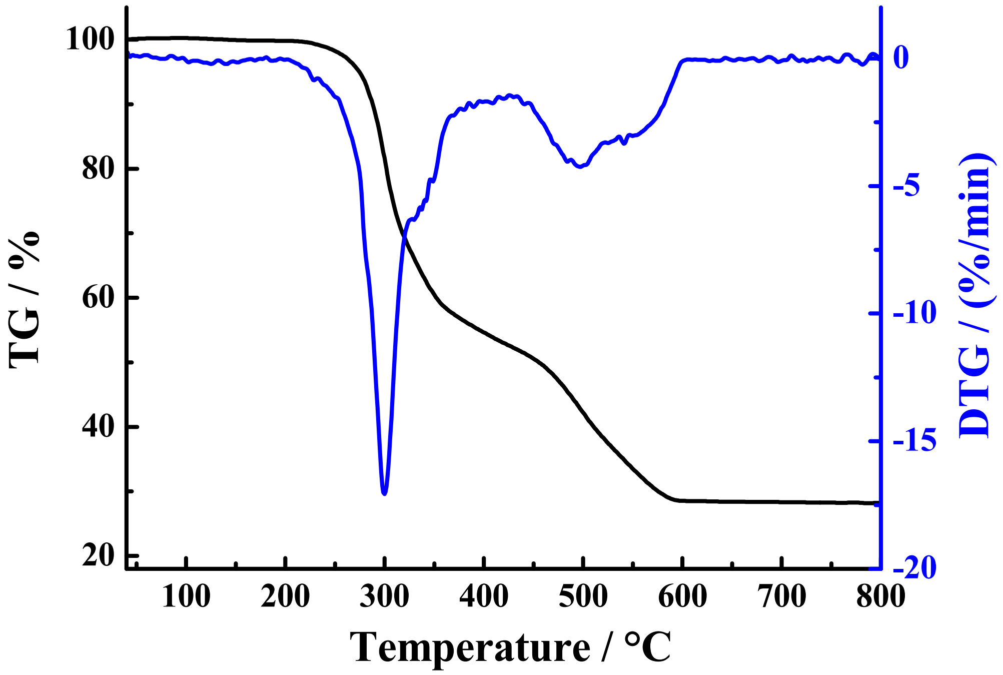

Figure S103 The TG-DTG curve of **2b**

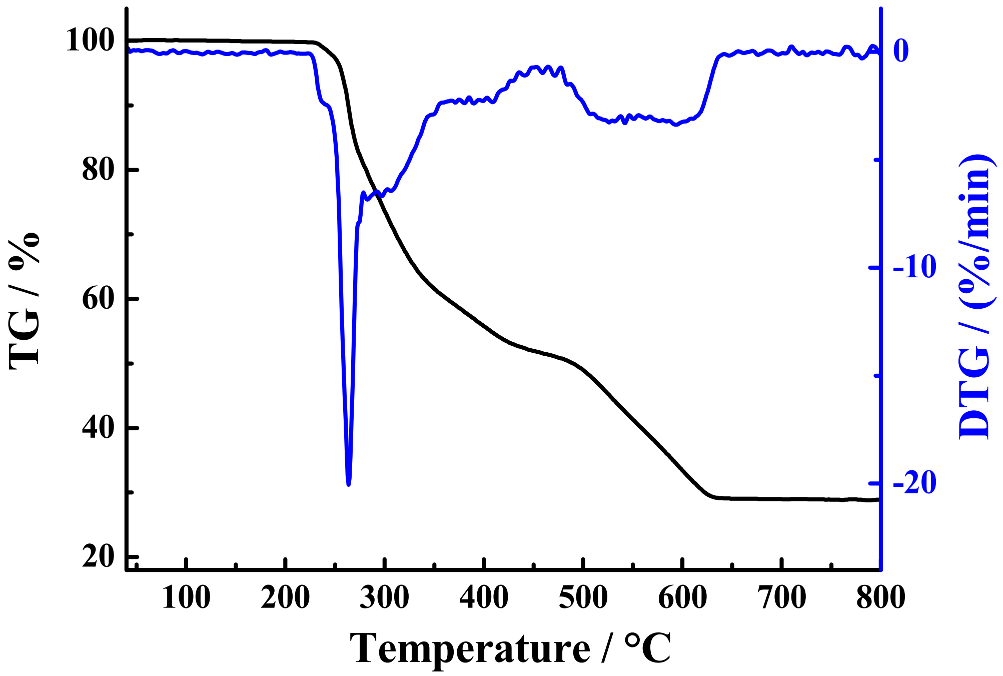

Figure S104 The TG-DTG curve of **3a**

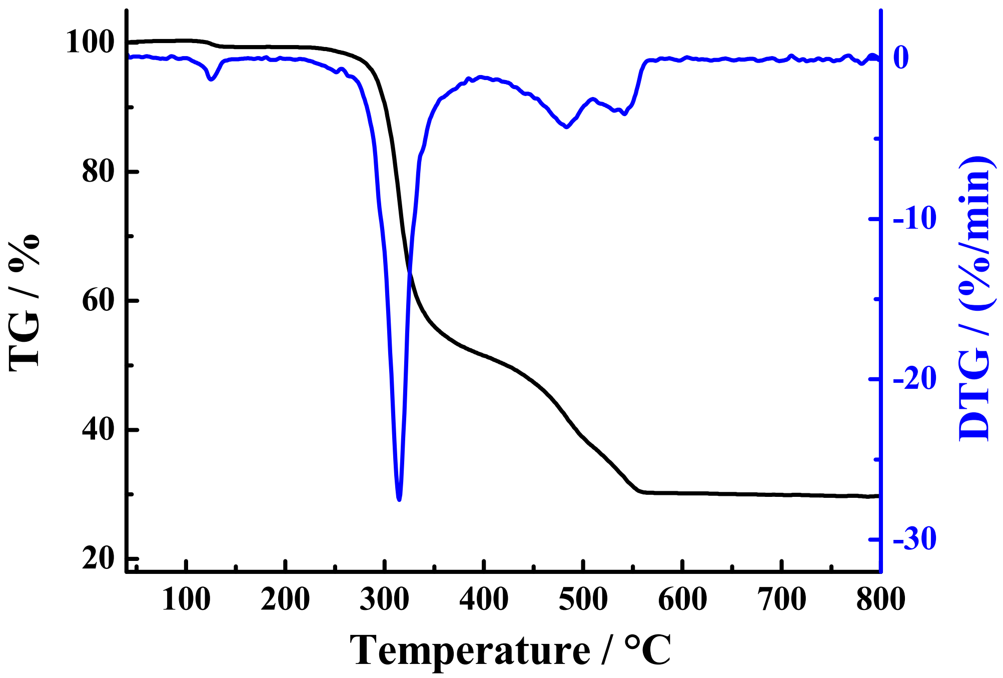

Figure S105 The TG-DTG curve of **3b**

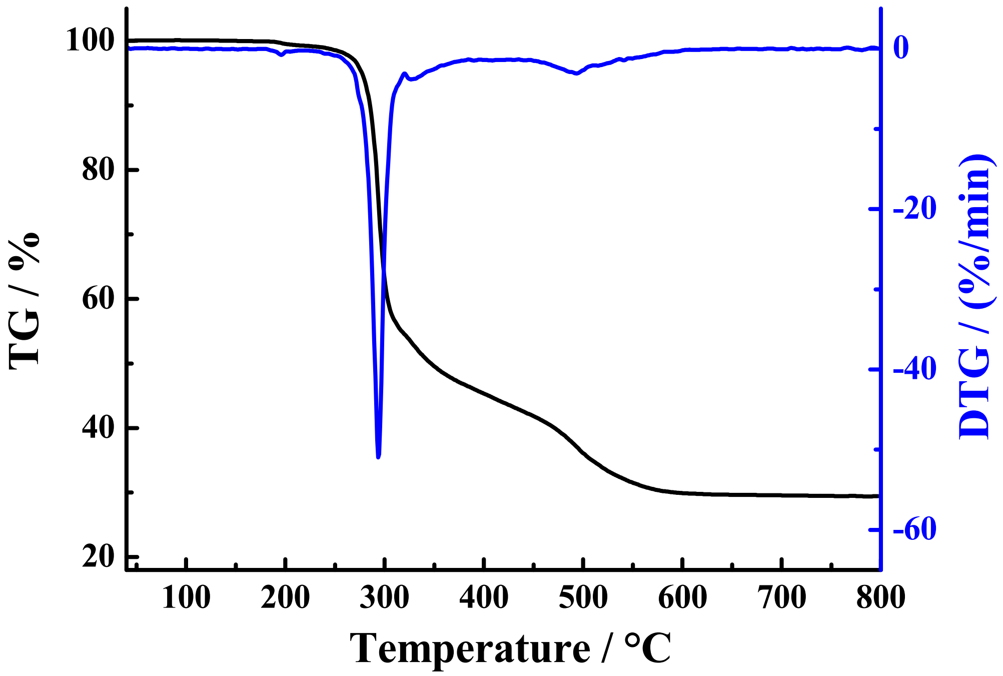

Figure S106 The TG-DTG curve of **4a**

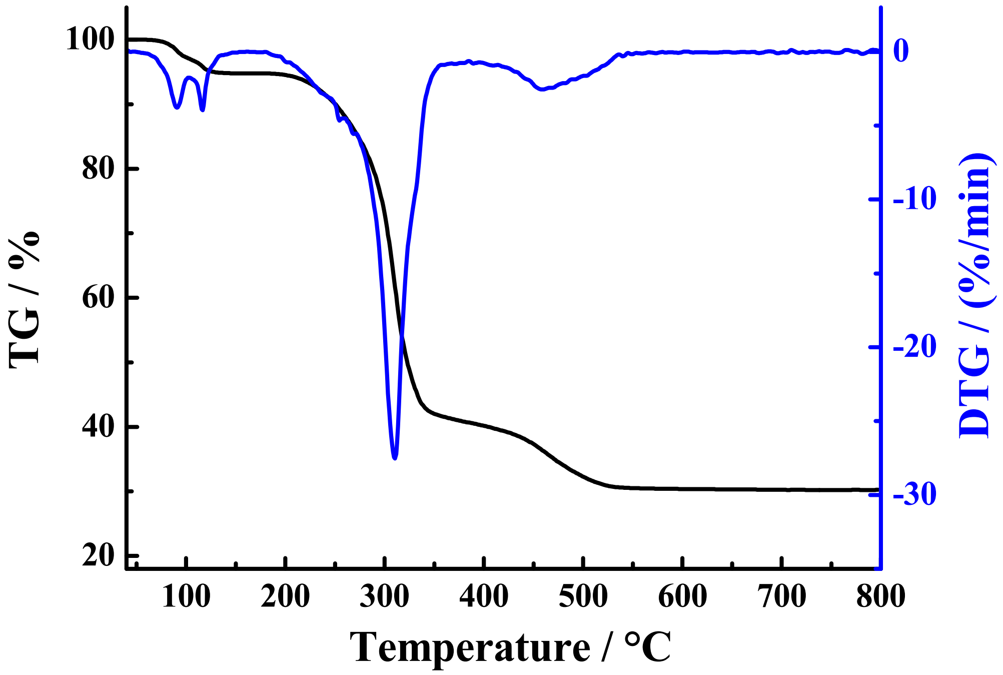

Figure S107 The TG-DTG curve of **4b**

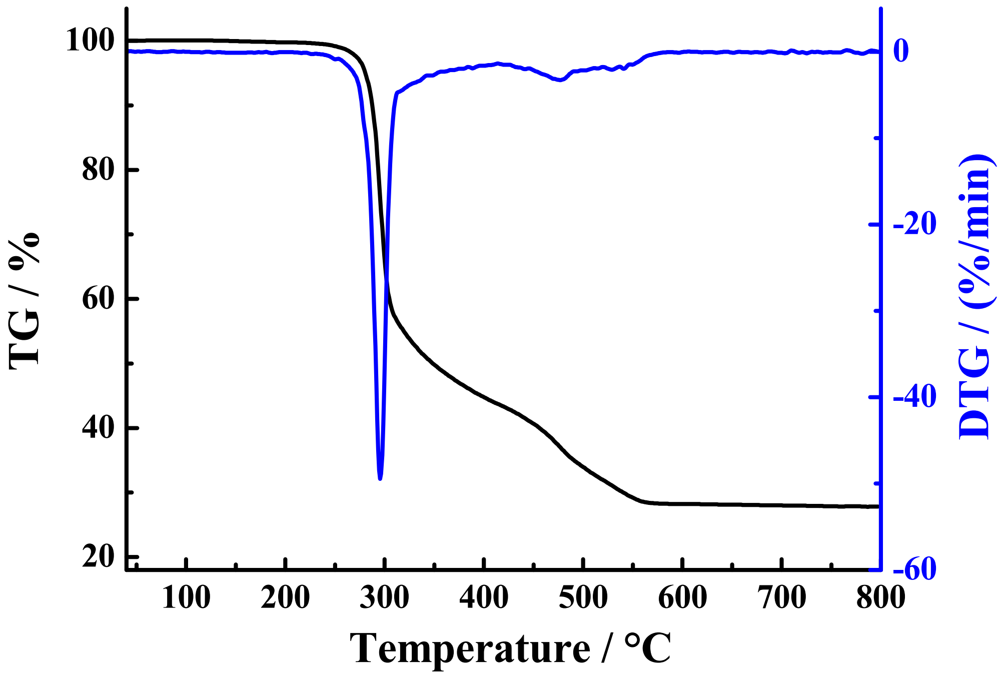

Figure S108 The TG-DTG curve of **5a**

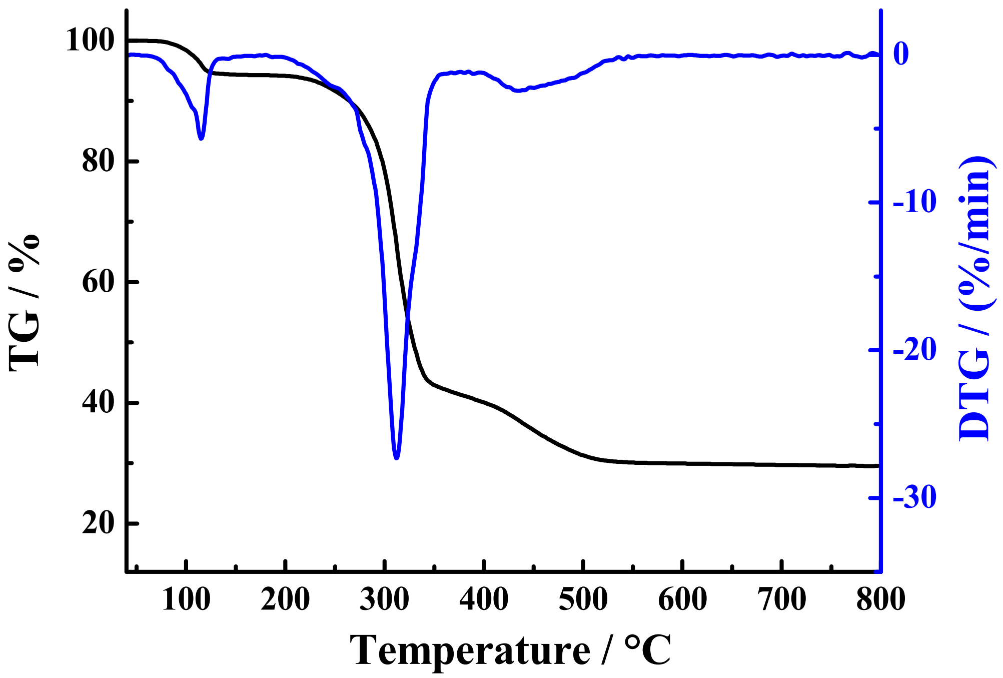

Figure S109 The TG-DTG curve of **5b**

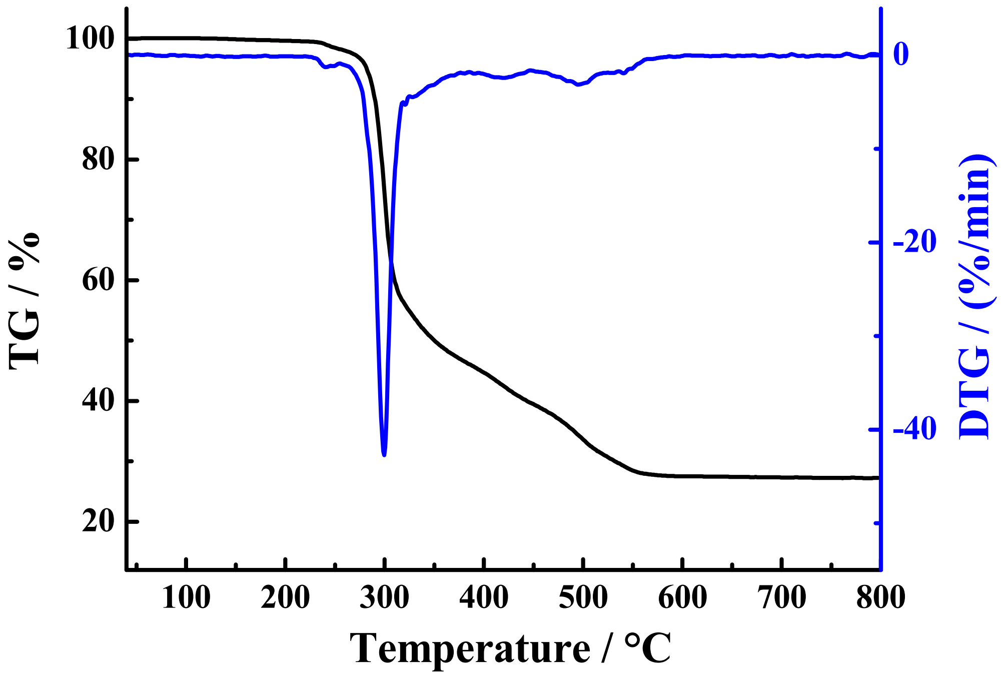

Figure S110 The TG-DTG curve of **6a**

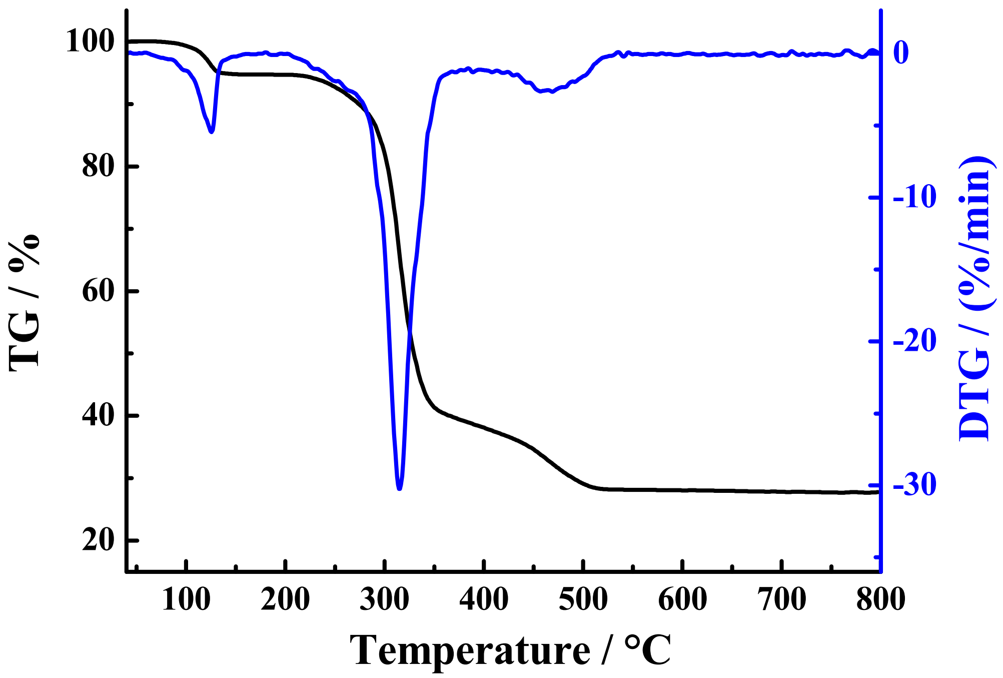

Figure S111 The TG-DTG curve of **6b**

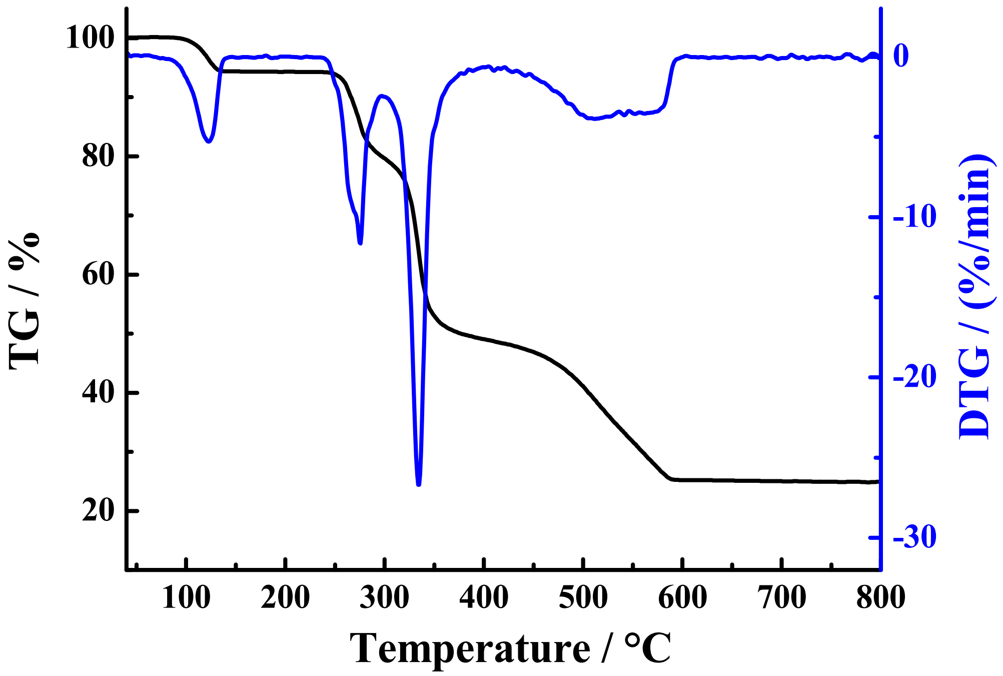

Figure S112 The TG-DTG curve of **7a**

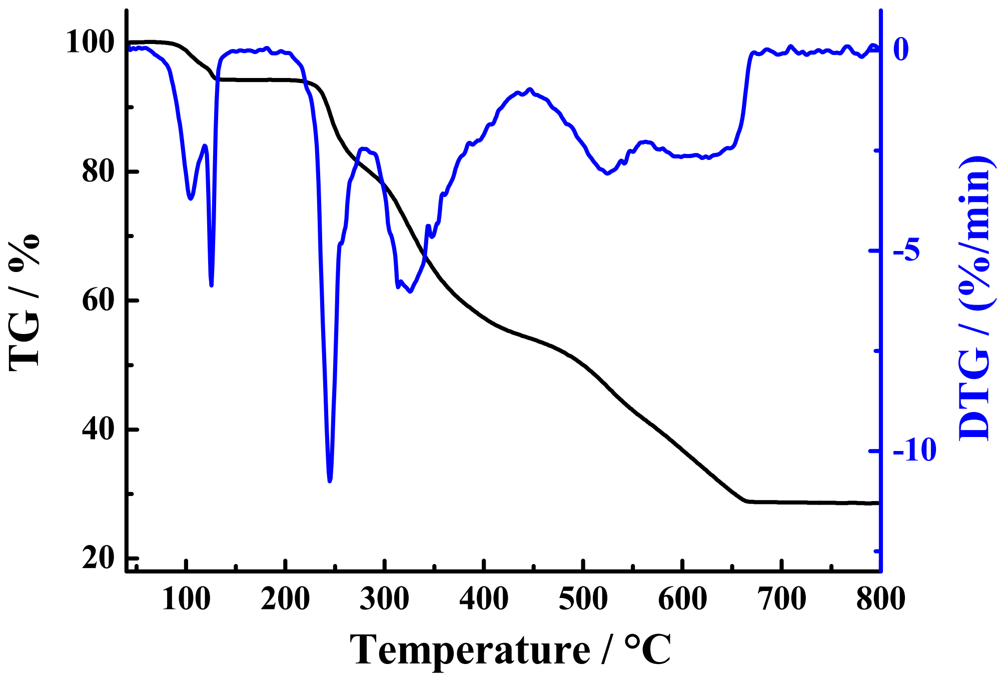

Figure S113 The TG-DTG curve of **7b**

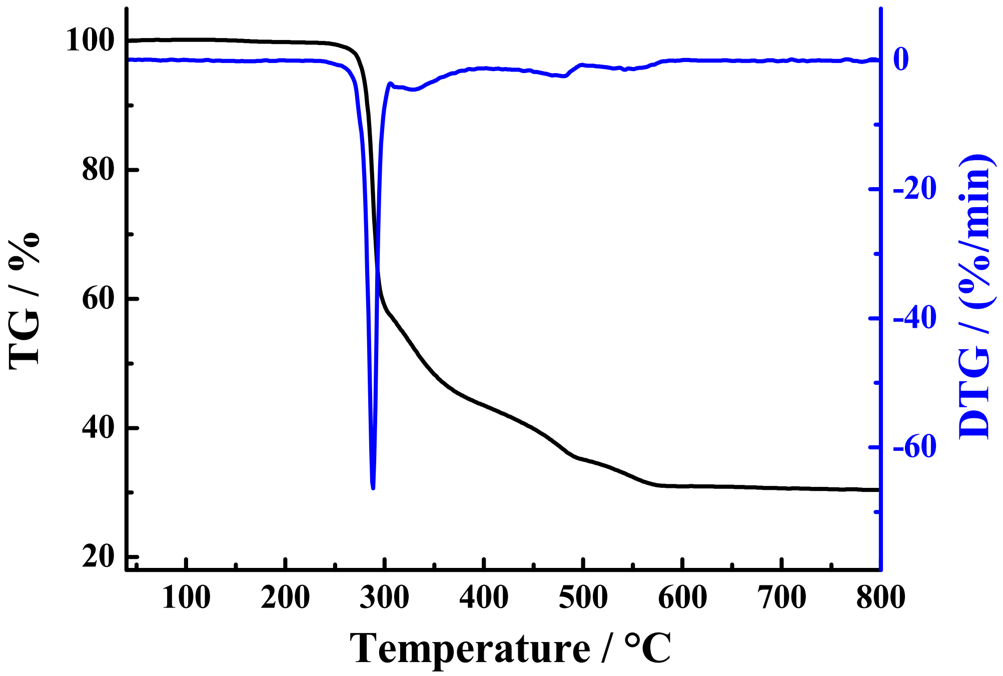

Figure S114 The TG-DTG curve of **8a**

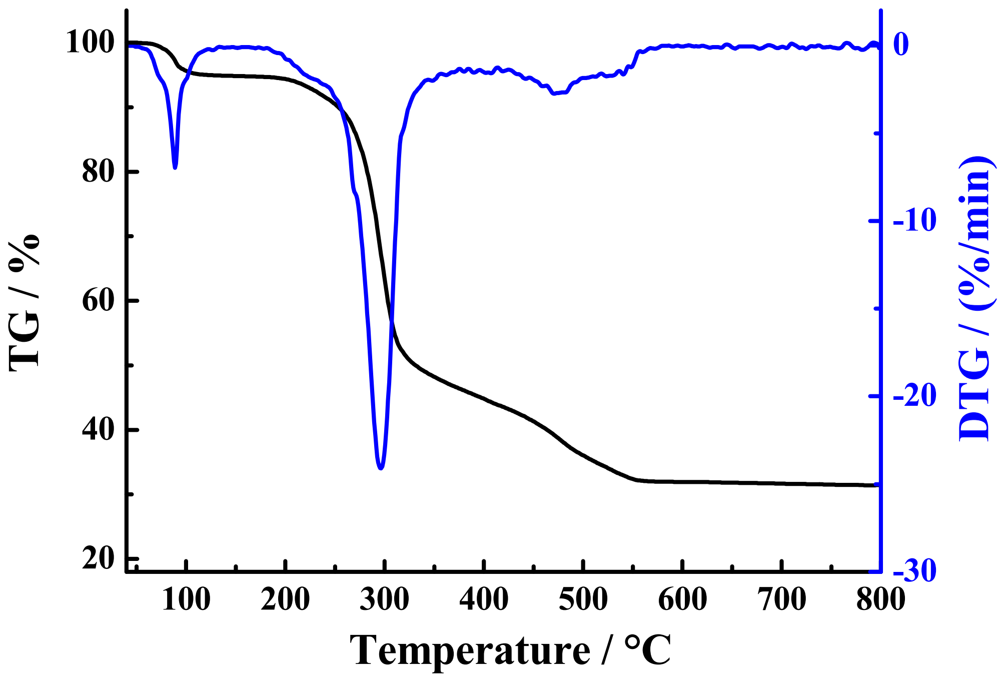

Figure S115 The TG-DTG curve of **8b**

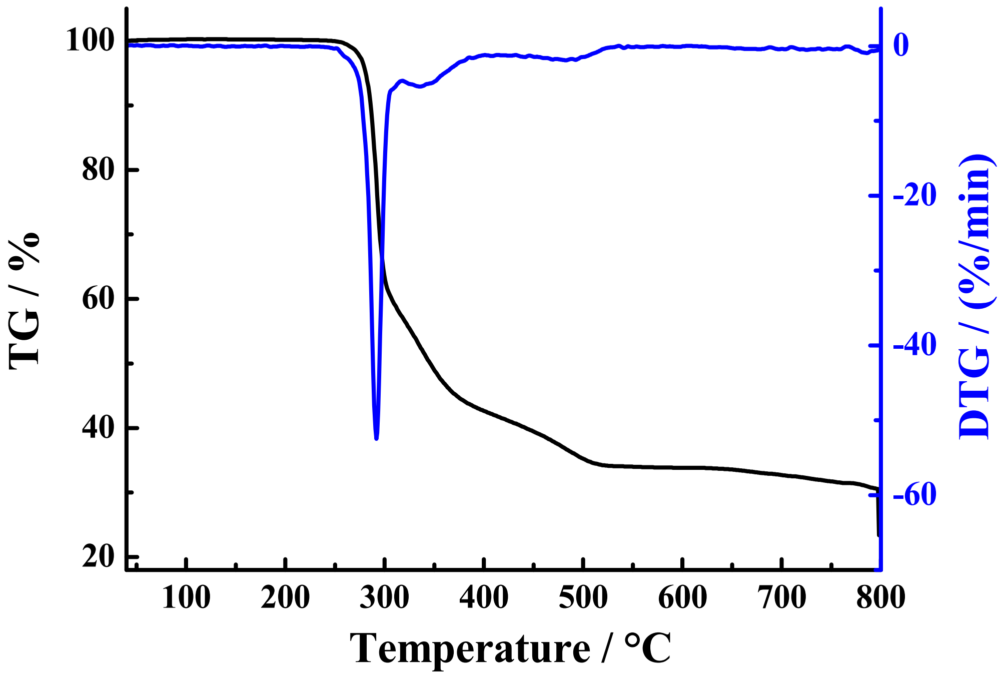

Figure S116 The TG-DTG curve of **9a**

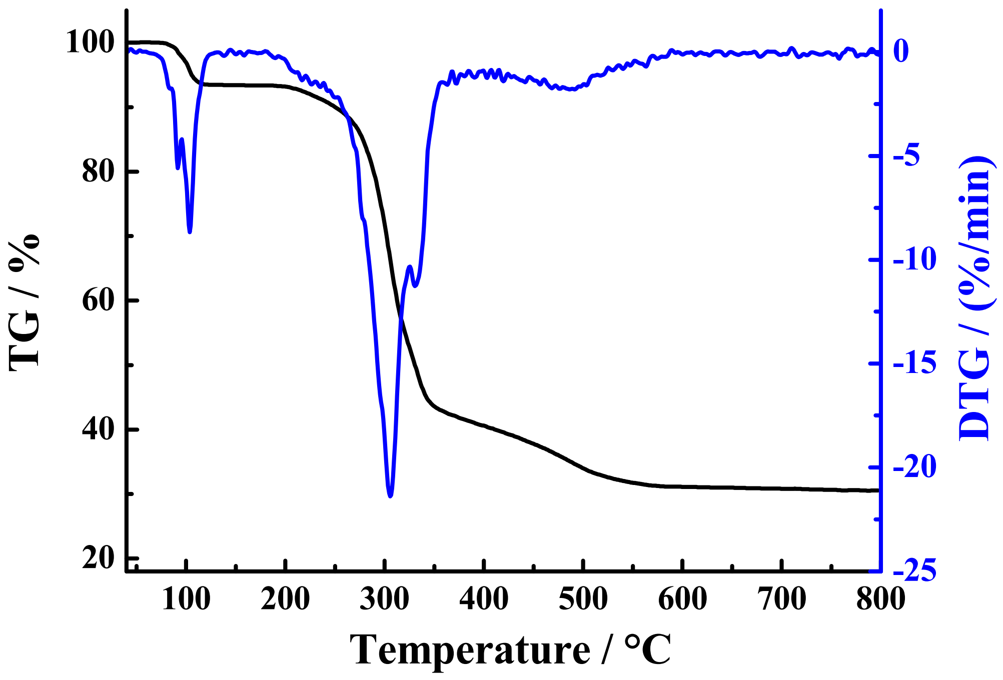

Figure S117 The TG-DTG curve of **9b**

5 Cell

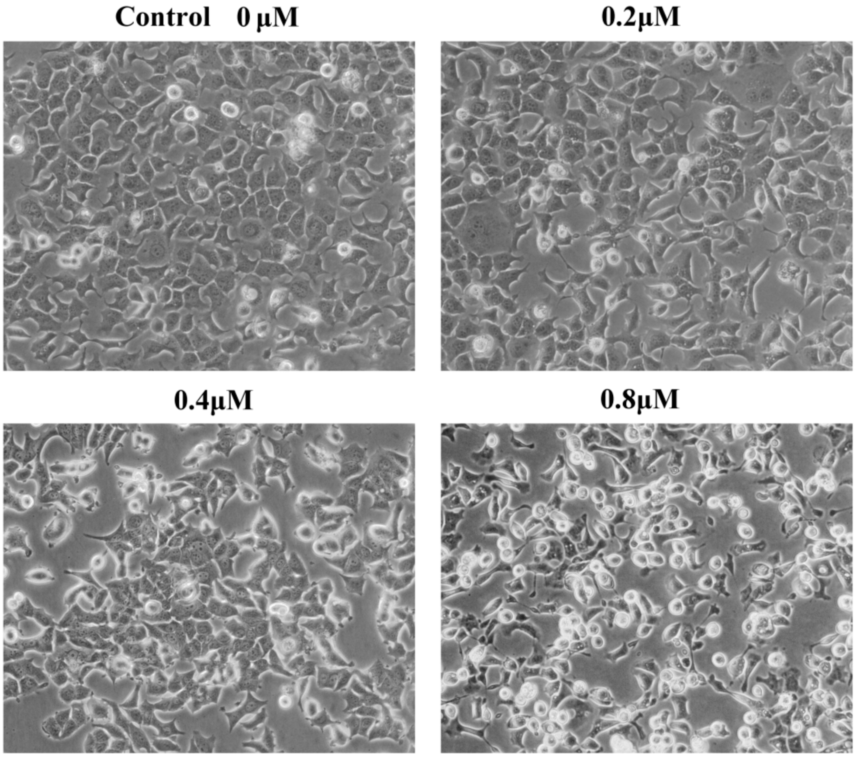

Figure S118. **4b** altered the morphology and growth status of MCF-7 cells. MCF-7 cells were observed under light microscopy after treatment with serial concentrations of at 24 hours.

6 Docking

Table S6 Calculated London dG scoring of the **4b** docking with DNA.

Ligand	London dG (kcal/mol)	Metal contact interactions residues	Distance(Å)
**C5**	-13.8816	DC *A9*	2.605

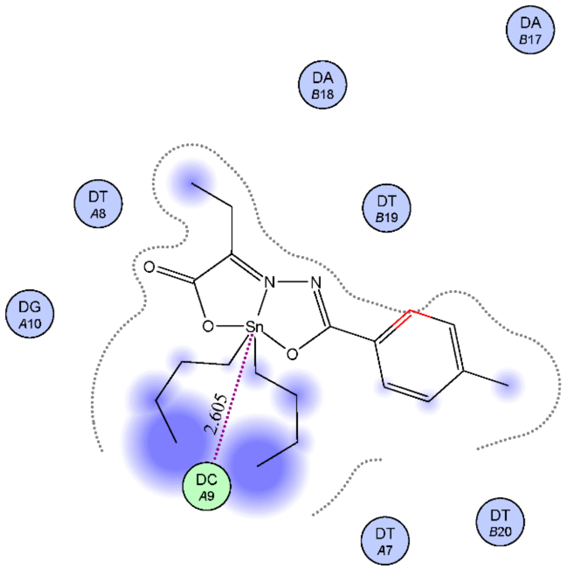

Figure S119 The 2D view of the interaction modes of **4b** and DNA.
