# Supplementary material for: Aroylhydrazone Diorganotin Complexes Causes DNA Damage and Apoptotic Cell Death: From Chemical Synthesis to Biochemical Effects
Source: Int J Mol Sci. 2021 Dec 16;22(24):13525. doi: 10.3390/ijms222413525 (PMC8709053; doi:10.3390/ijms222413525)

checkCIF/PLATON report

You have not supplied any structure factors. As a result the full set of tests cannot be run.

THIS REPORT IS FOR GUIDANCE ONLY. IF USED AS PART OF A REVIEW PROCEDURE FOR PUBLICATION, IT SHOULD NOT REPLACE THE EXPERTISE OF AN EXPERIENCED CRYSTALLOGRAPHIC REFEREE.

No syntax errors found. CIF dictionary Interpreting this report

Datablock: jwj0134

```
Bond precision:   C-C = 0.0080 A          Wavelength=0.71073

Cell:           a=10.637(3)         b=12.087(3)        c=12.177(3)
                alpha=100.935(3)    beta=108.490(3)    gamma=104.481(3)
Temperature:    296 K
```

	Calculated	Reported
Volume	1374.5(6)	1374.5(6)
Space group	P -1	P-1
Hall group	-P 1	?
Moiety formula	C24 H40 N2 O4 Sn	C24 H40 N2 O4 Sn
Sum formula	C24 H40 N2 O4 Sn	C24 H40 N2 O4 Sn
Mr	539.29	539.27
Dx,g cm-3	1.303	1.303
Z	2	2
Mu (mm-1)	0.957	0.957
F000	560.0	560.0
F000'	558.89	
h,k,lmax	12,14,14	12,14,14
Nref	4896	4865
Tmin,Tmax	0.818,0.826	0.824,0.832
Tmin'	0.818	

```
Correction method= # Reported T Limits: Tmin=0.824 Tmax=0.832
AbsCorr = MULTI-SCAN

Data completeness= 0.994            Theta(max)= 25.100

R(reflections)= 0.0378( 4198)                   wR2(reflections)=
                                                0.1177( 4865)
S = 1.071                  Npar= 372
```

The following ALERTS were generated. Each ALERT has the format
test-name_ALERT_alert-type_alert-level.
Click on the hyperlinks for more details of the test.

Alert level C

```
PLAT220_ALERT_2_C NonSolvent   Resd 1  C   Ueq(max)/Ueq(min) Range        4.3 Ratio
PLAT222_ALERT_3_C NonSolvent Resd 1  H   Uiso(max)/Uiso(min) Range        4.3 Ratio
PLAT234_ALERT_4_C Large Hirshfeld Difference Sn1A     --O1       .       0.18 Ang.
PLAT234_ALERT_4_C Large Hirshfeld Difference Sn1A     --C16      .       0.21 Ang.
PLAT234_ALERT_4_C Large Hirshfeld Difference Sn1A     --C20      .       0.18 Ang.
PLAT234_ALERT_4_C Large Hirshfeld Difference C12      --C14A     .       0.19 Ang.
PLAT241_ALERT_2_C High   'MainMol' Ueq as Compared to Neighbors of         O4 Check
PLAT242_ALERT_2_C Low    'MainMol' Ueq as Compared to Neighbors of         N1 Check
PLAT242_ALERT_2_C Low    'MainMol' Ueq as Compared to Neighbors of         C4 Check
PLAT242_ALERT_2_C Low    'MainMol' Ueq as Compared to Neighbors of        C12 Check
PLAT242_ALERT_2_C Low    'MainMol' Ueq as Compared to Neighbors of        C16 Check
PLAT242_ALERT_2_C Low    'MainMol' Ueq as Compared to Neighbors of        C20 Check
PLAT242_ALERT_2_C Low    'MainMol' Ueq as Compared to Neighbors of        C22 Check
PLAT260_ALERT_2_C Large Average Ueq of Residue Including       Sn1      0.131 Check
```

Alert level G

```
PLAT002_ALERT_2_G Number of Distance or Angle Restraints on AtSite         19 Note
PLAT003_ALERT_2_G Number of Uiso or Uij Restrained non-H Atoms ...         17 Report
PLAT005_ALERT_5_G No Embedded Refinement Details Found  in the CIF     Please Do !
PLAT154_ALERT_1_G The s.u.'s on the Cell Angles are Equal ..(Note)      0.003 Degree
PLAT232_ALERT_2_G Hirshfeld Test Diff (M-X)  Sn1      --O2       .        5.0 s.u.
PLAT232_ALERT_2_G Hirshfeld Test Diff (M-X)  Sn1      --O4       .        5.3 s.u.
PLAT232_ALERT_2_G Hirshfeld Test Diff (M-X)  Sn1A     --O2       .       12.0 s.u.
PLAT232_ALERT_2_G Hirshfeld Test Diff (M-X)  Sn1A     --O4       .       17.8 s.u.
PLAT232_ALERT_2_G Hirshfeld Test Diff (M-X)  Sn1A     --N1       .       11.5 s.u.
PLAT301_ALERT_3_G Main Residue  Disorder .............(Resd  1  )         29% Note
PLAT410_ALERT_2_G Short Intra H...H Contact  H16A     ..H18B     .       2.05 Ang.
                                                      x,y,z  =      1_555 Check
PLAT412_ALERT_2_G Short Intra XH3 .. XHn     H3       ..H13D     .       1.98 Ang.
                                                      x,y,z  =      1_555 Check
PLAT721_ALERT_1_G Bond    Calc     0.97000, Rep     0.96000 Dev...       0.01 Ang.
              C19      -H19C           1_555   1_555 ........     #   84 Check
PLAT779_ALERT_4_G Suspect or Irrelevant (Bond) Angle(s) in CIF ...      35.10 Deg.
              C10  -C8   -C10A    1_555   1_555   1_555 ......     #   45 Check
PLAT779_ALERT_4_G Suspect or Irrelevant (Bond) Angle(s) in CIF ...       6.70 Deg.
              SN1  -C20  -SN1A    1_555   1_555   1_555 ......     #   51 Check
PLAT779_ALERT_4_G Suspect or Irrelevant (Bond) Angle(s) in CIF ...       6.00 Deg.
              SN1  -C16  -SN1A    1_555   1_555   1_555 ......     #   61 Check
PLAT779_ALERT_4_G Suspect or Irrelevant (Bond) Angle(s) in CIF ...       2.70 Deg.
              SN1A -N1   -SN1     1_555   1_555   1_555 ......     #   99 Check
PLAT779_ALERT_4_G Suspect or Irrelevant (Bond) Angle(s) in CIF ...       6.50 Deg.
              SN1  -O1   -SN1A    1_555   1_555   1_555 ......     #  103 Check
PLAT779_ALERT_4_G Suspect or Irrelevant (Bond) Angle(s) in CIF ...      34.40 Deg.
              C14A -C12  -C15     1_555   1_555   1_555 ......     #  112 Check
PLAT779_ALERT_4_G Suspect or Irrelevant (Bond) Angle(s) in CIF ...      43.20 Deg.
              C13  -C12  -C15A    1_555   1_555   1_555 ......     #  113 Check
PLAT779_ALERT_4_G Suspect or Irrelevant (Bond) Angle(s) in CIF ...      40.00 Deg.
              C14  -C12  -C13A    1_555   1_555   1_555 ......     #  124 Check
PLAT789_ALERT_4_G Atoms with Negative _atom_site_disorder_group  #          1 Check
```

```
PLAT811_ALERT_5_G No ADDSYM Analysis: Too Many Excluded Atoms ....          ! Info
PLAT860_ALERT_3_G Number of Least-Squares Restraints ............         162 Note
PLAT899_ALERT_4_G SHELXL97  is Deprecated and Succeeded by SHELXL/       2018 Note
```

```
   0 ALERT level A = Most likely a serious problem - resolve or explain
   0 ALERT level B = A potentially serious problem, consider carefully
  14 ALERT level C = Check. Ensure it is not caused by an omission or oversight
  25 ALERT level G = General information/check it is not something unexpected

   2 ALERT type 1 CIF construction/syntax error, inconsistent or missing data
  18 ALERT type 2 Indicator that the structure model may be wrong or deficient
   3 ALERT type 3 Indicator that the structure quality may be low
  14 ALERT type 4 Improvement, methodology, query or suggestion
   2 ALERT type 5 Informative message, check
```

It is advisable to attempt to resolve as many as possible of the alerts in all categories. Often the minor alerts point to easily fixed oversights, errors and omissions in your CIF or refinement strategy, so attention to these fine details can be worthwhile. In order to resolve some of the more serious problems it may be necessary to carry out additional measurements or structure refinements. However, the purpose of your study may justify the reported deviations and the more serious of these should normally be commented upon in the discussion or experimental section of a paper or in the "special_details" fields of the CIF. checkCIF was carefully designed to identify outliers and unusual parameters, but every test has its limitations and alerts that are not important in a particular case may appear. Conversely, the absence of alerts does not guarantee there are no aspects of the results needing attention. It is up to the individual to critically assess their own results and, if necessary, seek expert advice.

Publication of your CIF in IUCr journals

A basic structural check has been run on your CIF. These basic checks will be run on all CIFs submitted for publication in IUCr journals (*Acta Crystallographica*, *Journal of Applied Crystallography*, *Journal of Synchrotron Radiation*); however, if you intend to submit to *Acta Crystallographica Section C* or *E* or *IUCrData*, you should make sure that full publication checks are run on the final version of your CIF prior to submission.

Publication of your CIF in other journals

Please refer to the *Notes for Authors* of the relevant journal for any special instructions relating to CIF submission.

PLATON version of 13/07/2021; check.def file version of 13/07/2021

Datablock jwj0134 - ellipsoid plot

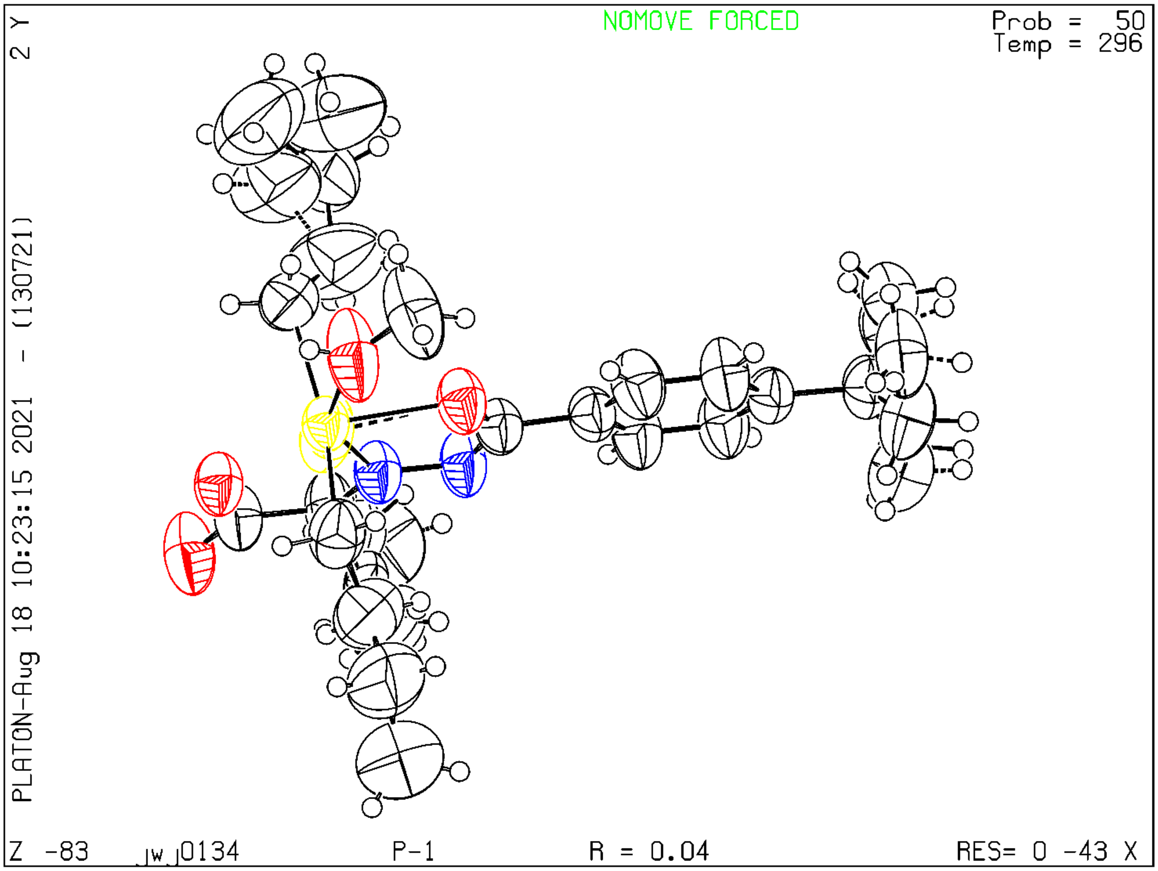

Supplement: Supplementary file 1 [file ijms-22-13525-s001.zip › CIF.zip!/6b-checkcif.pdf]
