# Supplementary material for: Aroylhydrazone Diorganotin Complexes Causes DNA Damage and Apoptotic Cell Death: From Chemical Synthesis to Biochemical Effects
Source: Int J Mol Sci. 2021 Dec 16;22(24):13525. doi: 10.3390/ijms222413525 (PMC8709053; doi:10.3390/ijms222413525)

checkCIF/PLATON report

You have not supplied any structure factors. As a result the full set of tests cannot be run.

THIS REPORT IS FOR GUIDANCE ONLY. IF USED AS PART OF A REVIEW PROCEDURE FOR PUBLICATION, IT SHOULD NOT REPLACE THE EXPERTISE OF AN EXPERIENCED CRYSTALLOGRAPHIC REFEREE.

No syntax errors found.   CIF dictionary   Interpreting this report

Datablock: jwj0153

Bond precision: C-C = 0.0042 A   Wavelength=0.71073

Cell:	a=12.375(6)	b=11.479(6)	c=16.659(8)
	alpha=90	beta=100.266(7)	gamma=90
Temperature:	296 K		

	Calculated	Reported
Volume	2329(2)	2328(2)
Space group	P 21/c	P2(1)/c
Hall group	-P 2ybc	?
Moiety formula	C48 H46 N6 O12 Sn2	C48 H46 N6 O12 Sn2
Sum formula	C48 H46 N6 O12 Sn2	C48 H46 N6 O12 Sn2
Mr	1136.33	1136.29
Dx,g cm-3	1.620	1.621
Z	2	2
Mu (mm-1)	1.143	1.143
F000	1144.0	1144.0
F000'	1141.87	
h,k,lmax	14,13,19	14,13,19
Nref	4145	4126
Tmin,Tmax	0.778,0.796	0.787,0.804
Tmin'	0.778	

Correction method= # Reported T Limits: Tmin=0.787 Tmax=0.804
AbsCorr = MULTI-SCAN

Data completeness= 0.995   Theta(max)= 25.100

R(reflections)= 0.0245(3623)   wR2(reflections)= 0.0641(4126)

S = 1.039   Npar= 313

The following ALERTS were generated. Each ALERT has the format

test-name_ALERT_alert-type_alert-level.

Click on the hyperlinks for more details of the test.

Alert level G

```
PLAT002_ALERT_2_G Number of Distance or Angle Restraints on AtSite         10 Note
PLAT005_ALERT_5_G No Embedded Refinement Details Found  in the CIF     Please Do !
PLAT710_ALERT_4_G Delete 1-2-3 or 2-3-4 Linear Torsion Angle ... #         15 Do !
              C12 -SN1 -C18 -C23    17.90  0.80   1_555   1_555   1_555   1_555
PLAT710_ALERT_4_G Delete 1-2-3 or 2-3-4 Linear Torsion Angle ... #         20 Do !
              C12 -SN1 -C18 -C19  -164.60  0.50   1_555   1_555   1_555   1_555
PLAT710_ALERT_4_G Delete 1-2-3 or 2-3-4 Linear Torsion Angle ... #         27 Do !
              C18 -SN1 -C12 -C17   162.50  0.50   1_555   1_555   1_555   1_555
PLAT710_ALERT_4_G Delete 1-2-3 or 2-3-4 Linear Torsion Angle ... #         32 Do !
              C18 -SN1 -C12 -C13   -20.80  0.80   1_555   1_555   1_555   1_555
PLAT860_ALERT_3_G Number of Least-Squares Restraints ............           7 Note
PLAT899_ALERT_4_G SHELXL97  is Deprecated and Succeeded by SHELXL/       2018 Note
```

0 **ALERT level A** = Most likely a serious problem - resolve or explain
0 **ALERT level B** = A potentially serious problem, consider carefully
0 **ALERT level C** = Check. Ensure it is not caused by an omission or oversight
8 **ALERT level G** = General information/check it is not something unexpected

0 ALERT type 1 CIF construction/syntax error, inconsistent or missing data
1 ALERT type 2 Indicator that the structure model may be wrong or deficient
1 ALERT type 3 Indicator that the structure quality may be low
5 ALERT type 4 Improvement, methodology, query or suggestion
1 ALERT type 5 Informative message, check

It is advisable to attempt to resolve as many as possible of the alerts in all categories. Often the minor alerts point to easily fixed oversights, errors and omissions in your CIF or refinement strategy, so attention to these fine details can be worthwhile. In order to resolve some of the more serious problems it may be necessary to carry out additional measurements or structure refinements. However, the purpose of your study may justify the reported deviations and the more serious of these should normally be commented upon in the discussion or experimental section of a paper or in the "special_details" fields of the CIF. checkCIF was carefully designed to identify outliers and unusual parameters, but every test has its limitations and alerts that are not important in a particular case may appear. Conversely, the absence of alerts does not guarantee there are no aspects of the results needing attention. It is up to the individual to critically assess their own results and, if necessary, seek expert advice.

Publication of your CIF in IUCr journals

A basic structural check has been run on your CIF. These basic checks will be run on all CIFs submitted for publication in IUCr journals (*Acta Crystallographica*, *Journal of Applied Crystallography*, *Journal of Synchrotron Radiation*); however, if you intend to submit to *Acta Crystallographica Section C* or *E* or *IUCrData*, you should make sure that full publication checks are run on the final version of your CIF prior to submission.

Publication of your CIF in other journals

Please refer to the *Notes for Authors* of the relevant journal for any special instructions relating to CIF submission.

PLATON version of 13/07/2021; check.def file version of 13/07/2021

Datablock jwj0153 - ellipsoid plot

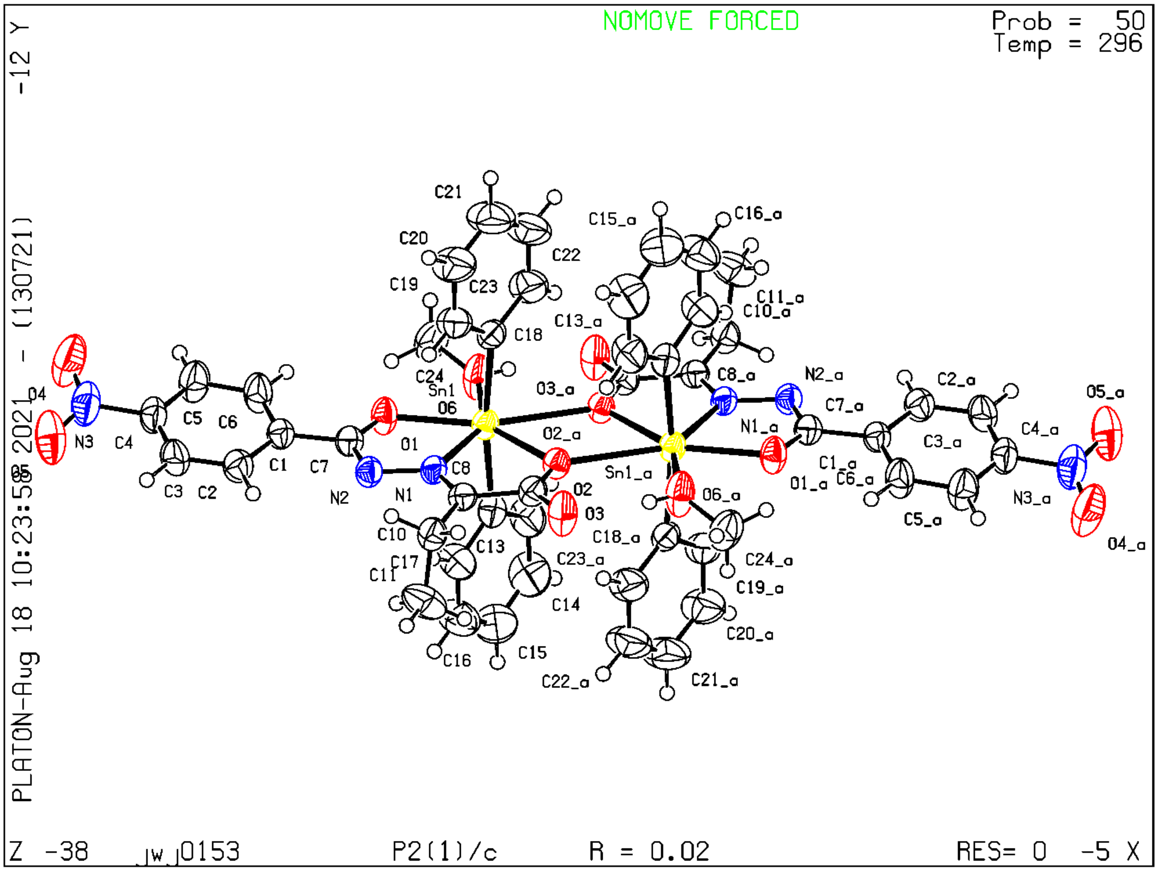

Supplement: Supplementary file 1 [file ijms-22-13525-s001.zip › CIF.zip!/7a-checkcif.pdf]
