# Supplementary material for: Aroylhydrazone Diorganotin Complexes Causes DNA Damage and Apoptotic Cell Death: From Chemical Synthesis to Biochemical Effects
Source: Int J Mol Sci. 2021 Dec 16;22(24):13525. doi: 10.3390/ijms222413525 (PMC8709053; doi:10.3390/ijms222413525)

checkCIF/PLATON report

You have not supplied any structure factors. As a result the full set of tests cannot be run.

THIS REPORT IS FOR GUIDANCE ONLY. IF USED AS PART OF A REVIEW PROCEDURE FOR PUBLICATION, IT SHOULD NOT REPLACE THE EXPERTISE OF AN EXPERIENCED CRYSTALLOGRAPHIC REFEREE.

No syntax errors found.   CIF dictionary   Interpreting this report

Datablock: jwj0129

```
Bond precision:   C-C = 0.0086 A          Wavelength=0.71073

Cell:             a=25.542(10)      b=25.542(10)      c=15.019(7)
                  alpha=90          beta=90           gamma=90
Temperature:      296 K

                 Calculated                   Reported
Volume           9798(9)                      9798(7)
Space group      I 41/a                       I4(1)/a
Hall group       -I 4ad                       ?
Moiety formula   C40 H62 N6 O12 Sn2           C40 H62 N6 O12 Sn2
Sum formula      C40 H62 N6 O12 Sn2           C40 H62 N6 O12 Sn2
Mr               1056.38                      1056.34
Dx,g cm-3        1.432                        1.432
Z                8                            8
Mu (mm-1)        1.080                        1.080
F000             4320.0                       4320.0
F000'            4311.32
h,k,lmax         30,30,17                     30,30,17
Nref             4366                         4361
Tmin,Tmax        0.780,0.789                  0.789,0.797
Tmin'            0.780

Correction method= # Reported T Limits: Tmin=0.789 Tmax=0.797
AbsCorr = MULTI-SCAN

Data completeness= 0.999           Theta(max)= 25.100

R(reflections)= 0.0437( 3702)                       wR2(reflections)=
                                                    0.1400( 4361)
S = 1.058                    Npar= 279
```

The following ALERTS were generated. Each ALERT has the format
test-name_ALERT_alert-type_alert-level.
Click on the hyperlinks for more details of the test.

Alert level C

```
PLAT220_ALERT_2_C NonSolvent   Resd 1  C   Ueq(max)/Ueq(min) Range        4.4 Ratio
PLAT222_ALERT_3_C NonSolvent Resd 1  H   Uiso(max)/Uiso(min) Range        4.6 Ratio
PLAT232_ALERT_2_C Hirshfeld Test Diff (M-X)  Sn1      --C12      .        5.5 s.u.
PLAT234_ALERT_4_C Large Hirshfeld Difference C18      --C19      .       0.20 Ang.
PLAT241_ALERT_2_C High   'MainMol' Ueq as Compared to Neighbors of         O6 Check
PLAT242_ALERT_2_C Low    'MainMol' Ueq as Compared to Neighbors of        Sn1 Check
PLAT242_ALERT_2_C Low    'MainMol' Ueq as Compared to Neighbors of        C18 Check
PLAT342_ALERT_3_C Low Bond Precision on  C-C Bonds ..............    0.00856 Ang.
PLAT601_ALERT_2_C Unit Cell Contains Solvent Accessible VOIDS of .         78 Ang**3
```

Alert level G

```
PLAT002_ALERT_2_G Number of Distance or Angle Restraints on AtSite          9 Note
PLAT003_ALERT_2_G Number of Uiso or Uij Restrained non-H Atoms ...          4 Report
PLAT005_ALERT_5_G No Embedded Refinement Details Found  in the CIF     Please Do !
PLAT083_ALERT_2_G SHELXL Second Parameter in WGHT  Unusually Large      23.45 Why ?
PLAT152_ALERT_1_G The Supplied and Calc. Volume s.u. Differ by ...          2 Units
PLAT860_ALERT_3_G Number of Least-Squares Restraints ............          26 Note
PLAT899_ALERT_4_G SHELXL97  is Deprecated and Succeeded by SHELXL/       2018 Note
```

0 **ALERT level A** = Most likely a serious problem - resolve or explain
0 **ALERT level B** = A potentially serious problem, consider carefully
9 **ALERT level C** = Check. Ensure it is not caused by an omission or oversight
7 **ALERT level G** = General information/check it is not something unexpected

1 ALERT type 1 CIF construction/syntax error, inconsistent or missing data
9 ALERT type 2 Indicator that the structure model may be wrong or deficient
3 ALERT type 3 Indicator that the structure quality may be low
2 ALERT type 4 Improvement, methodology, query or suggestion
1 ALERT type 5 Informative message, check

It is advisable to attempt to resolve as many as possible of the alerts in all categories. Often the minor alerts point to easily fixed oversights, errors and omissions in your CIF or refinement strategy, so attention to these fine details can be worthwhile. In order to resolve some of the more serious problems it may be necessary to carry out additional measurements or structure refinements. However, the purpose of your study may justify the reported deviations and the more serious of these should normally be commented upon in the discussion or experimental section of a paper or in the "special_details" fields of the CIF. checkCIF was carefully designed to identify outliers and unusual parameters, but every test has its limitations and alerts that are not important in a particular case may appear. Conversely, the absence of alerts does not guarantee there are no aspects of the results needing attention. It is up to the individual to critically assess their own results and, if necessary, seek expert advice.

Publication of your CIF in IUCr journals

A basic structural check has been run on your CIF. These basic checks will be run on all CIFs submitted for publication in IUCr journals (*Acta Crystallographica*, *Journal of Applied Crystallography*, *Journal of Synchrotron Radiation*); however, if you intend to submit to *Acta Crystallographica Section C* or *E* or *IUCrData*, you should make sure that full publication checks are run on the final version of your CIF prior to submission.

Publication of your CIF in other journals

Please refer to the *Notes for Authors* of the relevant journal for any special instructions relating to CIF submission.

PLATON version of 13/07/2021; check.def file version of 13/07/2021

Datablock jwj0129 - ellipsoid plot

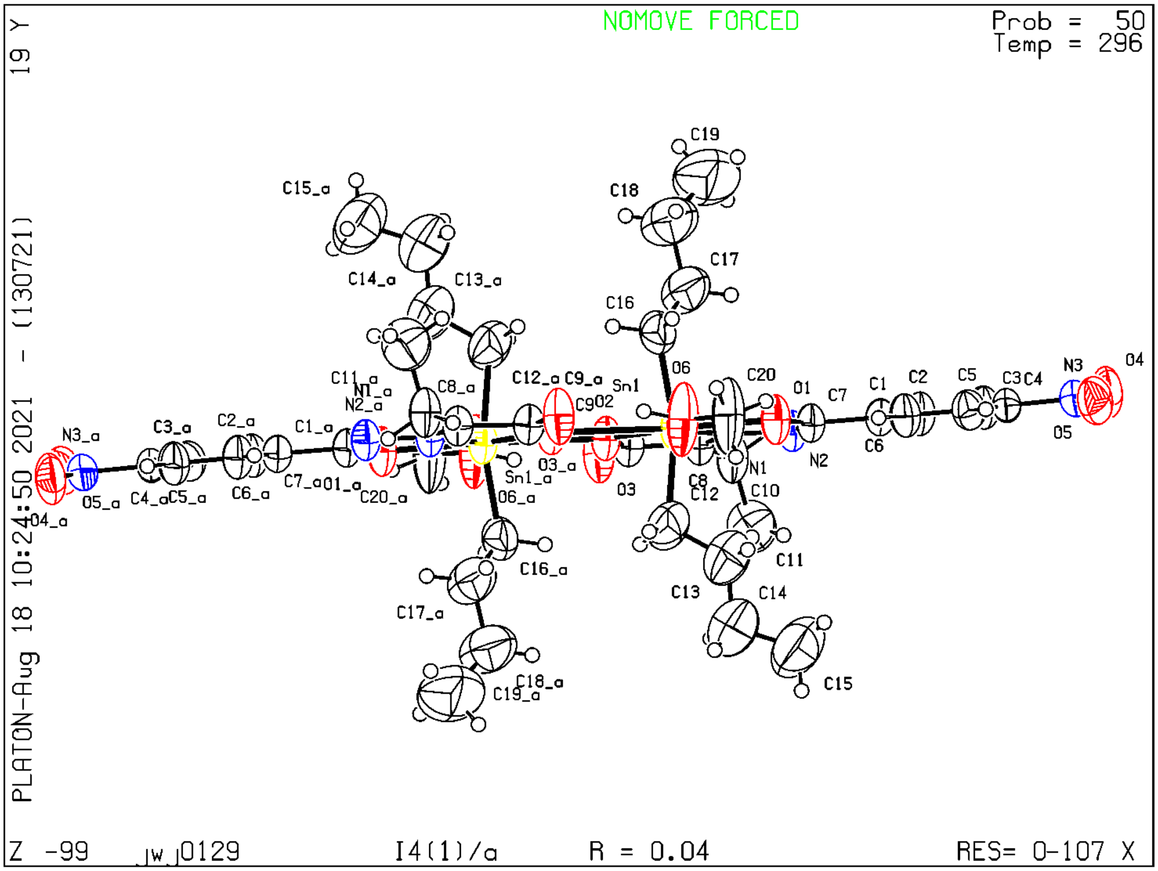

Supplement: Supplementary file 1 [file ijms-22-13525-s001.zip › CIF.zip!/7b-checkcif.pdf]
