# Supplementary material for: Aroylhydrazone Diorganotin Complexes Causes DNA Damage and Apoptotic Cell Death: From Chemical Synthesis to Biochemical Effects
Source: Int J Mol Sci. 2021 Dec 16;22(24):13525. doi: 10.3390/ijms222413525 (PMC8709053; doi:10.3390/ijms222413525)

checkCIF/PLATON report

You have not supplied any structure factors. As a result the full set of tests cannot be run.

THIS REPORT IS FOR GUIDANCE ONLY. IF USED AS PART OF A REVIEW PROCEDURE FOR PUBLICATION, IT SHOULD NOT REPLACE THE EXPERTISE OF AN EXPERIENCED CRYSTALLOGRAPHIC REFEREE.

No syntax errors found. CIF dictionary Interpreting this report

Datablock: jwj0170_a

```
Bond precision:   C-C = 0.0050 A          Wavelength=0.71073

Cell:             a=11.6256(6)     b=24.3106(13)       c=16.0727(8)
                  alpha=90         beta=107.884(1)     gamma=90
Temperature:      296 K

                 Calculated                  Reported
Volume           4323.1(4)                   4323.1(4)
Space group      P 21/c                      P2(1)/c
Hall group       -P 2ybc                     ?
Moiety formula   C18 H30 N2 O5 Sn            C18 H30 N2 O5 Sn
Sum formula      C18 H30 N2 O5 Sn            C18 H30 N2 O5 Sn
Mr               473.15                      473.13
Dx,g cm-3        1.454                       1.454
Z                8                           8
Mu (mm-1)        1.210                       1.210
F000             1936.0                      1936.0
F000'            1931.54
h,k,lmax         13,29,19                    13,29,19
Nref             7701                        7701
Tmin,Tmax        0.765,0.766                 0.768,0.777
Tmin'            0.750

Correction method= # Reported T Limits: Tmin=0.768 Tmax=0.777
AbsCorr = MULTI-SCAN

Data completeness= 1.000          Theta(max)= 25.100

R(reflections)= 0.0266( 6498)                wR2(reflections)=
                                             0.0652( 7701)
S = 1.044                 Npar= 485
```

The following ALERTS were generated. Each ALERT has the format
test-name_ALERT_alert-type_alert-level.
Click on the hyperlinks for more details of the test.

Alert level C

```
PLAT220_ALERT_2_C NonSolvent   Resd 1  C   Ueq(max)/Ueq(min) Range        3.3 Ratio
PLAT230_ALERT_2_C Hirshfeld Test Diff for    O4       --C1       .        6.5 s.u.
PLAT230_ALERT_2_C Hirshfeld Test Diff for    C3       --C4       .        5.2 s.u.
PLAT230_ALERT_2_C Hirshfeld Test Diff for    O9       --C19      .        7.0 s.u.
PLAT230_ALERT_2_C Hirshfeld Test Diff for    C19      --C20      .        5.5 s.u.
PLAT230_ALERT_2_C Hirshfeld Test Diff for    C20      --C21      .        6.0 s.u.
PLAT242_ALERT_2_C Low    'MainMol' Ueq as Compared to Neighbors of        C16 Check
```

Alert level G

```
PLAT003_ALERT_2_G Number of Uiso or Uij Restrained non-H Atoms ...          5 Report
PLAT005_ALERT_5_G No Embedded Refinement Details Found  in the CIF     Please Do !
PLAT398_ALERT_2_G Deviating  C-O-C    Angle From 120 for O4             105.7 Degree
PLAT398_ALERT_2_G Deviating  C-O-C    Angle From 120 for O9             104.6 Degree
PLAT789_ALERT_4_G Atoms with Negative _atom_site_disorder_group  #          2 Check
PLAT860_ALERT_3_G Number of Least-Squares Restraints ............          40 Note
PLAT899_ALERT_4_G SHELXL97  is Deprecated and Succeeded by SHELXL/        2018 Note
```

0 **ALERT level A** = Most likely a serious problem - resolve or explain
0 **ALERT level B** = A potentially serious problem, consider carefully
7 **ALERT level C** = Check. Ensure it is not caused by an omission or oversight
7 **ALERT level G** = General information/check it is not something unexpected

0 ALERT type 1 CIF construction/syntax error, inconsistent or missing data
10 ALERT type 2 Indicator that the structure model may be wrong or deficient
1 ALERT type 3 Indicator that the structure quality may be low
2 ALERT type 4 Improvement, methodology, query or suggestion
1 ALERT type 5 Informative message, check

It is advisable to attempt to resolve as many as possible of the alerts in all categories. Often the minor alerts point to easily fixed oversights, errors and omissions in your CIF or refinement strategy, so attention to these fine details can be worthwhile. In order to resolve some of the more serious problems it may be necessary to carry out additional measurements or structure refinements. However, the purpose of your study may justify the reported deviations and the more serious of these should normally be commented upon in the discussion or experimental section of a paper or in the "special_details" fields of the CIF. checkCIF was carefully designed to identify outliers and unusual parameters, but every test has its limitations and alerts that are not important in a particular case may appear. Conversely, the absence of alerts does not guarantee there are no aspects of the results needing attention. It is up to the individual to critically assess their own results and, if necessary, seek expert advice.

Publication of your CIF in IUCr journals

A basic structural check has been run on your CIF. These basic checks will be run on all CIFs submitted for publication in IUCr journals (*Acta Crystallographica*, *Journal of Applied Crystallography*, *Journal of Synchrotron Radiation*); however, if you intend to submit to *Acta Crystallographica Section C* or *E* or *IUCrData*, you should make sure that full publication checks are run on the final version of your CIF prior to submission.

Publication of your CIF in other journals

Please refer to the *Notes for Authors* of the relevant journal for any special instructions relating to CIF submission.

PLATON version of 13/07/2021; check.def file version of 13/07/2021

Datablock jwj0170_a - ellipsoid plot

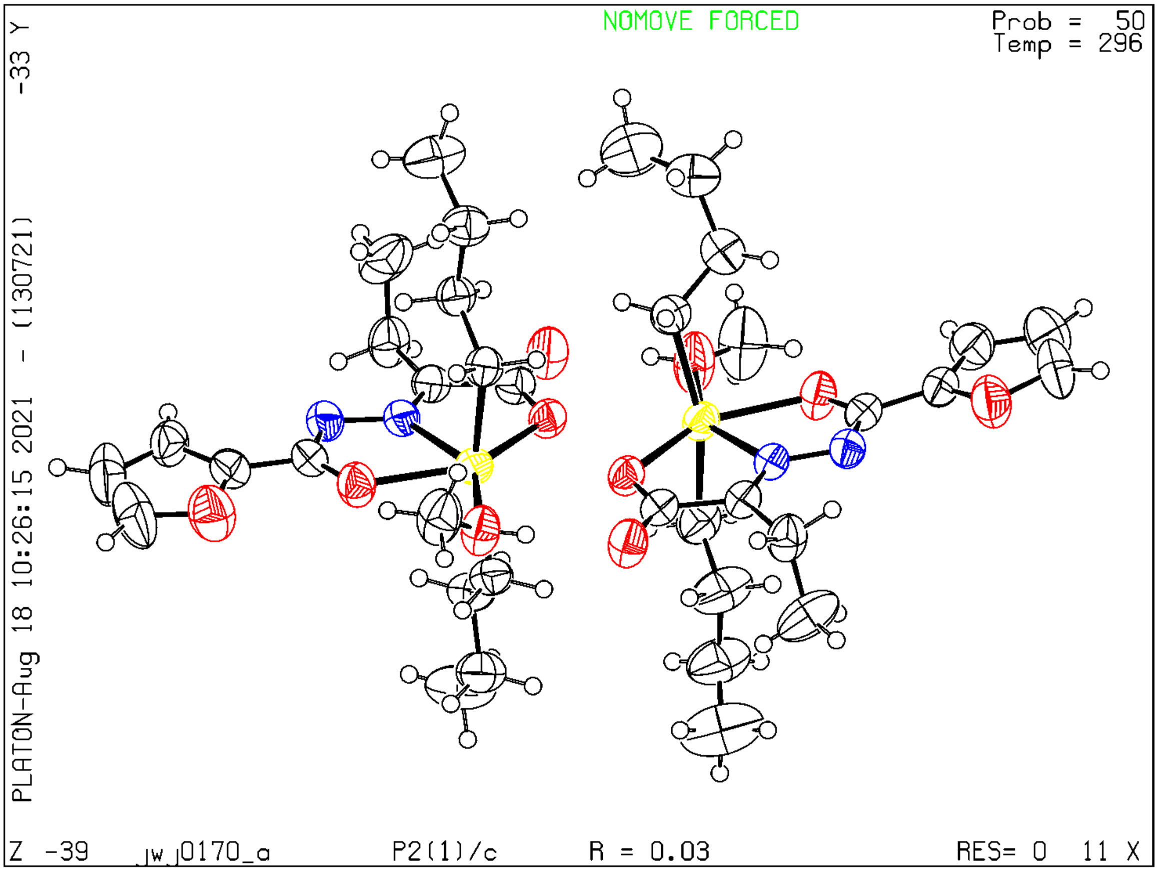

Supplement: Supplementary file 1 [file ijms-22-13525-s001.zip › CIF.zip!/8b-checkcif.pdf]
