# Supplementary material for: Aroylhydrazone Diorganotin Complexes Causes DNA Damage and Apoptotic Cell Death: From Chemical Synthesis to Biochemical Effects
Source: Int J Mol Sci. 2021 Dec 16;22(24):13525. doi: 10.3390/ijms222413525 (PMC8709053; doi:10.3390/ijms222413525)

checkCIF/PLATON report

You have not supplied any structure factors. As a result the full set of tests cannot be run.

THIS REPORT IS FOR GUIDANCE ONLY. IF USED AS PART OF A REVIEW PROCEDURE FOR PUBLICATION, IT SHOULD NOT REPLACE THE EXPERTISE OF AN EXPERIENCED CRYSTALLOGRAPHIC REFEREE.

No syntax errors found. CIF dictionary Interpreting this report

Datablock: jwj0171_a

Bond precision:	C-C = 0.0063 A		Wavelength=0.71073
Cell:	a=12.4982(8)	b=20.7695(14)	c=8.7242(6)
	alpha=90	beta=101.536(1)	gamma=90
Temperature:	296 K		

	Calculated	Reported
Volume	2218.9(3)	2218.9(3)
Space group	P 21/c	P2(1)/c
Hall group	-P 2ybc	?
Moiety formula	C18 H30 N2 O4 S Sn	C18 H30 N2 O4 S Sn
Sum formula	C18 H30 N2 O4 S Sn	C18 H30 N2 O4 S Sn
Mr	489.21	489.19
Dx,g cm-3	1.464	1.464
Z	4	4
Mu (mm-1)	1.269	1.269
F000	1000.0	1000.0
F000’	998.23	
h,k,lmax	14,24,10	14,24,10
Nref	3951	3939
Tmin,Tmax	0.754,0.756	0.759,0.768
Tmin’	0.739	

Correction method= # Reported T Limits: Tmin=0.759 Tmax=0.768
AbsCorr = MULTI-SCAN

Data completeness= 0.997 Theta(max)= 25.100

R(reflections)= 0.0307(3535) wR2(reflections)= 0.0774(3939)

S = 1.122 Npar= 273

The following ALERTS were generated. Each ALERT has the format
test-name_ALERT_alert-type_alert-level.
Click on the hyperlinks for more details of the test.

Alert level C

```
PLAT234_ALERT_4_C Large Hirshfeld Difference C1       --C2       .       0.19 Ang.
PLAT234_ALERT_4_C Large Hirshfeld Difference C12      --C13      .       0.18 Ang.
PLAT241_ALERT_2_C High   'MainMol' Ueq as Compared to Neighbors of         C4 Check
PLAT242_ALERT_2_C Low    'MainMol' Ueq as Compared to Neighbors of         C1 Check
```

Alert level G

```
PLAT002_ALERT_2_G Number of Distance or Angle Restraints on AtSite          8 Note
PLAT005_ALERT_5_G No Embedded Refinement Details Found  in the CIF     Please Do !
PLAT230_ALERT_2_G Hirshfeld Test Diff for    S1A      --C1       .        7.3 s.u.
PLAT230_ALERT_2_G Hirshfeld Test Diff for    C4       --C2A      .        5.2 s.u.
PLAT301_ALERT_3_G Main Residue  Disorder ..............(Resd  1  )        23% Note
PLAT773_ALERT_2_G Check long C-C Bond in CIF: C2       --C1              1.71 Ang.
PLAT773_ALERT_2_G Check long C-C Bond in CIF: C2A      --C4              1.71 Ang.
PLAT773_ALERT_2_G Check long C-C Bond in CIF: C2A      --C1              1.79 Ang.
PLAT779_ALERT_4_G Suspect or Irrelevant (Bond) Angle(s) in CIF ...       5.40 Deg.
              S1A  -C1   -C2      1_555   1_555   1_555 ......     #   52 Check
PLAT779_ALERT_4_G Suspect or Irrelevant (Bond) Angle(s) in CIF ...       6.60 Deg.
              S1   -C1   -C2A     1_555   1_555   1_555 ......     #   56 Check
PLAT779_ALERT_4_G Suspect or Irrelevant (Bond) Angle(s) in CIF ...       8.90 Deg.
              S1A  -C3   -C2      1_555   1_555   1_555 ......     #   84 Check
PLAT779_ALERT_4_G Suspect or Irrelevant (Bond) Angle(s) in CIF ...       8.40 Deg.
              S1   -C4   -C2A     1_555   1_555   1_555 ......     #   90 Check
PLAT789_ALERT_4_G Atoms with Negative _atom_site_disorder_group  #          1 Check
PLAT860_ALERT_3_G Number of Least-Squares Restraints .............          7 Note
PLAT899_ALERT_4_G SHELXL97  is Deprecated and Succeeded by SHELXL/       2018 Note
```

0 **ALERT level A** = Most likely a serious problem - resolve or explain
0 **ALERT level B** = A potentially serious problem, consider carefully
4 **ALERT level C** = Check. Ensure it is not caused by an omission or oversight
15 **ALERT level G** = General information/check it is not something unexpected

0 ALERT type 1 CIF construction/syntax error, inconsistent or missing data
8 ALERT type 2 Indicator that the structure model may be wrong or deficient
2 ALERT type 3 Indicator that the structure quality may be low
8 ALERT type 4 Improvement, methodology, query or suggestion
1 ALERT type 5 Informative message, check

It is advisable to attempt to resolve as many as possible of the alerts in all categories. Often the minor alerts point to easily fixed oversights, errors and omissions in your CIF or refinement strategy, so attention to these fine details can be worthwhile. In order to resolve some of the more serious problems it may be necessary to carry out additional measurements or structure refinements. However, the purpose of your study may justify the reported deviations and the more serious of these should normally be commented upon in the discussion or experimental section of a paper or in the "special_details" fields of the CIF. checkCIF was carefully designed to identify outliers and unusual parameters, but every test has its limitations and alerts that are not important in a particular case may appear. Conversely, the absence of alerts does not guarantee there are no aspects of the results needing attention. It is up to the individual to critically assess their own results and, if necessary, seek expert advice.

Publication of your CIF in IUCr journals

A basic structural check has been run on your CIF. These basic checks will be run on all CIFs submitted for publication in IUCr journals (*Acta Crystallographica*, *Journal of Applied Crystallography*, *Journal of Synchrotron Radiation*); however, if you intend to submit to *Acta Crystallographica Section C* or *E* or *IUCrData*, you should make sure that full publication checks are run on the final version of your CIF prior to submission.

Publication of your CIF in other journals

Please refer to the *Notes for Authors* of the relevant journal for any special instructions relating to CIF submission.

PLATON version of 13/07/2021; check.def file version of 13/07/2021

Datablock jwj0171_a - ellipsoid plot

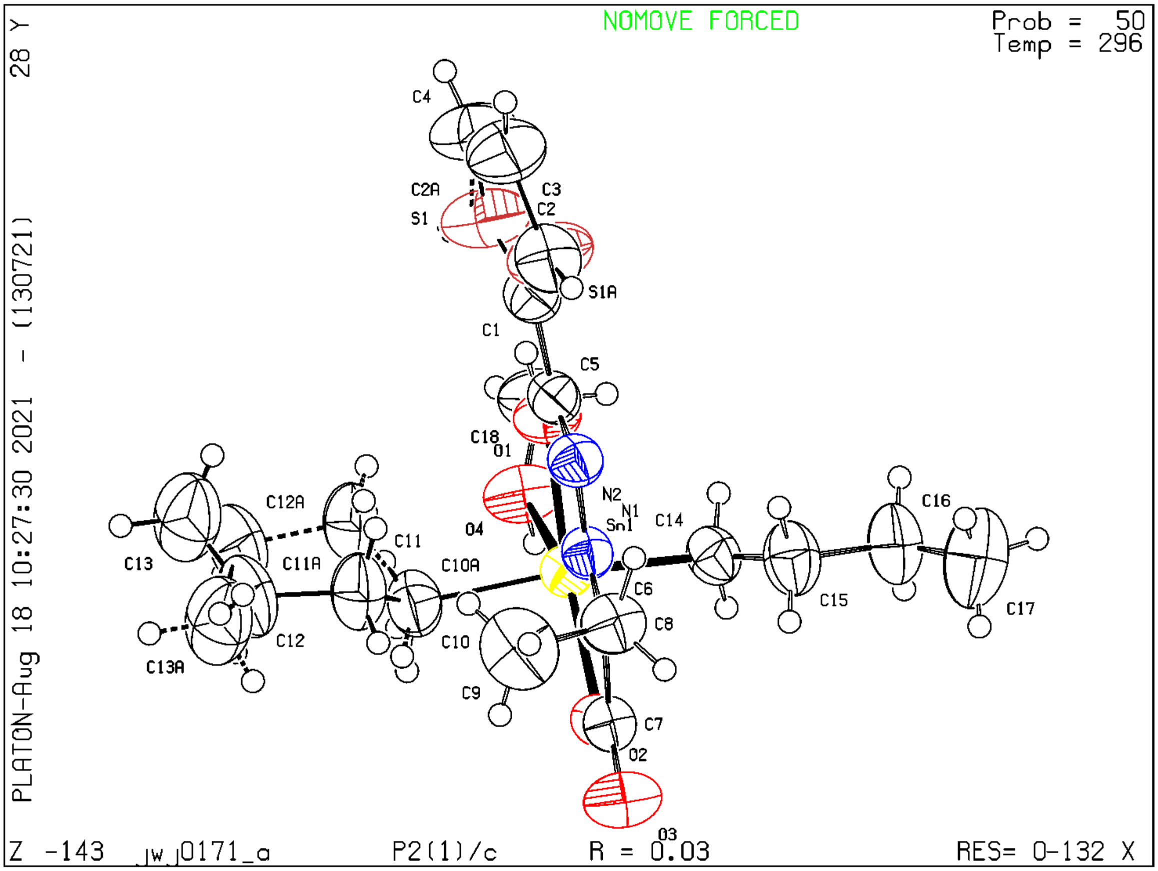

Supplement: Supplementary file 1 [file ijms-22-13525-s001.zip › CIF.zip!/9b-checkcif.pdf]
